# Mechanistic insights for the preparation of improved metal–fluoride based radioimaging agents

**DOI:** 10.1039/d6sc06081a

**Published:** 2026-09-22

**Authors:** Victoria K. Greenacre, Rhys P. King, John M. Dyke, Gillian Reid

**Affiliations:** a School of Chemistry and Chemical Engineering, University of Southampton Southampton SO17, 1BJ UK v.k.greenacre@soton.ac.uk jmdyke@soton.ac.uk G.Reid@soton.ac.uk

## Abstract

Metal–fluoride complexes incorporating fluorine-18 are advancing PET imaging, and creating new opportunities for Total Body PET and theranostics, but the factors governing metal–chelator selection, radiofluorination mechanisms, and hydroxide competition remain unclear. Although several Al–^18^F tracers based on NOTA and NODA chelators have reached clinical use, mechanistic understanding is limited and has hindered both extension to other metals and chelators and understanding of the relative merits of “one-pot” (radio)fluorination protocols *vs.* late-stage (radio)fluorination of pre-formed complexes and the design criteria to optimise (radio)metal/^18^F tracers for theranostics. Here, density functional theory (DFT) calculations are used to investigate F^−^/Cl^−^ and OH^−^/Cl^−^ substitution in the archetypal [MCl(Bn-NODA)] (M = Al, Ga). These reactions proceed through a concerted one-step pathway involving temporary dissociation of a carboxylate arm and formation of a transient ionic intermediate, as well as a two-step pathway. Hydroxide substitution is exothermic, favouring formation of [M(OH)(Bn-NODA)], and provides insight into hydroxide competition during radiolabelling. Parallel DFT studies reveal how OH^−^ influences [MF(Bn-NODA)] and radiolabelling efficiency. The computational results align with experimental observations of rapid, late-stage ^18^F radiolabelling of pre-formed gallium complexes under mild conditions and provide design principles for developing new metal–fluoride radiotracers for imaging and therapy. More broadly, the study highlights the value of computational screening in guiding the development of a wider range of metal–fluoride-based agents for imaging and therapy.

## Abstract 2

DFT studies of metal complexes relevant to ^18^F PET tracers reveal that both the metal and precise chelator identities govern halide exchange, hydrolysis and stability, providing valuable insights for the design of new M–^18^F radiopharmaceuticals.

## Graphical abstract

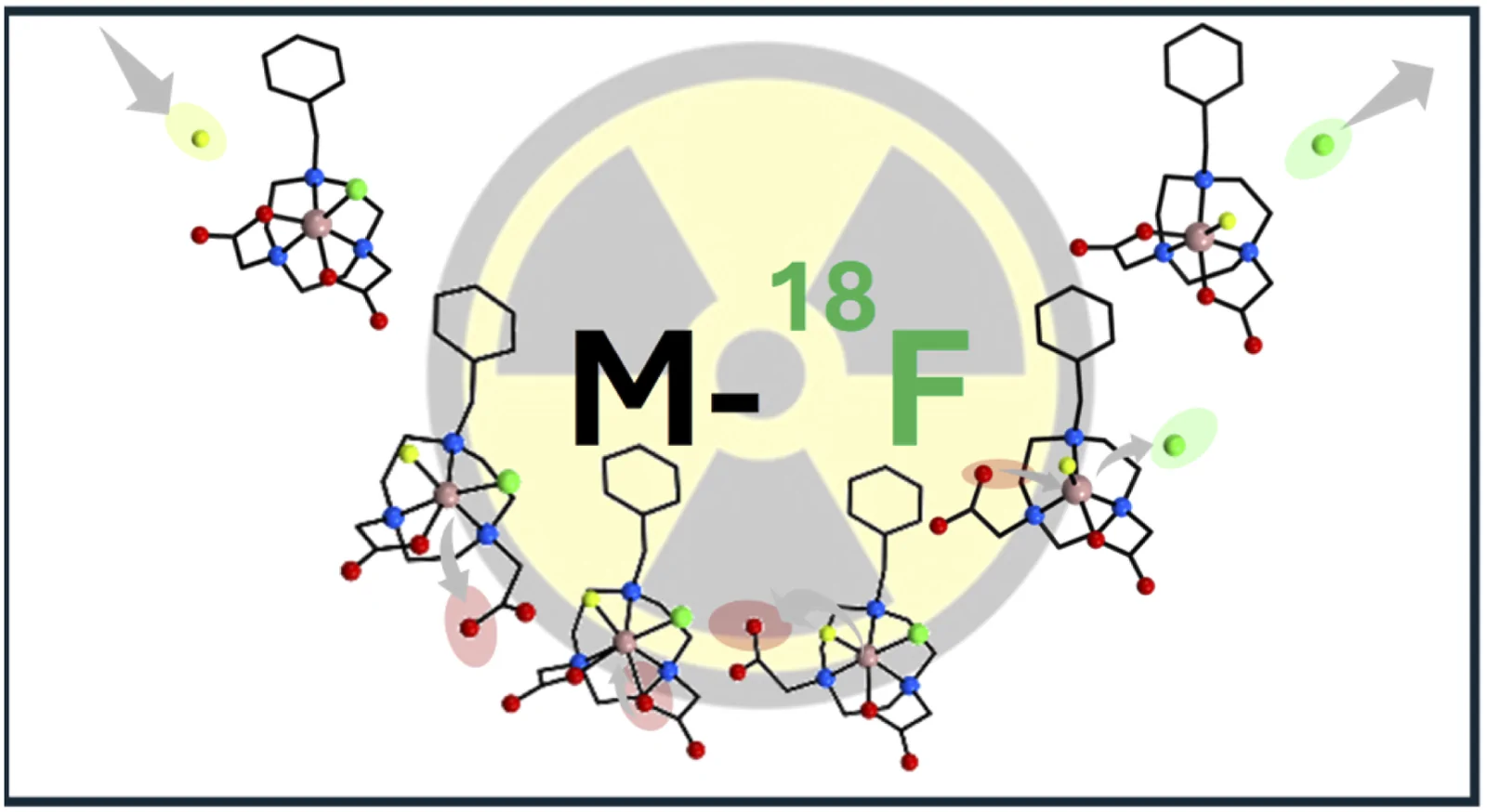

## Introduction

Positron emission tomography (PET) is a non-invasive imaging method used widely in medicine for diagnosing and monitoring various diseases and conditions. Fluorine-18 (^18^F, *t*_1/2_ = 109 min) is the most widely used positron-emitting isotope for PET, due to its favourable decay characteristics, short half-life, high positron yield and low positron energy, allowing high spatial resolution.^1–3^ [^18^F]FDG (FDG = fluorodeoxyglucose) is the most widely used PET imaging agent in the clinic, although it lacks selectivity or disease specificity. The development of a range of ‘[^18^F]AlF’ based PET tracers by McBride and co-workers^4–7^ offered a new generation of highly selective PET tracers that combine the favourable characteristics of fluorine-18, with the selectivity achieved by incorporating a small peptide to the metal-bound chelator.^8–10^ This approach draws on well-established methods in radiometal-based imaging and therapeutics, enabling high specificity for targeting diseased organs and receptors for ^18^F-imaging in PET, while reducing the required radiochemical dose.^11,12^ The advent of Total Body PET (TBP) has increased the demand further, requiring the development of highly selective PET tracers to enable simultaneous monitoring of multiple diseases and metabolic processes in patients.^13–15^

While several kit-based ‘[^18^F]AlF’ PET tracers are now in clinical use,^16^ with clear advantages over [^18^F]FDG through targeted binding of the tracer to disease sites, the principles governing (trivalent) metal ion and chelator selection, as well as optimal radiofluorination strategies, remain poorly understood. Improved matching of metal ions and macrocyclic chelators, considering factors such as ionic radius, Lewis acidity, complex charge and how the specific arrangement of the anionic donor groups at the metal influence the incoming fluoride ion, as well as hard–soft acid–base characteristics, could enable new theranostic agents that combine ^18^F and radiometals within a single construct, while also facilitating milder radiolabelling conditions. A detailed mechanistic understanding of the formation (and hydrolysis) of the metal fluoride tracer complexes is therefore required for the rational design and optimisation of next generation metal–fluoride-based agents for imaging and therapy. In current ‘[^18^F]AlF’ tracers,^17–20^ the hard Lewis acidic Al^3+^ ion is favoured due to its high Al–F bond dissociation energy (BDE) in ‘AlF^2+^’ (∼159 kcal mol^−1^)^21^ and ability of N/O-donor chelators (carrying the bioconjugate) to stabilise the [^18^F]AlF moiety *in vivo*. The reported method for preparation of ‘[^18^F]AlF’ tracers often involves a ‘one-pot’ reaction of AlCl_3_·6H_2_O with [^18^F]F^−^ in acetate buffer (pH ∼4), followed by heating (110 °C) to promote coordination to a pentadentate NOTA or NODA macrocycle carrying a small peptide, forming a stable [^18^F]AlF–chelate–peptide complex (Scheme 1),^4–7,11^ although this may not be optimal. However, this approach is highly pH-dependent, with low pH favouring HF formation, which can in turn produce the very strongly H-bonding bifluoride (HF_2_^−^) ion, and high pH leading to aluminium hydroxide precipitation, both of which reduce radiochemical yields.^22,23^ Consequently, alternative radiolabelling strategies, including lower-temperature methods and the use of acyclic chelators, are being actively explored.^24^

**Scheme 1:**
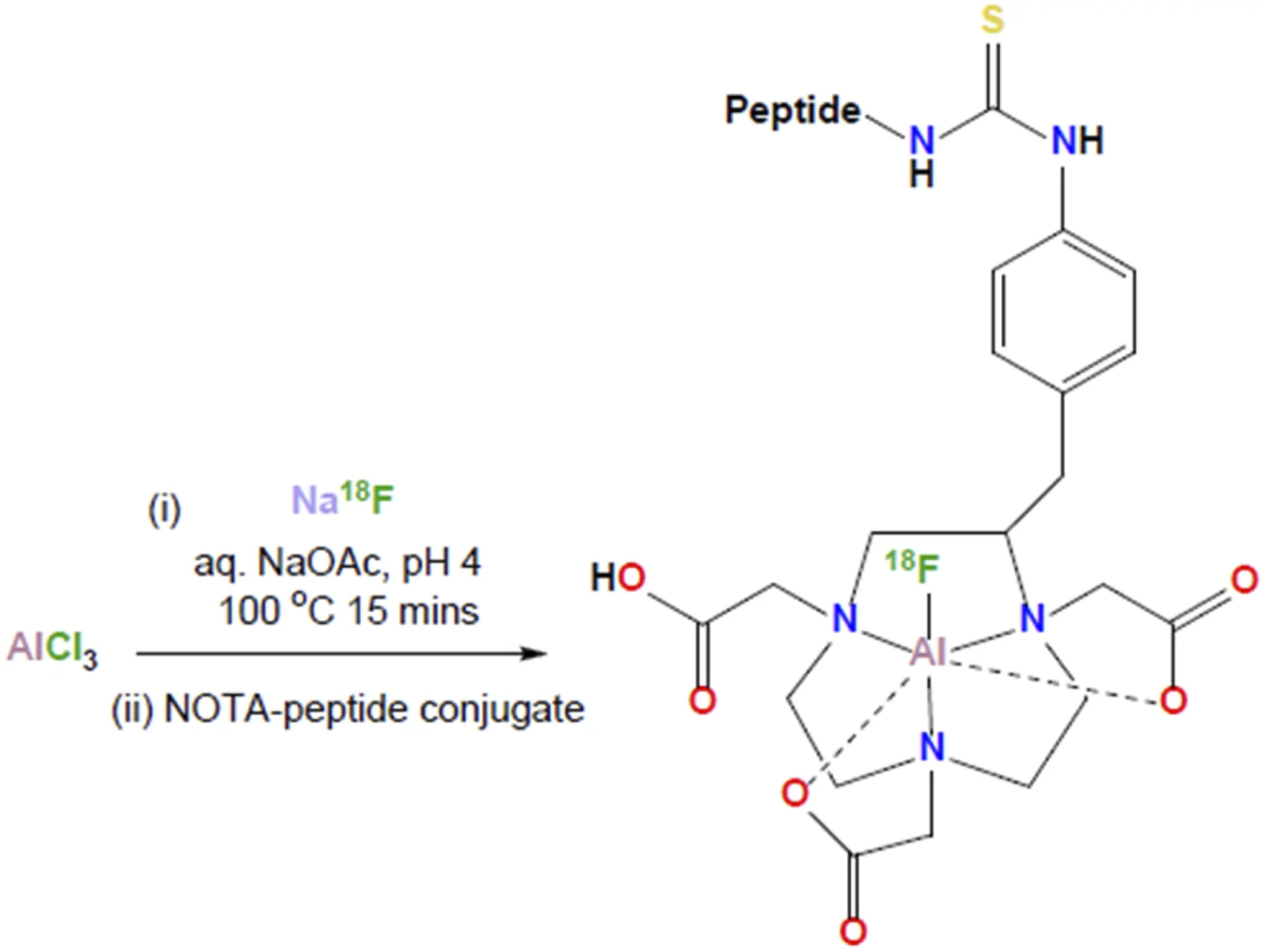
Showing typical conditions for preparation of an [^18^F]-AlF PET tracer, from ref. 4.

Consideration of other trivalent metals (M) with high fluoride affinity has stimulated a growing body of research on alternative radiofluorinated metal–chelate complexes of the form [M^18^F(L)] as radiotracers for ^18^F-based PET imaging. Typically, M = Ga(iii),^25–30^ Fe(iii),^31,32^ In(iii),^33^ or Sc(iii),^34^ while L is a functionalised triaza-macrocycle selected to confer high thermodynamic stability and produce kinetically inert complexes that are stable in competitive media such as serum and phosphate-buffered saline (PBS). Current work is exploring development of potential theranostic agents, by combining ^18^F PET imaging with a therapeutic radionuclide, *e.g.*^44/47^Sc or ^177^Lu,^35,36^ (*e.g.*Scheme 2). In these systems the radiofluorine can be introduced in the final step of the synthesis by the addition of aqueous [^18^F]F^−^ to a solution containing an appropriate pre-formed chelate complex, *e.g.* [GaCl(Bn-NODA)],^27^ [GaCl(Bn-NODP)]^28^ and [M′X_3_(BnMe_2_-tacn)] (M′ = Al, X = Cl;^37^ M′ = Ga, X = Cl or F;^25,26^ M′ = Fe, X = F)^31,32^ (Scheme 3), which allows good control of the coordination chemistry, reducing the likelihood of forming insoluble metal hydroxide precipitates. Introducing the radiofluorine at the final step is also beneficial due to its short *t*_1/2_. In the Ga^3+^ and Al^3+^ systems, the higher affinity of the M^3+^ for F^−^ over Cl^−^, enables ready introduction of the fluoride in (partially) aqueous solution. Significantly, using pre-formed chelate complexes can also allow the key radiofluorination step to be performed under less stringent pH constraints and at lower temperature (room temperature or with brief heating 80 °C 10 min^−1^) compared to the typical ‘[^18^F]AlF’ conditions.

**Scheme 2:**
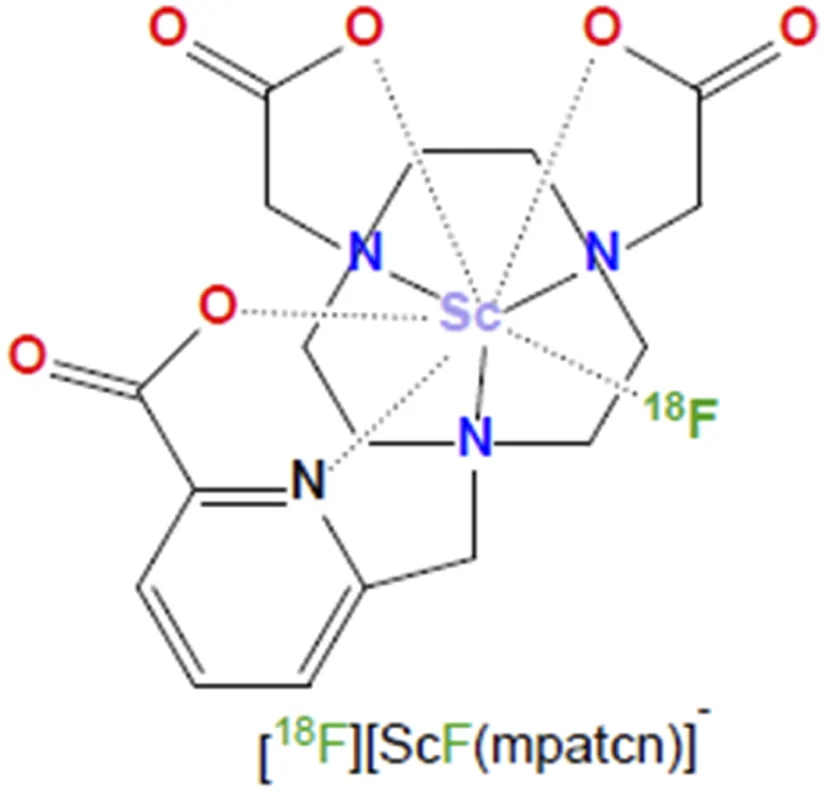
Showing an example of a Sc–^18^F complex developed towards novel ^18^F/^44/47^Sc theranostic pairs, from ref. 34.

**Scheme 3:**
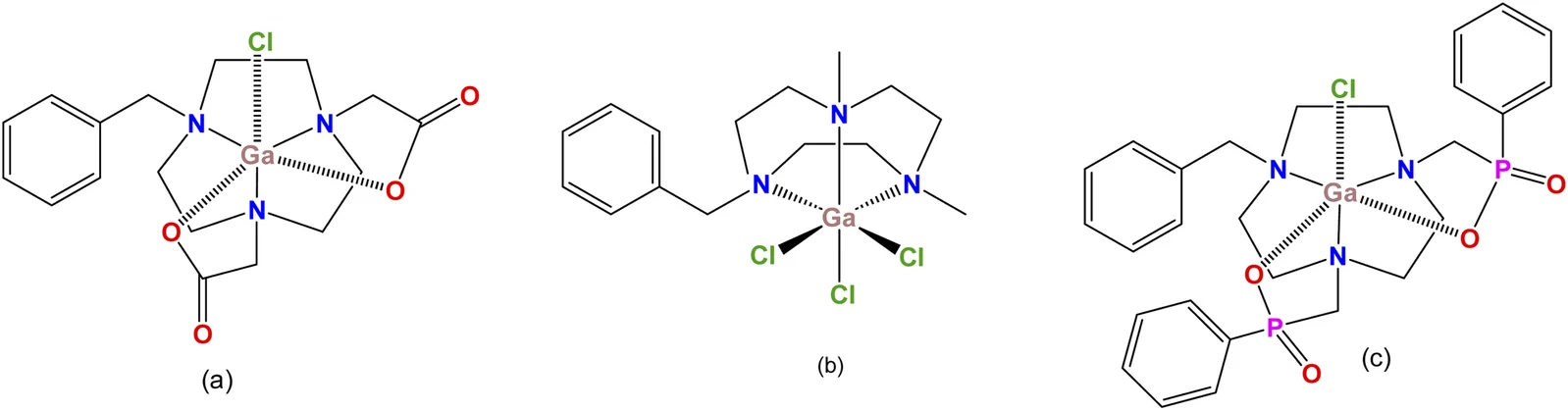
The structures of (a) [GaCl(Bn-NODA)] from ref. 27; (b) [GaCl_3_(BnMe_2_-tacn)] from ref. 25 and (c) [GaCl(Bn-NODP)] from ref. 28.

It is clear from the available experimental data that both the choice of metal and multidentate chelator are key in determining the radiofluorination conditions necessary and the tracer stability under physiological conditions. For example, whereas [^18^F][AlF(Bn-NODA)] remains stable at pH 7.4,^8^ [^18^F][GaF(Bn-NODA)] liberates [^18^F]F^−^ under the same conditions.^27^ However, the complexes [^18^F][GaF_3_(BnMe_2_-tacn)]^25,26^ and [^18^F][GaF(Bn-NODP)]^28^ retain their fluoride ligands at this pH. This suggests that fluoride retention is dependent on the pendant function in the multidentate chelator (*i.e.* alkyl, carboxylate, phosphinate). These observations suggest that defluorination is governed not simply by metal–fluoride bond strength but by ligand-assisted pathways, such as cleavage of the metal–carboxylate bonds, as well as exchange with water, hydroxide or other halide ions.^38^

Despite growing interest in metal–macrocyclic platforms for ^18^F radiolabelling, theoretical investigations of the underlying fluorination and defluorination mechanisms remain scarce. One DFT study compared two synthetic routes to ‘[^18^F]AlF’ tracers: (i) fluoride binding prior to chelation and (ii) fluorination of a pre-formed Al–chelate complex. Although the calculations suggested that (ii) fluorination of the pre-assembled Al-NOTA^R^ complex is energetically preferred (NOTA^R^ is a modified NOTA ligand carrying a –CH_2_C(O)Me pendant group in place of one of the carboxylate groups),^39^ the analysis was limited to stationary-point energies and did not consider transition-states or use IRC calculations to link the different species on the proposed reaction pathway. Consequently, mechanistic conclusions remain tentative, highlighting the need for more comprehensive computational studies that explicitly characterise intermediates, transition states, and reaction energetics in metal–fluoride radiolabelling systems.

To address this, we selected the [MX(Bn-NODA)] series (M = Al, Ga; X = Cl, F) as model systems because their fluorination behaviour and radiochemical stabilities have been experimentally characterised and show clear metal-dependent differences. In addition, X-ray crystallographic and spectroscopic data are available for most members of the series, providing the precise coordination environments and geometric parameters, as well as establishing the specific isomer present in each case (Scheme 4).^8,27^ This provides a strong platform for investigating the energetics and mechanisms of halide substitution and contributing to developing design principles for improved metal–chelate carriers for fluorine-18 PET imaging.

**Scheme 4:**
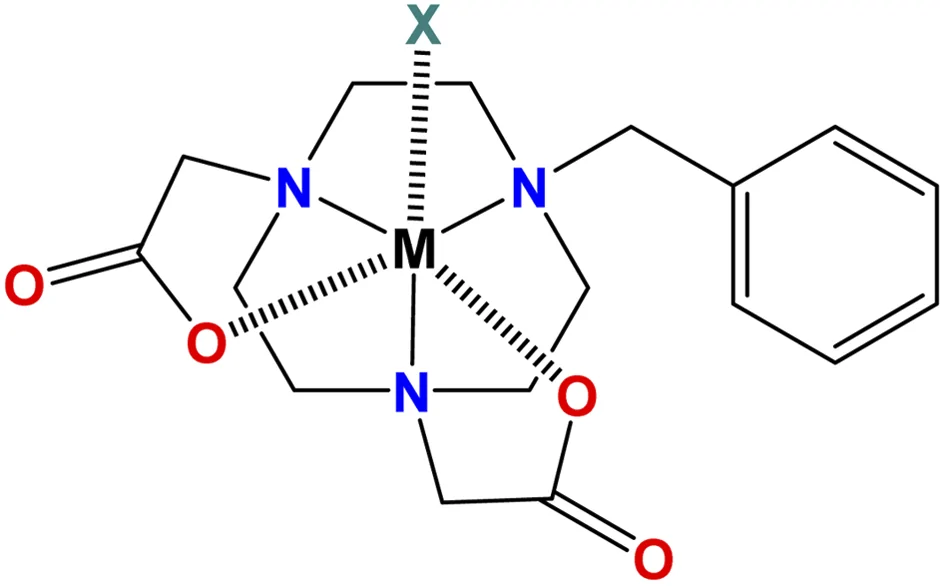
Showing the isomer present in [MX(Bn-NODA)] (M = Al, X = F; M = Ga, X = Cl, F).^8,27^

Here, we present a detailed DFT investigation of the energetics and mechanisms of halide and hydroxide substitution reactions in the [MX(Bn-NODA)] series (M = Al, Ga; X = Cl, F). By examining Cl^−^/F^−^, Cl^−^/OH^−^, and F^−^/OH^−^ exchange pathways, the study investigates the influence of both the metal ion and the macrocyclic ligand on radiolabelling, defluorination, and hydrolysis processes, providing molecular-level insight relevant to the design of next-generation metal-^18^F PET radiotracers.

## Results and discussion

Three reactions (1)–(3) were investigated for both Al and Ga using Density Functional Theory (DFT) calculations, with IRC calculations performed to confirm that for each reaction the complexes, intermediates and transition states identified are genuinely linked on a single energy profile.

1 F^−^ + [MCl(Bn-NODA)] → [MF(Bn-NODA)] + Cl^−^

2 OH^−^ + [MCl(Bn-NODA)] → [M(OH)(Bn-NODA)] + Cl^−^

3 OH^−^ + [MF(Bn-NODA)] → [M(OH)(Bn-NODA)] + F^−^

The initial focus was on reaction (1), with the objective to use DFT calculations to establish, for each metal (Ga and Al), the reaction mechanism and energetics. Reaction (2) is an OH^−^/Cl^−^ exchange reaction, and a comparison of the mechanisms and energetics of reactions (1) and (2)*via* DFT calculations will give information on how OH^−^ competes with F^−^, affecting the overall yield of the fluorinated complexes, [MF(Bn-NODA)], for both Ga and Al, while reaction (3) probes the decomposition (defluorination) of [MF(Bn-NODA)] under alkaline conditions for M = Ga and Al. This is important, as the higher Lewis acidity of Al^3+^ compared to Ga^3+^ is expected to confer greater pH stability on [AlF(Bn-NODA)] compared to [GaF(Bn-NODA)]. The presence of the solvent (water) was included using the polarizable continuum model (PCM) in the calculations.^40^ In the case of reaction (1) for M = Ga, calculations were carried out both in the absence of a solvent (*i.e.* gas-phase) and in the presence of the solvent (water) in order to establish the effects of solvation. The DFT calculations used the BP86 functional^41^ augmented with the Grimme correction for dispersion^42^ using 6-311++G(d,p) basis sets with GAUSSIAN 16,^43^ and AIM calculations were carried out for reaction (1) for M = Ga and Al in solution and M = Ga in the gas-phase.^44–46^ More details of these calculations are given in the Computational details section below. The basis set was selected for its ability to calculate gas-phase electron affinities (EAs) of atomic fluorine, chlorine and the hydroxyl radical,^47–51^ as these provide a measure of the ability of the BP86-D3/6-311++G(d,p) level of calculation to compute reliable relative energies of the reactions considered. Justification of the choice of this basis set and EAs of F, Cl and OH calculated with other basis sets and functionals are given in the SI (Section 11).

### Computed structures of [GaX(Bn-NODA)]·2H_2_O (X = Cl, F) and [AlF(Bn-NODA)]·2H_2_O

Since single crystal X-ray structural data are available for [GaX(Bn-NODA)]·2H_2_O (X = F, Cl) from our earlier work^27^ and for [AlF(Bn-NODA)]·2H_2_O from ref. 8. BP86-D3/6-311++G(d,p) geometry optimization calculations were performed on [GaX(Bn-NODA)] (X = F, Cl) and [AlF(Bn-NODA)], as well as these complexes with two water molecules added, to give initial structures for optimization with H_2_O in the positions they adopt in the crystal lattice. For the unsolvated complexes, the computed structures are compared with the crystal structures (Fig. 1, SI Sections S1a and S2a), and the computed values of the key bond lengths and some key bond angles involving the metal are compared with the corresponding experimental parameters in Table 1. These show that the metal is six-coordinate, bonded to two O atoms, one N atom (N_*trans*_) lying *trans* to the M–X bond and two other N atoms. There is good agreement between the experimentally determined and computed values both without and with added H_2_O. However, unsurprisingly, the positions of the water molecules in the crystal structures are not replicated in the [MX(Bn-NODA)] calculations when these are added, since their positions in the crystals are determined by the 3D lattice. It is notable that on comparing the experimental bond lengths in Table 1 for [GaF(Bn-NODA)] and [AlF(Bn-NODA)], the M–N bonds are slightly shorter for M = Al by ∼0.04 Å, but the M–F and M–O bonds are shorter by ∼0.10 Å. These reduced bond lengths are probably due to the higher Lewis acidity of Al^3+^ compared to Ga^3+^ as mentioned previously.^27^ This trend is also observed in the computed bond lengths for these compounds.

**Fig. 1:**
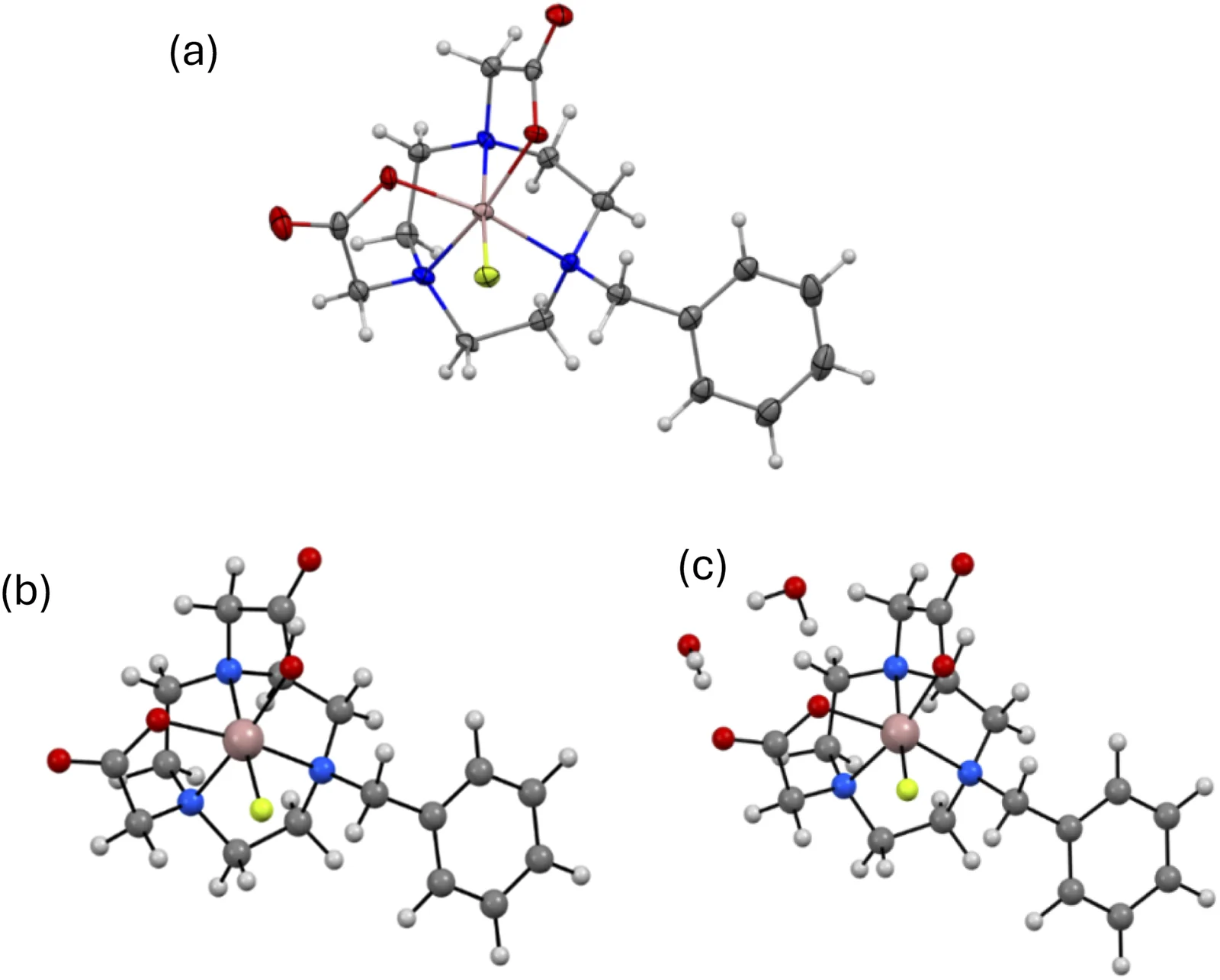
Geometries of [GaCl(Bn-NODA)], (a) crystal structure (with lattice water molecules omitted) from ref. 27, (b) optimised geometry of the [GaF(Bn-NODA)] molecule in the gas phase, and (c) optimised geometry of [GaX(Bn-NODA)] molecule with two H_2_O molecules added; all calculations were performed at the BP86-D3/6-311++G(d,p) level.

**Table 1:** Key computed geometric parameters for [GaX(Bn-NODA)] (X = F, Cl) and [AlF(Bn-NODA)] compared to experimental X-ray crystallographic data (all calculations were performed at the BP86-D3/6-311++G(d,p) level in the gas phase)

Bond distances/Å; bond angles/°	Experimental (X-ray) M = Ga: ref. 20; M = Al: ref. 8	Calculated (isolated complex)	Calculated (with lattice H_2_O – see text)
**[GaCl(Bn-NODA)]·2H**_**2**_ **O**			
Ga–Cl	2.266(4)	2.262	2.305
Ga–O	1.888(13)	1.932	1.989
	2.010(11)	1.966	2.038
Ga–N	2.094(13)	2.253	2.164
	2.147(13)	2.197	2.038
Ga–N(*trans*)	2.122(11)	2.236	2.157
N–Ga–O	165.2(4)	159.482	163.977
	163.6(5)	158.539	163.938
Cl–Ga–N(*trans*)	177.7(4)	172.714	177.092

**[GaF(Bn-NODA)]·2H**_**2**_ **O**			
Ga–F	1.821(2)	1.846	1.873
Ga–O	1.940(3)	1.928	1.975
	1.980(3)	1.957	2.053
Ga–N	2.082(4)	2.229	2.137
	2.146(3)	2.187	2.119
Ga–N(*trans*)	2.111(3)	2.221	2.144
N–Ga–O	165.73(13)	161.124	164.720
	165.15(14)	160.526	164.261
F–Ga–N(*trans*)	175.41(12)	166.775	177.456

**[AlF(Bn-NODA)]·2H**_**2**_ **O**			
Al–F	1.7091(14)	1.746	1.748
Al–O	1.8856(15)	1.848	1.834
	1.8444(18)	1.873	1.911
Al–N	2.050(2)	2.124	2.163
	2.1124(16)	2.170	2.119
Al–N(*trans*)	2.0862(16)	2.159	2.152
N–Al–O	165.70(8)	162.772	163.014
	165.62(8)	162.323	164.183
F–Al–N(*trans*)	174.28(7)	167.935	168.136

### Mechanisms and energetics of reactions (1)–(3) for M = Ga or Al

The six reactions involving halide (or hydroxide) substitution at M = Ga or Al investigated in this work, (1)–(3) above, are listed in Table 2. Each reaction could take place *via* two overall processes, a 2-step dissociative process, reactions (4a) and (4b), or a reaction on the same potential energy surface 1-step process, reaction (4c):

4a 2-step substitution: [MY(Bn-NODA)] → [M(Bn-NODA)]^+^ + Y^−^

4b X^−^ + [M(Bn-NODA)]^+^ → [MX(Bn-NODA)]

4c 1-step substitution: X^−^ + [MY(Bn-NODA)] → [MX(Bn-NODA)] + Y^−^

**Table 2:** Computed reaction energies and standard free energies for the 2-step substitution (reactions (4a) and (4b) in kcal mol^−1^)

Reaction X^−^ + MY(Bn-NODA)	Reaction (4a) Δ*E* (Δ*G*^*ϕ*^_f(298 K)_) solution (H_2_O)	Reaction (4b) Δ*E* (Δ*G*^*ϕ*^_f(298 K)_) solution (H_2_O)	Dissociation of [MY(Bn-NODA)] to the neutral products M(Bn-NODA) + Y Δ*E* (Δ*G*^*ϕ*^_f(298 K)_) solution (H_2_O)
**F^−^+ [MCl(Bn-NODA)]** a			
M = Ga	21.6 (12.8)	−31.0 (−23.0)	115.0 (102.7)
M = Al	25.7 (16.1)	−44.4 (−34.8)	132.5 (121.0)

**OH^−^+ [MCl(Bn-NODA)]**			
M = Ga	21.6 (12.8)	−36.7 (−26.3)	115.0 (102.7)
M = Al	25.7 (16.1)	−46.1 (−33.5)	132.5 (121.0)

**OH^−^+ [MF(Bn-NODA)]**			
M = Ga	31.0 (22.9)	−36.7 (−26.3)	125.5 (112.1)
M = Al	44.5 (34.8)	−46.1 (−33.5)	133.2 (118.6)

a Values for M = Ga in the gas phase: (4a) 132.9 (123.7); (4b): −158.7 (−154.3); dissociation: 113.0 (101.0).

The reaction energies (Δ*E*) and free energies (Δ*G*^*ϕ*^_f,298 K_) for (4a) and (4b) involving the 2-step process could be calculated straightforwardly. However, for the 1-step process, (4c), the potential energy surface needed to be investigated to establish the stationary points and hence reveal the detailed reaction mechanism. Initially, it was anticipated that each reaction (4c) would proceed from the reactants to a reactant complex (RC) then *via* a transition state (TS) to a product complex (PC) and on to the products. However, in practice it was found that, in each case, the reaction pathway is more complicated than this since ionic complexes (ICs) of the type [MXY(Bn-NODA)]^−^ need to be considered as reaction intermediates. In each case, searches of the potential energy surface showed that each reaction proceeds *via* two ionic complexes (IC1 and IC2). From the reactants, a reactant complex (RC) is formed which converts to IC1*via* a transition state (TS1). IC1 then converts to IC2*via* another TS (ICts), and IC2 converts to a product complex PC*via* another TS (TS2). The PC then connects to the products (Fig. 2). Both the 2-step and 1-step processes were investigated in this work in solution (water). The F^−^ + [GaCl(Bn-NODA)] → [GaF(Bn-NODA)] + Cl^−^ substitution reaction was also investigated in the gas-phase to allow a comparison to be made between the gas-phase and solution results. The computed reaction energies and standard free energies of reactions (4a) and (4b) for the six reactions studied are summarised in Table 2, and the reaction energies and standard free energies of the stationary points located for reaction (4c) are listed in Tables 3–6. In all cases, reaction (4a) is endothermic, and reaction (4b) is exothermic and barrierless. The rate determining step of (4a) + (4b) is (4a), *i.e.* the reaction rate in the 2-step reaction does not depend on the concentration of the nucleophile (F^−^ or OH^−^); it depends only on [MY(Bn-NODA)].

**Fig. 2:**
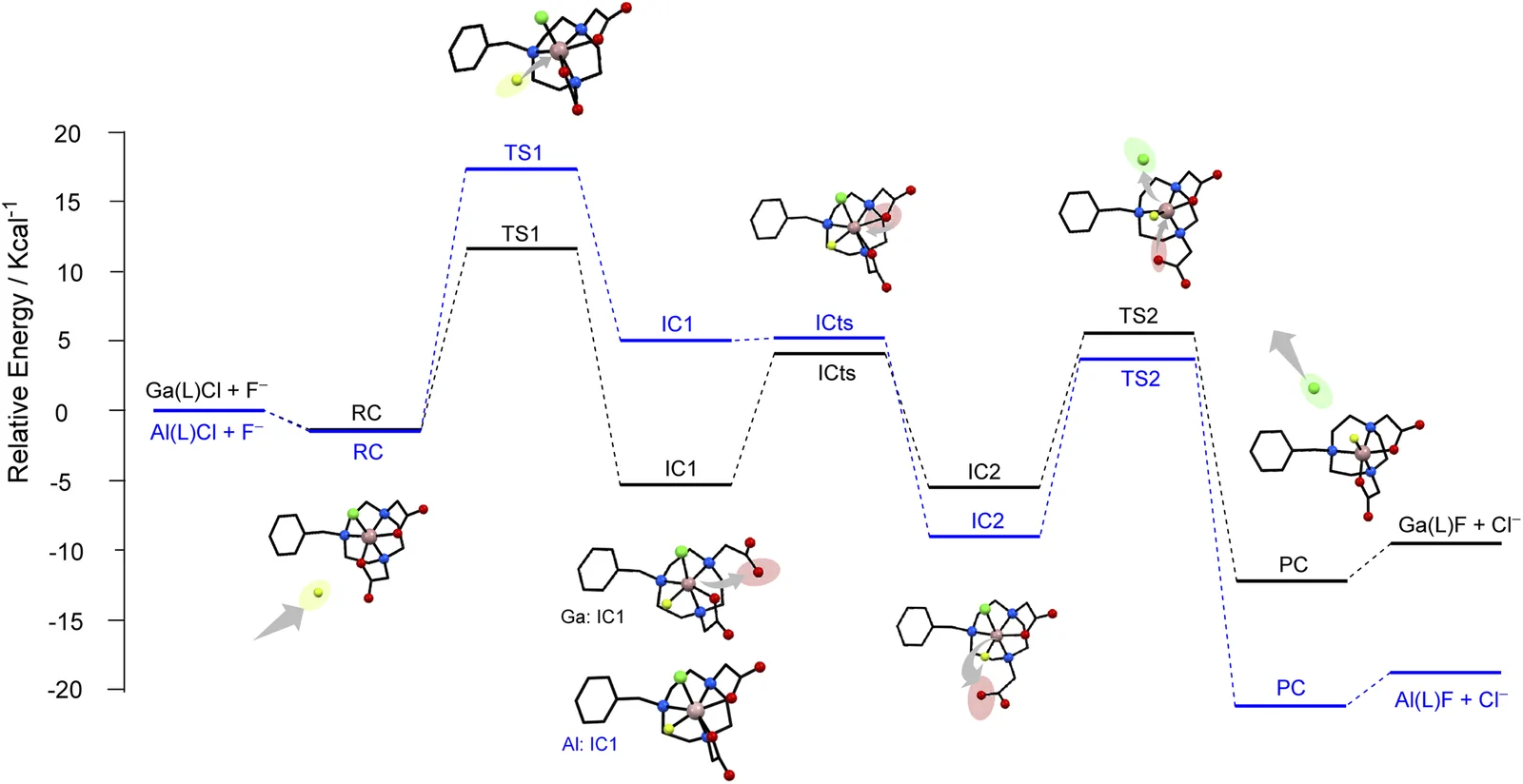
Overlay of the schematic potential energy diagrams for the reactions F^−^ + [GaCl(L)] in solution (water) (black) and F^−^ + [AlCl(L)] in solution (water) (blue) (L = Bn-NODA). Values were computed at the BP86-D3/6-311++G(d,p) level.

**Table 3:** Relative energies (Δ*E*, kcal mol^−1^) of the stationary points on the potential energy surface for the F^−^ + [MCl(Bn-NODA)] reactions (M = Ga and Al), located at the BP86-D3/6-311++G(d,p) level. (Relative Δ*G*^*ϕ*^_f(298 K)_ values are in brackets) (reaction (4c))

Stationary point	Δ*E*, kcal mol^−1^ solution (H_2_O) M = Ga	Δ*E*, kcal mol^−1^ solution (H_2_O) M = Al	Δ*E*, kcal mol^−1^ gas-phase M = Ga
F^−^ + [MCl(Bn-NODA)]	0.0 (0.0)	0.0 (0.0)	0.0 (0.0)
RC	−1.4 (+5.4)	−1.3 (+5.7)	−39.9 (−33.5)
TS1	+11.4 (+18.4)	+17.3 (+24.8)	−20.6 (−15.0)
IC1 [MClF(Bn-NODA)]^−^	−5.3 (+1.9)	+5.0 (+11.3)	−42.2 (−37.7)
ICts	+4.3 (+11.4)	+5.3 (+11.9)	−35.7 (−29.9)
IC2 [MClF(Bn-NODA)]^−^	−5.4 (+2.2)	−9.3 (−2.0)	−37.2 (−31.4)
TS2	+5.6 (+12.3)	+4.0 (+10.7)	−36.6 (−29.6)
PC	−12.2 (−5.2)	−21.3 (−14.5)	−63.1 (−59.6)
Cl^−^ + [MF(Bn-NODA)]	−9.4 (−10.2)	−18.7 (−18.7)	−30.9 (−30.6)

**Table 4:** Relative energies (Δ*E*, kcal mol^−1^) of the stationary points on the potential energy surface of the OH^−^ + [MCl(Bn-NODA)] reactions, located at the BP86-D3/6-311++G(d,p) level (relative Δ*G*^*ϕ*^_f(298 K)_ values in brackets) (reaction (4c))

Stationary point	Δ*E*, kcal mol^−1^ solution (H_2_O) M = Al	Δ*E*, kcal mol^−1^ solution (H_2_O) M = Ga
OH^−^ + [MCl(Bn-NODA)]	0.0 (0.0)	0.0 (0.0)
RC	−2.1 (+6.4)	−2.2 (+5.7)
TS1	+19.0 (+27.8)	+12.2 (+21.7)
IC1 [M(OH)Cl(Bn-NODA)]^−^	−13.3 (−1.9)	−13.7 (−2.4)
ICts	−4.8 (+6.6)	0.0 (+10.1)
IC2 [M(OH)Cl(Bn-NODA)]^−^	−14.6 (−3.4)	−15.0 (−4.5)
TS2	−0.7 (+9.2)	−5.0 (+4.9)
PC	−22.8 (−13.6)	−17.8 (−8.5)
Cl^−^ + [M(OH)(Bn-NODA)]	−20.4 (−17.4)	−15.1 (−13.4)

**Table 5:** Relative energies (Δ*E*, kcal mol^−1^) of the stationary points on the potential energy surface of the OH^−^ + [MF(Bn-NODA)] reactions, located at the BP86-D3/6-311++G(d,p) level. (Relative Δ*G*^*ϕ*^_f(298 K)_ values are in brackets) (reaction (4c))

Stationary point	Δ*E*, kcal mol^−1^ solution (H_2_O) M = Al	Δ*E*, kcal mol^−1^ solution (H_2_O) M = Ga
OH^−^ + [MF(Bn-NODA)]	0.0 (0.0)	0.0 (0.0)
RC	−2.3 (+5.1)	−2.2 (+6.2)
TS1	+19.3 (+29.2)	+12.5 (+24.5)
IC1 [M(OH)F(Bn-NODA)]^−^	−12.5 (−0.2)	−14.3 (−1.7)
ICts	−4.3 (+7.7)	−6.0 (+5.6)
IC2 [M(OH)F(Bn-NODA)]^−^	−13.8 (−1.7)	−15.5 (−3.6)
TS2	+18.8 (+30.1)	+7.1 (+16.9)
PC	−3.1 (+7.3)	−7.1 (+3.0)
F^−^ + [M(OH)(Bn-NODA)]	−1.6 (+1.2)	−5.6 (−3.3)

**Table 6:** Computed reaction energies and relative energies of the highest barrier in the reaction profile relative to the reactants (kcal mol^−1^) of reactions (4c) in solution

Reaction^a^	Highest barrier Δ*E* (Δ*G*^*ϕ*^_f(298 K)_)	Overall reaction Δ*E* (Δ*G*^*ϕ*^_f(298 K)_)
**F^−^+ [MCl(Bn-NODA)]**		
M = Ga	TS1: +11.4 (+18.4)	−9.4 (−10.2)
M = Al	TS1: +17.3 (+24.8)	−18.7 (−18.7)

**OH^−^+ [MCl(Bn-NODA)]**		
M = Ga	TS1: +12.2 (+21.7)	−15.1 (−13.4)
M = Al	TS1: +19.0 (+27.8)	−20.4 (−17.4)

**OH^−^+ [MF(Bn-NODA)]**		
M = Ga	TS1: +12.5 (+24.5)	−5.6 (−3.3)
M = Al	TS1/TS2: +19.3 (+29.2)/+18.8 (+30.1)	−1.6 (+1.2)

a Gas phase for M = Ga: highest barrier TS1: −20.6 (−15.0); overall reaction: −30.9 (−30.6).

#### (i) F^−^ + [GaCl(Bn-NODA)] reaction

For the 2-step pathway, Δ*E*(4a) was calculated as 132.9 kcal mol^−1^ (Δ*G*^*ϕ*^_f,298 K_ = 123.7 kcal mol^−1^) for the gas-phase case and 21.6 kcal mol^−1^ (Δ*G*^*ϕ*^_f,298 K_ = 12.8 kcal mol^−1^) for the solution case (in water). The corresponding Δ*E*(4b) values are −158.7 kcal mol^−1^ (Δ*G*^*ϕ*^_f,298 K_ = −154.3 kcal mol^−1^) (gas-phase) and −31.0 kcal mol^−1^(Δ*G*^*ϕ*^_f,298 K_ = −23.0 kcal mol^−1^ (in solution)). The large differences observed between the gas-phase and solution values are due to the ions being more strongly solvated than the neutrals in reactions (4a) and (4b). It is also interesting to compare the [GaCl(Bn-NODA)] dissociation to neutral products ([GaCl(Bn-NODA)] → [Ga(Bn-NODA)] + Cl) with reaction (4a) (dissociation to ionic products). The computed neutral dissociation energies Δ*E* are 113.0 kcal mol^−1^ (Δ*G*^*ϕ*^_f,298 K_ = 101.0 kcal mol^−1^) for the gas-phase case and 115.0 kcal mol^−1^ (Δ*G*^*ϕ*^_f,298 K_ = 102.7 kcal mol^−1^) for the solution case (see Table 2 and its footnote). These values mean that in the gas-phase the neutral dissociation channel is the favoured pathway, whereas in solution (water) the ionic channel is favoured. Similar results were obtained for the other reactions investigated (Table 2).

Schematic potential energy diagrams for the solution and gas-phase cases of reaction (4c) are shown in Fig. 2 and S1d, respectively. It is found that, for the 1-step process (4c), exploration of the reaction surface shows the same stationary points and pathway in both. The values in Table 3 show that on going from the gas-phase to the ‘in solution’ case, all energies of the stationary points relative to the reagents are more positive and the overall reaction is less exothermic in the solvated case; notably the ionic intermediates and the three transition states are at more positive energies. Δ*E* for (4c) in the gas-phase is −30.9 kcal mol^−1^*cf.* −9.4 kcal mol^−1^ in the solvated case. Diagrams of the structures of the species at the stationary points in Fig. 2 and S1d are shown in the SI (Section S1). It is notable that the structures of the ionic complexes IC1 and IC2 are similar in the solution case, differing mainly in the orientation of the two carboxylate groups relative to the F–Ga–Cl unit, while the Ga–F distance is slightly shorter and the Ga–Cl distance is slightly longer in IC2 than IC1.

To understand the non-bonded interactions in each of the species at the stationary points, AIM calculations were performed for F^−^ + [GaCl(Bn-NODA)] (gas-phase and solution) and F^−^ + [AlCl(Bn-NODA)] (solution) (1-step cases); the results are listed in the SI (Table S9(a)–(v)). For the M = Ga solution case, the number of non-bonded interactions located (in brackets) were RC (6), TS1 (8), IC1 (11), ICts (8), IC2 (8), TS2 (8) and PC (6). For the M = Ga gas-phase case, similar structures and numbers of non-bonded interactions were obtained (RC (8), TS1 (8), IC1 (6), ICts (8), IC2 (8), TS2 (9) and PC (5)). However, the RC structure that connects with both the reactants and TS1 is different in the solution compared to the gas-phase case, with the F atom positioned between one of the carboxylate groups and the benzyl group in the solution case, whereas in the gas-phase case it sits between the two carboxylate groups, on the other side of the structure from the benzyl group (Fig. 3). For all the non-bonded interactions listed above (which are H-bonding or van der Waals (vdW) in nature) *ρ* and ∇^2^*ρ* are both positive and small, as expected. For example, in the RC in solution the six non-bonded interactions consist of five H-bonds (H···C, H···O, H···F(2), and H···Cl) and one vdW bond (O···F), whereas for the RC in the gas-phase there are eight non-bonded interactions consisting of seven H-bonds (H···Cl(3), H···F(2), H···C and H···O) and one vdW bond (H···H) (Fig. 3). For M = Al in solution, the RC is structurally very similar to the M = Ga solution case, showing seven non-bonding interactions; it has the same non-bonding interactions as the M = Ga case, with one extra H···Cl interaction. Non-bonded interactions for all the stationary point structures located for F^−^ + [GaCl(Bn-NODA)] in solution and the gas-phase and F^−^ + [AlCl(Bn-NODA)] in solution are shown in the AIM summary in the SI, (Section 9).

**Fig. 3:**
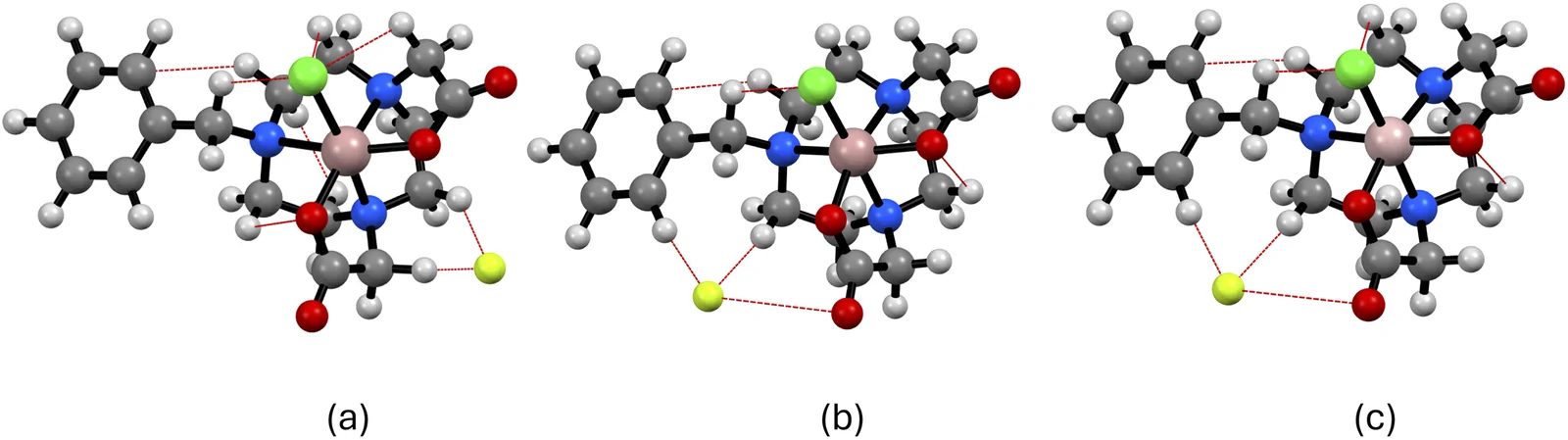
Computed structures of the RC for the reaction F^−^ + [MCl(Bn-NODA)] at the BP86-D3/6-311++G(d,p) level, (a) M = Ga(gas), (b) M = Ga (solution/H_2_O) and (c) M = Al (solution/H_2_O), showing located non-bonding interactions. Atom key: Ga (pink), Cl (green), F (yellow), O (red), N (blue), C (dark grey), H (light grey).

For all the stationary points in Fig. 2 the computed Ga-donor bond distances for the atoms close to gallium, as well as the computed Mulliken charges on these atoms, are listed in the SI (Table S1a, c, f and h). As the reaction proceeds, the Ga–Cl bond lengthens, and the Ga–F bond shortens, as expected, with the negative charge on Cl increasing while that on F decreases.

For M = Ga, in the solution case the three Ga–N bonds show only small changes throughout the reaction. The most striking structural change involves dissociation of one carboxylate pendant arms of the Bn-NODA ligand at each ionic complex, IC1 and IC2. Thus, one Ga–O distance increases from 2.04 Å in the [GaCl(Bn-NODA)] starting complex to 4.33 Å in IC1 (sum of the vdW radii for Ga and O = 3.39 Å),^52^ reduces to 2.23 Å in transition state ICts (re-coordinated) and remains approximately constant at 2.03 Å to IC2, TS2, PC and the products. The second Ga–O bond which has been constant at 1.99 Å increases to 2.37 Å in ICts, increases further to 4.07 Å in IC2 (dissociated), before gradually decreasing to 3.19 Å in TS2, 1.98 Å in the PC and 2.03 Å in the final [GaF(Bn-NODA)] complex. In the ionic complexes, IC1 and IC2, Ga is best described as six-coordinate with an FClN_3_O donor set. The coordination environments calculated for IC1, ICts and IC2 are illustrated in Fig. 4. For the stationary points the charges on the atoms close to Ga are listed and discussed in the SI (Section S8).

**Fig. 4:**
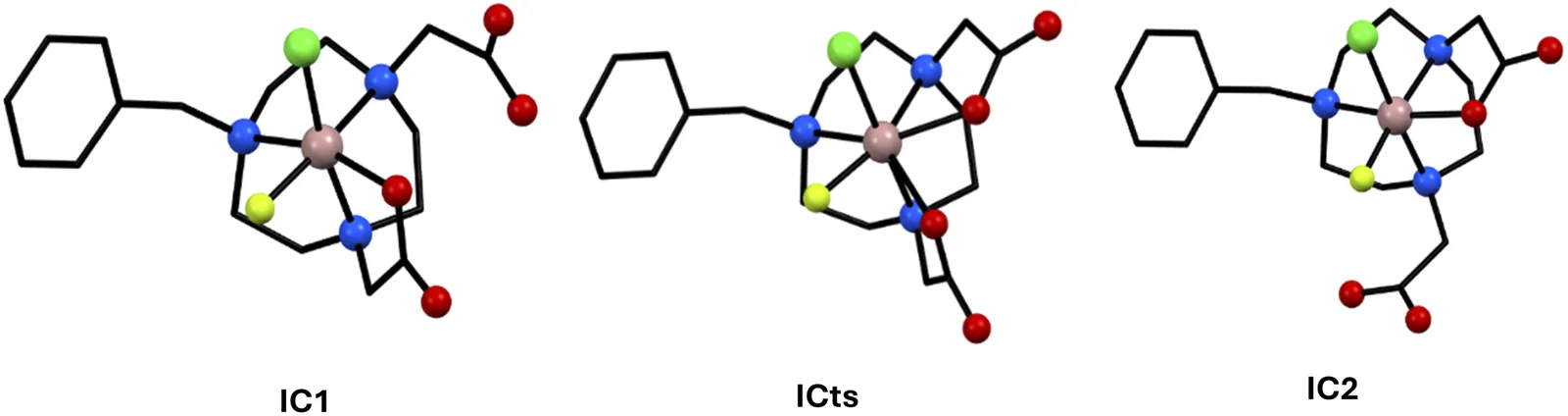
View of the structures calculated for IC1, ICts and IC2 involved in the 1-step substitution reaction of F^−^ with [GaCl(Bn-NODA)] in water; IC1 and IC2 are each six-coordinate with one carboxylate arm dissociated.

The bond length changes for the M = Al solution case are similar, as summarised below, while those for the M = Ga (gas-phase) calculations are in the SI (Section S1).

#### (ii) F^−^ + [AlCl(Bn-NODA)] reaction

For the one-step process, (4c), the overall reaction profile shows the same stationary points as in the gallium analogue (solvated case) (Fig. 2), with two ionic reaction intermediates IC1 and IC2, differing mainly in the orientation of the two carboxylate groups, connected by a TS (ICts). The F^−^ + [AlCl(Bn-NODA)] solvated reaction is more exothermic than for Ga, with Δ*E* values of −18.7 kcal mol^−1^ (Al) compared to −9.4 kcal mol^−1^ (Ga) (Fig. 2 and Table 3), however, the energy barrier *via*TS1 is higher for M = Al (TS1 Δ*E* values: +17.3 kcal mol^−1^ (Al); +11.4 kcal mol^−1^ (Ga)).

For the stationary points shown in the potential energy diagram in Fig. 2, the computed Al–X bond distances for the atoms close to aluminium (X = F and/or Cl, O (2 values), N (3 values)), as well as the Mulliken charges on these atoms, are presented in Table S8a. As in the case for M = Ga (solvated), as the reaction proceeds, the Al–Cl bond elongates and the Al–F bond shortens, while the negative charge on Cl increases and the negative charge of F decreases, and the three Al–N bonds are essentially unchanged throughout the reaction. In contrast, much larger changes are observed in one of the Al–O bonds, increasing from 1.88 Å (coordinated) in [AlCl(Bn-NODA)] to 2.06 Å in IC1, to 2.25 Å in ICts, to 4.07 Å in IC2 (uncoordinated). It then decreases to 3.04 Å in TS2 and to 1.89 Å in the final [AlF(Bn-NODA)] product. The sum of vdW radii for Al and O is 3.36 Å,^51^ hence in IC2 the Al can be regarded as six-coordinate *via* one F, one Cl, three N bonds and one Al–O bond. In contrast, in TS1, IC1, ICts and TS2 the Al can be considered as seven-coordinate, with an ClFO_2_N_3_ donor set. The pattern of negative charges on the O and N atoms throughout the reaction profile follows the same trend as for the Ga analogue (see SI, Section S8).

### Reaction of OH^−^ ion with [MX(Bn-NODA)] (M = Ga, Al; X = Cl, F) in water

The presence of hydroxide ions is expected to have a significant effect on the reaction chemistry for these Lewis acidic metal ions in aqueous solution, both for the fluorination stage and in terms of the stability of the final fluoride complexes at physiological pH. Experimental literature shows that in the case of the Group 13 metal chloro complexes with various tacn based ligands, hydroxide- or oxide-bridged species can form upon exposure to water,^25,53^ although these can be avoided by buffering to lower pH. The Group 13 fluoro complexes are generally much more resistant to hydrolysis. However, understanding the role of hydroxide may provide insights into the stability of fluorine-18 labelled metal–chelate tracer complexes under physiological conditions (pH ∼7.4). To explore this further, we also investigated computationally the effect of adding an OH^−^ ion to the [MX(Bn-NODA)] complexes in water, *via* a similar approach.

#### (iii) OH^−^ + [GaCl(Bn-NODA)] reaction

For the 1-step process (4c) the overall reaction profile shows the same stationary points as shown in Fig. 2 (see Fig. 5 and Table 4). Reaction (4c) has an overall Δ*E* = −15.1 kcal mol^−1^ (Δ*G*^*ϕ*^_f,298 K_ = −13.4 kcal mol^−1^). The corresponding values for F^−^ + [GaCl(Bn-NODA)] are −9.4 kcal mol^−1^ (−10.2 kcal mol^−1^). As observed previously for the other 1-step reactions discussed above, the main potential barrier for OH^−^ + [GaCl(Bn-NODA)] reaction (4c) occurs at TS1 Δ*E* = +12.2 kcal mol^−1^ (Δ*G*^*ϕ*^_f,298 K_ = 21.7 kcal mol^−1^). TS1 for F^−^ + [GaCl(Bn-NODA)] is at Δ*E* = +11.4 kcal mol^−1^ (Δ*G*^*ϕ*^_f,298 K_ = +18.4 kcal mol^−1^). The Ga–X distances and Mulliken charges on the atoms bonded to Ga for the stationary points located on the reaction surface (Fig. 5) are listed in the SI (Section S8b). Similar trends are observed as in the OH^−^ + [AlCl(Bn-NODA)] and the F^−^ + [GaCl(Bn-NODA)] reactions. However, for OH^−^ + [GaCl(Bn-NODA)], unlike the OH^−^ + [AlCl(Bn-NODA)] (where one Ga–O bond increases to larger than the sum of the vdW radii at IC1, ICts, IC2) but similar to the F^−^ + [GaCl(Bn-NODA)] case, one Ga–O bond increases to greater than the sum of the vdW radii of Ga and O (3.39 Å (ref. 51)) at IC1. It then decreases to below this value at ICts and increases again to greater than 3.39 Å at IC2. It has lower values in TS2, PC and [GaCl(Bn-NODA)]. The other Ga–O bond remains approximately constant throughout.

**Fig. 5:**
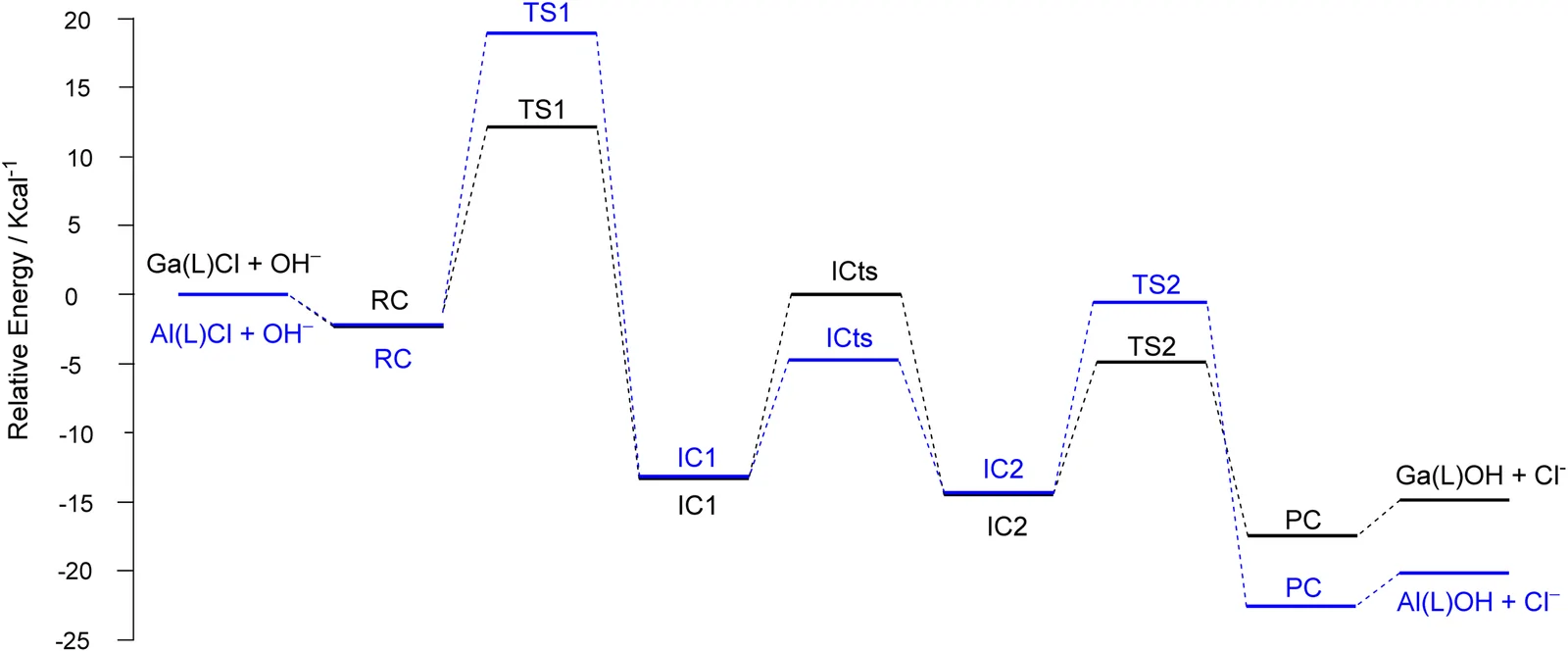
Schematic potential energy diagrams for the reaction OH^−^ + [GaCl(L)] in the solvent (H_2_O) (black) and the reaction OH^−^ + [AlCl(L)] (L = Bn-NODA) in solution (H_2_O) (blue). Values were computed at the BP86-D3/6-311++G(d,p) level. (v) OH^−^ + [MF(Bn-NODA)] (M = Ga, Al).

For the stationary points shown in the potential energy diagram in Fig. 5, the computed Ga–X bond distances for the atoms close to gallium (X = F and/or Cl, O (2 values), N (3 values)) as well as the Mulliken charges on these atoms, are presented in the SI (Section 8(b)).

#### (iv) OH^−^ + [AlCl(Bn-NODA)] reaction

For the 1-step process (4c) the overall reaction profile shows the same stationary points as seen in Fig. 2, in this case with an overall Δ*E* of −20.4 kcal mol^−1^ (Δ*G*^*ϕ*^_f,298 K_ = −17.4 kcal mol^−1^). This compares with −18.7 kcal mol^−1^ (Δ*G*^*ϕ*^_f,298 K_ = −18.7 kcal mol^−1^) for F^−^ + [AlCl(Bn-NODA)] (Tables 3 and 4). The highest potential energy barrier for reaction (4c) occurs at TS1, Δ*E* = 19.0 kcal mol^−1^ (Δ*G*^*ϕ*^_f,298 K_ = 27.8 kcal mol^−1^). This is also the case for the F^−^ + [AlCl(Bn-NODA)] reaction (Δ*E* = 17.3 kcal mol^−1^; Δ*G*^*ϕ*^_f,298 K_ = 24.8 kcal mol^−1^), *i.e.* the barrier to TS1 is greater and the overall reaction more exothermic in the OH^−^ case.

As the OH^−^ + [AlCl(Bn-NODA)] reaction (4c) proceeds, the Al–Cl bond gets longer, and the Al–OH bond gets shorter. The negative charge on Cl increases and the negative charge on the O atom of O–H decreases. As observed for F^−^ + [AlCl(Bn-NODA)], the three Al–N bond lengths change only slightly throughout the reaction, but of the two Al–O(carboxylate) bonds, one increases in length to 3.59, 3.87, 3.72 Å in IC1, ICts, IC2, from 1.88 Å in [AlCl(Bn-NODA)], before decreasing to 2.92 Å in TS2, 1.95 Å in PC and 1.94 Å in [Al(OH)(Bn-NODA)]. The second Al–O(carboxylate) bond remains essentially unchanged (1.92 Å) throughout the reaction. Therefore, in IC1, ICts, and IC2 the Al is effectively six-coordinate with one carboxylate arm dissociated. This elongation of one Al–O bond in IC1, ICts and IC2 to greater than the sum of the atomic vdW radii (3.36 Å)^51^ is more pronounced than in the F^−^ + [AlCl(Bn-NODA)] reaction where only one Al–O bond is significantly elongated to greater than the sum of the vdW radii in IC2.

For the stationary points located, the Al–X bond distances and computed Mulliken charges on atoms close to Al are listed in the SI, Section S8b, and a schematic potential energy diagram for this reaction is shown in Fig. 5.

Similar changes in bond lengths and atomic charge densities were observed for these reactions as observed for the other reactions (4c). In each case, the negative charge on the F atom increases and the negative charge on the O atom of the OH group decreases as the reaction proceeds. The negative charges on the other oxygen atoms show only small changes and the charges on the N atoms decrease steadily throughout the reaction. The Ga–N bond lengths remain approximately constant throughout the reaction. Of the three Ga–O bond distances, in each case one increases to above the sum of the vdW radii (*i.e.* dissociated) for IC1, ICts and IC2, while the others remain lower (coordinated) and approximately constant (Fig. 6).

**Fig. 6:**
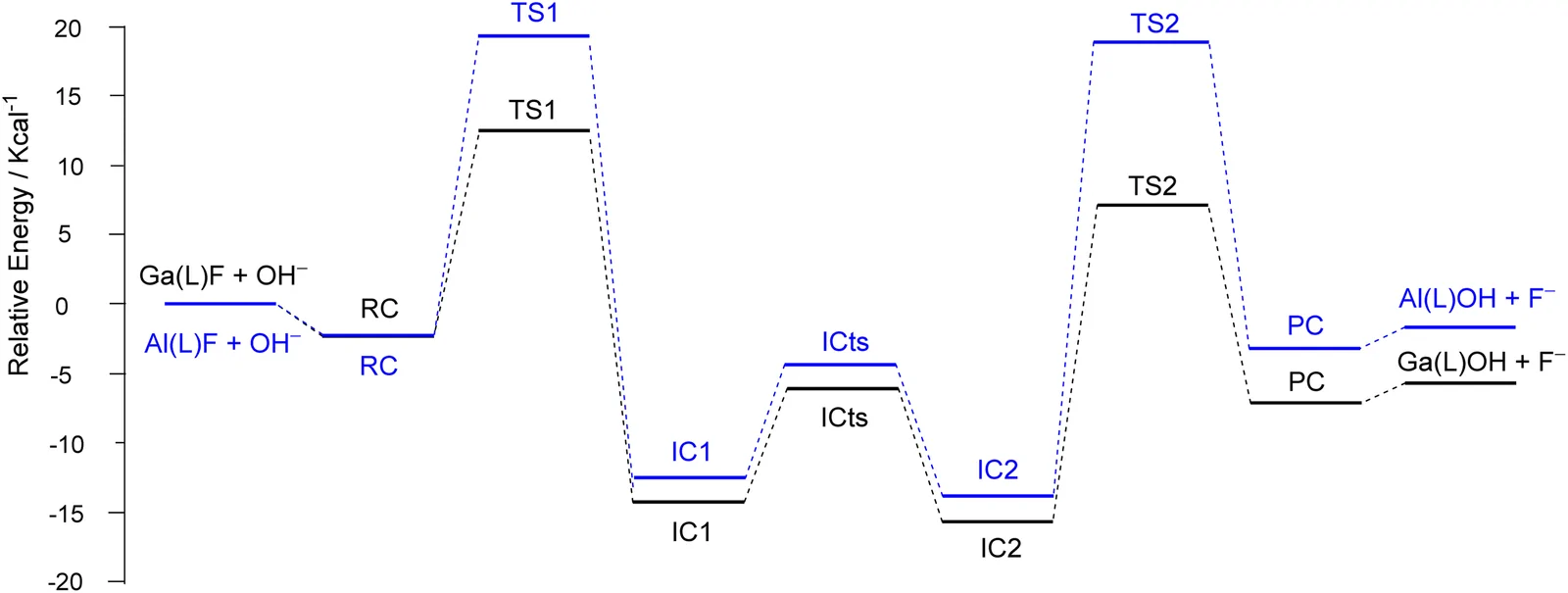
Schematic potential energy diagrams for the reaction OH^−^ + [GaF(L)] in solution (water) (black) and the reaction OH^−^ + [AlF(L)] in solution (water) (blue); (L = Bn-NODA). Values were computed at the BP86-D3/6-311++G(d,p) level.

Inspection of Table 5 and Fig. 6 shows that in the OH^−^ + [GaF(Bn-NODA)] reaction, TS1 clearly has the highest energy, whereas in the OH^−^ + [AlF(Bn-NODA)] reaction the energies of TS1 and TS2 are very similar, with Δ*E* (Δ*G*^*ϕ*^_f,298 K_) values which are very close, +19.3 (+29.2) kcal mol^−1^ (at TS1) and +18.8 (+30.1) kcal mol^−1^ (at TS2), respectively.

As noted, all six reactions investigated are exothermic (Fig. 2, 5, 6 and Tables 3–5). For the F^−^ + [MCl(Bn-NODA)] (M = Al, Ga) reactions this is mainly because the M–F bond in the product is stronger than the M–Cl bond in the reactant, and this effect is larger for Al than Ga (for ionic dissociation, Fig. 2 and Table 3). This can be seen in Table 2, where Δ*E* values for reactions (4a) and (4b) are listed, consistent with expected relative bond energies.^21,54,55^ The same trend is observed for the OH^−^ + [MCl(Bn-NODA)] reactions (Fig. 5 and Table 4), where the M–OH bond in the product is stronger than the M–Cl bond in the reactant, and this effect is larger for Al than Ga (Table 2), as expected.^21,53,54^ In the case of the OH^−^ + [MF(Bn-NODA)] reactions (Fig. 6 and Table 5), the Ga-containing product is lower than the Al-containing product, unlike in the other four reactions, where the Al-containing product lies lower. This is because the M–OH bond in the product is stronger than the M–F bond in the reactant, with this effect being larger for Ga than Al and the M–F bond in the reactant being stronger for Al than Ga, for ionic dissociation, consistent with expected M–X bond energies.^21,53,54^

### Comparison with available experimental evidence

Inspection of the results in Tables 2 and 6 for the two X^−^ + [MX(Bn-NODA)] pathways, the 2-step process (4a) and (4b) and the 1-step process (4c) for the six reactions studied, shows that the rate-determining steps are reaction (4a) for the 2-step process (as reaction (4b) is exothermic in all cases, Table 2) and passage over the highest barrier in the reaction profile of the 1-step process, which is TS1 in all six reactions (for the OH^−^ + Al(L)F reaction the barrier heights for TS1 and TS2 are very similar) (Table 6). Also, on comparing the reaction energies for (4a) (Δ*E* values in column 2 of Table 2) with the values of the highest barrier for (4c) (Δ*E* values in column 2 of Table 6) it can be seen that the highest barriers of (4c) listed in Table 6 are lower than the reaction energies of (4a), indicating that the 1-step process is the dominant reaction pathway for the six reactions in aqueous solution, although comparison of the Δ*G* values indicate that both reactions will almost certainly occur together.

The results of these calculations have the following points of contact with available experimental evidence: in the earlier work^27^ on halide ion exchange it was found that [GaF(Bn-NODA)] could be produced by addition of aqueous F^−^ to an aqueous solution of [GaCl(Bn-NODA)] at or near room temperature (although radiofluorination was more typically performed using NaOAc buffer at pH 4). [^18^F][GaF(Bn-NODA)] was found to be stable under acidic conditions (to pH values of at least 6), but unstable to defluorination at pH ∼7.5 (EtOH/PBS). In contrast, Shetty and co-workers showed that [^18^F][AlF(Bn-NODA)] is stable at pH 4 and at pH 7.4 in HSA (human serum albumin) for at least 2 h,^8^*i.e.* [AlF(Bn-NODA)] is more stable than [GaF(Bn-NODA)] under mildly alkaline conditions. Also, as noted in the Introduction, in the direct preparation of [AlF(Bn-NODA)] from ‘AlF^2+^’ and H_2_Bn-NODA at pH 4 (NaOAc), heating at *ca.* 110 °C is required.^8^ There is also some experimental evidence concerning the mechanism of decomposition of [GaF(Bn-NODA)] under mildly alkaline conditions.^27^ The related fluoride complexes, [GaF_3_(BnMe_2_tacn)] and [GaF(Bn-NODP)] (which do not contain Ga–O(carboxylate) bonds), are stable for several hours at pH 7.4,^25,28^ inferring that neither the Ga–F bonds or the Ga–N(tacn) bonds are inherently unstable under these conditions. Hence it was concluded that the pH-dependent instability of [GaF(Bn-NODA)], causing loss of F^−^, is associated primarily with the Ga–O (carboxylate) bonds in this molecule. This is supported by the results of the DFT calculations in the solution phase reported herein, which suggest that in the one-step process one of the Ga–O(carboxylate) bonds dissociates at some of the intermediate stationary points along the reaction surface, before re-coordinating later as the reaction proceeds. This occurs for all six solution reactions: F^−^ + [GaCl(Bn-NODA)] at IC1 and IC2; F^−^ + [AlCl(Bn-NODA)] at IC2; OH^−^ + [AlCl(Bn-NODA)] at IC1, ICts and IC2; OH^−^ + [GaCl(Bn-NODA)] at IC1 and IC2; OH^−^ + [GaF(Bn-NODA)] and OH^−^ + [AlF(Bn-NODA)] at IC1, ICts and IC2. In effect, the ionic complex, [MXY(Bn-NODA)]^−^, is “breathing” by increasing and decreasing one M–O(carboxylate) bond along the reaction surface to allow the ligand Y^−^ to be replaced by X^−^.

The fact that [AlF(Bn-NODA)] is more stable than [GaF(Bn-NODA)] under mildly alkaline conditions can be explained since for the reaction OH^−^ + [AlF(Bn-NODA)], the computed Δ*E* (Δ*G*^*ϕ*^_f,298 K_) value for the highest barrier, (4c) (Table 6) is higher than that of the analogous OH^−^ + [GaF(Bn-NODA)] reaction.

Also, the observation that the preparation of [AlF(Bn-NODA)] involves heating to ∼100 °C,^8^ whereas [GaF(Bn-NODA)] can be prepared by halide exchange from [GaCl(Bn-NODA)] at room temperature^27^ is consistent with the computed Δ*E* (Δ*G*^*ϕ*^_f,298 K_) values for the highest barrier for F^−^ + [MCl(Bn-NODA)] (M = Al, Ga) (Table 6), which indicate that the values are higher for M = Al, even though this reaction is more exothermic *cf.* for M = Ga.

Table 6 can also be used to infer the relative stability of [MCl(Bn-NODA)] for M = Ga and Al under alkaline conditions, as the higher value for Δ*E* (Δ*G*^*ϕ*^_f,298 K_) for the highest barrier for M = Al means that [AlCl(Bn-NODA)] will be more stable than [GaCl(Bn-NODA)] under alkaline conditions, even although the OH^−^ + [AlCl(Bn-NODA)] reaction is more exothermic than the OH^−^ + [GaCl(Bn-NODA)] reaction.

### Comparison with available computational evidence

As noted in the Introduction, only one previous computational study is directly comparable with the present work. This DFT investigation employed an M06-2X functional with a cc-pVTZ basis set with PCM to simulate the aqueous environment.^38^ Inspection of the computed EAs for F and Cl listed in the SI (Section S11), obtained with different functional/basis set combinations, shows that the M06-2X/cc-pVTZ combination gives EAs for F and Cl, which are comparable with the EAs obtained in this work using the BP86-D3/6-311++G(d,p) combination (SI ection S11). In ref. 38, the relative energies of two alternative synthesis pathways for [^18^F][AlF(L)] (L = NOTA^R^) formation were investigated *i.e.* (i) fluorination of [Al(H_2_O)_6_]^2+^ followed by chelation and (ii) chelation of NOTA^R^ to Al^3+^ followed by fluorination. The work proposed plausible mechanistic routes for (i) and (ii), which involved selecting possible reaction intermediates. For each pathway these reaction intermediates were confirmed to be energy minima, but IRC calculations were not used to establish whether these minima were connected to reactants or products on the same reaction surface. Also, the fundamental steps involved in each reaction pathway were not considered (*e.g*. in (i) conversion of [Al(H_2_O)_6_]^3+^ + L^2−^ to [Al(L)]^+^ + 6H_2_O and in (ii) conversion of [AlF(H_2_O)_5_]^2+^ + L^2−^ to [AlF(L)] + 5H_2_O, these processes were each considered as one step, whereas it is likely that they each involve several steps). Therefore, the conclusion that pathway (ii) dominates over (i) should be treated with caution, although there is some evidence from NMR experiments that supports route (ii) being the dominant pathway.^39^

### Computational details

DFT calculations were carried out using the BP86 functional,^41^ augmented with the Grimme correction for dispersion (D3(BJ) version),^42^ using 6-311++G(d,p) basis sets with GAUSSIAN 16.^43^ Geometry optimizations were performed for each structure investigated and energy minima were confirmed by the absence of any imaginary frequencies. For the potential energy surfaces investigated, each located transition state (TS) was shown, using IRC calculations, to have only one imaginary frequency and to connect to minima on the surface. Reactions (1) for M = Ga and Al, in solution (water), and reaction (1) for M = Ga in the gas-phase were studied in more detail using AIM (atoms-in-molecules) theory to investigate the number, nature and strength of the formally non-bonded interactions in each stationary point structure located on the reaction surface. It was anticipated that similar non-bonded interactions occur in the stationary points located for the other reactions investigated. The outputs from the geometry optimization calculations were used in AIM calculations with GAUSSIAN16 and MULTIWFN.^50^ AIM theory^51^ is based upon a topological analysis of the electron density *ρ*(*r*) of a molecular system. It identifies a bond critical point (BCP) in a bond, which is a saddle point along the gradient path ∇^2^*ρ*(*r*) connecting two local electron *ρ*(*r*) density maxima. From the values of *ρ*(*r*) and ∇^2^*ρ*(*r*) at the BCPs, it is possible to determine the nature of a bond.^52^ In the solution calculations, the aqueous solvent was simulated with the PCM (SMD) method. It is recognised that use of an implicit solvent model like this is an approximation for a polar solvent such as water, where hydrogen-bonding will be present. Treating the solvent with an explicit method would be more appropriate, but this would be much more resource intensive and is outside the scope of the present work.

For the reaction F^−^ + [AlCl(L)] → [AlF(L)] + Cl^−^ in water, two functionals (BP86-D3 and M06-2X) with the same basis set (6-311++G(d,p) were investigated and gave very similar results (see SI Section S13).

## Conclusion

Despite the growing body of experimental work (mostly) on [AlF(L)] tracers, comparatively few theoretical studies have investigated how changes in reaction conditions, choice of metal or chelator design influence reaction mechanisms and product formation. This work represents the first systematic computational investigation of substitution chemistry in metal–chelate systems relevant to radiofluorine-based imaging agents. Using DFT calculations, we examined the reactions F^−^ + [MCl(Bn-NODA)], and OH^−^ + [MX(Bn-NODA)] (M = Ga, Al; X = Cl, F), with a PCM (water) environment to elucidate the mechanisms governing halide exchange and hydrolysis. The calculated reaction profiles indicate that each reaction is thermodynamically favourable and proceeds on one potential energy surface, as confirmed by IRC analyses. Although both the concerted and stepwise pathways almost certainly occur together, the former is predicted to play a major role under the conditions considered. A key mechanistic finding is that halide substitution is facilitated by the reversible dissociation of a metal–carboxylate bond, generating transient coordination environments that enable ligand exchange. Across all systems studied, the highest activation barrier was consistently greater for aluminium than for gallium, providing a molecular-level explanation for experimentally observed differences in reactivity.

The calculations also successfully rationalise reported experimental observations relevant to the preparation and stability of ^18^F-labelled PET imaging agents, including the greater stability of [AlF(Bn-NODA)] relative to [GaF(Bn-NODA)] under mildly alkaline conditions and the higher temperatures required for aluminium–fluoride complex formation. Taken together, the results demonstrate that both the metal centre and chelator framework play critical and interdependent roles in determining reaction pathways, kinetic barriers, and product stability.

More broadly, this study highlights the value of computational chemistry as a predictive tool for the development of next-generation metal–fluoride radiopharmaceuticals. The approach presented here provides a framework for screening metal–chelator combinations, evaluating alternative synthetic routes, and guiding experimental optimisation, thereby accelerating the design of improved PET imaging and therapeutic agents. Future studies that explicitly account for ionic strength, buffer composition, and physiologically relevant media will further refine these predictions and provide a more complete understanding of the factors governing tracer synthesis and stability under practical conditions.

## Author contributions

Project conceptualization and funding (G. R.); planning of work (J. M. D., V. K. G. and G. R.), DFT calculations (V. K. G. and R. P. K.); data analysis, manuscript preparation (J. M. D., V. K. G. and G. R.), reviewing and editing (all authors).

## Conflicts of interest

There are no conflicts to declare.

## Supplementary Material

SC-OLF-D6SC06081A-s001

## Data Availability

Further supporting information on the overall geometries, including the *xyz* coordinates and the results of the AIM calculations (PDF) is available free of charge at https://doi.org/10.5258/SOTON/D3709. Supplementary information (SI): comprises full computational details and results of the AIM calculations for all of the reactions described in this study. See DOI: https://doi.org/10.1039/d6sc06081a.
