# Supplementary material for: Mechanistic insights for the preparation of improved metal–fluoride based radioimaging agents
Source: Chem Sci. 2026 Sep 22. Online ahead of print. doi: 10.1039/d6sc06081a (PMC13635819; doi:10.1039/d6sc06081a)
Supplement: Supplementary file 1 [file SC-OLF-D6SC06081A-s001.pdf]

Supplementary Information (SI) for Chemical Science.
This journal is © The Royal Society of Chemistry 2026

Mechanistic insights for preparation of improved metal-fluoride based radioimaging agents

Victoria K. Greenacre, Rhys P. King, John M. Dyke* and Gillian Reid*

School of Chemistry and Chemical Engineering, University of Southampton, Southampton SO17 1BJ, UK; email: jmdyke@soton.ac.uk, g.reid@soton.ac.uk

Table of Contents

1. Supplementary data for [GaCl(L)] + F^- → [GaF(L)] +Cl^- reaction (L = Bn=NODA) 4
1a) Comparison to crystal structure [GaCl(Bn-NODA)]·$2H_2O$] 4
1b) Gas-phase calculations: Geometry Tables 4
1c) Gas-phase calculations: Image of each stationary point. 5
1d) Gas-phase calculations: Charge Density Table (Mulliken Analysis) 8
1e) Gas-phase calculations: Potential Energy Diagram...8
1f) Gas-phase calculations: Summary of structural changes 8
1g) Solution-phase calculations: Geometry Tables...9
1h) Solution-phase calculations: Image of each stationary point 9
1i) Solution-phase calculations: Charge Density Table (Mulliken Analysis).........................12
2. Supplementary data for [AlCl(L)] + F^- → [AlF(L)] +Cl^- reaction 13
2a) Comparison to crystal structure [AlF(Bn-NODA)]·$2H_2O$] 13
2b) Solution-phase calculations: Geometry Tables..14
2c) Solution-phase calculations: Image of each stationary point 14
2d) Solution-phase calculations: Charge Density Table (Mulliken Analysis) 17
3. Supplementary data for [GaCl(L)] + OH^- → [Ga(OH)(L)] +Cl^- reaction 18
3a) Solution-phase calculations: Geometry Tables..18
3b) Solution-phase calculations: Image of each stationary point 18
3c) Solution-phase calculations: Charge Density Table (Mulliken Analysis).......................21
4. Supplementary data for [AlCl(L)] + OH^- → [Al(OH)(L)] + Cl^- reaction...........................22
4a) Solution-phase calculations: Geometry Tables..22
4b) Solution-phase calculations: Image of each stationary point 22
4c) Solution-phase calculations: Charge Density Table (Mulliken Analysis).......................25
5. Supplementary data for [GaF(L)] + OH^- → [Ga(OH)(L)] + F^- reaction 26
5a) Solution-phase calculations: Geometry Tables..26
5b) Solution-phase calculations: Image of each stationary point 26
5c) Solution-phase calculations: Charge Density Table (Mulliken Analysis).......................29
6. Supplementary data for [AlF(L)] + OH^- → [Al(OH)(L)] + F^- reaction 30
6a) Solution-phase calculations: Geometry Tables..30

6b) Solution-phase calculations: Image of each stationary point 30
6c) Solution-phase calculations: Charge Density Table (Mulliken Analysis).......................33
7. Comparison of the geometries between [MX(L)] systems 34
7a) RC geometries 34
7b) TS1 geometries..35
7c) IC1 geometries 36
7d) ICts geometries..37
7e) IC2 geometries...38
7f) TS2 geometries...39
7g) PC geometries 40
8. Summary for M-X Bond Lengths and M, X Charge Densities 41
8a) Reaction F^{-} + [MCl(L)], L=Bn-NODA 41
8b) Reaction OH^{-} + [MCl(L)], L=Bn-NODA 42
8c) Reaction OH^{-} + [MF(L)] for M=Ga and Al, L=Bn-NODA 42
9. Atoms in Molecules – Summary for [MCl(L)] + F^{-} → [MF(L)] + Cl^{-}.................................44
9a) Gas-phase Calculations – Gallium Reaction Complex (**RC**)...44
9b) Gas-phase Calculations – Gallium Transition State 1 (**TS1**) 46
9c) Gas-phase Calculations – Gallium Ionic Complex 1 (**IC1**) 47
9d) Gas-phase Calculations – Gallium Ionic Complex transition state (**ICts**) 49
9e) Gas-phase Calculations – Gallium Ionic Complex 2 (**IC2**)..50
9f) Gas-phase Calculations – Gallium Transition State 2 (**TS2**) 52
9g) Gas-phase Calculations – Gallium Product Complex (**PC**)...53
9h) Solution-phase Calculations – Gallium Reaction Complex (**RC**) 55
9i) Solution-phase Calculations – Gallium Transition State 1(**TS1**) 57
9j) Solution-phase Calculations – Gallium Ionic Complex 1 (**IC1**)......................................58
9k) Solution-phase Calculations – Gallium Ionic Complex transition state (**ICts**) 60
9l) Solution-phase Calculations – Gallium Ionic Complex 2 (**IC2**).......................................62
9m) Solution-phase Calculations – Gallium Transition State 2 (**TS2**) 63
9n) Solution-phase Calculations – Gallium Product Complex (**PC**) 65
9o) Solution-phase Calculations – Aluminium Reaction Complex (**RC**).............................66
9p) Solution-phase Calculations – Aluminium Transition State 1 (**TS1**) 68
9q) Solution-phase Calculations – Aluminium Ionic Complex 1 (**IC1**)................................69
9r) Solution-phase Calculations – Aluminium Ionic Complex transition state (**ICts**)...........71
9s) Solution-phase Calculations – Aluminium Ionic Complex 2 (**IC2**) 73
9t) Solution-phase Calculations – Aluminium Transition State 2 (**TS2**) 74
9u) Solution-phase Calculations – Aluminium Product Complex (**PC**)...............................76
9v) Comparison of RC structures from AIM data 78
9w) Comparison of IC1 structures from AIM data 78

10. Geometric data and energies for Ga(L), $Ga(L)^+$, Al(L), $Al(L)^+$ 79
10a) Gas phase $Ga(L)^+$ & $Ga(L)^0$ geometries 79
10b) Solution phase $Ga(L)^+$ & $Ga(L)^0$ geometries 79
10c) Solution phase $Al(L)^+$ & $Al(L)^0$ geometries 80
11. Electron Affinity calculations and tables for F, Cl, OH 81
11a) Benchmarking for Cl / F electron affinity 81
11b) Benchmarking for OH electron affinity 82
11c) Justification for the choice of basis set 83
12. Extra information on the DFT calculations 84
13. References 84

1. Supplementary data for [GaCl(L)] + F^- → [GaF(L)] + Cl^- reaction (L = Bn=NODA)

1a) Comparison to crystal structure [GaCl(Bn-NODA)]·$2H_2O$]

(a)

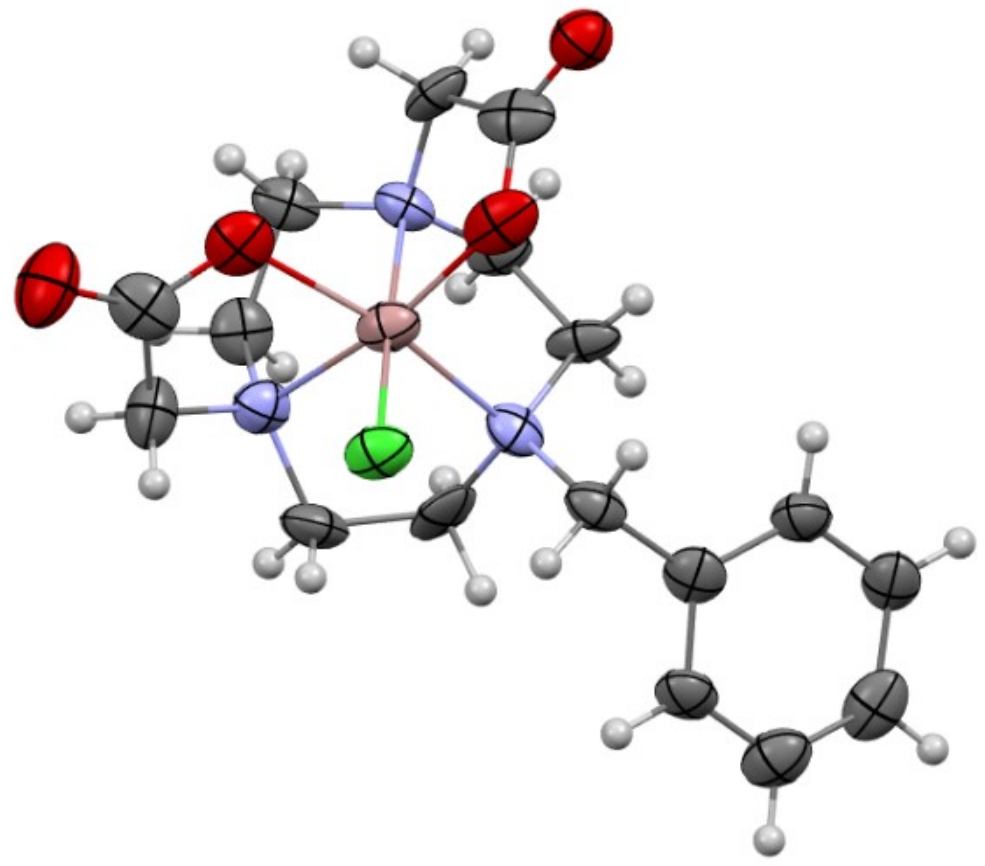

(b)

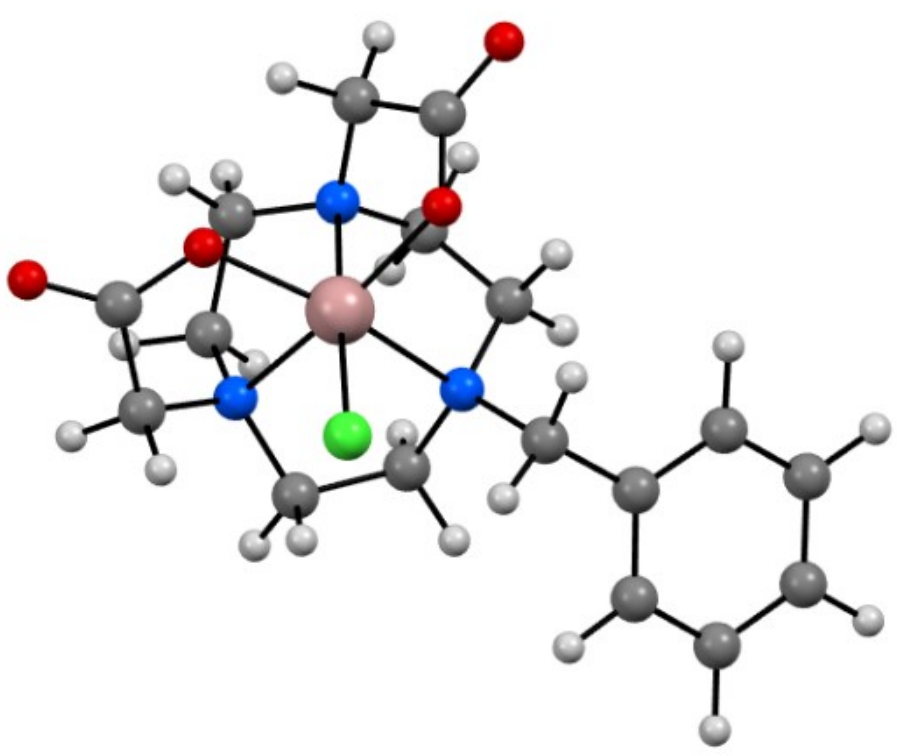

(c)

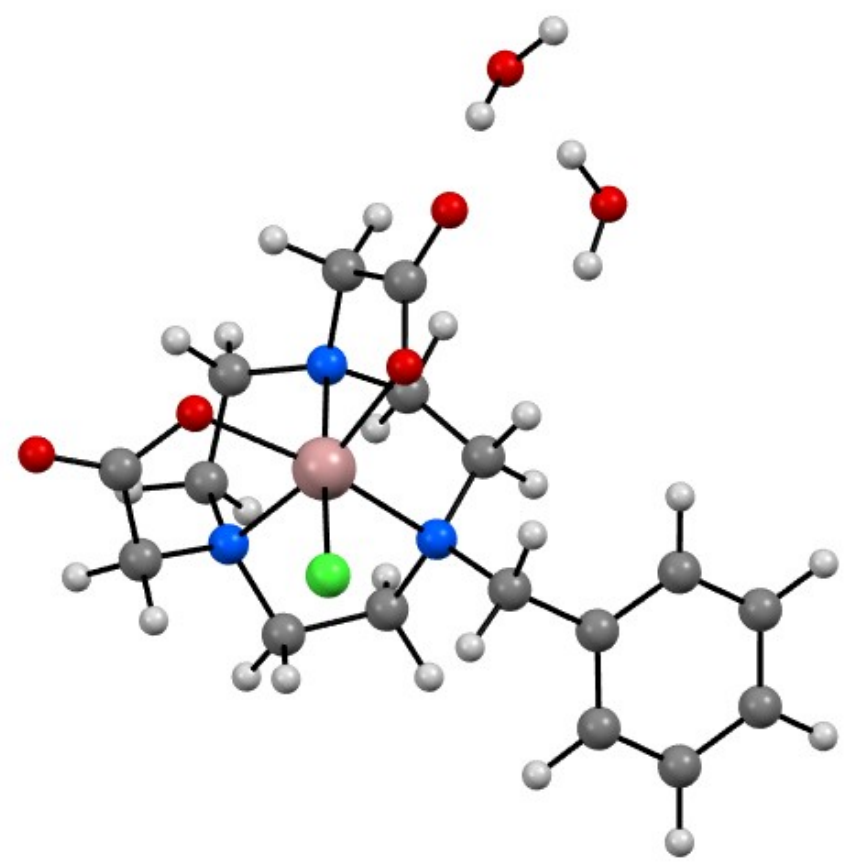

Geometries of [GaCl(Bn-NODA)], (a) crystal structure (lattice water molecules are omitted) from ref. 27, (b) optimized geometry of the [GaCl(Bn-NODA)] molecule in the gas phase, and (c) optimized geometry of [GaCl(Bn-NODA)] molecule with two H_2O molecules added; all calculations were performed at the BP86-D3/6-311++G(d,p) level.

1b) Gas-phase calculations: Geometry Tables

Note: Cl/OH-Ga-$N_{(trans)}$ values are taken as the closest to 180º, Ga-$N_{(trans)}$ is the N atom trans to Cl. Ga-O taken as the shortest Ga-O distance from each carboxylate arm.

In the gas phase – RC has a different geometry, relating to the position of the incoming Cl^-

	[GaCl(L)]	RC	TS1	IC1	ICts	IC2	TS2	PC	[GaF(L)]
Ga-Cl	2.26219	2.32811	2.33452	2.24648	2.39892	2.48950	2.84699	5.84605	--X--
Ga-F	--X--	4.30966	2.78362	1.84525	1.84198	1.84469	1.83033	1.84169	1.84614
Ga-O	1.93157<br>1.96634	1.95060<br>1.90535	1.91147<br>1.94111	1.94720<br>2.01045	1.97942<br>1.92438	2.01465<br>1.91713	2.03425<br>1.91631	1.95219<br>1.98990	1.92813<br>1.95716
Ga-N	2.25347<br>2.19716	2.35125<br>2.19386	2.86439<br>2.21999	3.9986<br>4.14582	2.95573<br>2.48190	2.23750<br>2.93644	2.68440<br>2.19794	2.16075<br>2.17875	2.22888<br>2.18650

Ga-N$_{(trans)}$	2.23584	2.19395	2.35130	2.25930	2.95745	2.93406	2.67428	2.23484	2.22149
N-Ga-O	159.48185<br>158.53937	162.36664<br>160.35574	150.43235<br>151.07393	128.43160<br>93.56934	120.29717<br>128.78539	134.92342<br>121.31353	143.67931<br>131.36333	161.08631<br>160.73332	161.12444<br>160.52607
Cl-Ga-F	--X--	147.15417	146.64954	124.43818	84.42728	80.60220	72.26613	68.29630	--X--
Cl-Ga-N$_{(trans)}$	172.71387	166.88584	145.53075	145.56296	132.09936	133.84785	137.57922	103.54638	--X--
F-Ga-N$_{(trans)}$	--X--	123.52699	134.51297	134.98845	155.47320	155.66440	147.94178	171.84102	166.77455
**Energy**	**-3512.739524**	**-3612.679995**	**-3612.660519**	**-3612.695065**	**-3612.684596**	**-3612.687116**	**-3612.686116**	**-3612.728322**	**-3152.346149**
**Energy G**	**-3512.404255**	**-3612.349979**	**-3612.330478**	**-3612.366679**	**-3612.354374**	**-3612.356731**	**-3612.353864**	**-3612.397258**	**-3152.009611**

[GaCl(L)] + F^- = -3512.739524 + -99.888239 au = **E = -3612.627763** & G = -3612.306652 au

[GaF(L)] + Cl^- = -3152.346149 + -460.330815 au = **E = -3612.676969** & G = -3612.355449 au.

1c) Gas-phase calculations: Image of each stationary point.

In the gas phase – RC has a different geometry, relating to the position of the incoming Cl^-

[GaCl(L)]	RC
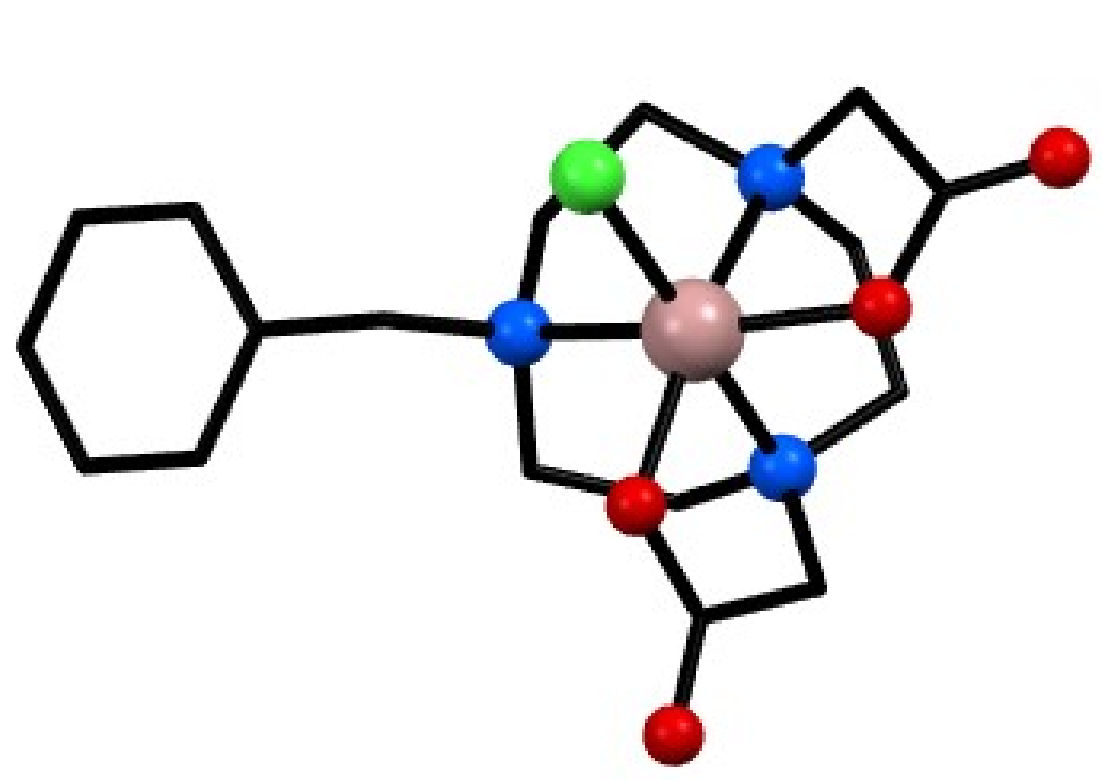	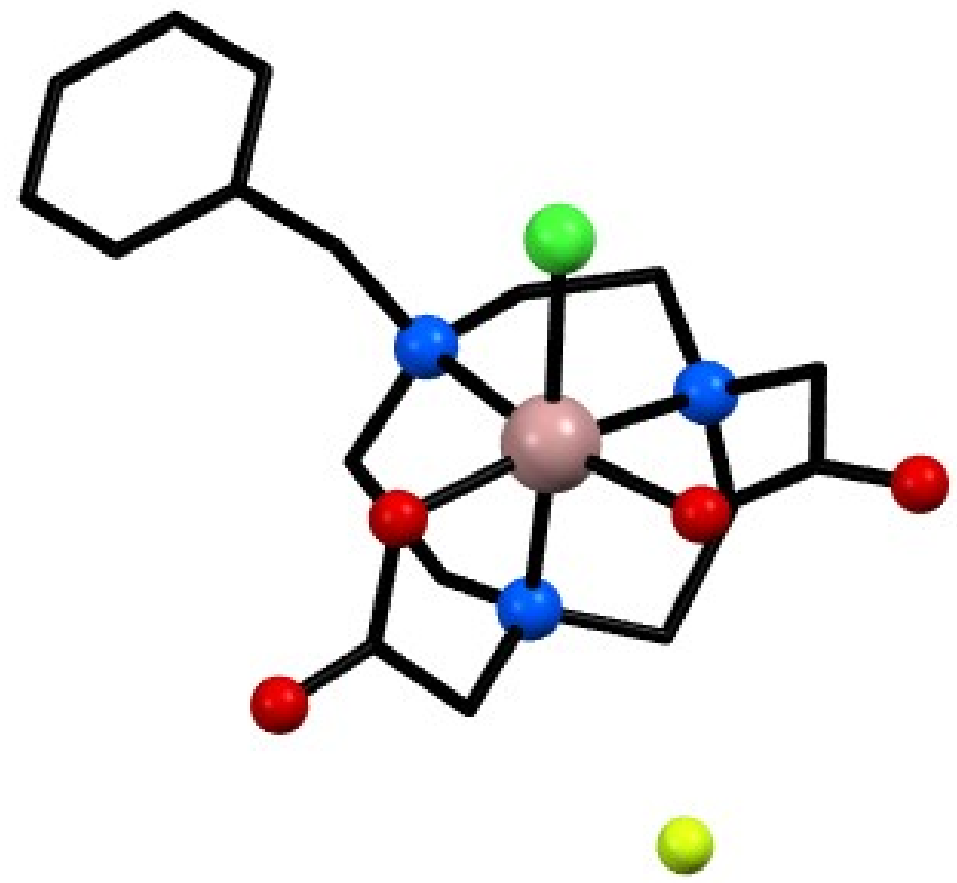
TS1	IC1
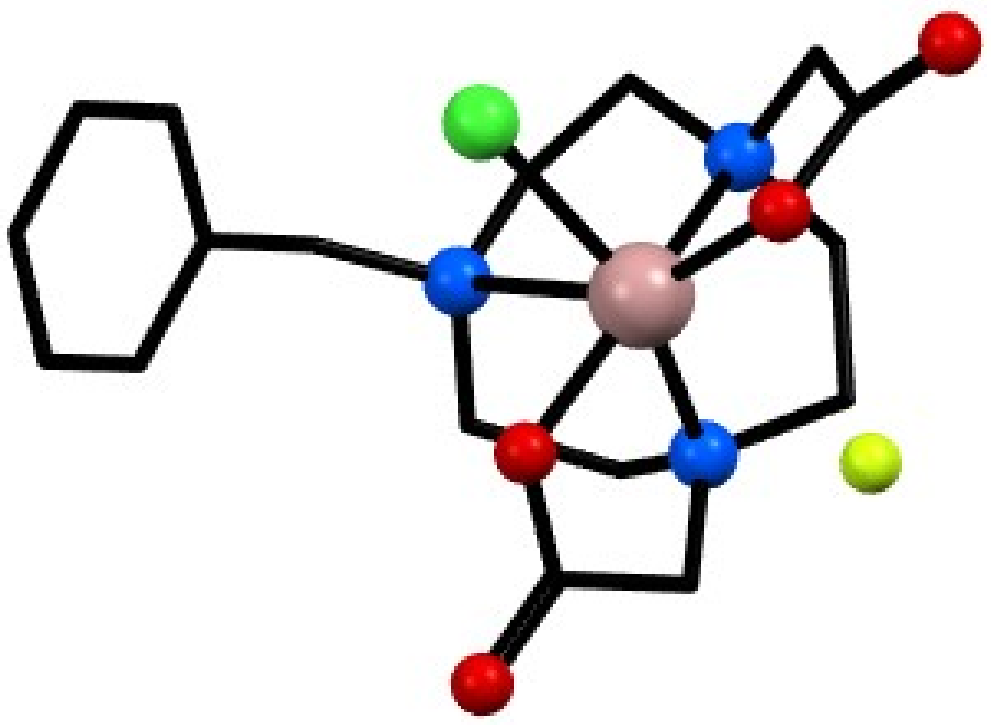	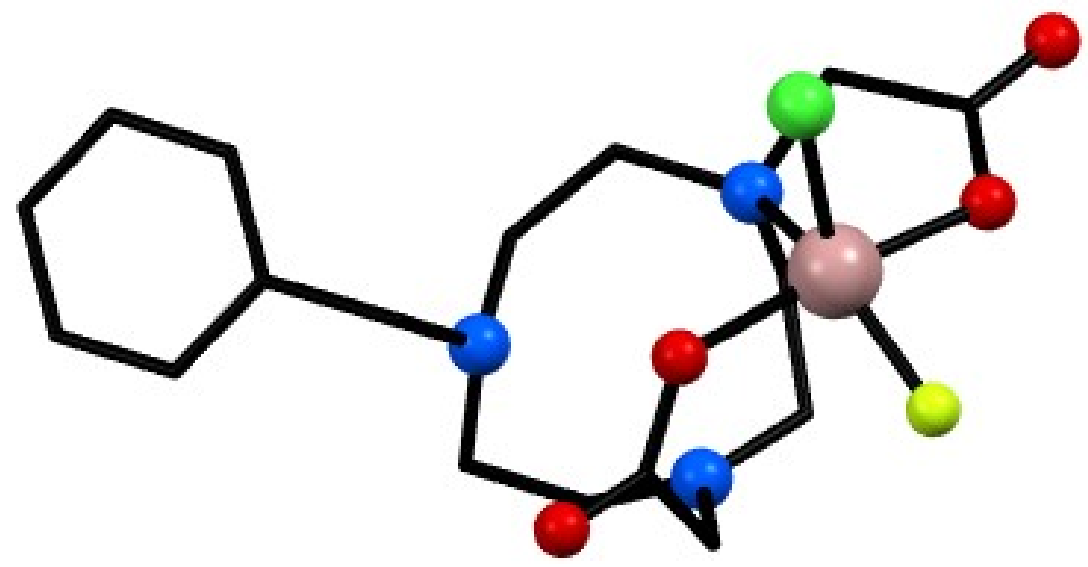
ICts	IC2

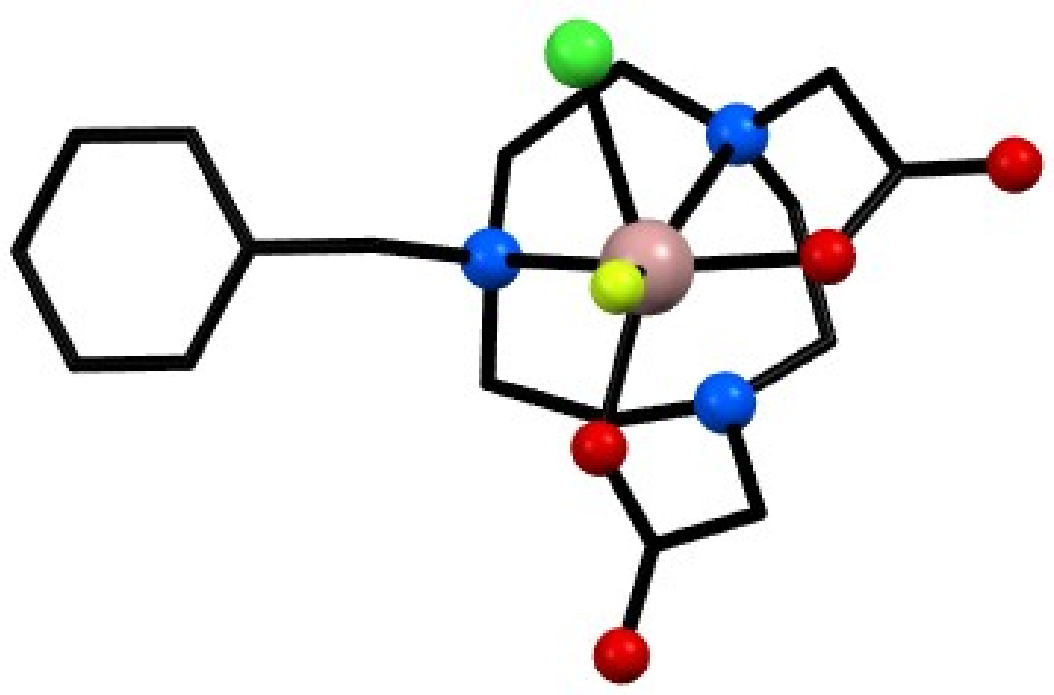	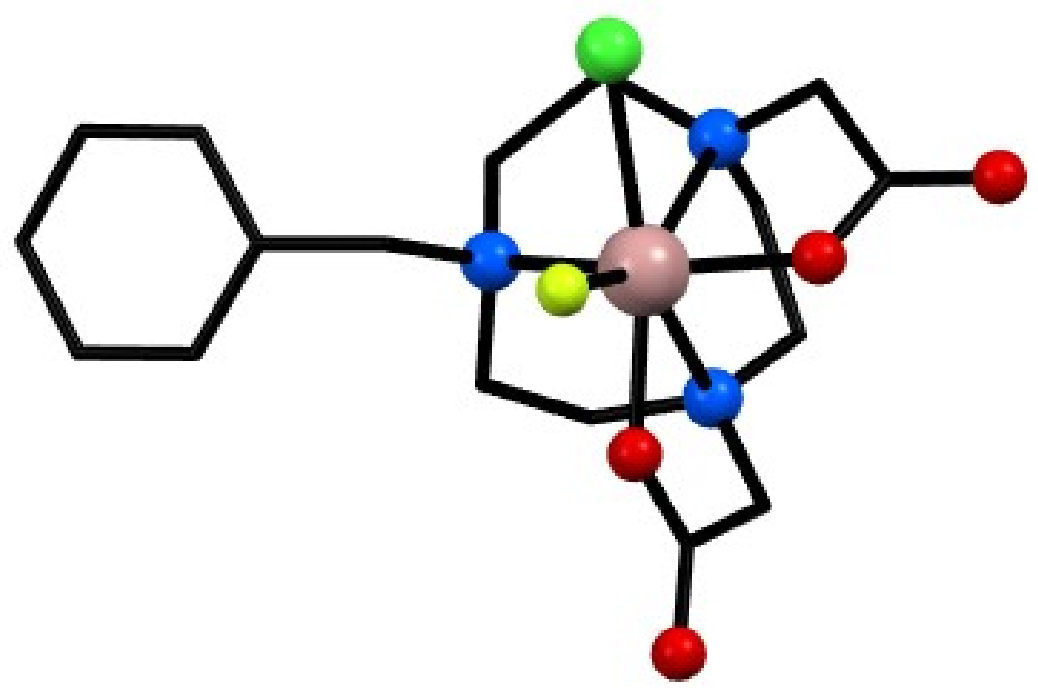
TS2	PC
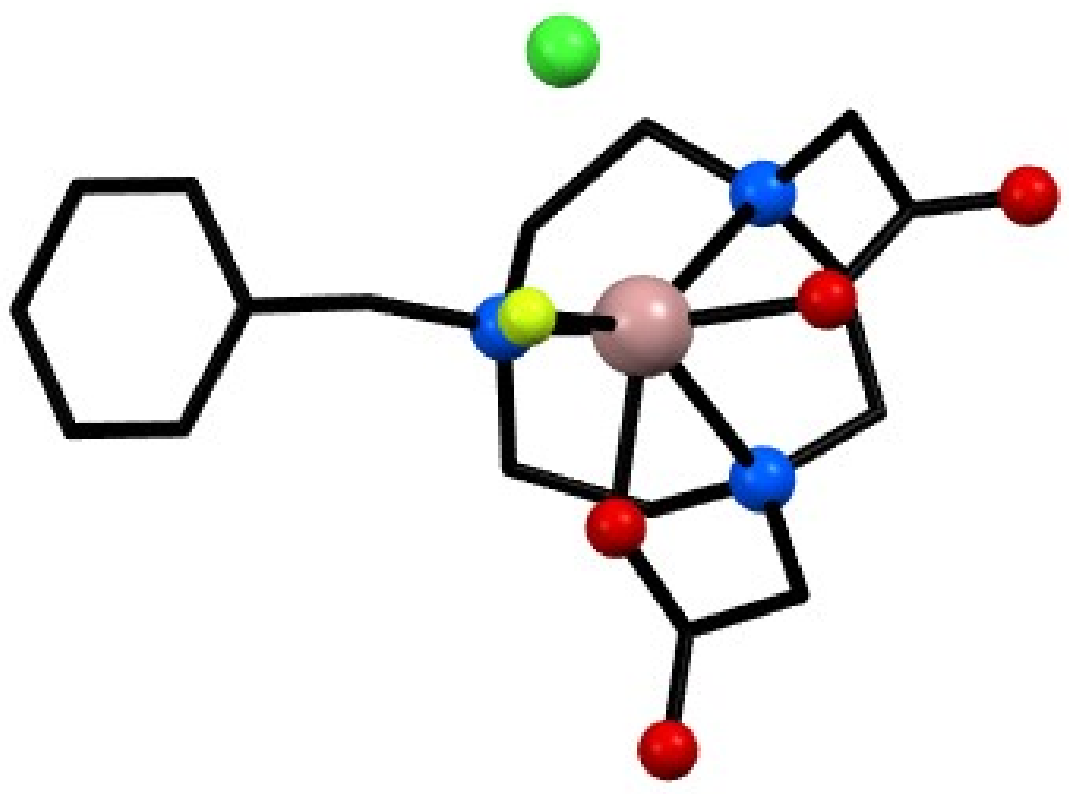	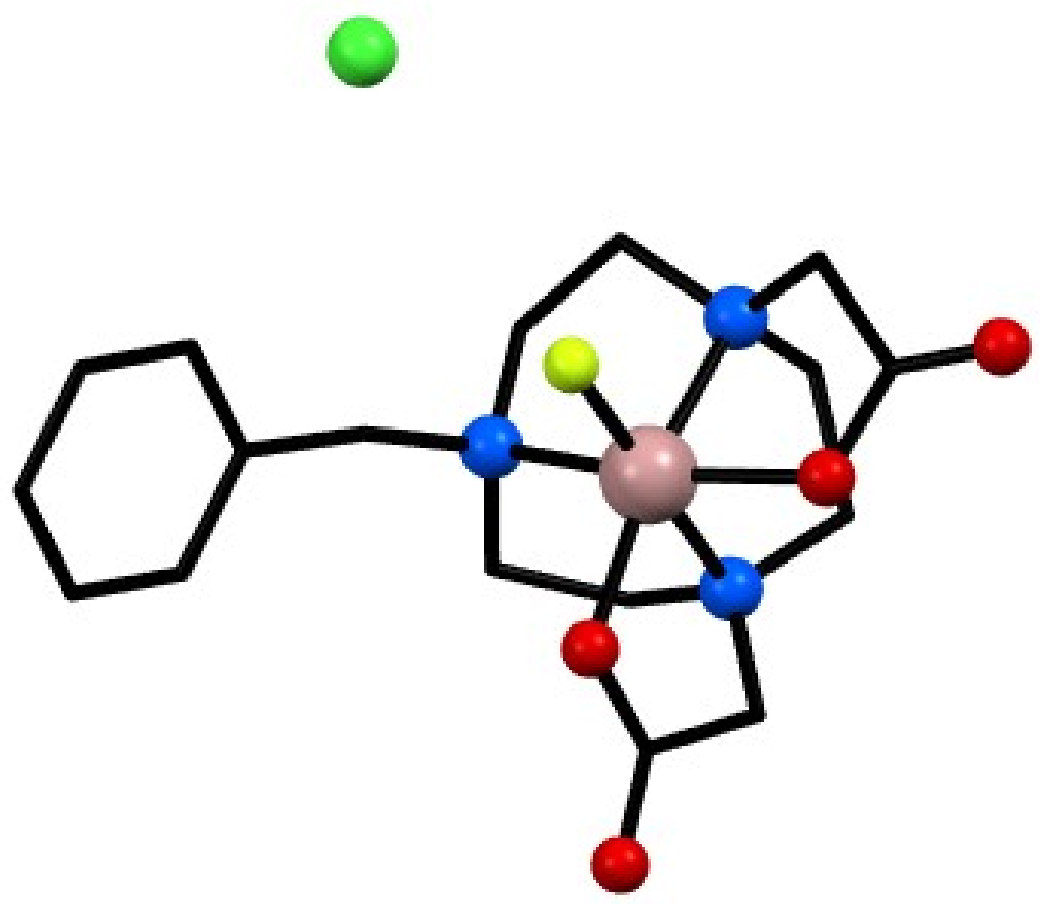
[GaF(L)]	
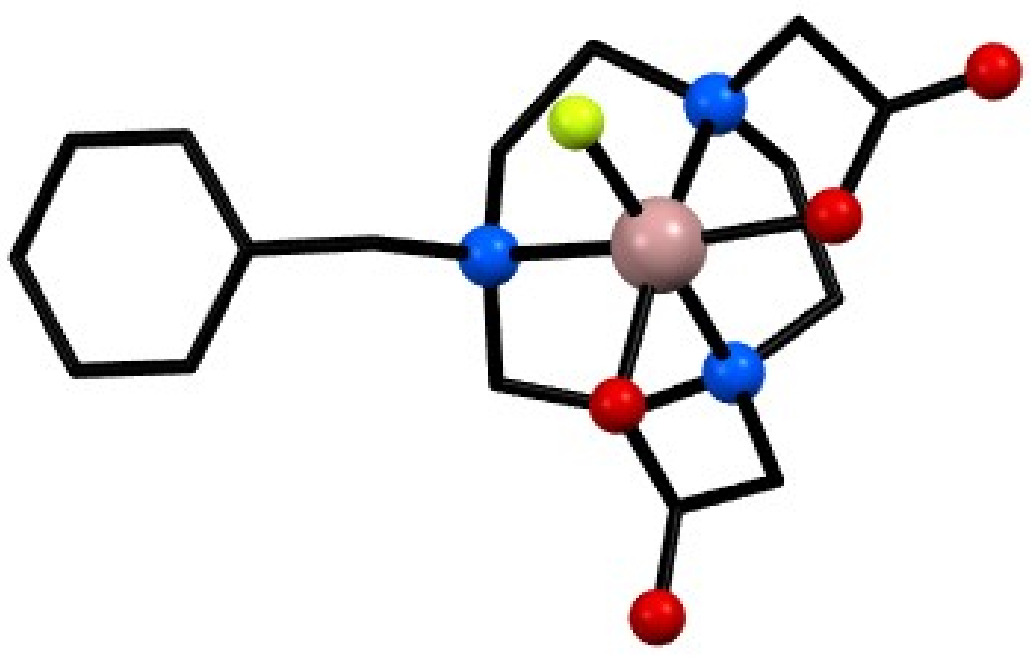	

**[GaCl(L)]**	**RC**
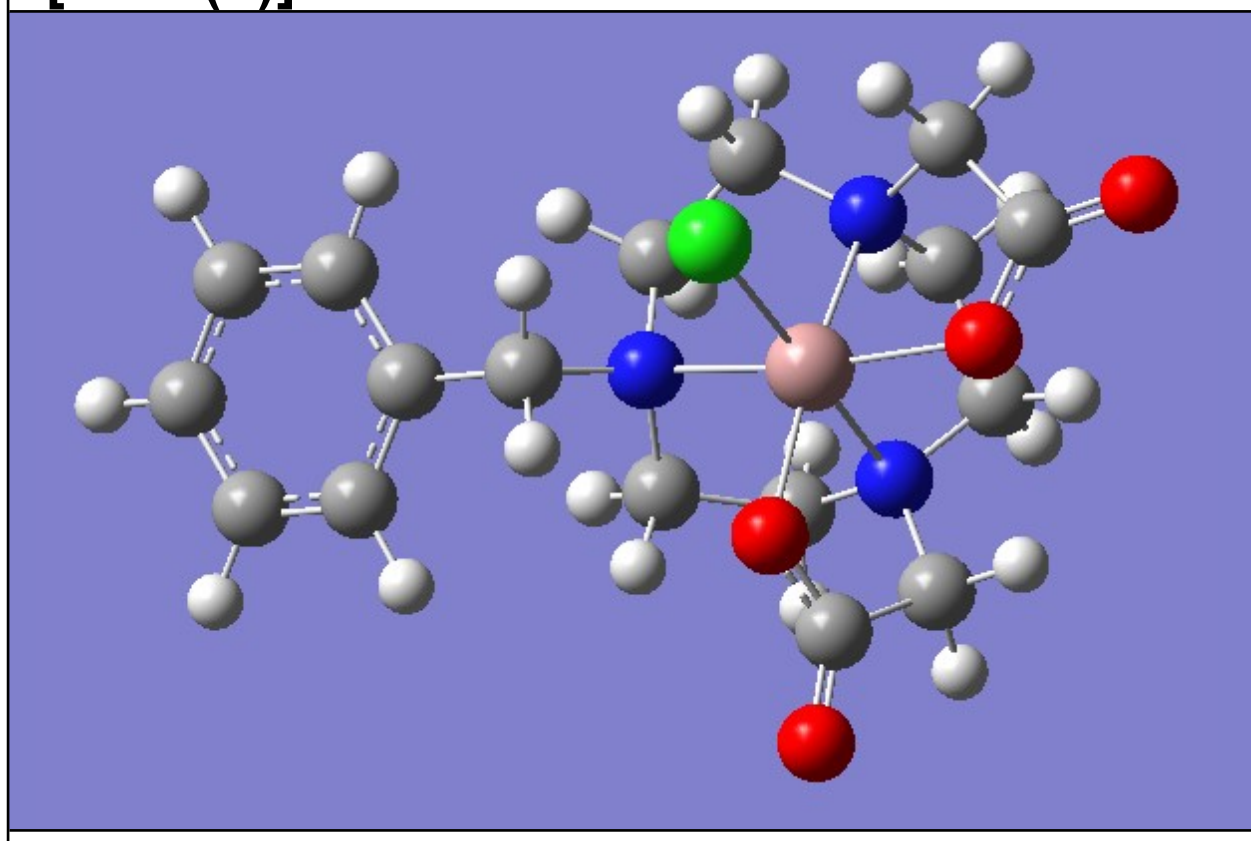	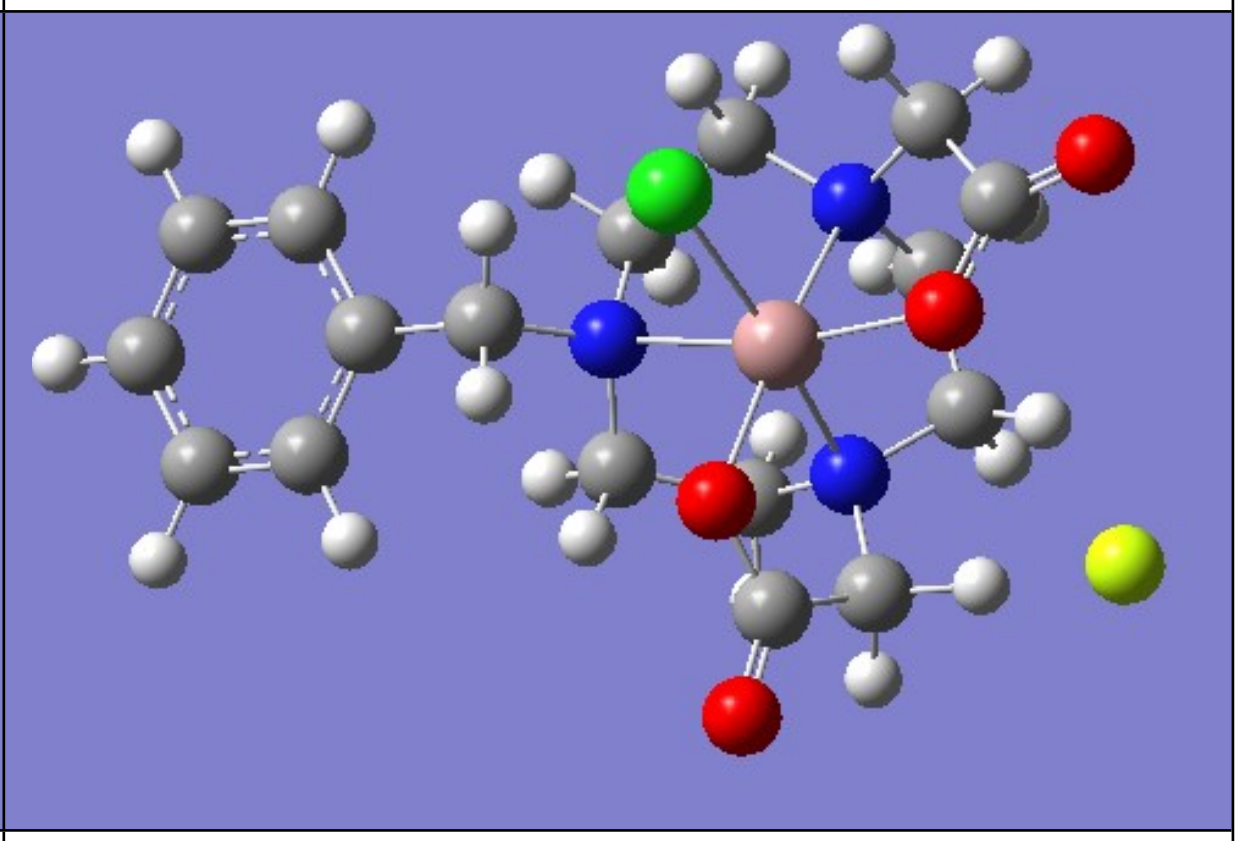
**TS1**	**IC1**

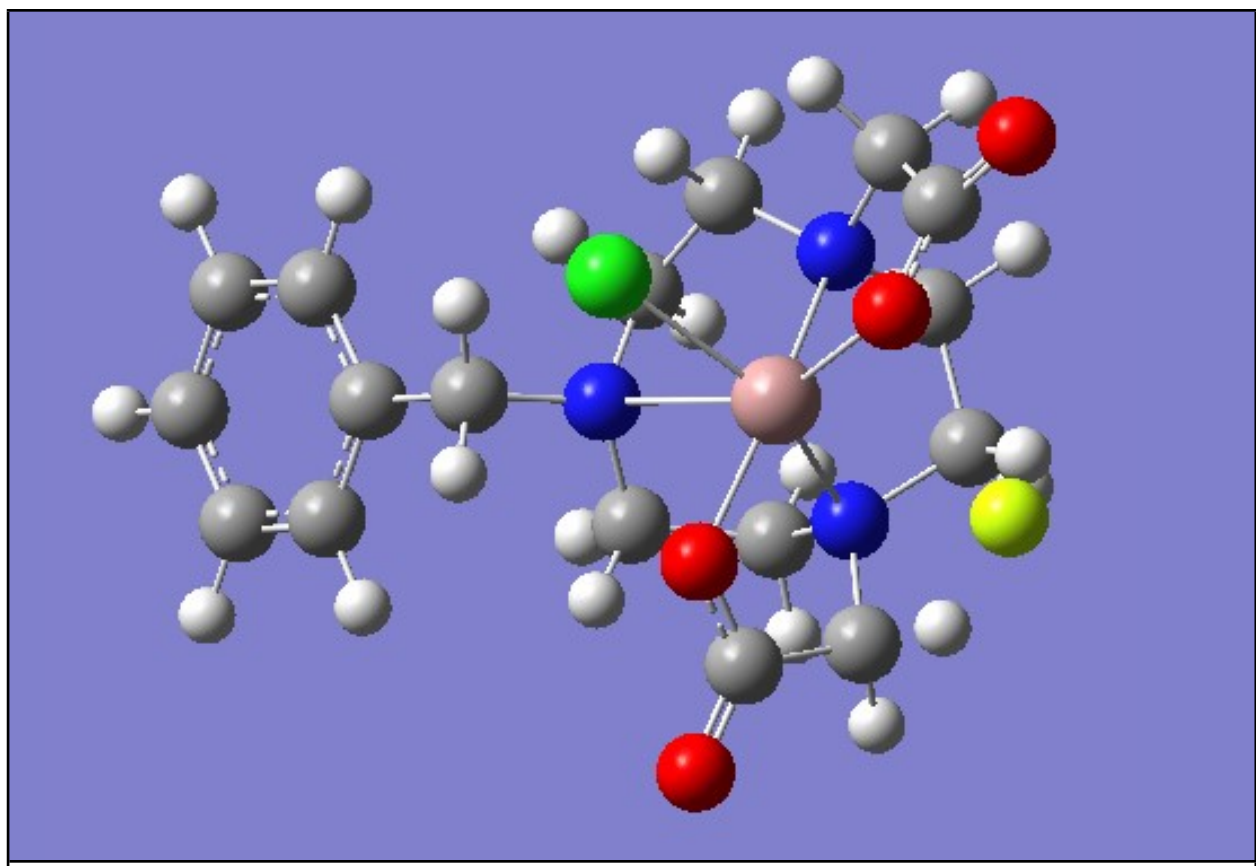	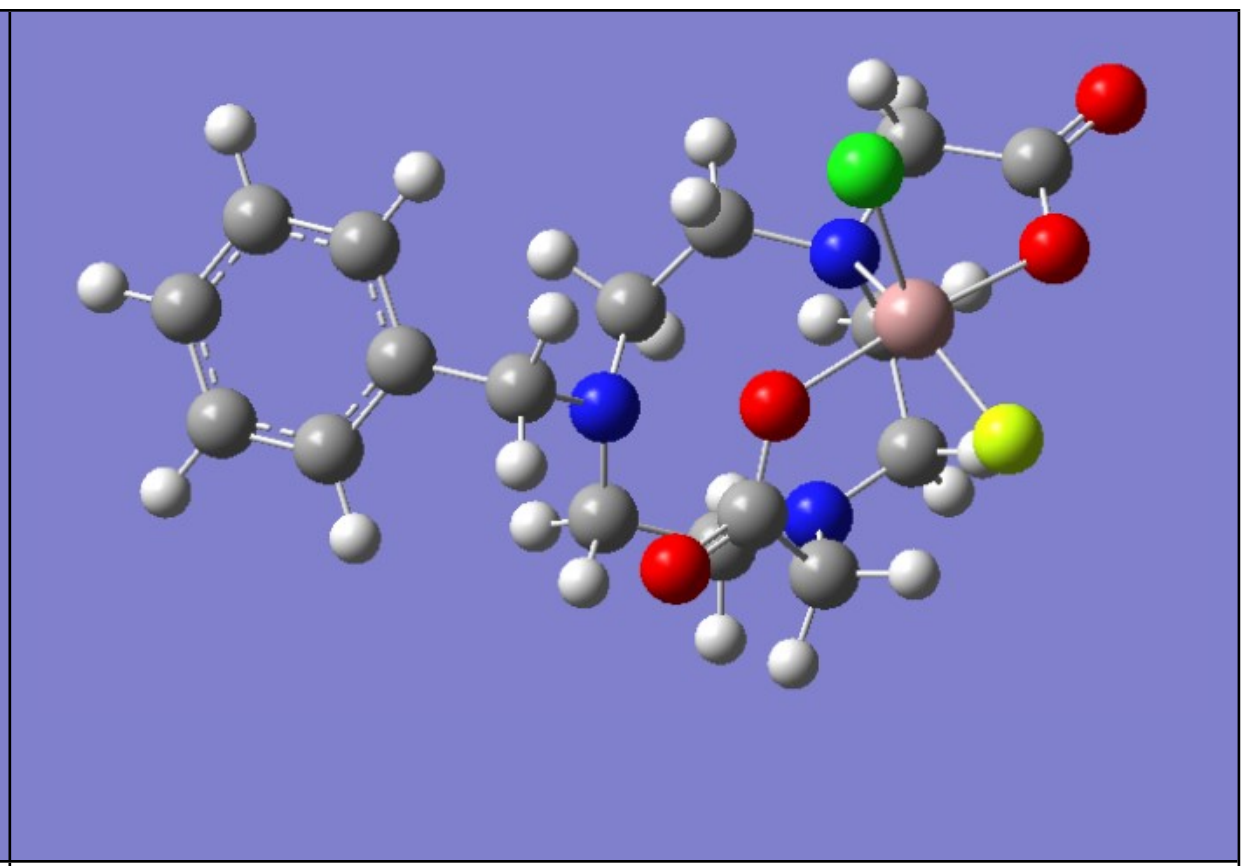
**ICts**	**IC2**
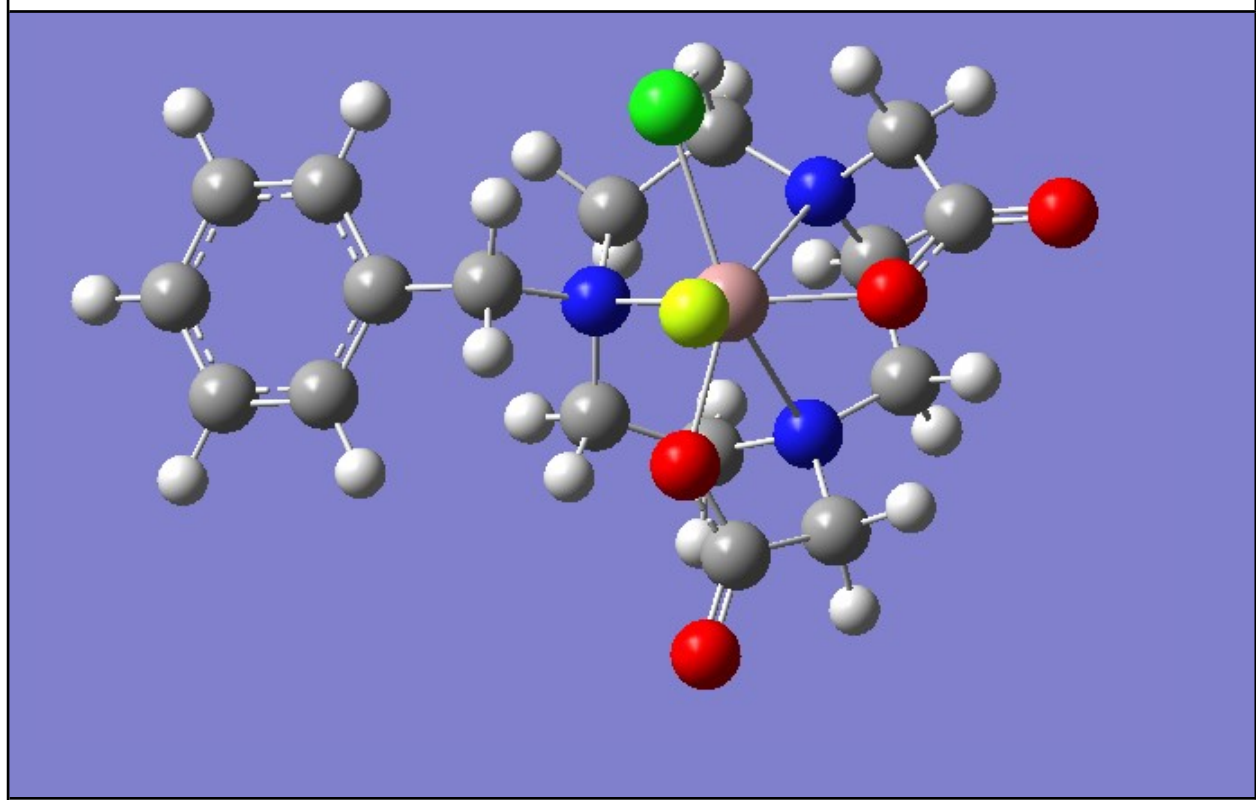	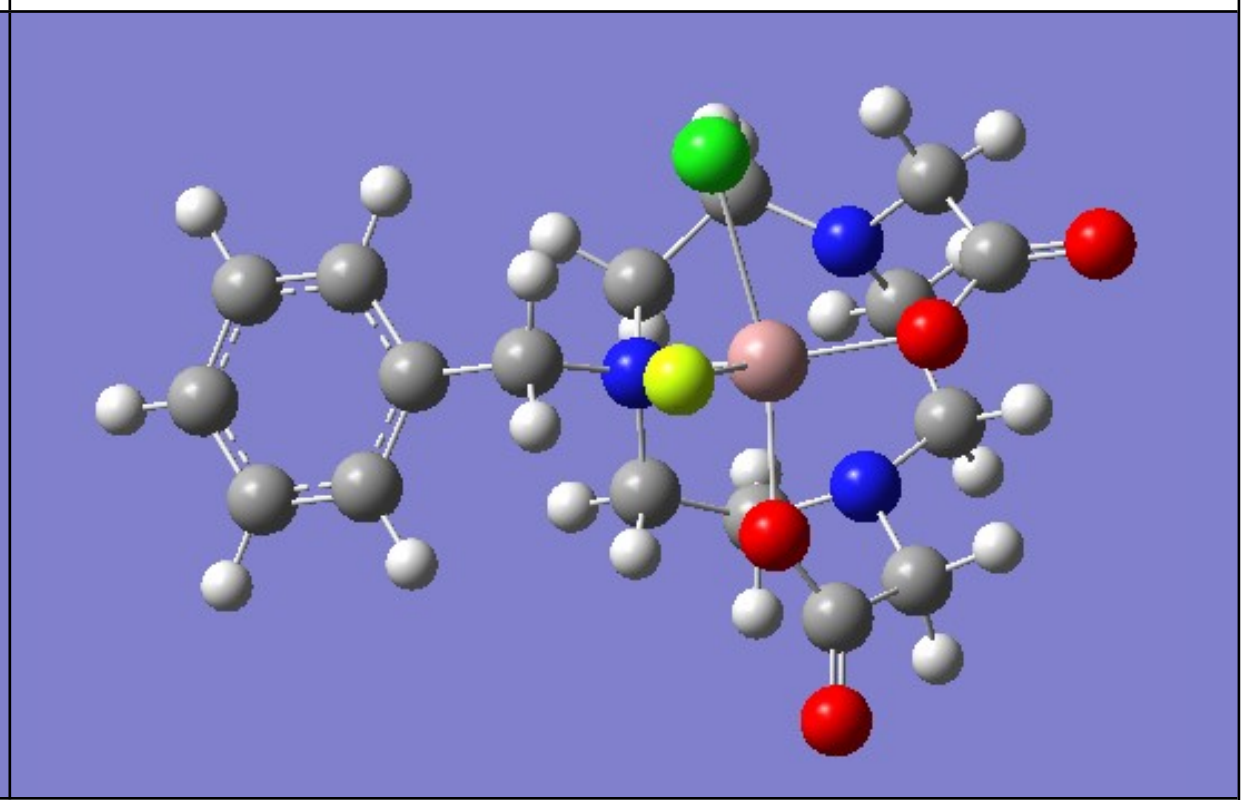
**TS2**	**PC**
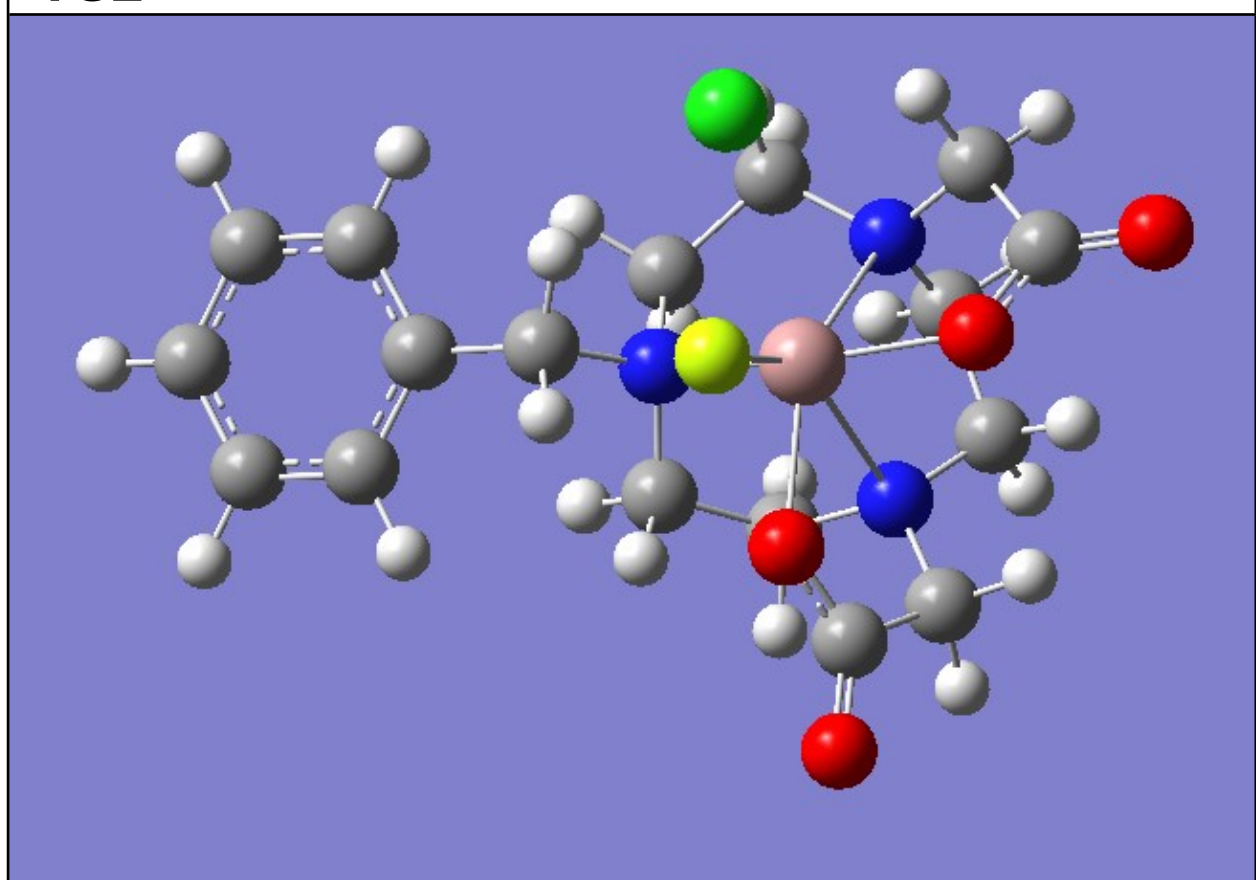	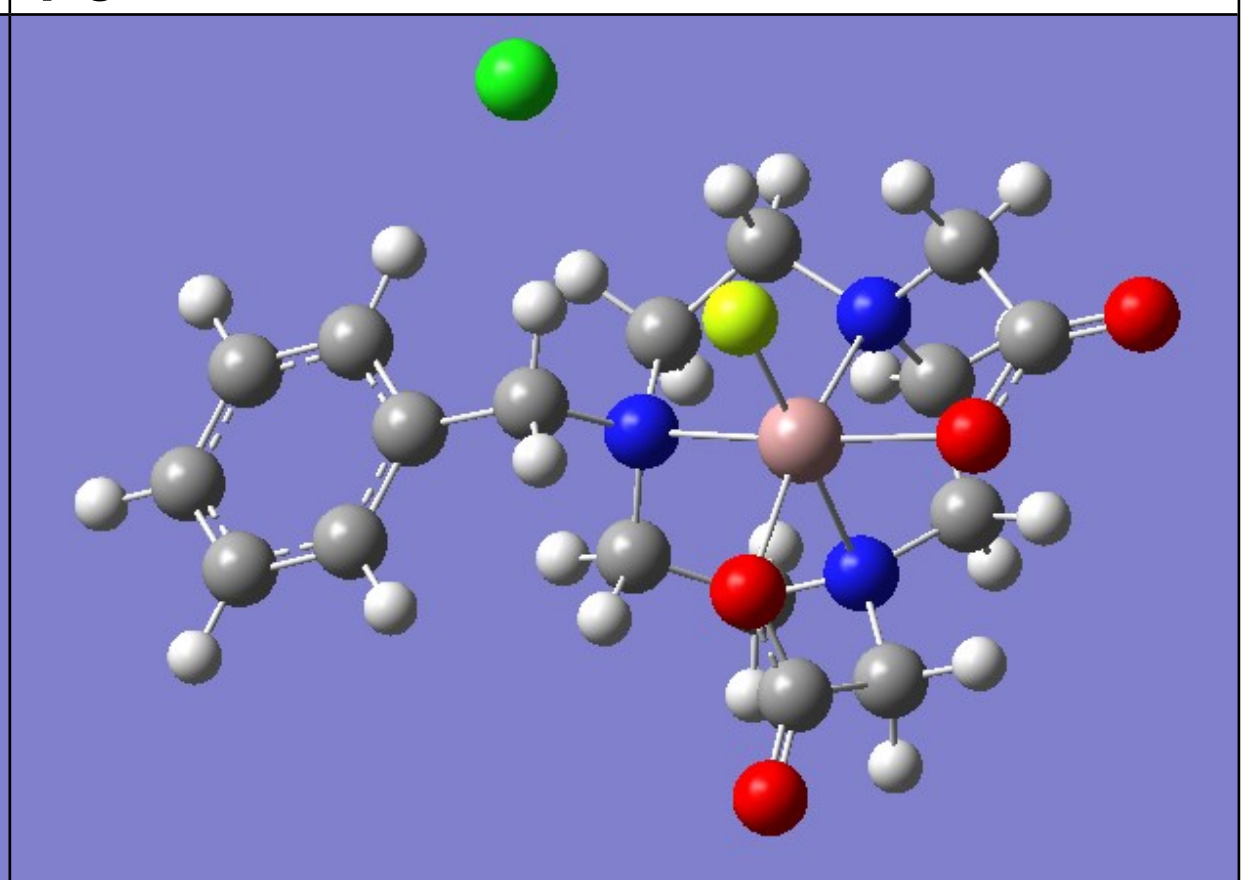
**[GaF(L)]**	
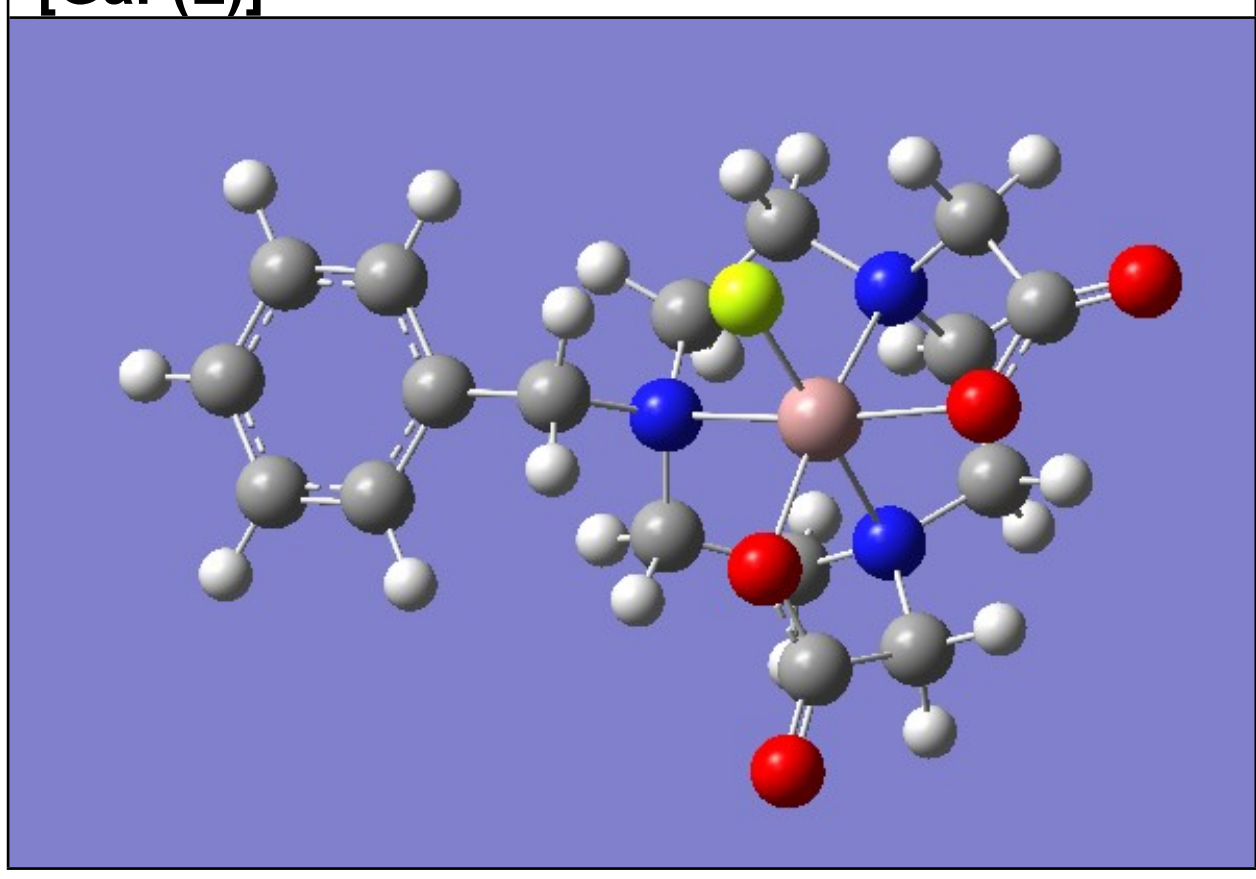	

1d) Gas-phase calculations: Charge Density Table (Mulliken Analysis)

Mulliken charges of [GaCl(L)] + F^- geometries around Ga centre. In gas-phase/6-311++G(d,p)

	[GaCl(L)]	RC	TS1	IC1	ICts	IC2	TS2	PC	[GaF(L)]
**Ga**	1.35232	1.53254	1.27585	0.85594	1.52412	1.53896	1.82163	1.81852	1.79169
**Cl**	-0.41269	-0.48897	-0.42370	-0.25690	-0.27681	-0.31696	-0.43521	-0.66572	--X--
**F**	--X--	-0.62359	-0.55385	-0.49762	-0.48545	-0.41598	-0.40572	-0.54971	-0.55191
**$O_{(3)}$**	-0.40781	-0.35476	-0.23113	-0.29499	-0.47194	-0.39260	-0.44344	-0.53069	-0.52453
**$O_{(8)}$**	-0.38463	-0.44202	-0.33808	-0.15149	-0.40210	-0.43720	-0.47121	-0.45079	-0.46434
**$N_{(5)}$**	-0.1052	-0.23007	+0.07836	+0.18817	+0.17082	+0.11976	+0.07742	-0.11751	-0.09217
**$N_{(6)}$**	-0.40582	-0.39928	-0.41219	+0.12980	-0.07696	-0.11769	-0.33864	-0.20854	-0.18539
**$N_{(15)}$**	-0.21494	-0.16257	+0.15619	+0.55162	+0.18514	+0.04920	-0.07379	-0.12096	-0.10808

Atom numbers for N atoms and carboxylate atoms around Ga vary slightly between geometries, reported values are the equivalent positions, with the carboxylate oxygens being the closest to the metal centre.

1e) Gas-phase calculations: Potential Energy Diagram

Schematic potential energy diagrams for the reaction F– +Ga(L)Cl in the gas phase. Values were computed at the BP86-D3/6-311++G(d,p) level; (ΔE, ΔGϕf,298K) values are shown on each stationary point in kcal.mol-1

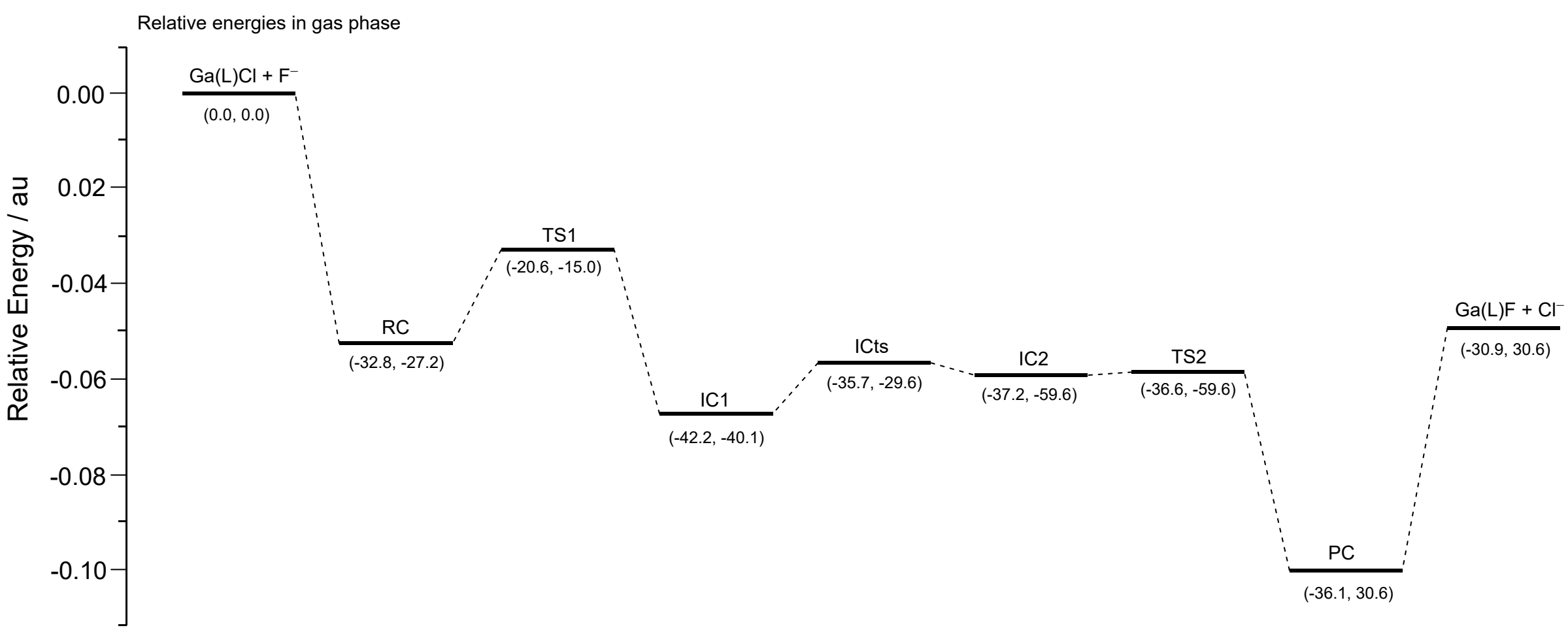

1f) Gas-phase calculations: Summary of structural changes

Computed structural changes of the stationary points in the F^- + [GaCl(Bn-NODA)] reaction in the gas-phase:

For the gas-phase case the Ga-N_{trans} bond increases as the reaction proceeds from 2.24 Å in [GaCl(Bn-NODA)] to 2.96 Å in the TS **ICts**. It then reduces to 2.22 Å in [GaF(Bn-NODA)]. The other two Ga-N bonds show a similar change with maximum values at **IC1**, with the two Ga-O bond lengths being approximately constant throughout the reaction. The ionic complex **IC1** Ga can be viewed as 5-coordinate with Ga-F, Ga-Cl, Ga-N_{trans} and two Ga-O bonds as the two other Ga-N distances are all greater than the sum of the van der Waals radii of Ga and N of 3.42 Å (26)) (at 3.99 Å, and 4.14 Å; Ga-N_{trans} is 2.26 Å). The O atoms bonded to Ga show an increase in negative charge from [GaCl (Bn-NODA)] to [GaF(Bn-NODA)], but a decrease from

[GaCl(Bn-NODA)] to the ionic complex **IC1** and then an increase to [GaF(Bn-NODA)]. Similarly, the N atoms show a decrease of negative charge from [GaCl(Bn-NODA)] to the ionic complex **IC1** (where all nitrogen atoms are positively charged). The negative charge then increases from **IC1** to **ICts**, **IC2**, **TS2**, **PC** and [GaF(Bn-NODA)]. The overall negative charge on the N atoms is lower in [GaF(Bn-NODA)] than [GaCl(Bn-NODA)].

1g) Solution-phase calculations: Geometry Tables

Note: Cl/OH-Ga-N$_{(trans)}$ values are taken as the closest to 180º, Ga-N$_{(trans)}$ is the N atom trans to Cl. Ga-O taken as the shortest Ga-O distance from each carboxylate arm.

	**[GaCl(L)]**	**RC**	**TS1**	**IC1**	**ICts**	**IC2**	**TS2**	**PC**	**[GaF(L)]**
Ga-Cl	2.30639	*2.30980*	*2.27269*	*2.30223*	*2.35305*	*2.38388*	*3.75717*	*5.16079*	--X--
Ga-F	--X--	4.81545	2.82606	*1.91963*	*1.91885*	*1.86848*	1.82958	1.87254	1.87615
Ga-O	1.98211<br>2.03987	1.98120<br>2.04872	1.99758<br>2.38631	2.03950<br>4.33521	2.37484<br>2.22684	4.07830<br>2.03129	3.18941<br>1.99284	1.98732<br>2.03665	1.98074<br>2.02988
Ga-N	2.16068<br>2.12532	2.12674<br>2.15510	2.22833<br>2.17249	2.16657<br>2.20394	2.37976<br>2.27564	2.15408<br>2.16033	2.05401<br>2.12511	2.11710<br>2.14493	2.14594<br>2.12196
Ga-N$_{(trans)}$	2.15449	2.15070	2.14159	2.14124	2.15691	2.17354	2.10984	2.14970	2.14836
N-Ga-O	163.69865<br>163.97174	163.58453<br>164.15550	139.71984<br>151.99060	160.40935<br>134.69244	147.66321<br>141.66639	162.73158<br>127.33232	163.41991<br>147.24192	164.50660<br>164.78997	164.70717<br>165.13779
Cl-Ga-F	--X--	104.27835	88.96974	*94.25733*	*89.77807*	*92.61243*	71.83356	46.97072	--X--
Cl-Ga-N$_{(trans)}$	177.85099	177.87561	178.29177	*171.73253*	*171.10268*	*175.38837*	161.22986	133.32673	--X--
F-Ga-N$_{(trans)}$	--X--	135.37400	151.72349	*168.27645*	154.66656	*174.34337*	145.60227	175.83314	176.24347
**Energy**	**-3512.801938**	**-3612.832099**	**-3612.811626**	**-3612.838223**	**-3612.822992**	**-3612.838419**	**-3612.820963**	**-3612.849256**	**-3152.410228**
**Energy G**	**-3512.466085**	**-3612.499470**	**-3612.478747**	**-3612.505062**	**-3612.489977**	**-3612.504551**	**-3612.488509**	**-3612.516371**	**-3152.074653**

[GaCl(L)] + F^- = -3512.801938 + -100.027891 = **E = -3612.82983** & G = -3612.508134 au.
[GaF(L)] + Cl^- = -3152.410228 + -460.434655 = **E = -3612.844883** & G = -3612.524331 au.

1h) Solution-phase calculations: Image of each stationary point

[GaCl(L)]	RC
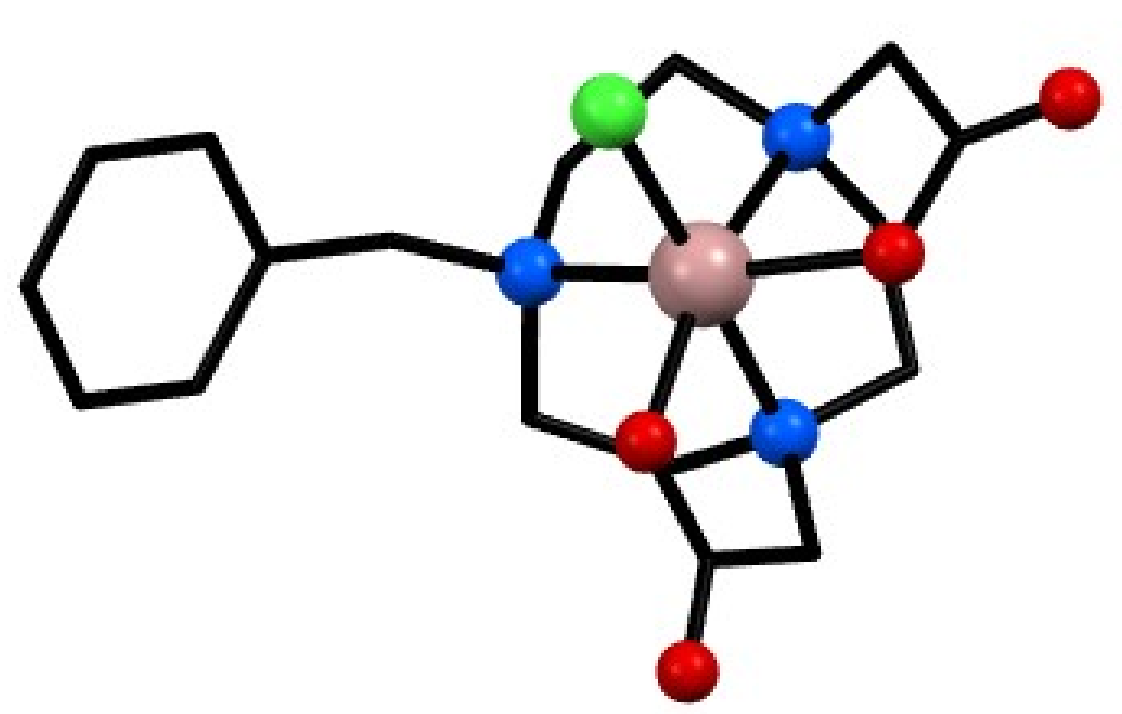	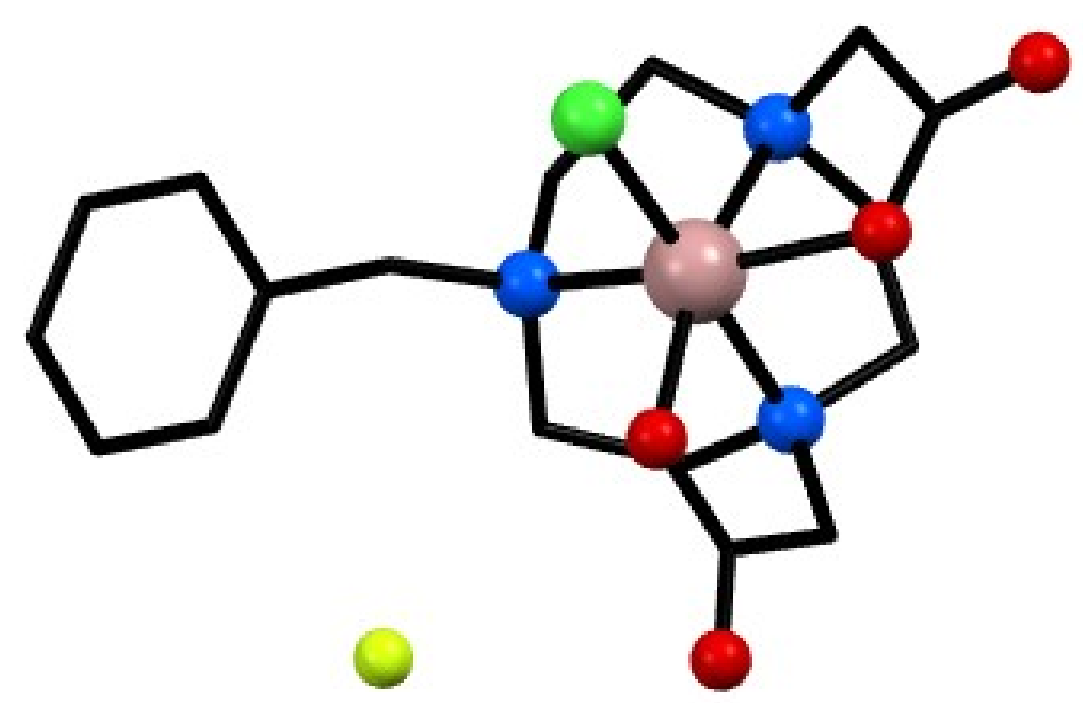
TS1	IC1

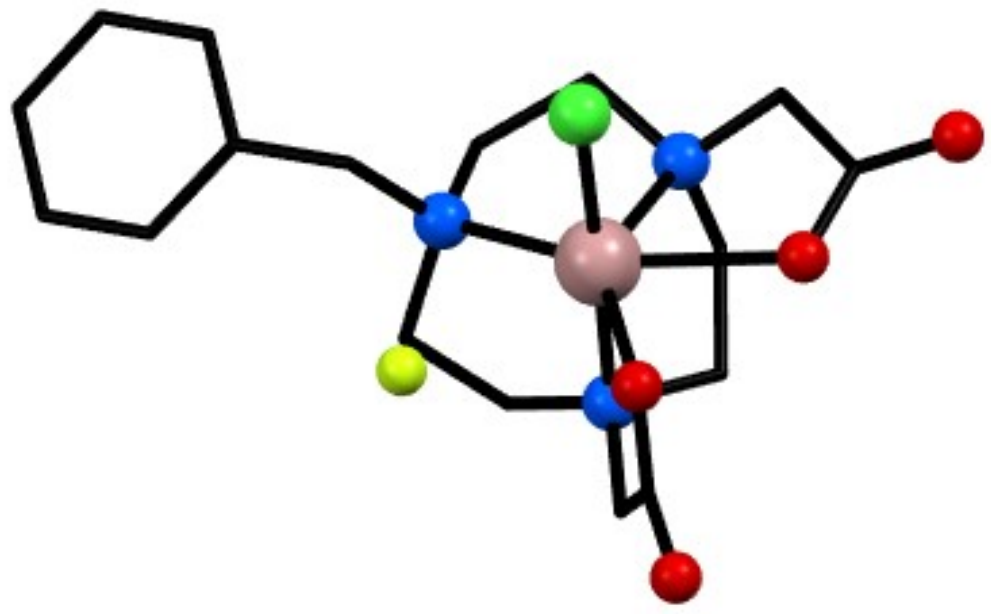	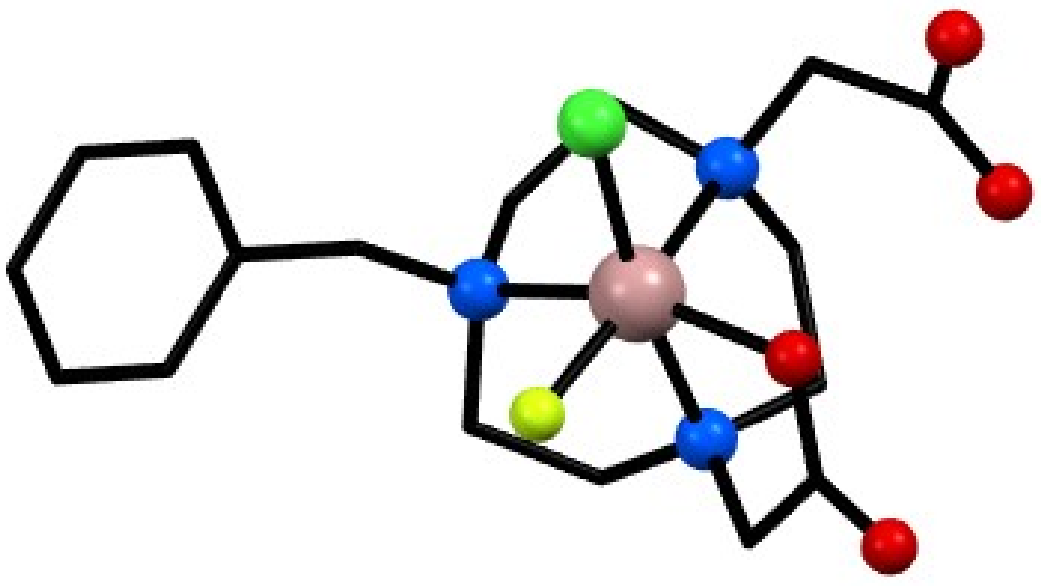
ICts	IC2
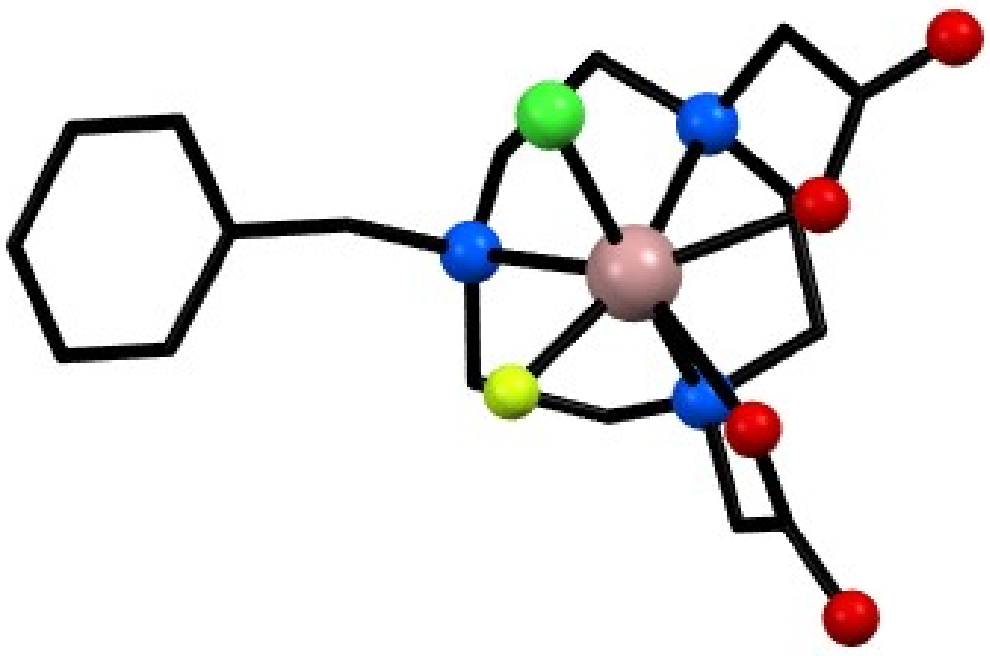	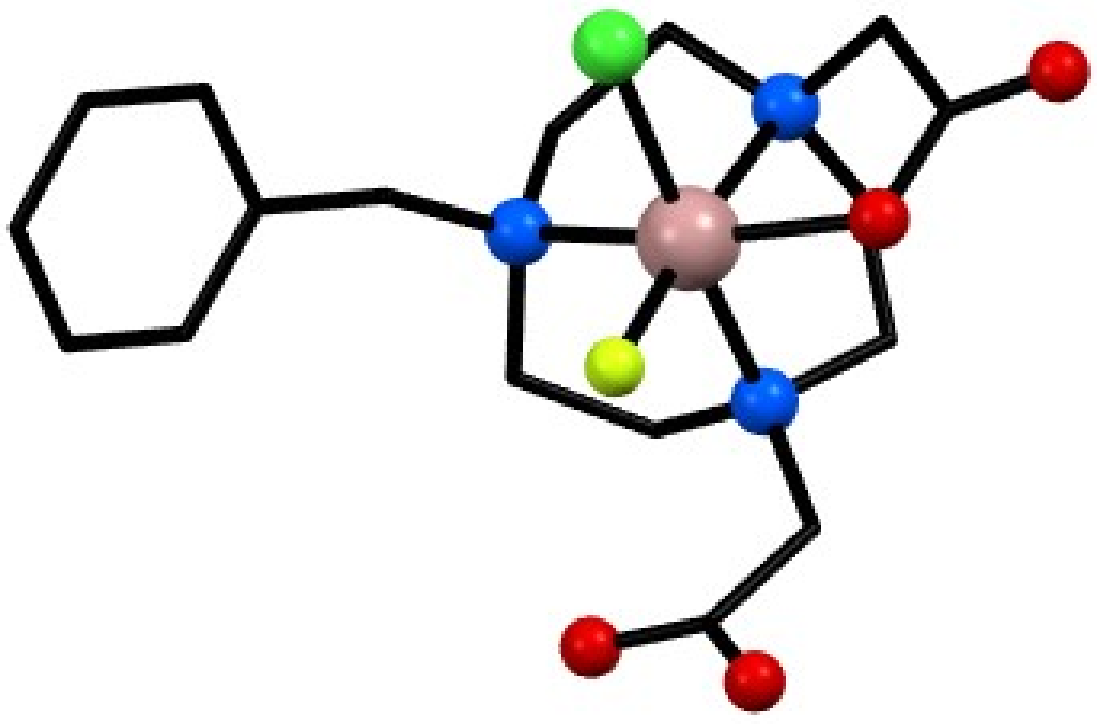
TS2	PC
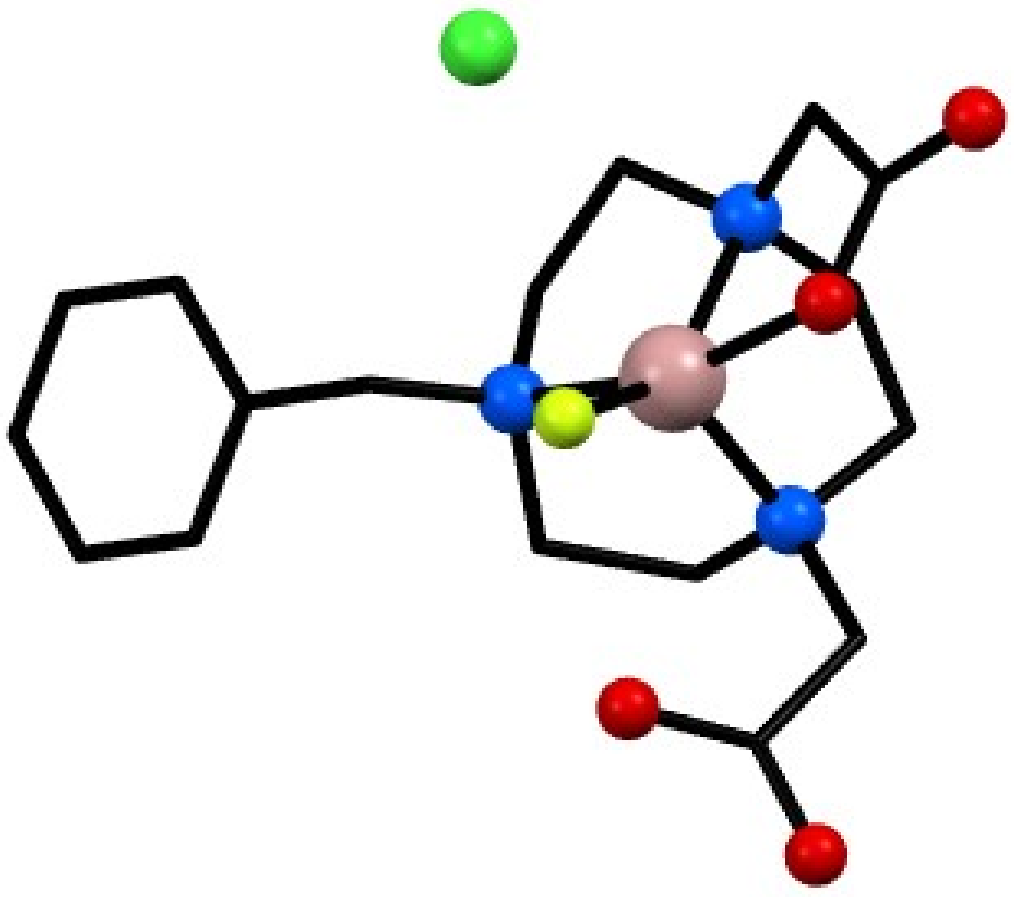	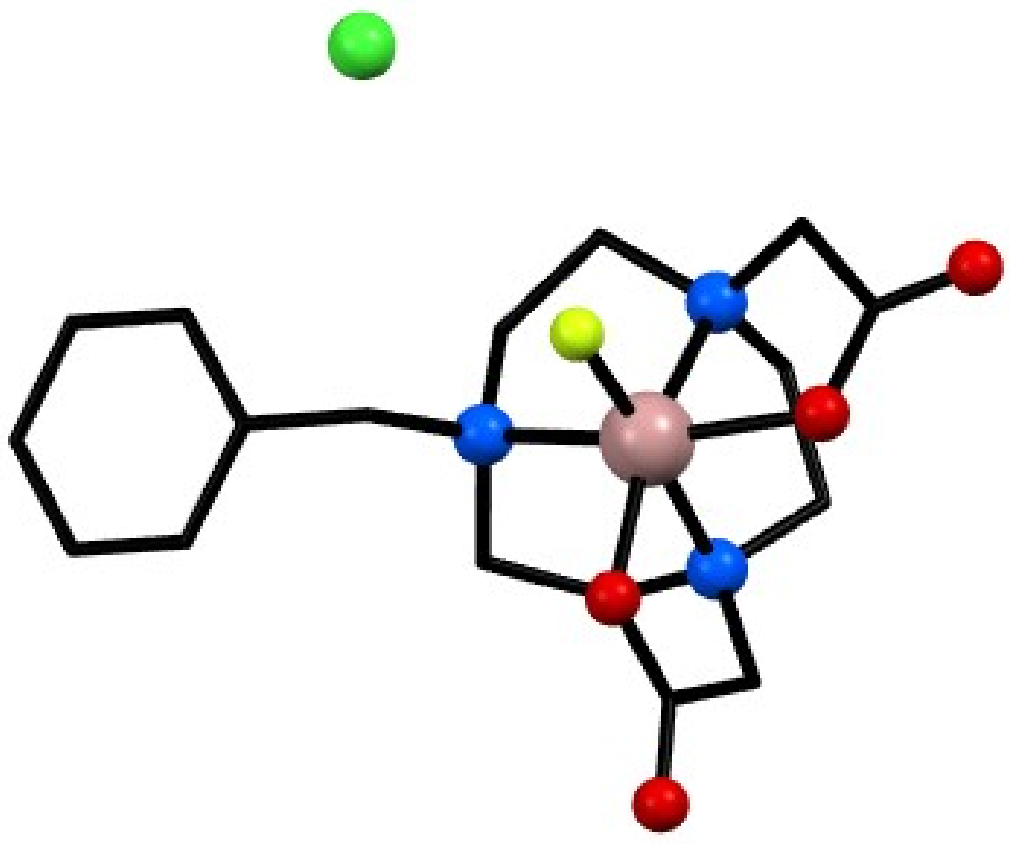
[GaF(L)]	
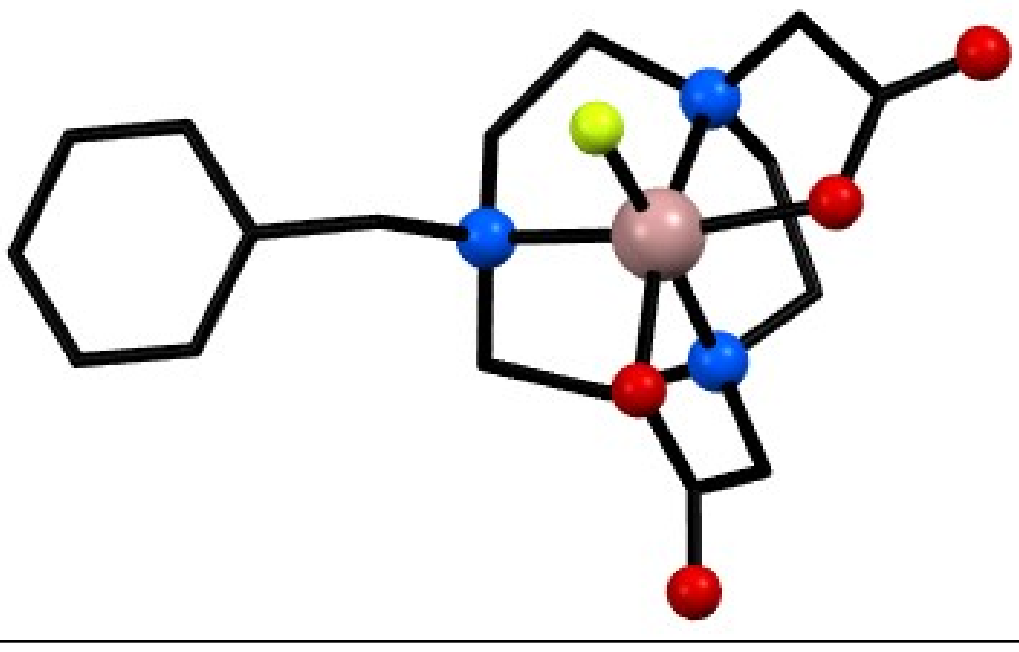	

**[GaCl(L)]**	**RC**
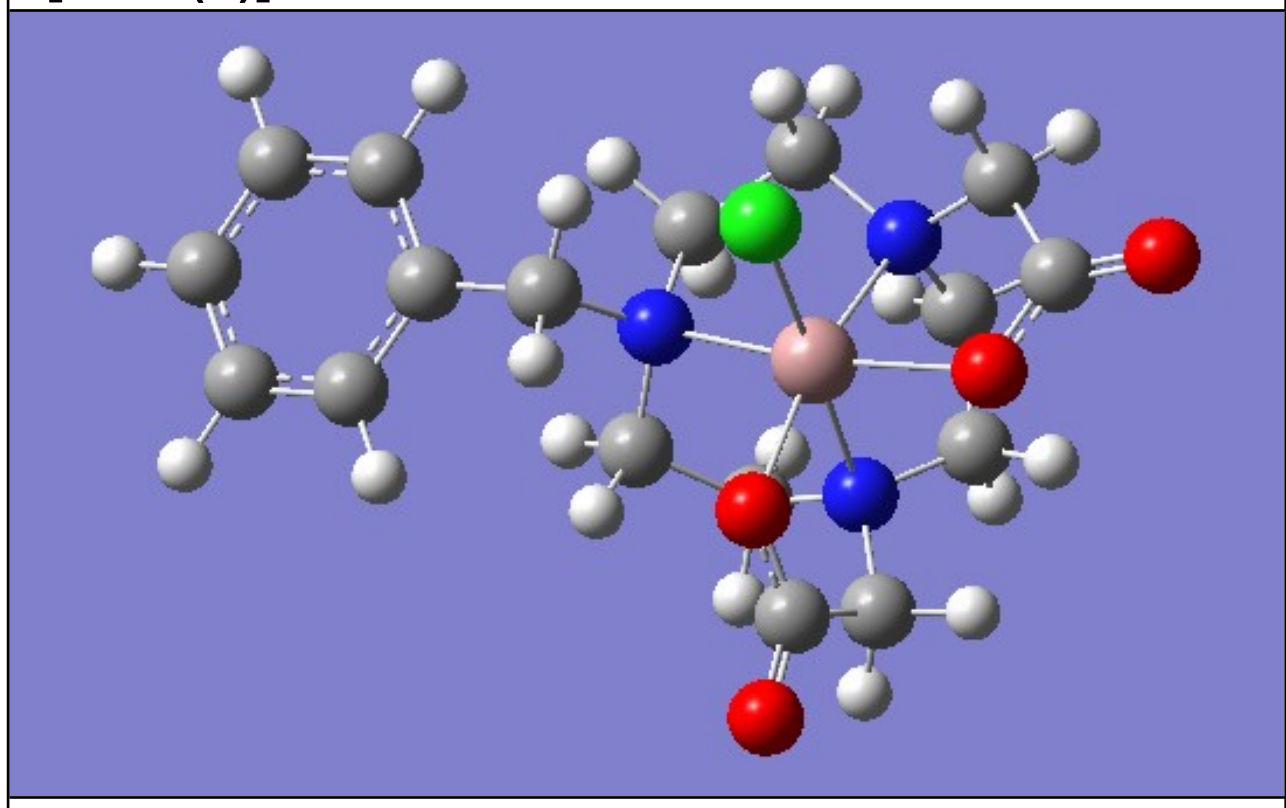	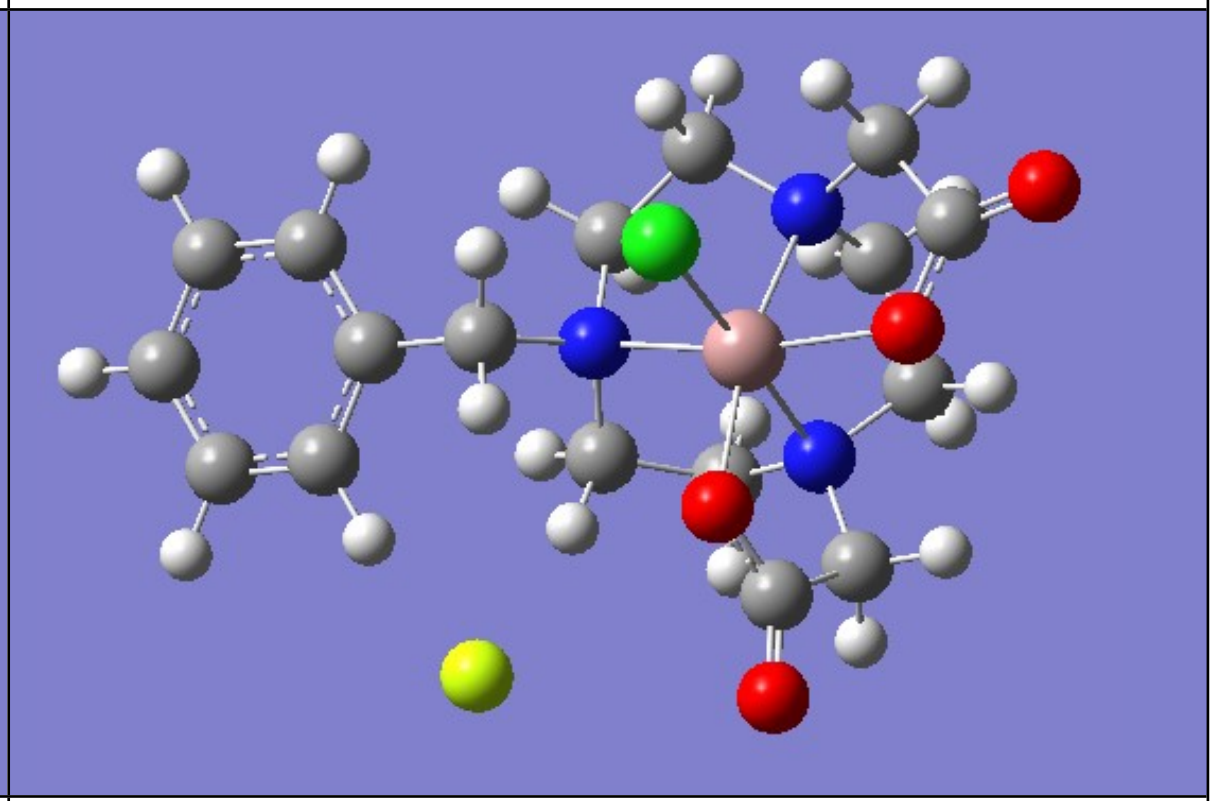
**TS1**	**IC1**
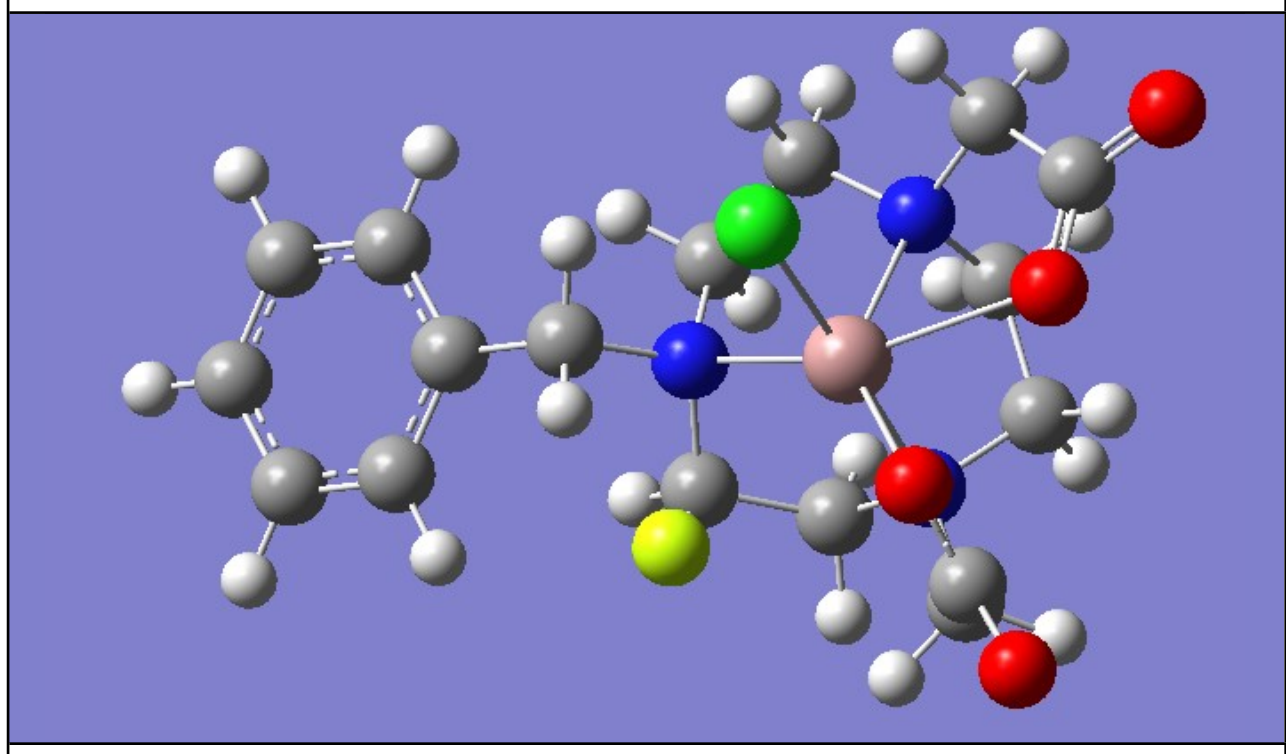	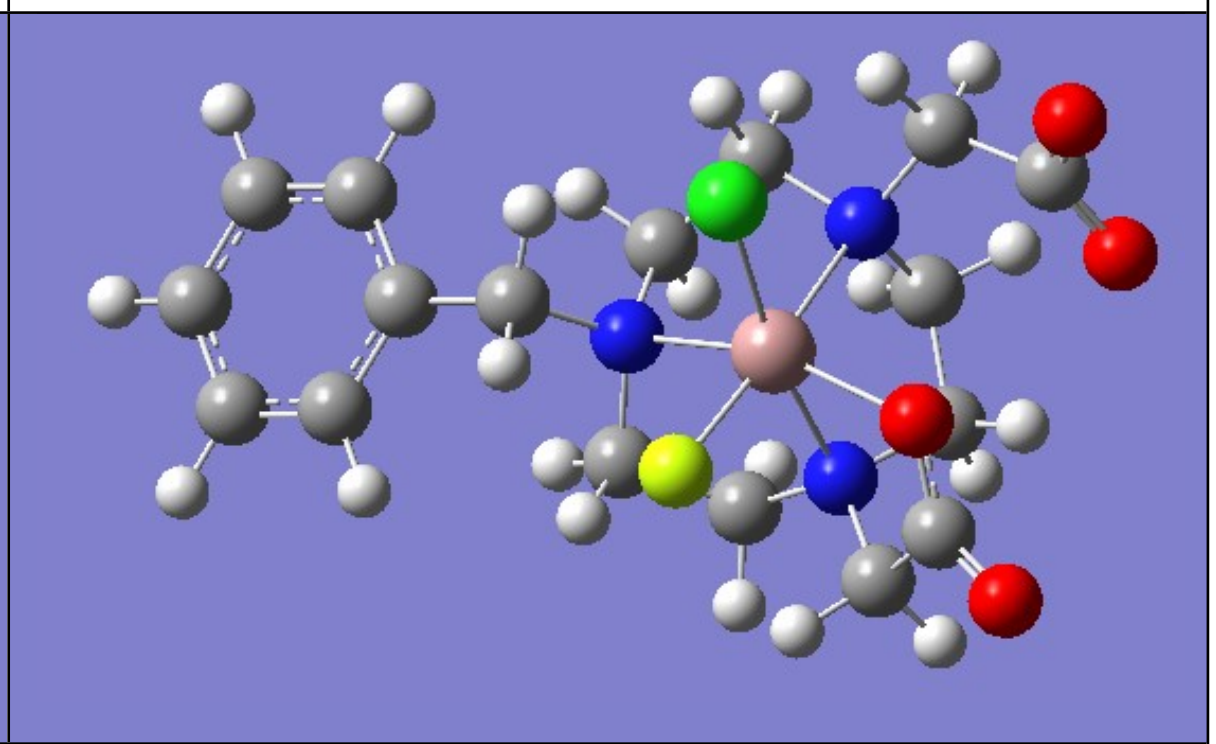
**ICts**	**IC2**
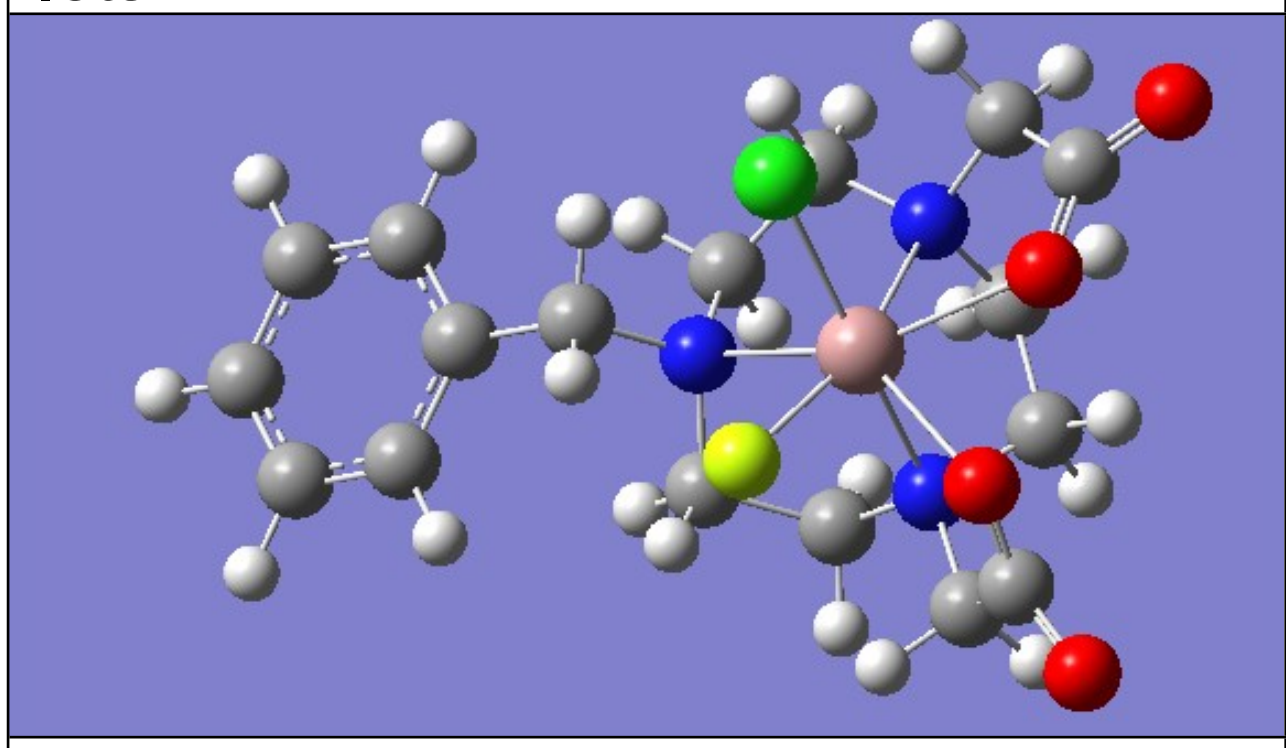	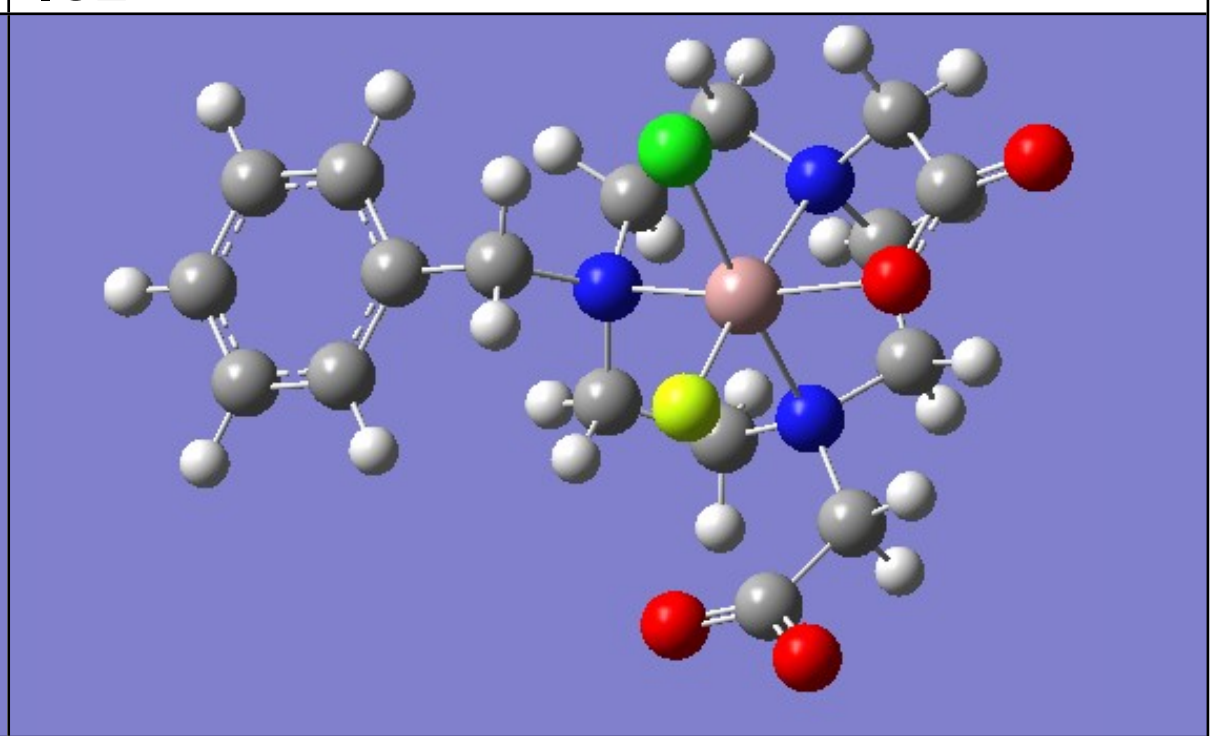
**TS2**	**PC**
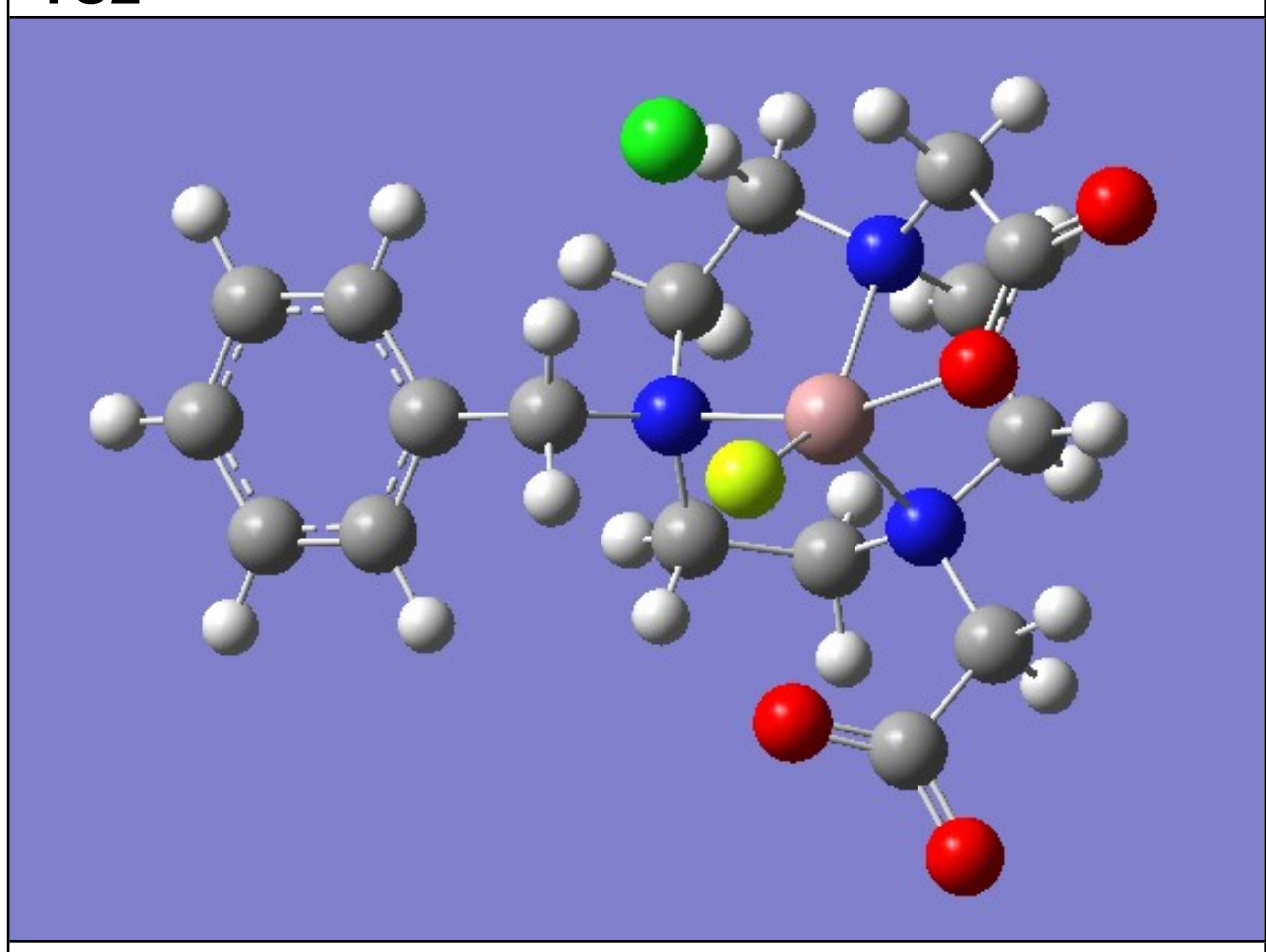	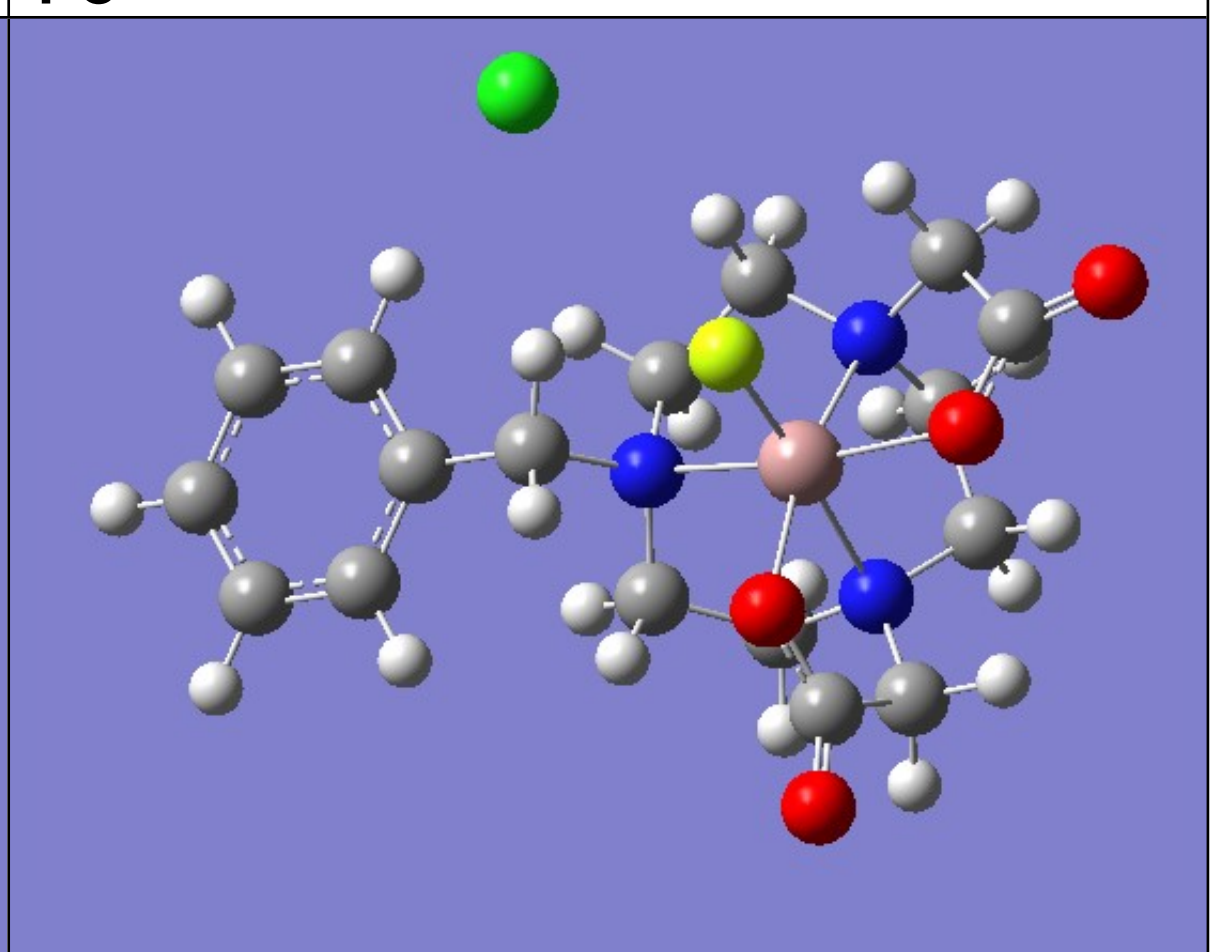
**[GaF(L)]**	

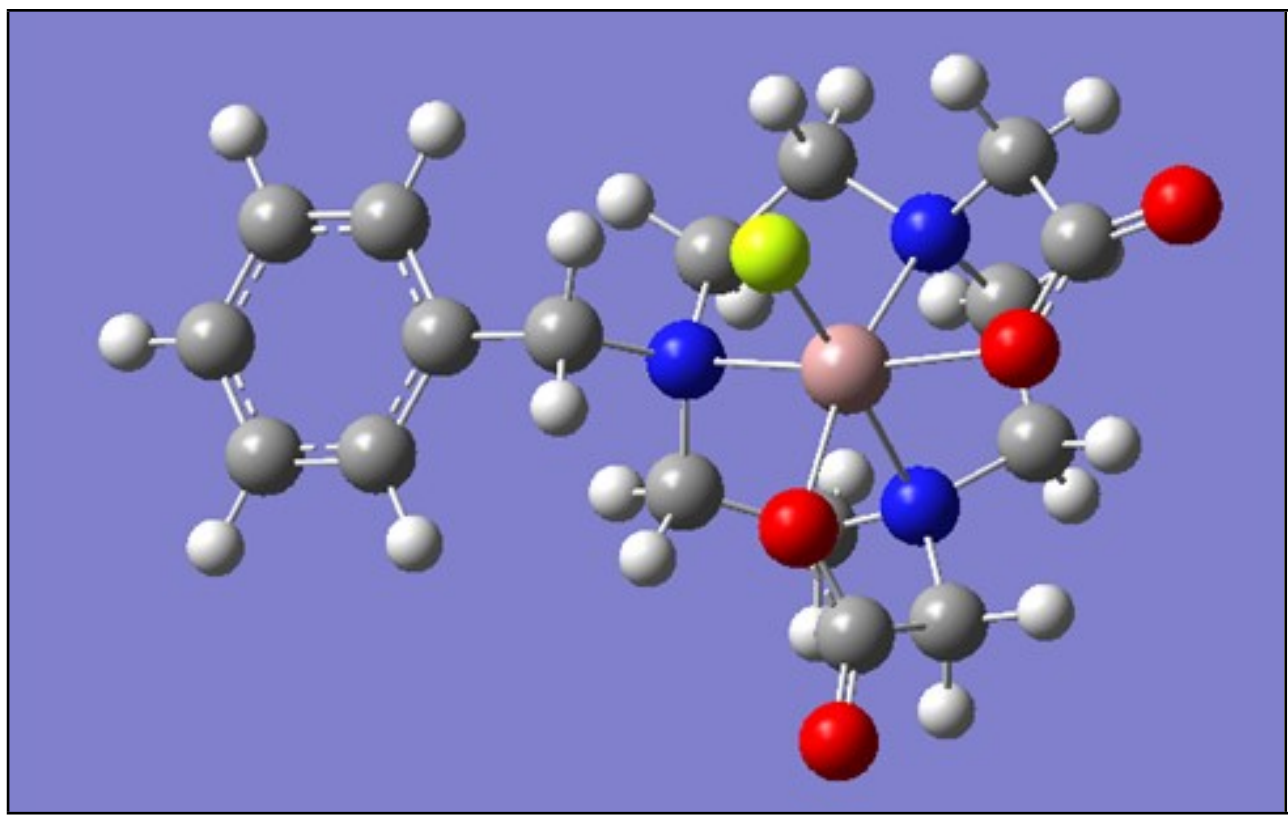

1i) Solution-phase calculations: Charge Density Table (Mulliken Analysis)

Mulliken charges of [GaCl(L)] + F^- geometries around Ga centre. In solution/6-311++G(d,p)

	[GaCl(L)]	RC	TS1	IC1	ICts	IC2	TS2	PC	[GaF(L)]
**Ga**	1.46647	1.51271	1.31480	1.34655	1.13723	1.573480	1.573197	2.246111	2.232263
**Cl**	-0.54546	-0.55532	-0.60535	-0.61478	-0.64473	-0.51902	-0.81915	-0.85264	--*X*--
**F**	--*X*--	-0.85969	-0.74615	-0.5871	-0.53697	-0.55191	-0.47927	-0.64774	-0.66119
**$O_{(3)}$**	-0.52157	-0.51899	-0.50027	-0.51566	-0.45102	-0.54903	-0.41974	-0.65109	-0.65119
**$O_{(8)}$**	-0.49942	-0.41065	-0.47740	-0.45121	-0.53574	-0.53248	-0.51524	-0.63544	-0.64002
**$N_{(5)}$**	-0.10288	-0.10846	-0.25653	-0.06296	-0.08490	-0.01687	+0.04152	-0.14784	-0.14683
**$N_{(6)}$**	-0.32072	-0.32270	-0.16377	-0.20701	-0.13671	-0.33687	-0.26231	-0.32643	-0.32237
**$N_{(15)}$**	-0.17032	-0.23434	0.01971	-0.01893	+0.0677	-0.24659	-0.12032	-0.19878	-0.25288

Atom numbers for N atoms and carboxylate atoms around Ga vary slightly between geometries, reported values are the equivalent positions, with the carboxylate oxygens being the closest to the metal centre.

2. Supplementary data for [AlCl(L)] + F^- → [AlF(L)] +Cl^- reaction

2a) Comparison to crystal structure [AlF(Bn-NODA)]·$2H_2O$]

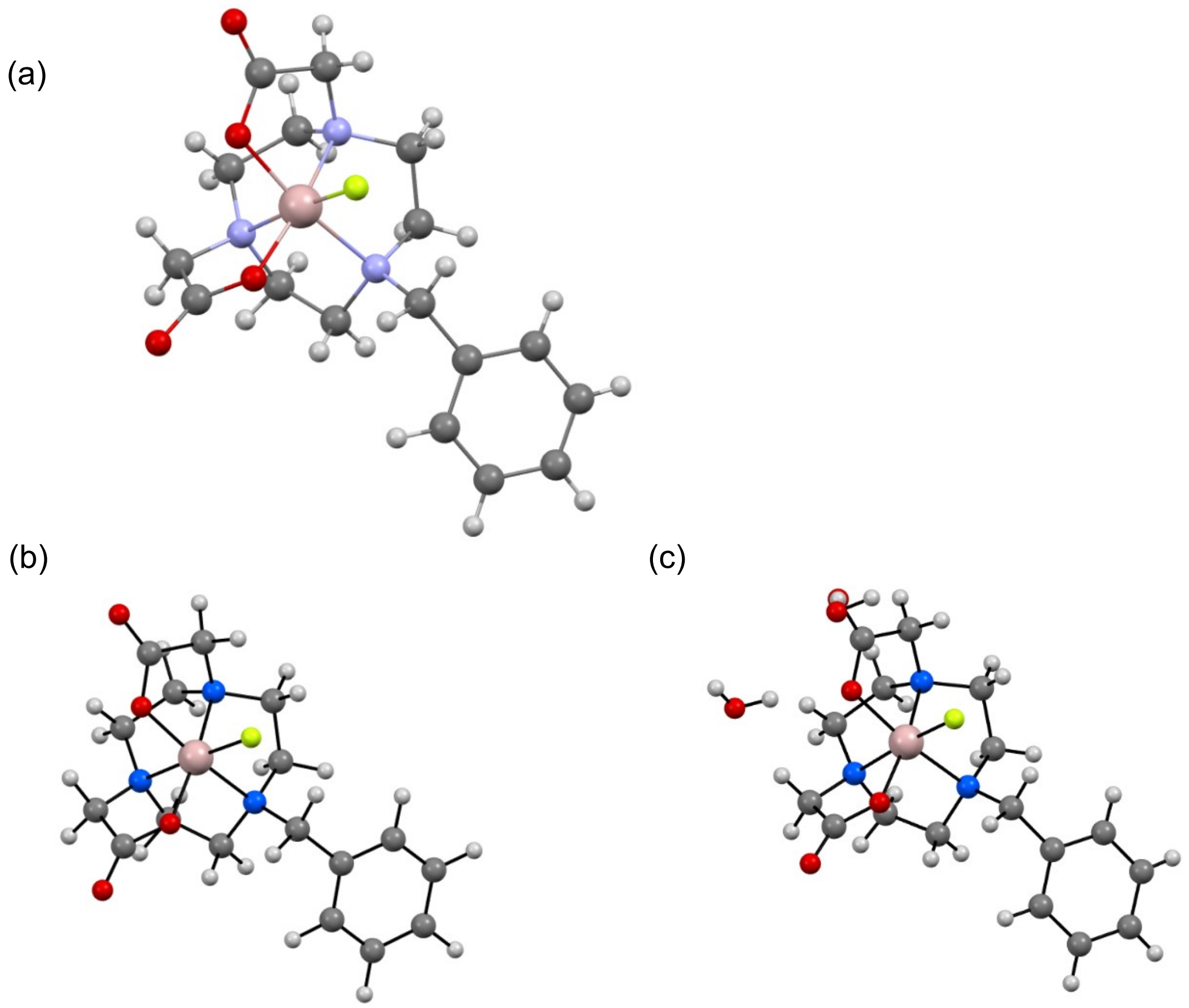

Geometries of [AlF(Bn-NODA)], (a) crystal structure (lattice water molecules are omitted) from ref. 8, (b) optimized geometry of the [AlF(Bn-NODA)] molecule in the gas phase, and (c) optimized geometry of [AlF(Bn-NODA)] molecule with two H_2O molecules added; all calculations were performed at the BP86-D3/6-311++G(d,p) level.

2b) Solution-phase calculations: Geometry Tables

Note: Cl/OH-Al-$N_{(trans)}$ values are taken as the closest to 180º, Al-$N_{(trans)}$ is the N atom trans to Cl. Al-O taken as the shortest Al-O distance from each carboxylate arm.

	[AlCl(L)]	RC	TS1	IC1	ICts	IC2	TS2	PC	[AlF(L)]
Al-Cl	2.27408	2.27549	2.23783	2.30607	2.33783	2.35059	3.69034	5.18612	--X--
Al-F	--X--	4.93389	2.67597	1.81536	1.79929	1.75857	1.72843	1.76460	1.76736
Al-O	1.92511<br>1.88389	1.88375<br>1.92996	1.93123<br>2.11948	2.11745<br>2.06374	2.03571<br>2.24964	1.92070<br>4.07461	1.88953<br>3.04048	1.93305<br>1.89208	1.92933<br>1.88779
Al-N	2.11584<br>2.07135	2.07424<br>2.11130	2.28234<br>2.17679	2.55916<br>2.30733	2.46494<br>2.27703	2.11439<br>2.10618	2.07676<br>2.06657	2.10144<br>2.06575	2.10434<br>2.06779
Al-$N_{(trans)}$	2.09744	2.09588	2.09196	2.11021	2.12148	2.12524	2.02098	2.10224	2.10244
N-Al-O	165.87812<br>166.27117	165.77901<br>166.36677	151.15084<br>144.33986	142.46075<br>141.98165	140.81699<br>146.19992	164.36491<br>128.02509	165.17919<br>149.78513	166.39421<br>165.93867	166.57613<br>166.06697
Cl-Al-F	--X--	106.98314	88.18960	89.73994	89.30773	93.37603	69.05943	44.36788	--X--
Cl-Al-$N_{(trans)}$	178.55368	178.60426	174.54314	167.23741	166.43840	174.59650	161.77578	133.92234	--X--
F-Al-$N_{(trans)}$	--X--	133.79791	147.41908	143.29714	149.04968	174.85914	143.16094	176.70999	176.14123
**Energy**	-1830.249307	-1930.279341	-1930.249621	-1930.269165	-1930.268748	-1930.291939	-1930.270782	-1930.311128	-1469.872386
**Energy G**	-1829.909922	-1929.942918	-1929.912444	-1929.934027	-1929.933075	-1929.955236	-1929.934989	-1929.975099	-1469.532764

[AlCl(L)] + F^- = -1830.249307 + -100.02789 = **E = -1930.277197** & G = -1929.951971 au.
[AlF(L)] + Cl^- = -1469.872386 + -460.434655 = **E = -1930.307041** & G = -1929.982442 au.

2c) Solution-phase calculations: Image of each stationary point

[AlCl(L)]

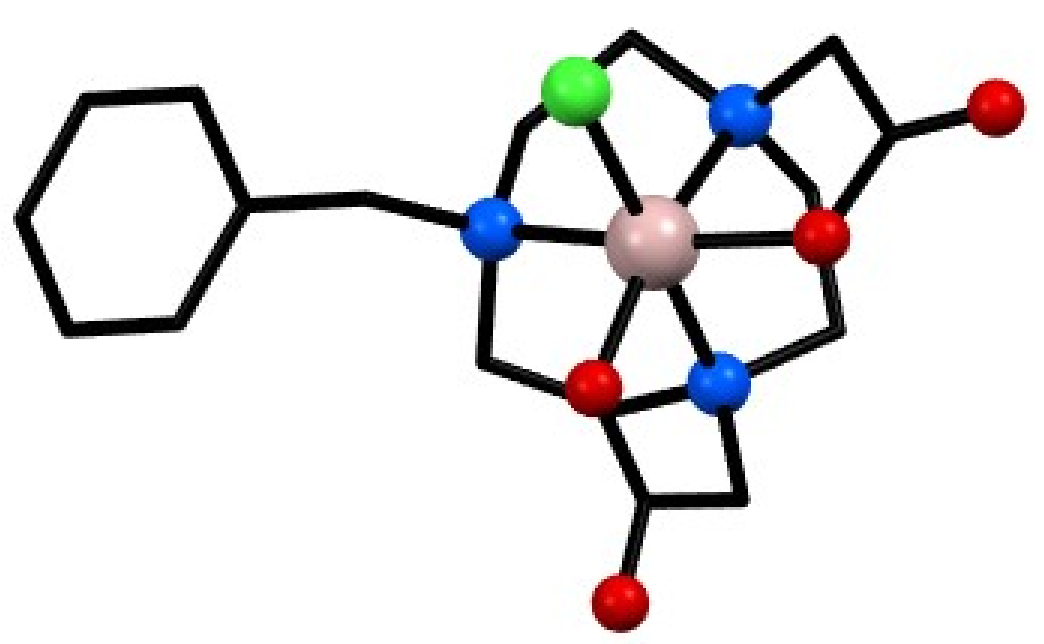

RC

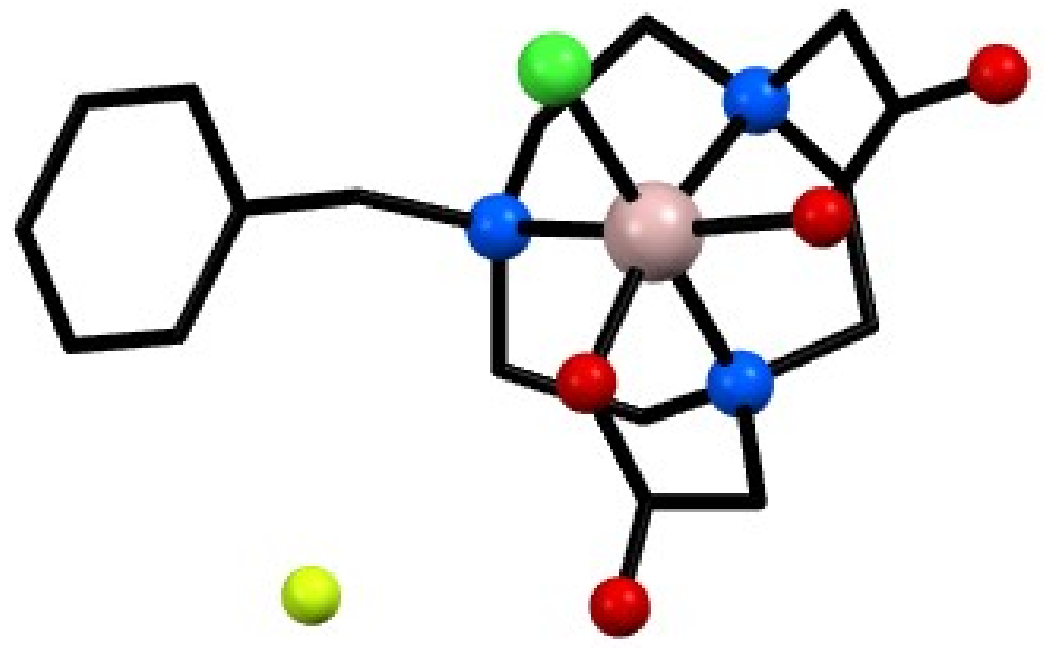

TS1

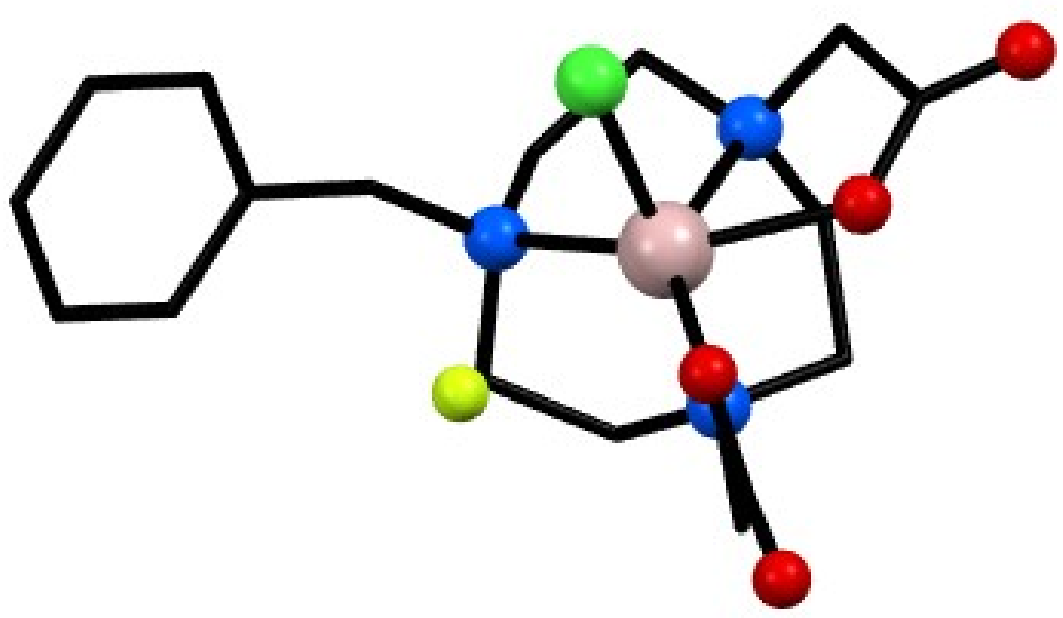

IC1

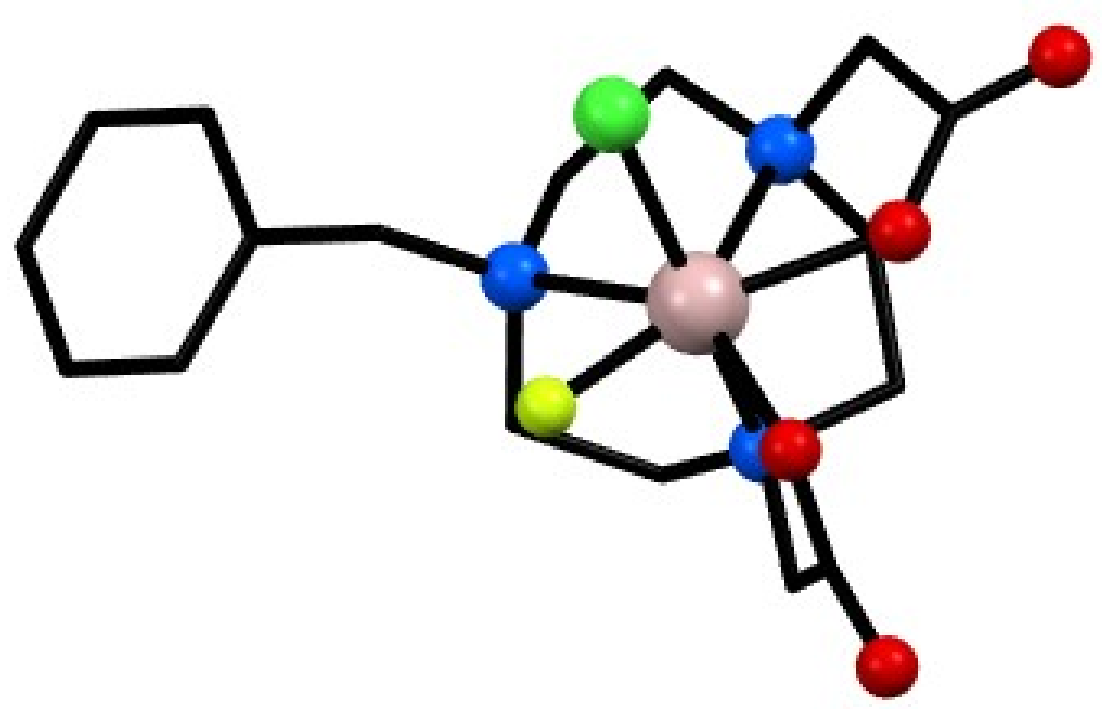

ICts

IC2

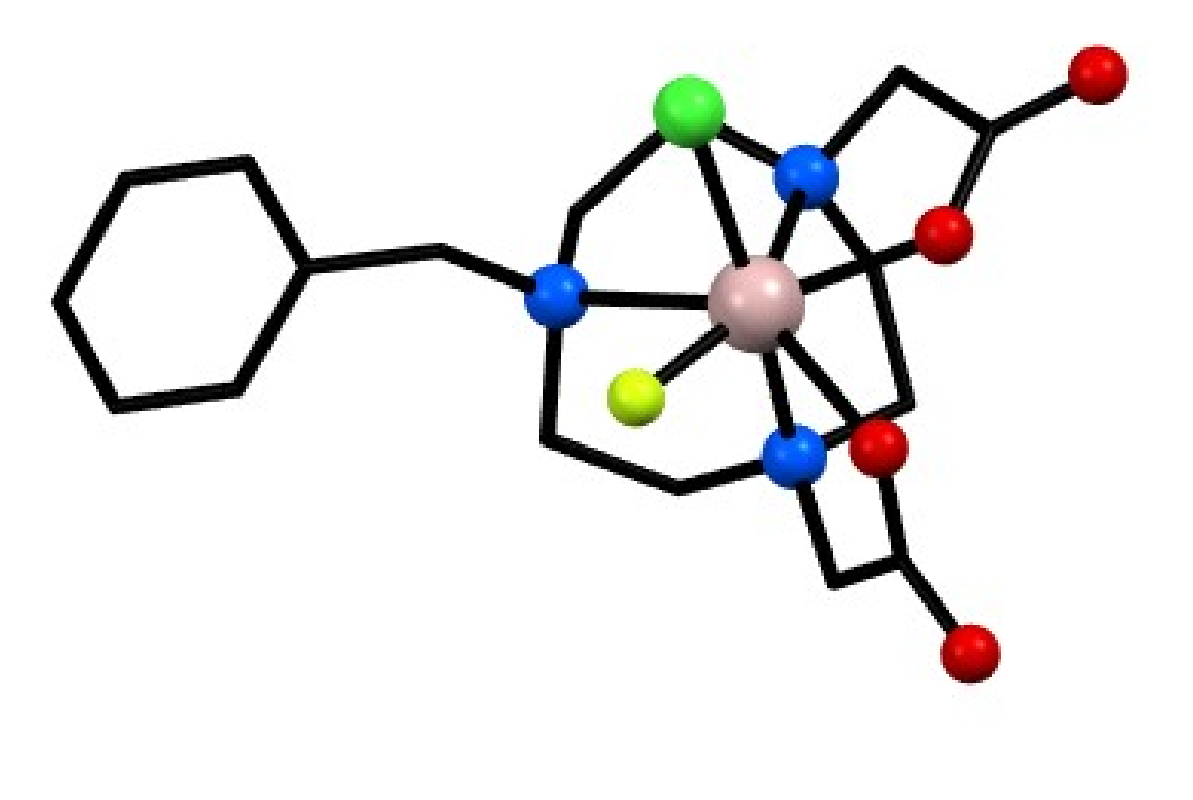	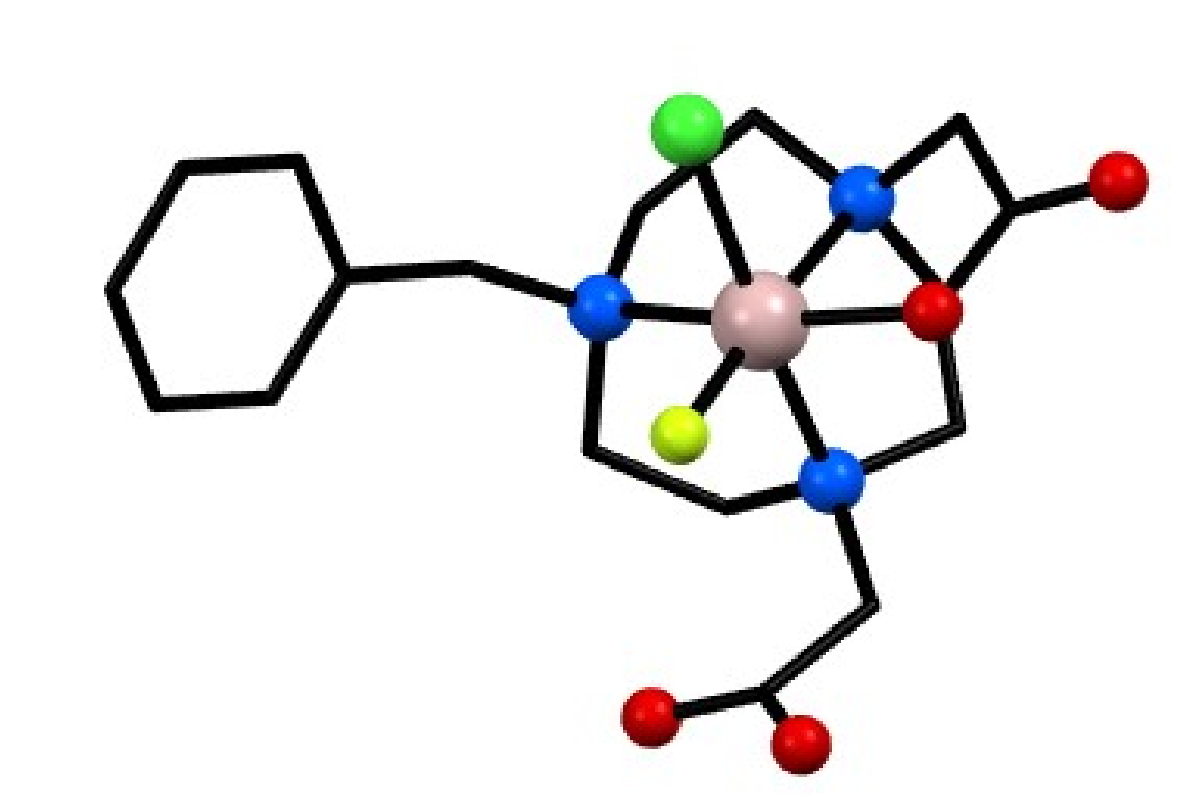
TS2	PC
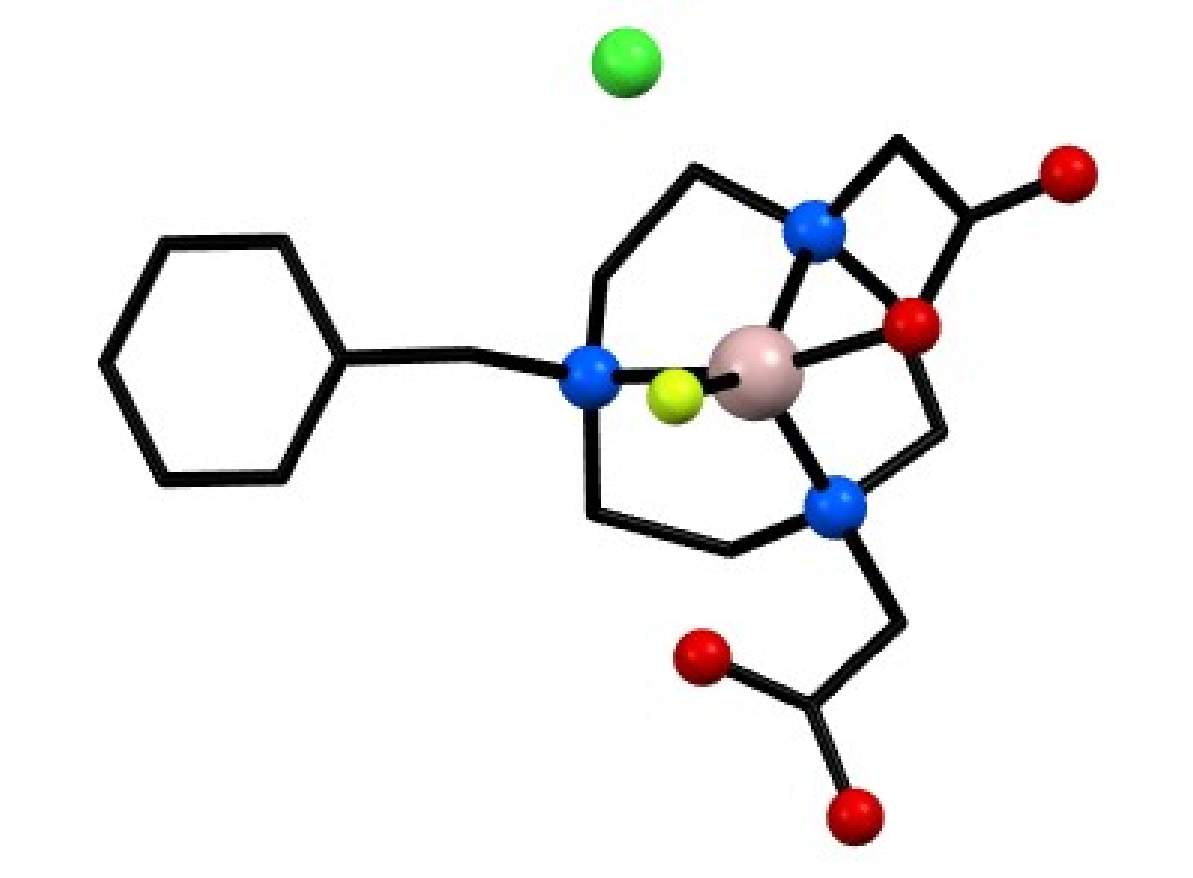	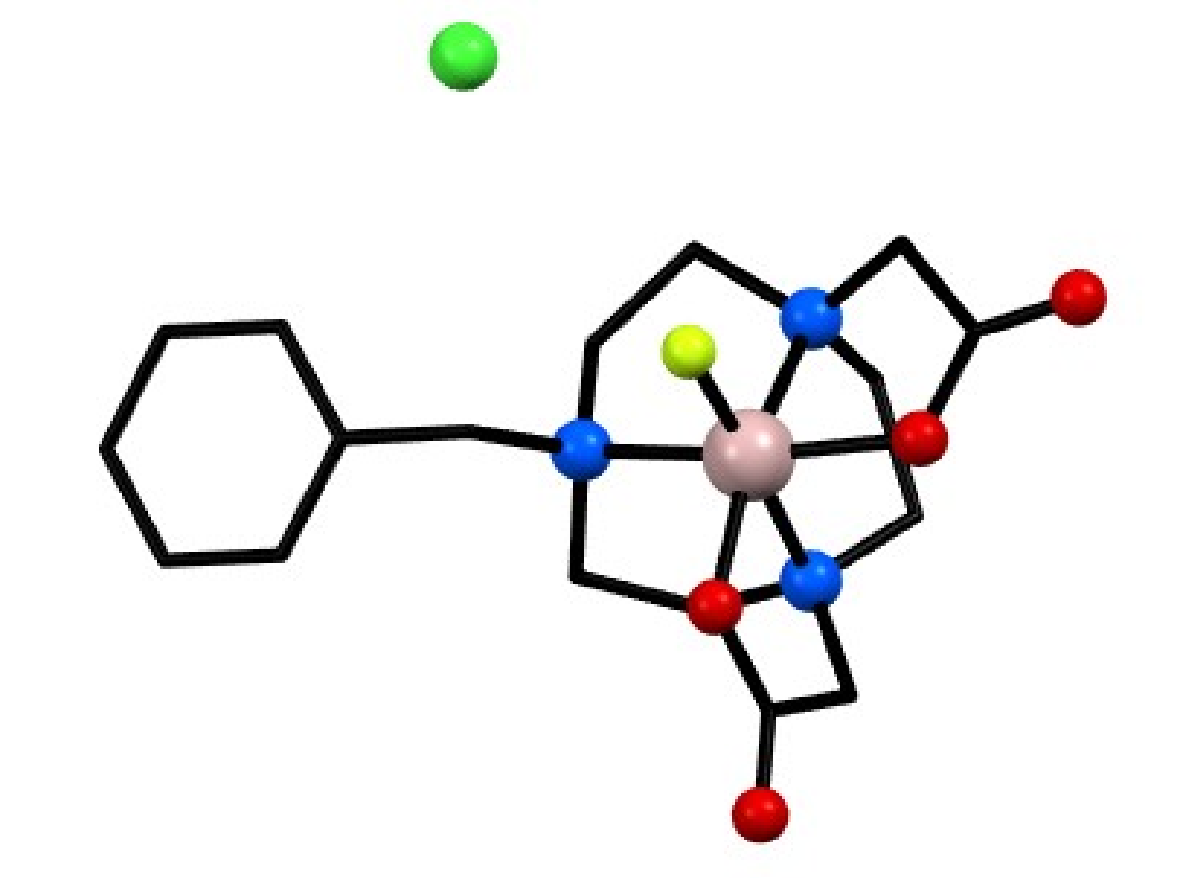
[AlF(L)]	
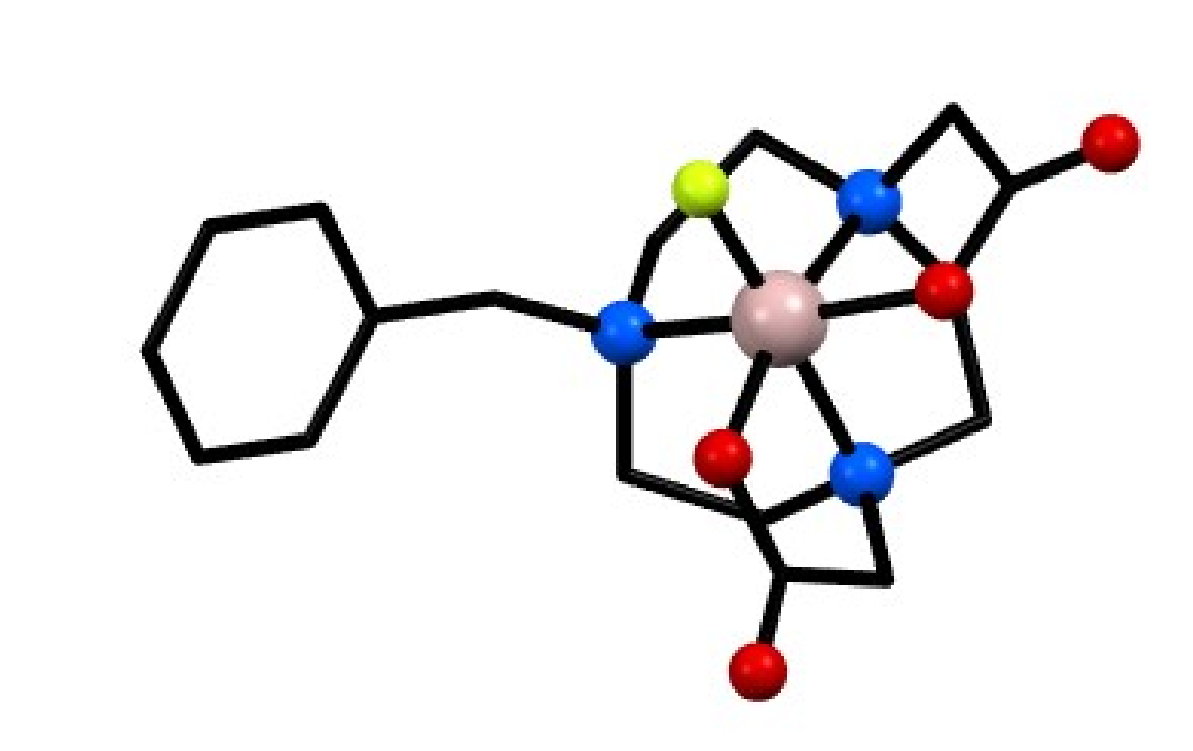	

**[AlCl(L)]**	**RC**
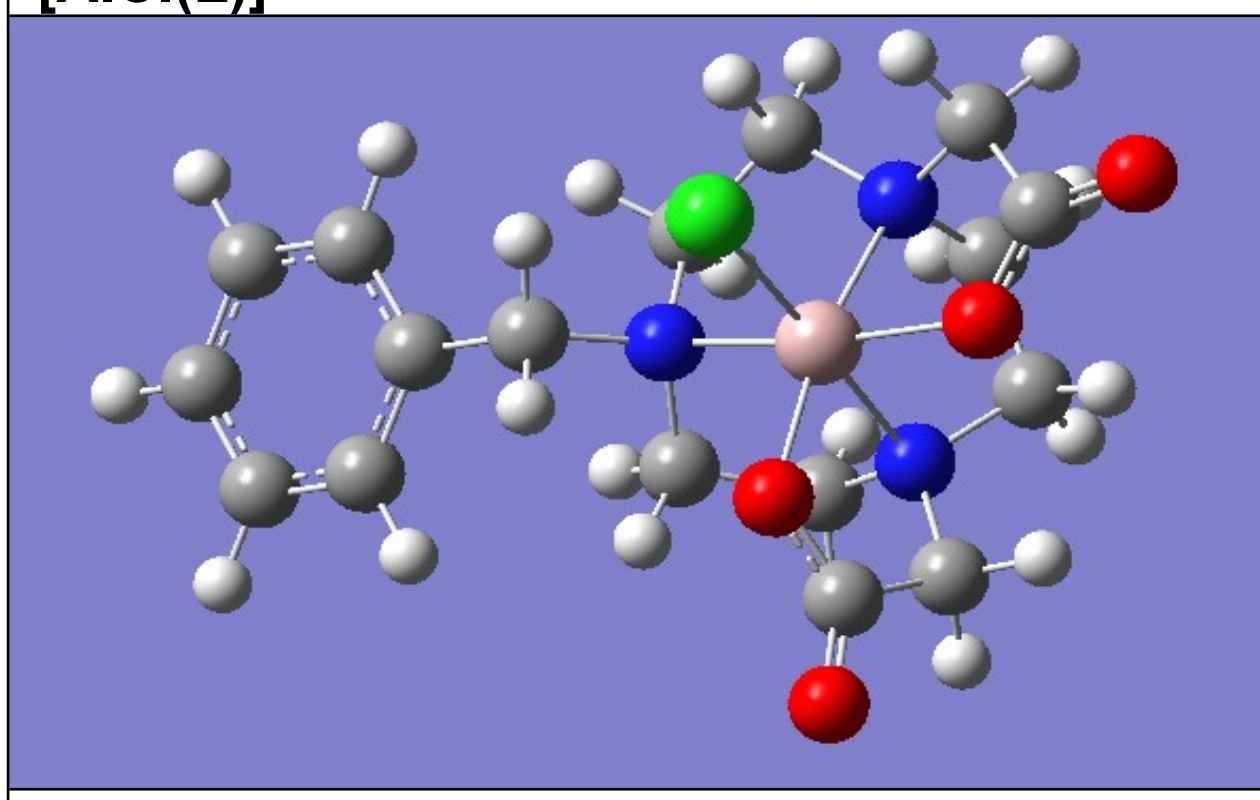	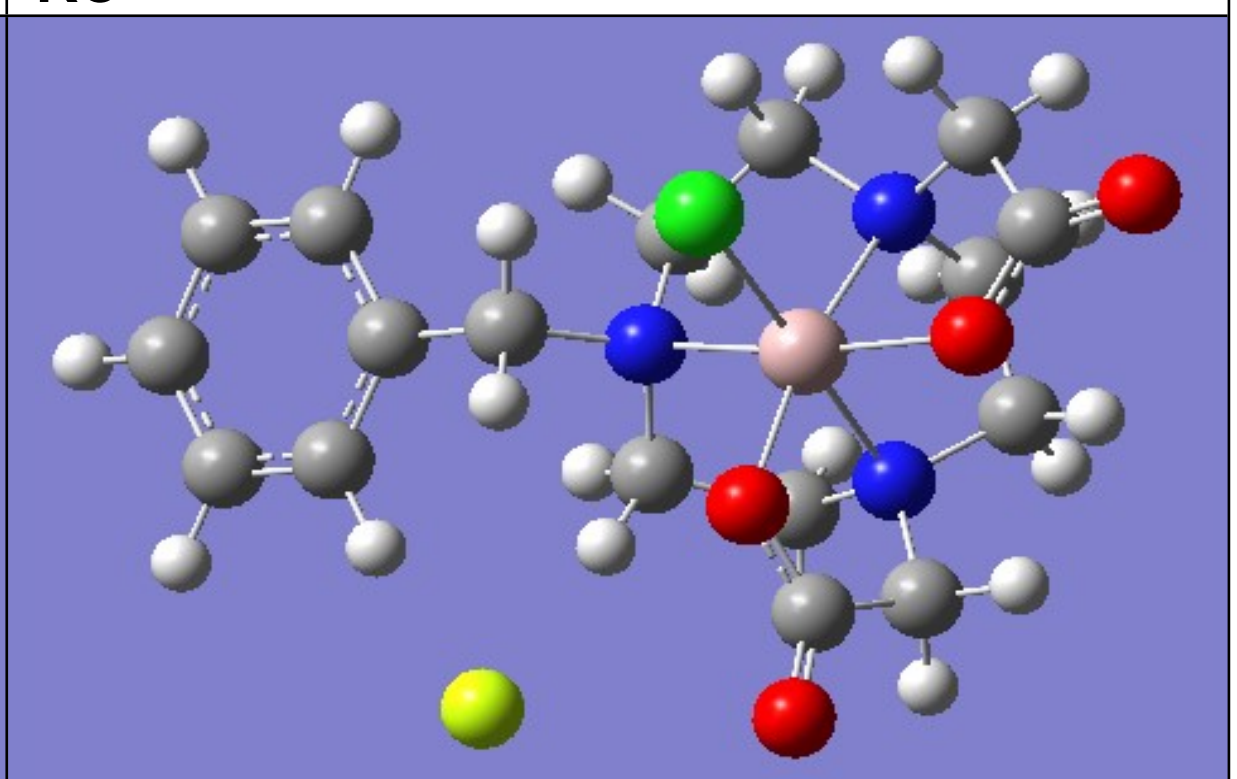
**TS1**	**IC1**

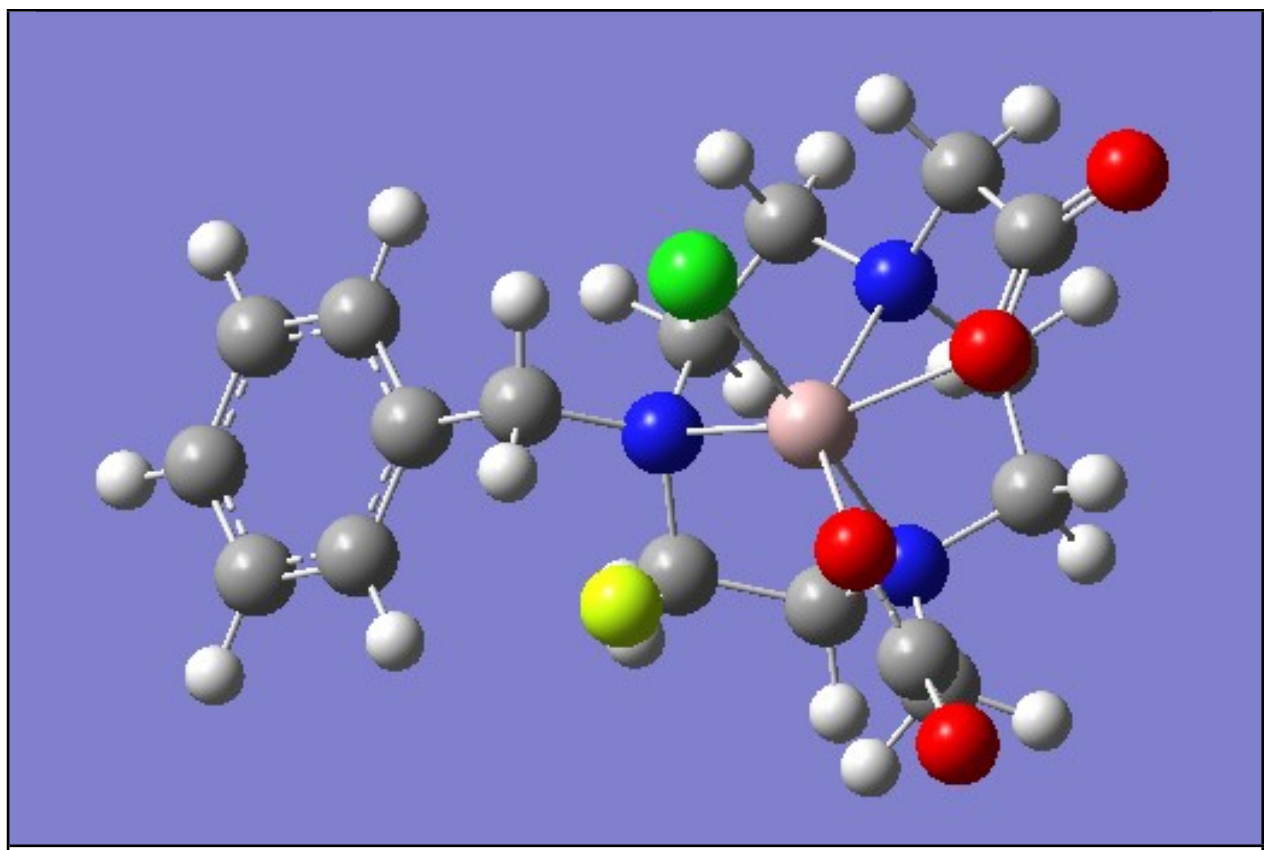	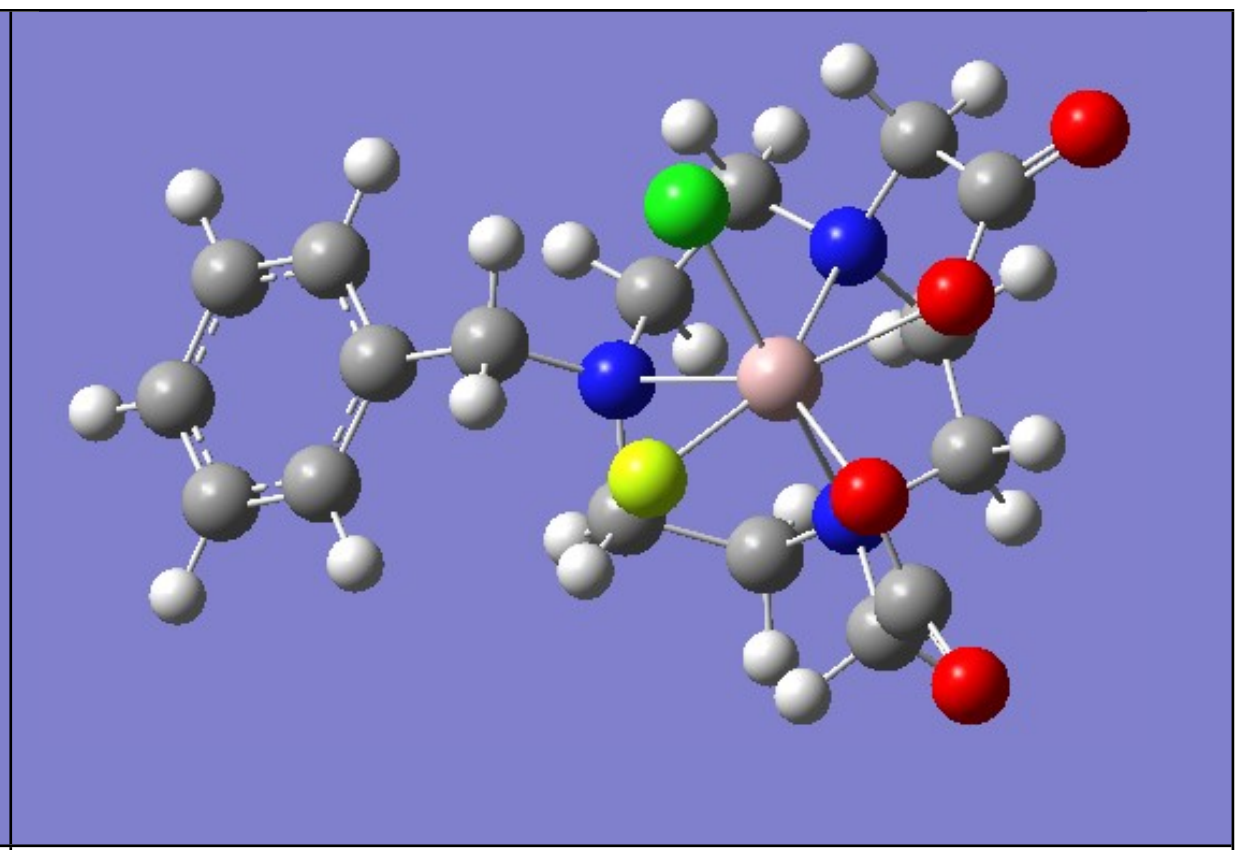
**ICts**	**IC2**
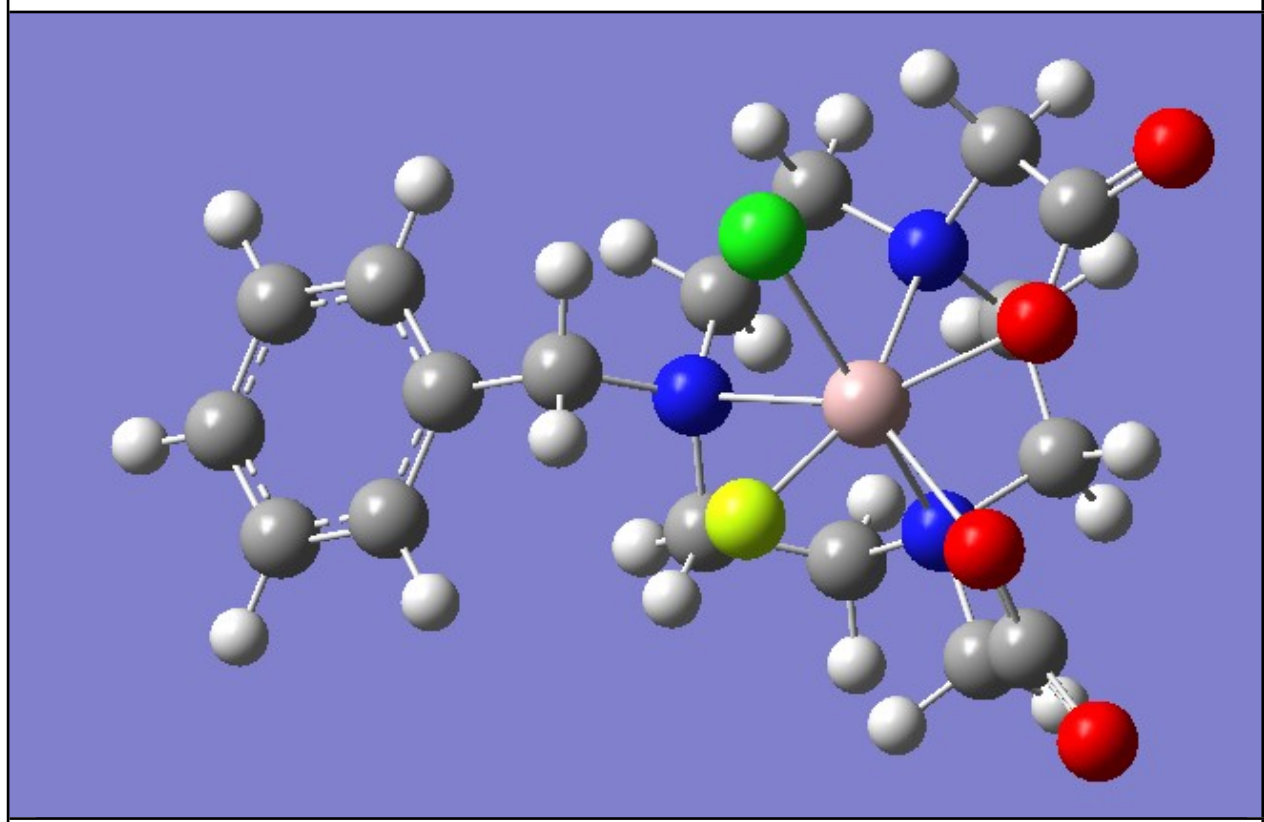	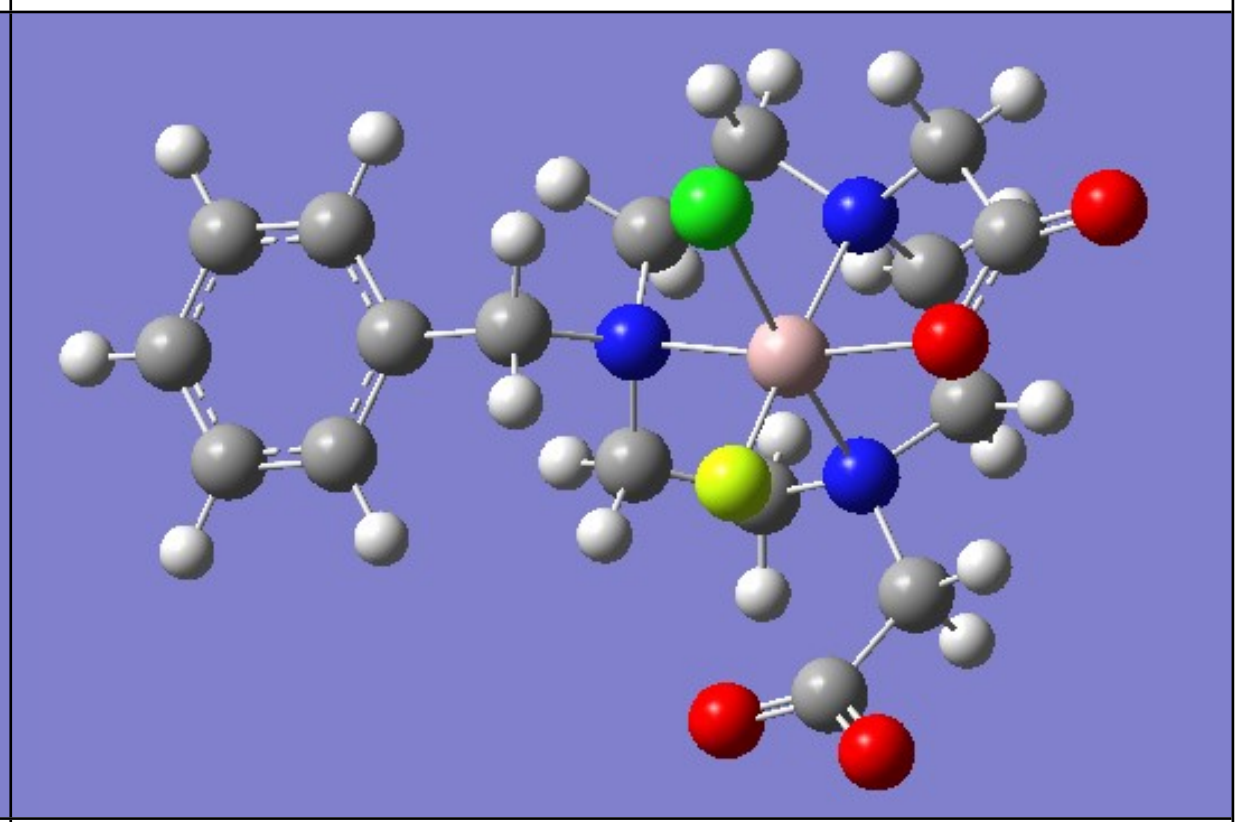
**TS2**	**PC**
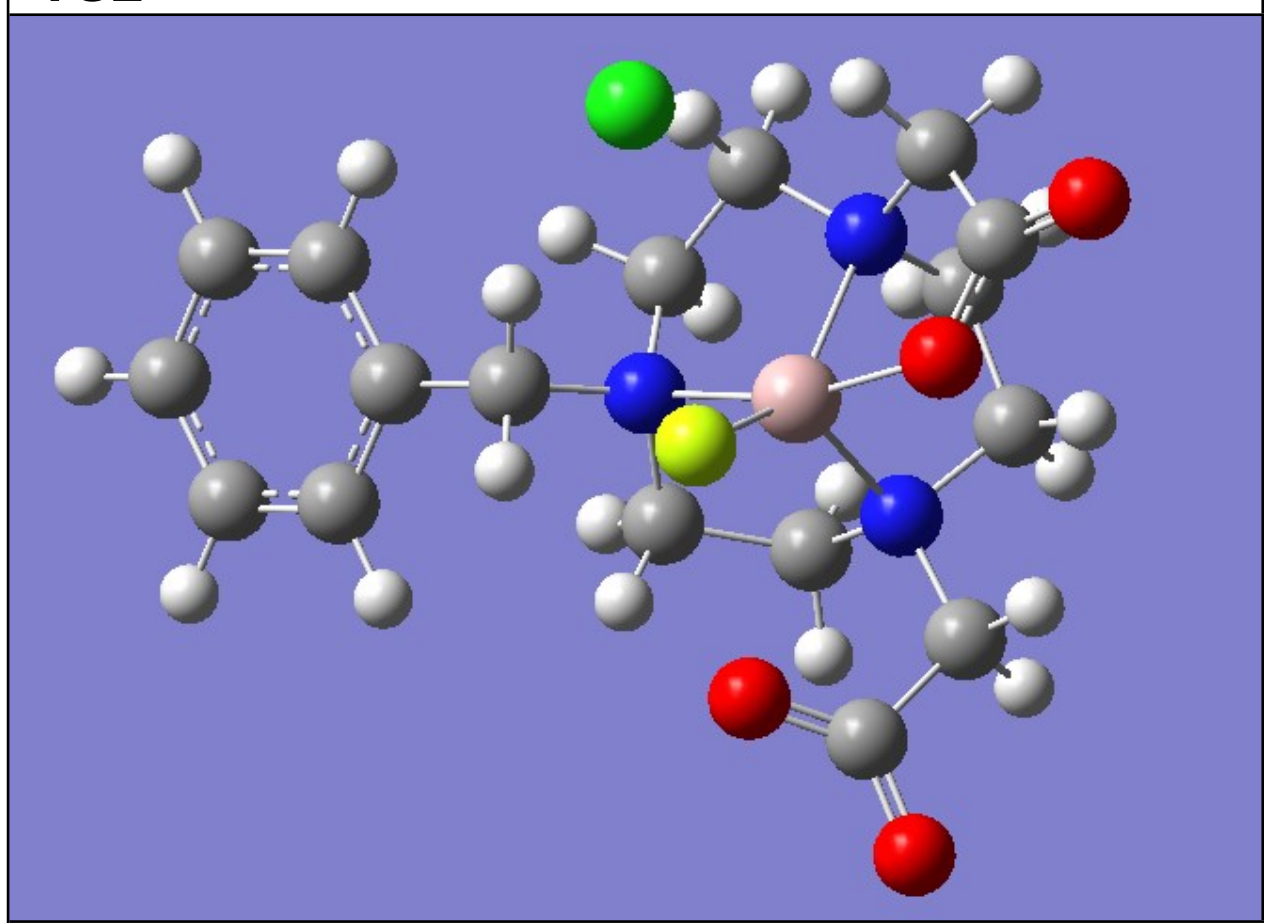	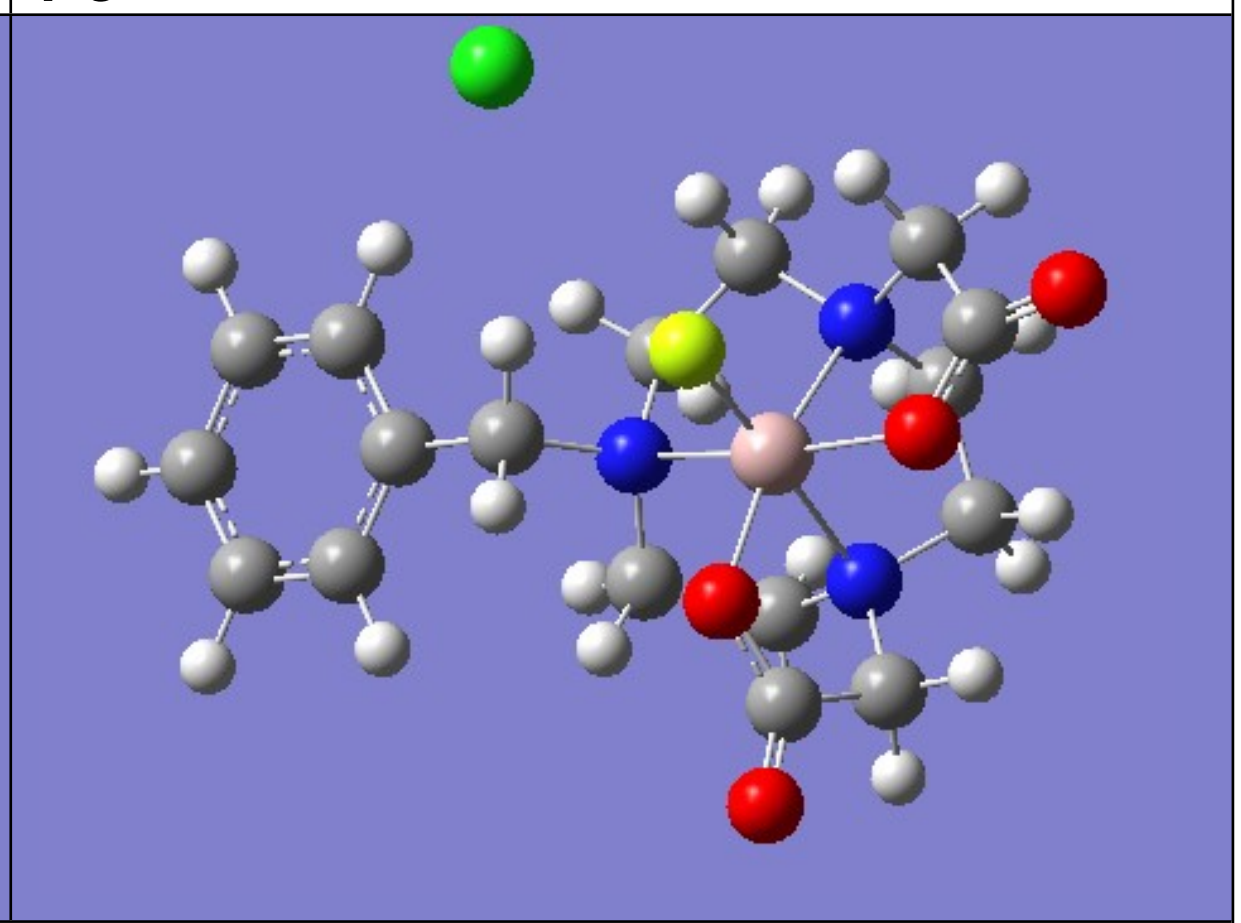
**[AlF(L)]**	
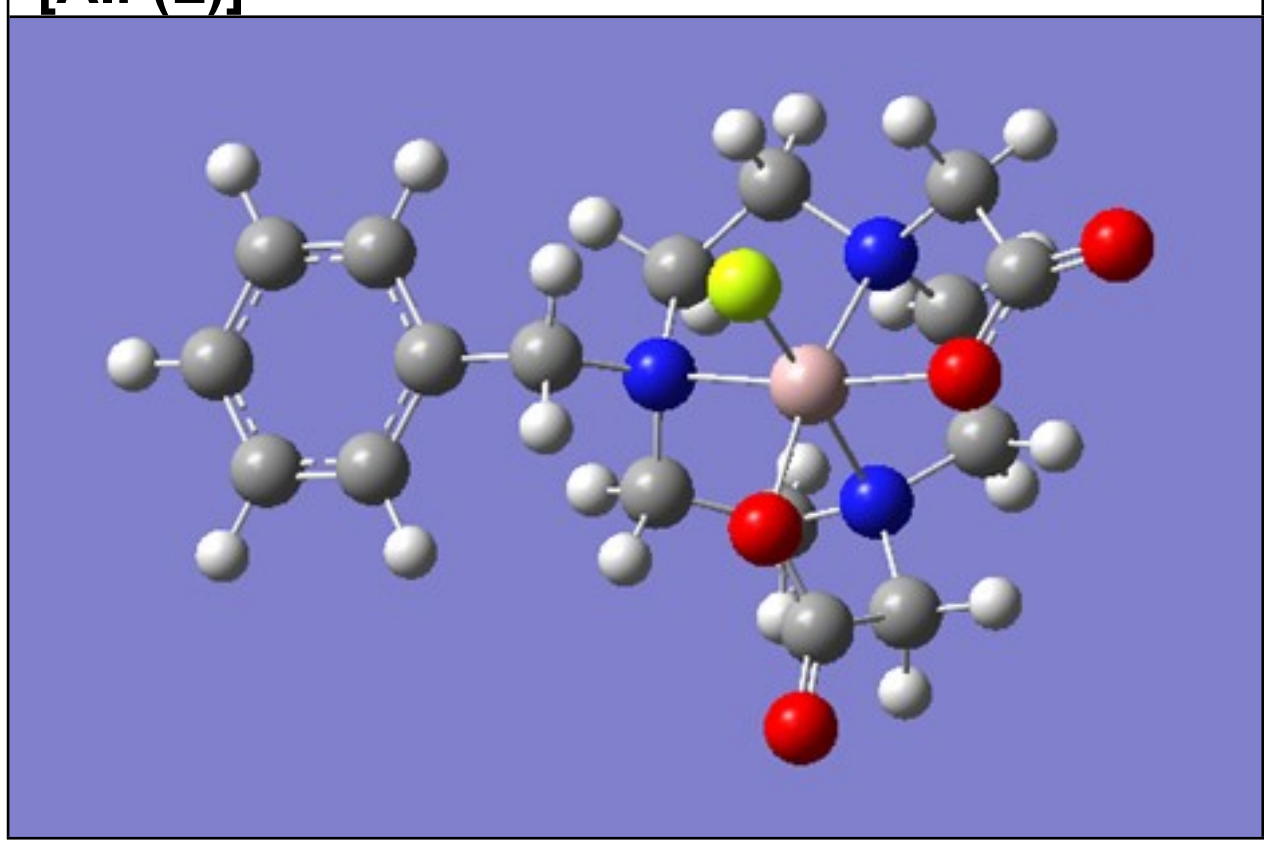	

2d) Solution-phase calculations: Charge Density Table (Mulliken Analysis)

Mulliken charges of [AlCl(L)] + F^- geometries around Al centre. In solution/6-311++G(d,p)

	[AlCl(L)]	RC	TS1	IC1	ICts	IC2	TS2	PC	[AlF(L)]
**Al**	1.19341	1.15765	1.0895	1.44492	1.36315	1.78634	-1.6427	1.51903	1.42251
**Cl**	-0.73261	-0.71959	-0.70132	-0.82914	-0.82283	-0.66705	-0.83247	-0.87156	--*x*--
**F**	--*x*--	-0.86294	-0.72114	-0.48237	-0.44888	-0.47534	-0.34582	-0.62892	-0.63826
**$O_{(2)}$**	-0.49589	-0.50001	-0.50445	-0.47463	-0.40892	-0.49700	-0.30844	-0.63664	-0.63221
**$O_{(7)}$**	-0.46445	-0.44359	-0.24167	-0.36977	-0.38914	-0.52436	-0.47145	-0.63556	-0.63514
**$N_{(4)}$**	-0.14800	-0.15232	-0.20429	-0.11452	-0.03140	-0.02060	0.43388	-0.07101	-0.07760
**$N_{(5)}$**	-0.30794	-0.30919	-0.13947	-0.09476	-0.13589	-0.32508	-0.11566	-0.24320	-0.26659
**$N_{(14)}$**	-0.09577	-0.10312	0.10988	0.18334	0.09605	-0.13533	0.17268	-0.07804	-0.15275

Atom numbers for N atoms and carboxylate atoms around Al vary slightly between geometries, reported values are the equivalent positions, with the carboxylate oxygens being the closest to the metal centre.

3. Supplementary data for [GaCl(L)] + OH¯→ [Ga(OH)(L)] +Cl¯ reaction

3a) Solution-phase calculations: Geometry Tables

Note: Cl/OH-Ga-N$_{(trans)}$ values are taken as the closest to 180º, Ga-N$_{(trans)}$ is the N atom trans to Cl. Ga-O taken as the shortest Ga-O distance from each carboxylate arm.

	**[GaCl(L)]**	**RC**	**TS1**	**IC1**	**ICts**	**IC2**	**TS2**	**PC**	**[Ga(OH)(L)]**
Ga-Cl	2.30639	2.30877	2.27531	2.41669	2.33401	2.42059	4.06389	5.1413	--X--
Ga-OH	--X--	5.55774	2.89601	1.88167	1.90878	1.87257	1.84995	1.89403	1.89525
Ga-O	1.98211<br>2.03987	2.04422<br>1.98062	2.45256<br>2.01447	2.05235<br>3.56209	2.87891<br>2.71868	2.05560<br>3.70196	2.04668<br>2.96828	2.01090<br>2.06462	2.05846<br>2.00667
Ga-N	2.16068<br>2.12532	2.12764<br>2.16435	2.17383<br>2.22279	2.17400<br>2.18718	2.16605<br>2.21683	2.19382<br>2.18213	2.16983<br>2.10330	2.14921<br>2.14001	2.14370<br>2.1412
GaN$_{(trans)}$	2.15449	2.15382	2.14004	2.23786	2.15254	2.23773	2.05334	2.18191	2.17869
N-GaO	163.69865<br>163.97174	164.01543<br>163.62982	150.71184<br>137.33640	160.37288<br>130.14822	145.66703<br>145.26218	159.33087<br>133.80795	161.61392<br>151.61975	163.02918<br>161.29604	161.52024<br>163.08875
Cl-GaOH	--X--	128.77459	88.41496	95.07928	93.97725	92.44593	71.56632	47.90800	--X--
Cl-GaN$_{(trans)}$	177.85099	177.48664	179.67659	170.66424	167.60651	171.37946	157.47855	132.64065	--X--
OH-GaN$_{(trans)}$	--X--	122.94995	154.66012	174.05559	171.36801	176.24155	142.02984	175.30830	175.72329
**Energy**	**-3512.801938**	**-3588.785861**	**-3588.762870**	**-3588.804138**	**-3588.782264**	**-3588.806211**	**-3588.790338**	**-3588.810596**	**-3128.371685**
**Energy G**	**-3512.466085**	**-3588.445135**	**-3588.419700**	**-3588.458133**	**-3588.438249**	**-3588.461495**	**-3588.446539**	**-3588.467754**	**-3128.026033**

[GaCl(L)] + OH^- = -3512.80194 + -75.980355 = **E = -3588.782293** & G = -3588.454278 au

[Ga(OH)(L)] + Cl^- = -3128.371685+ -460.434655 **= E = -3588.806340** & G= -3588.475713 au

3b) Solution-phase calculations: Image of each stationary point

[GaCl(L)]	RC
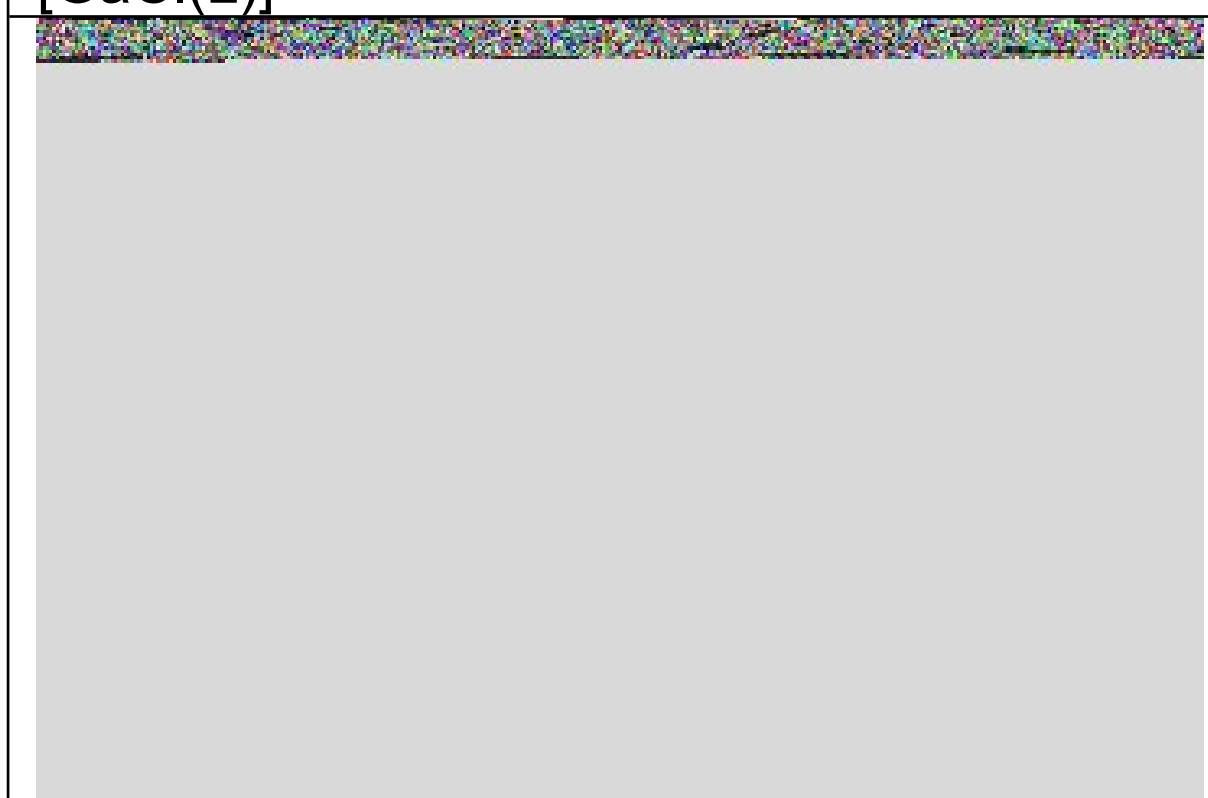	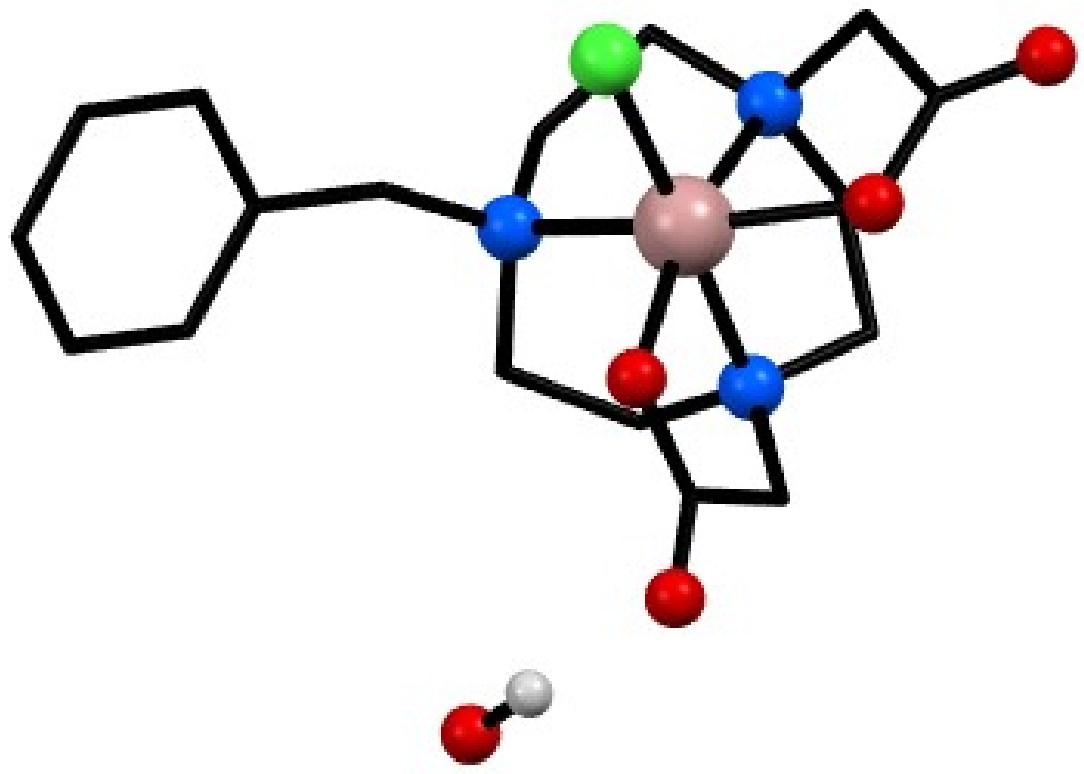
TS1	IC1

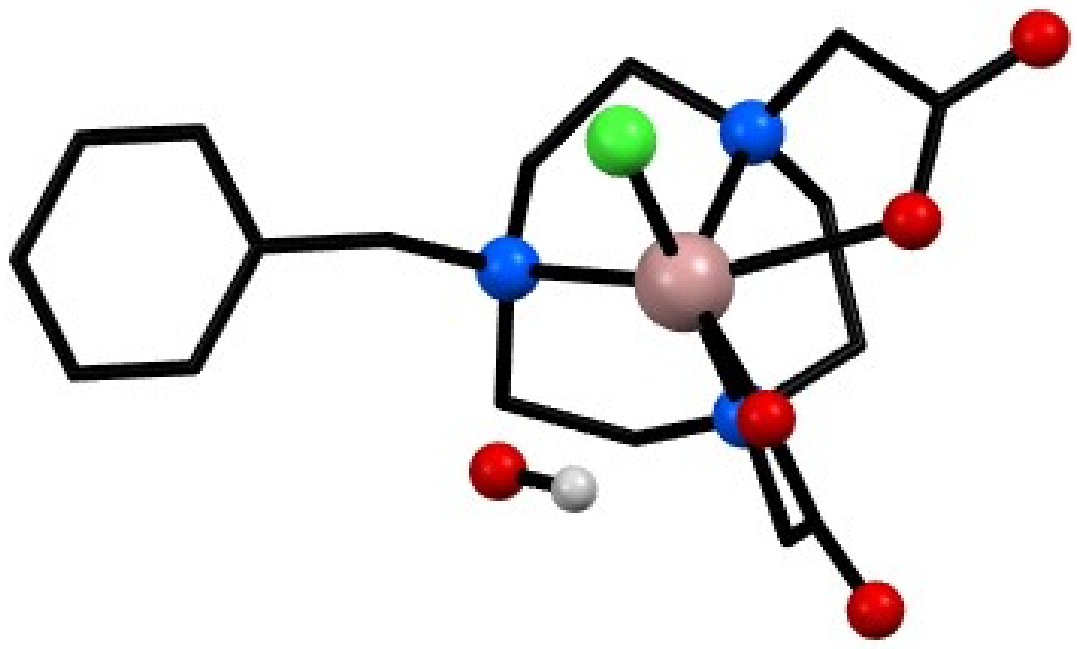	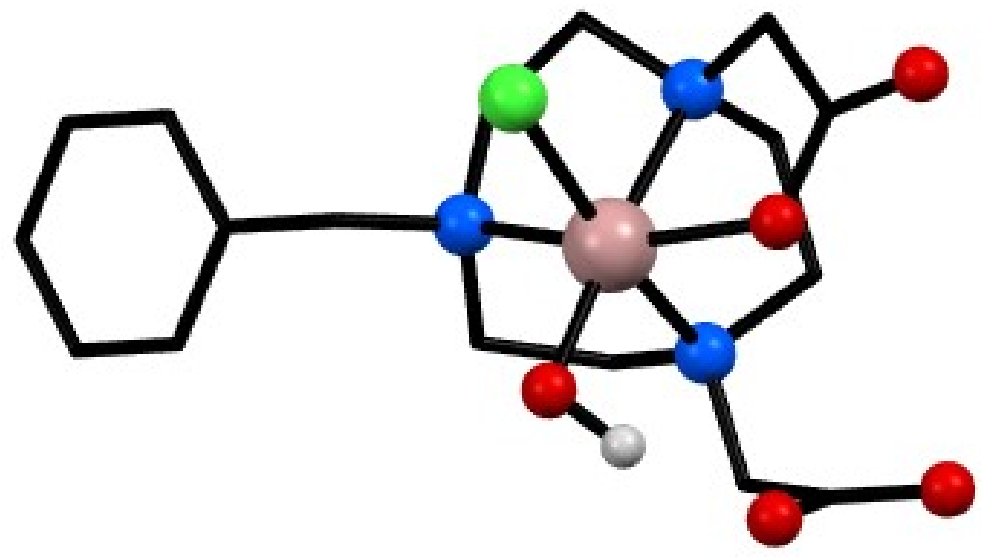
ICts	IC2
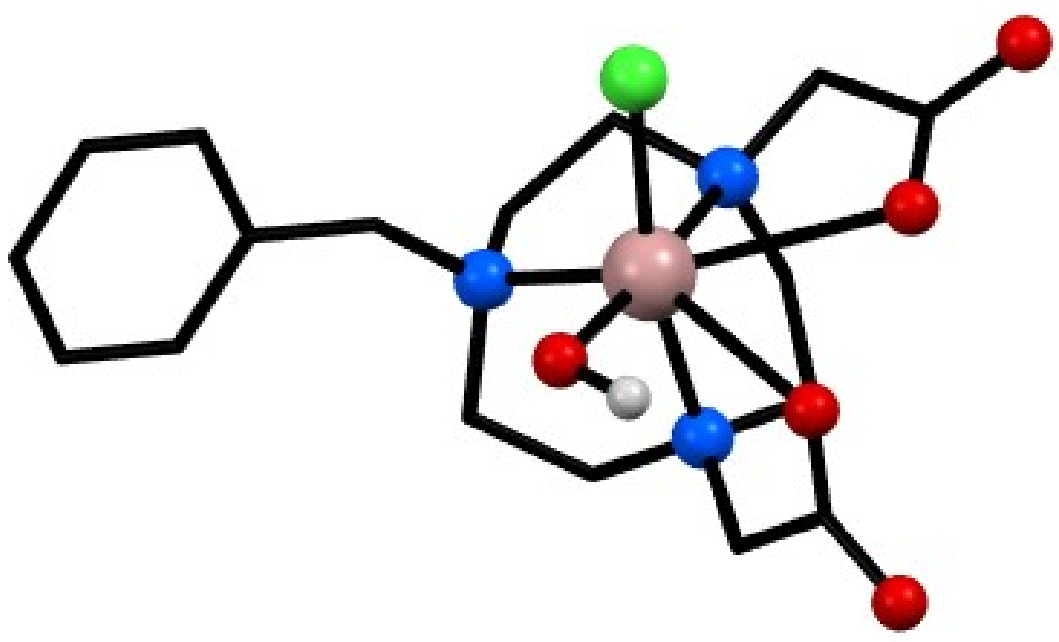	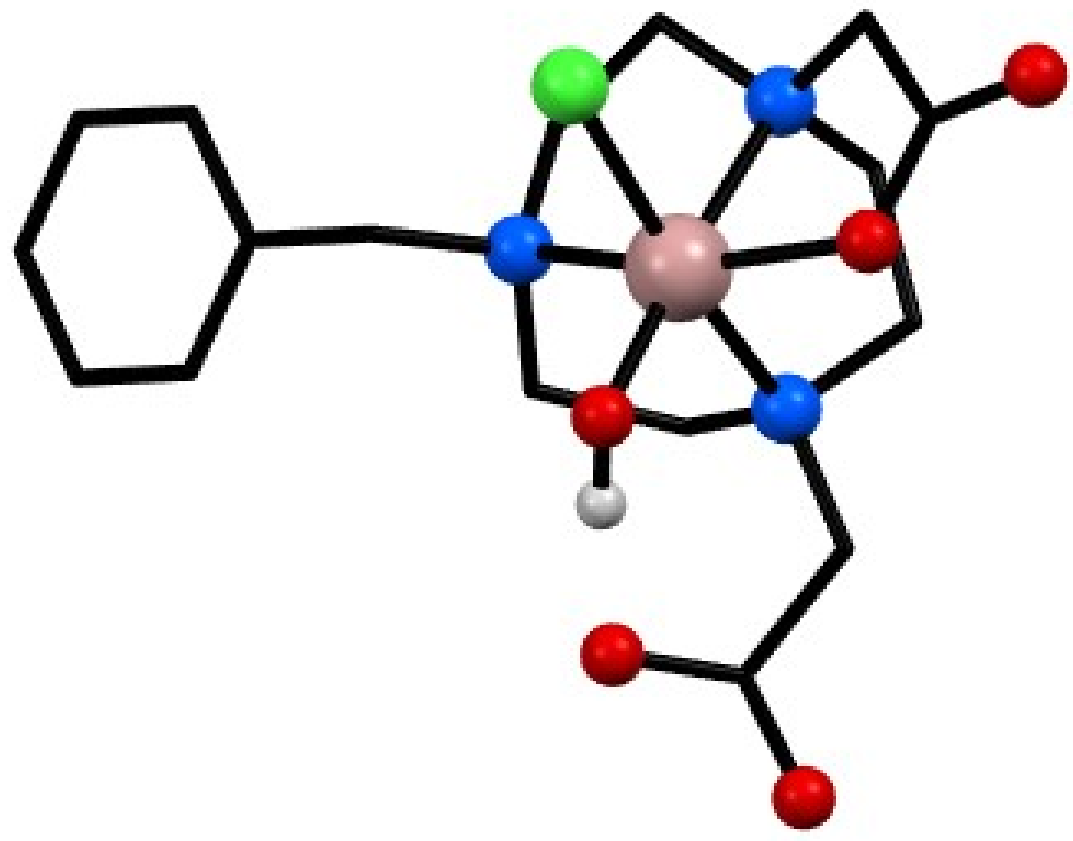
TS2	PC
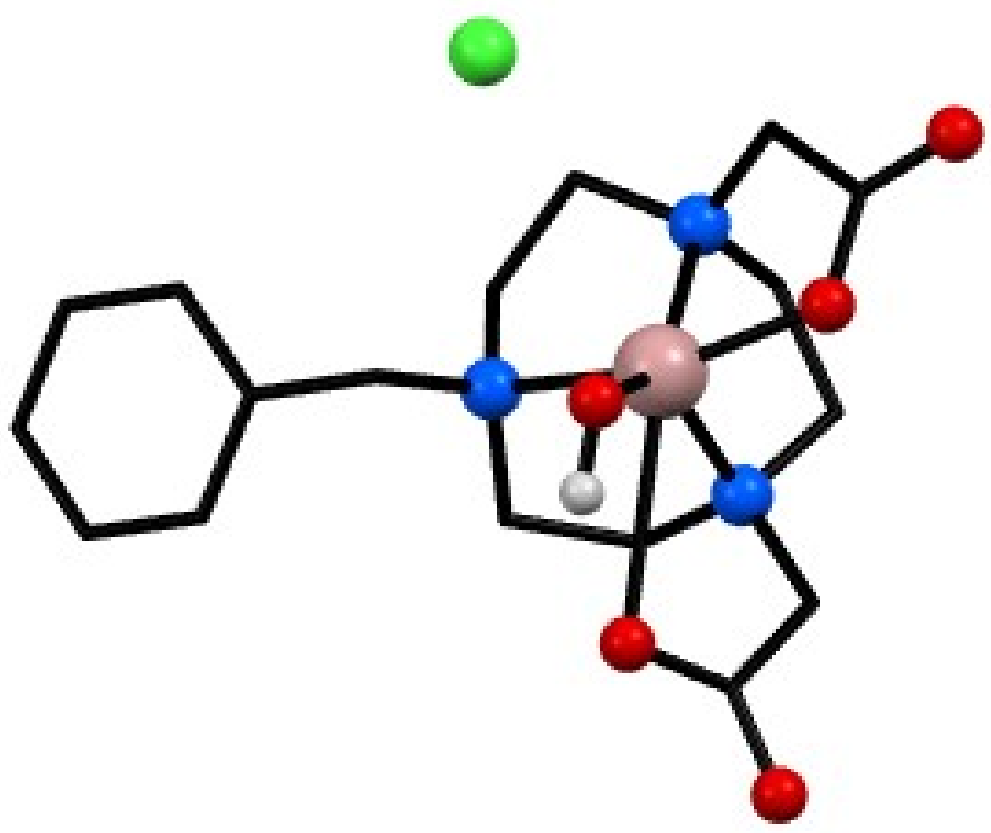	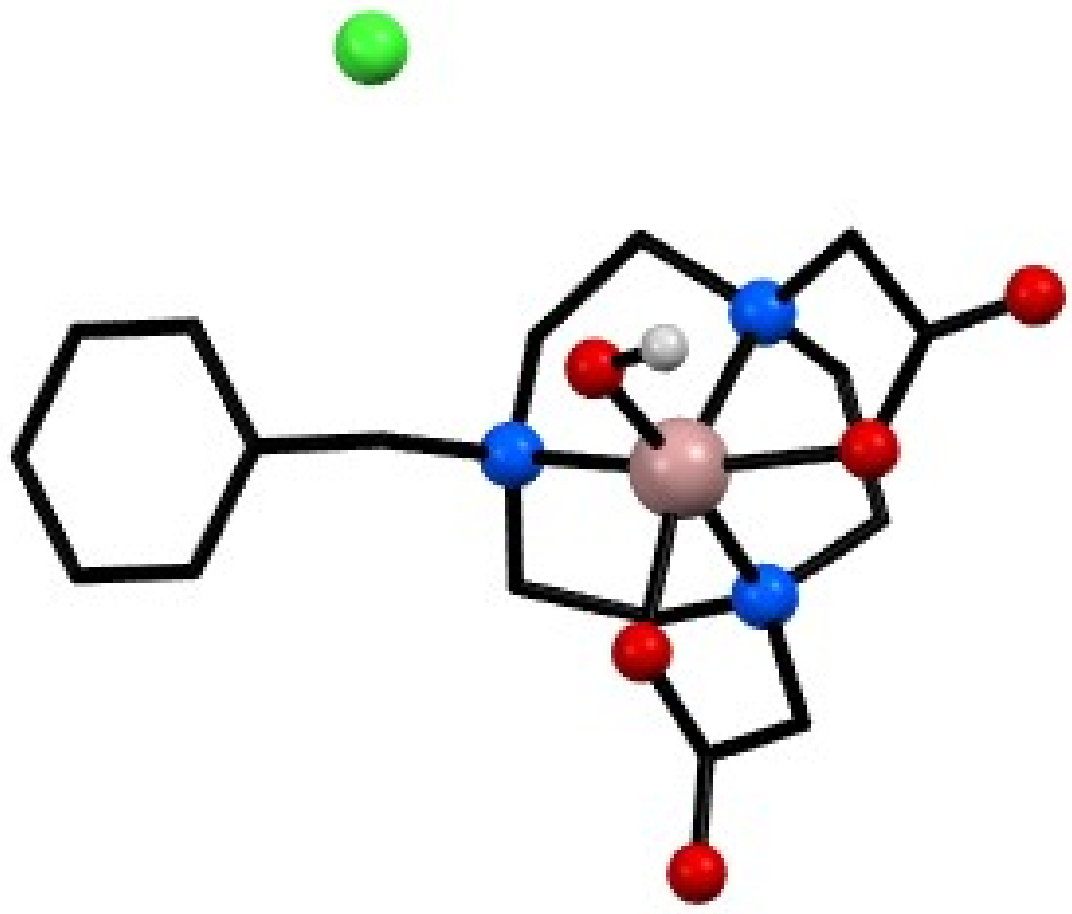
[Ga(OH)(L)]	
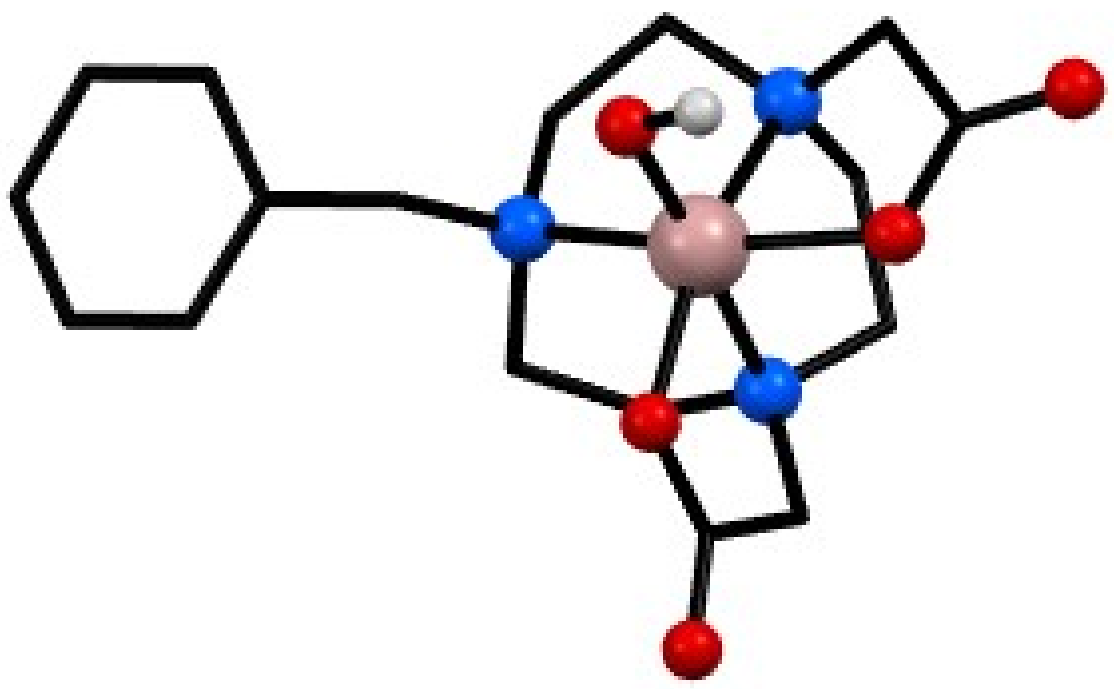	

[GaCl(L)]	RC
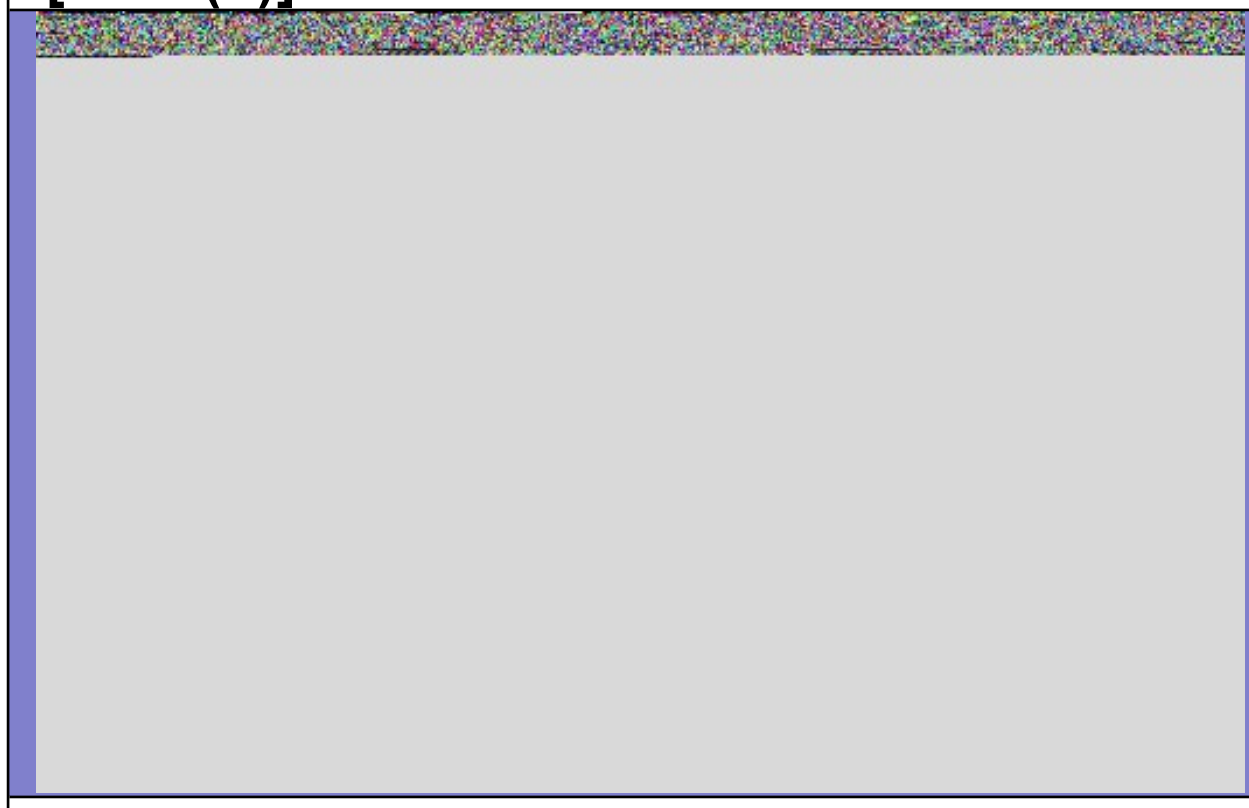	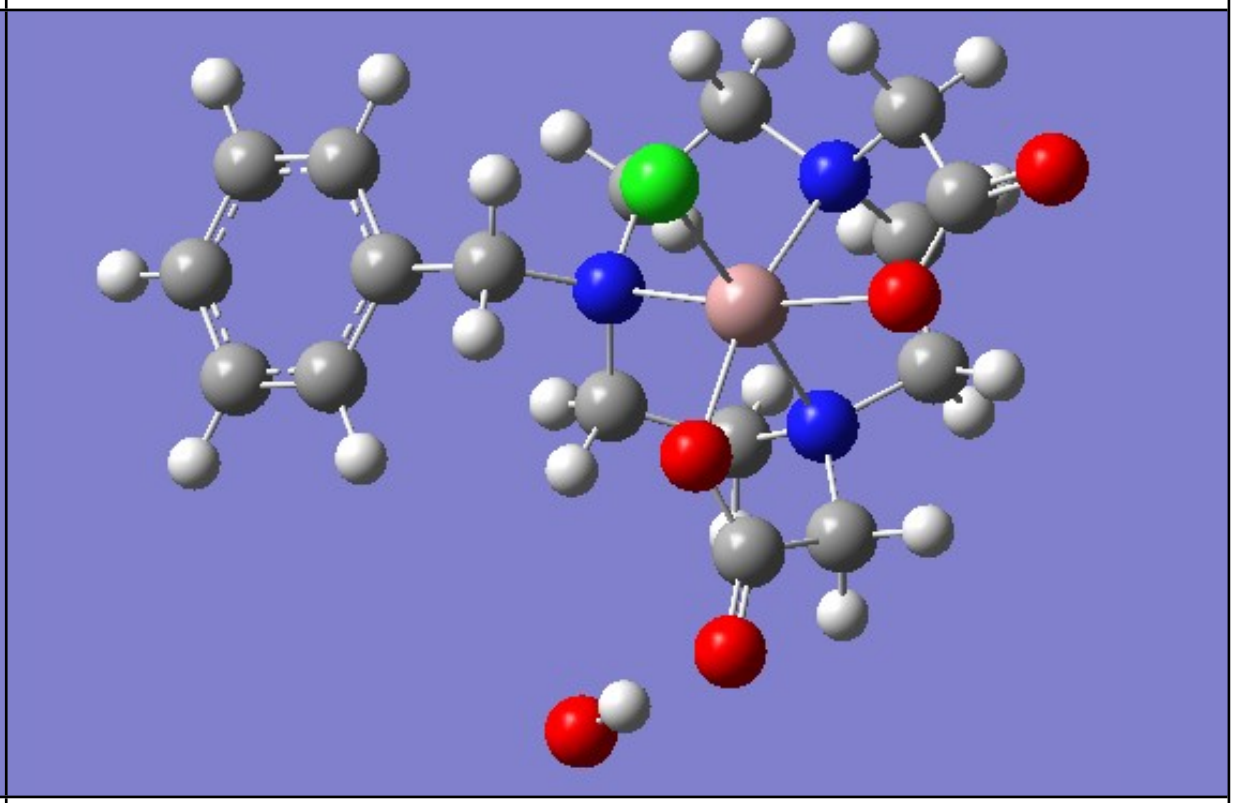
**TS1**	**IC1**
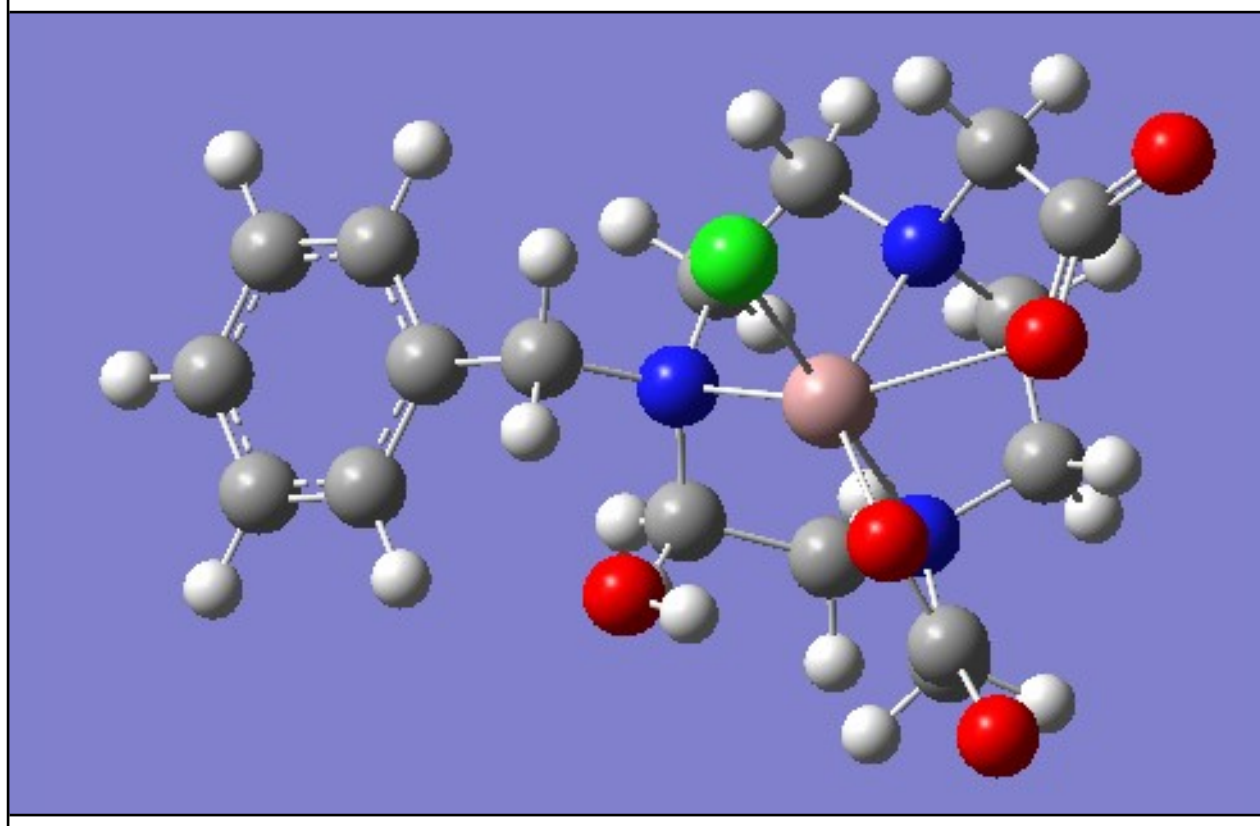	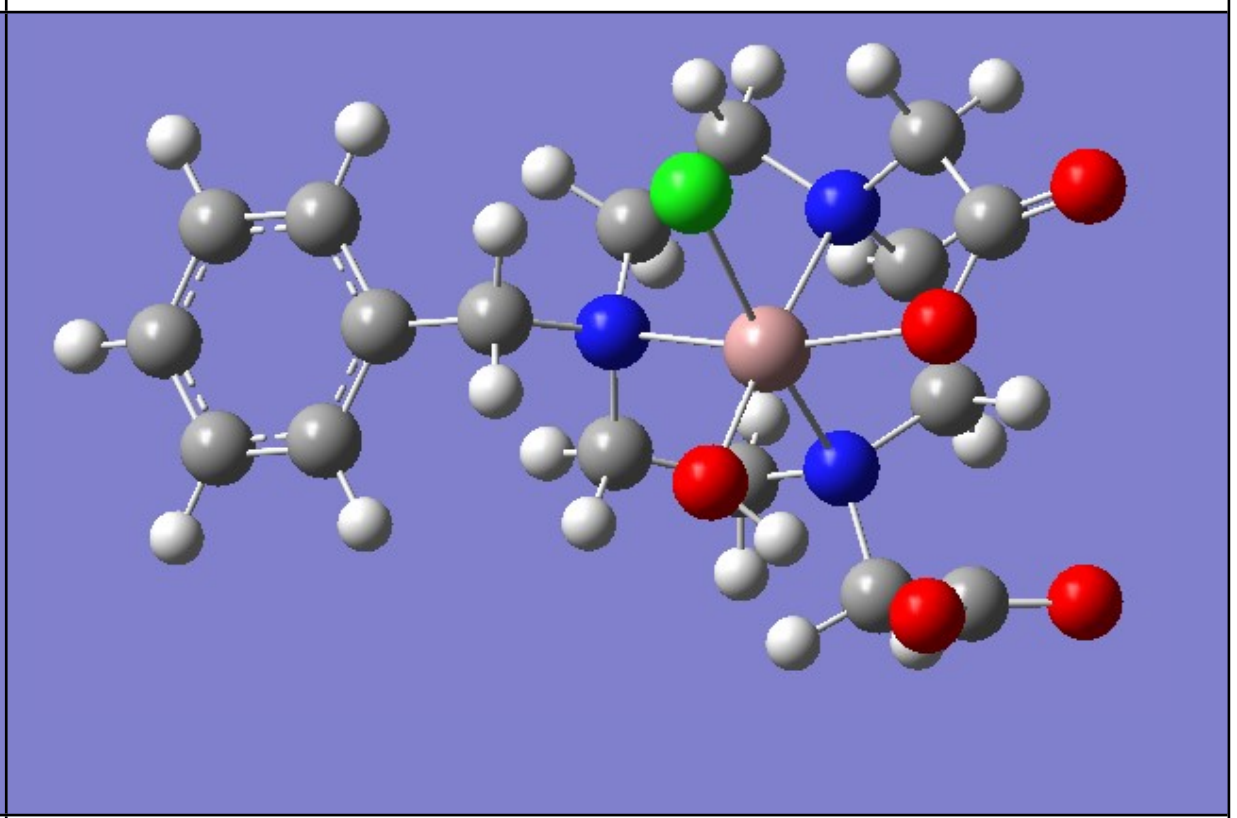
**ICts**	**IC2**
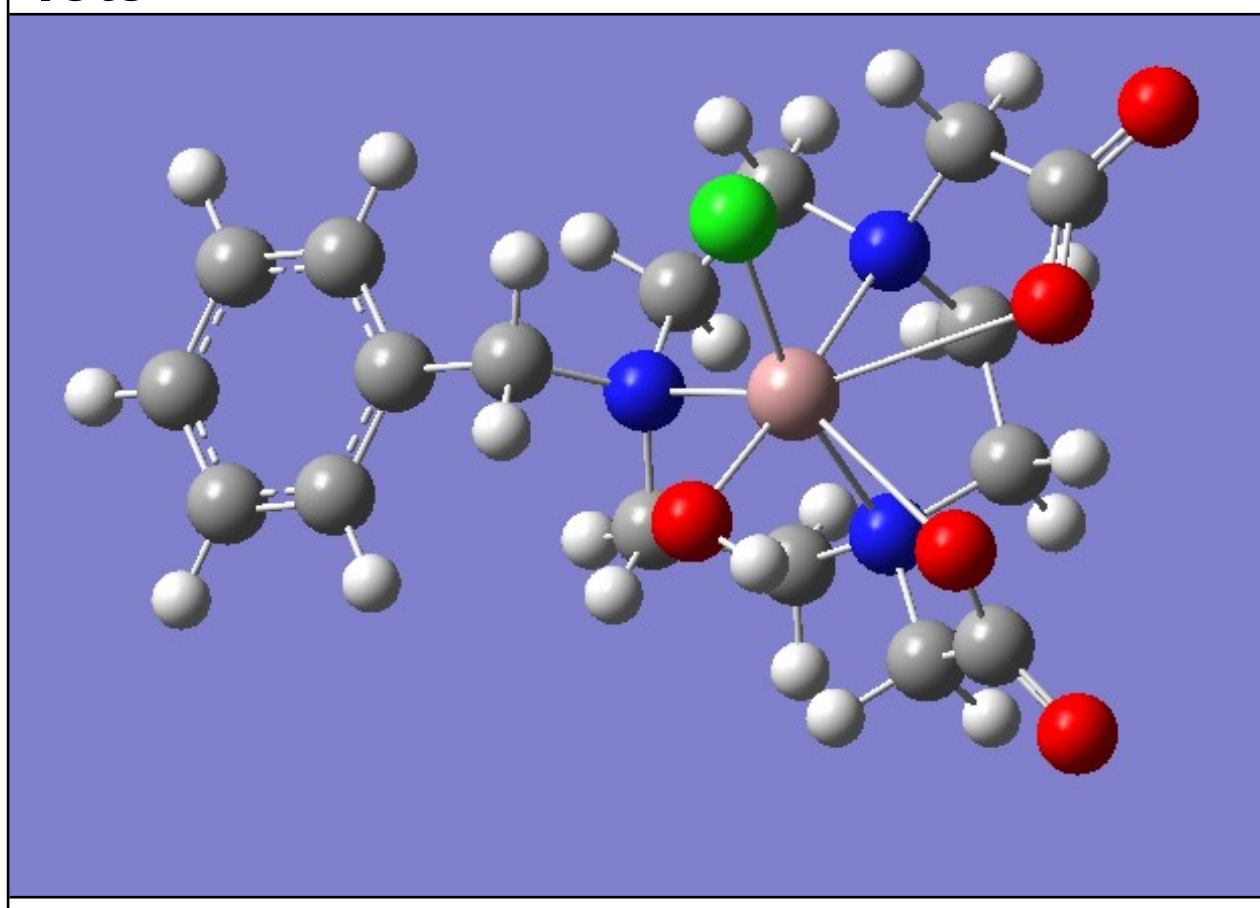	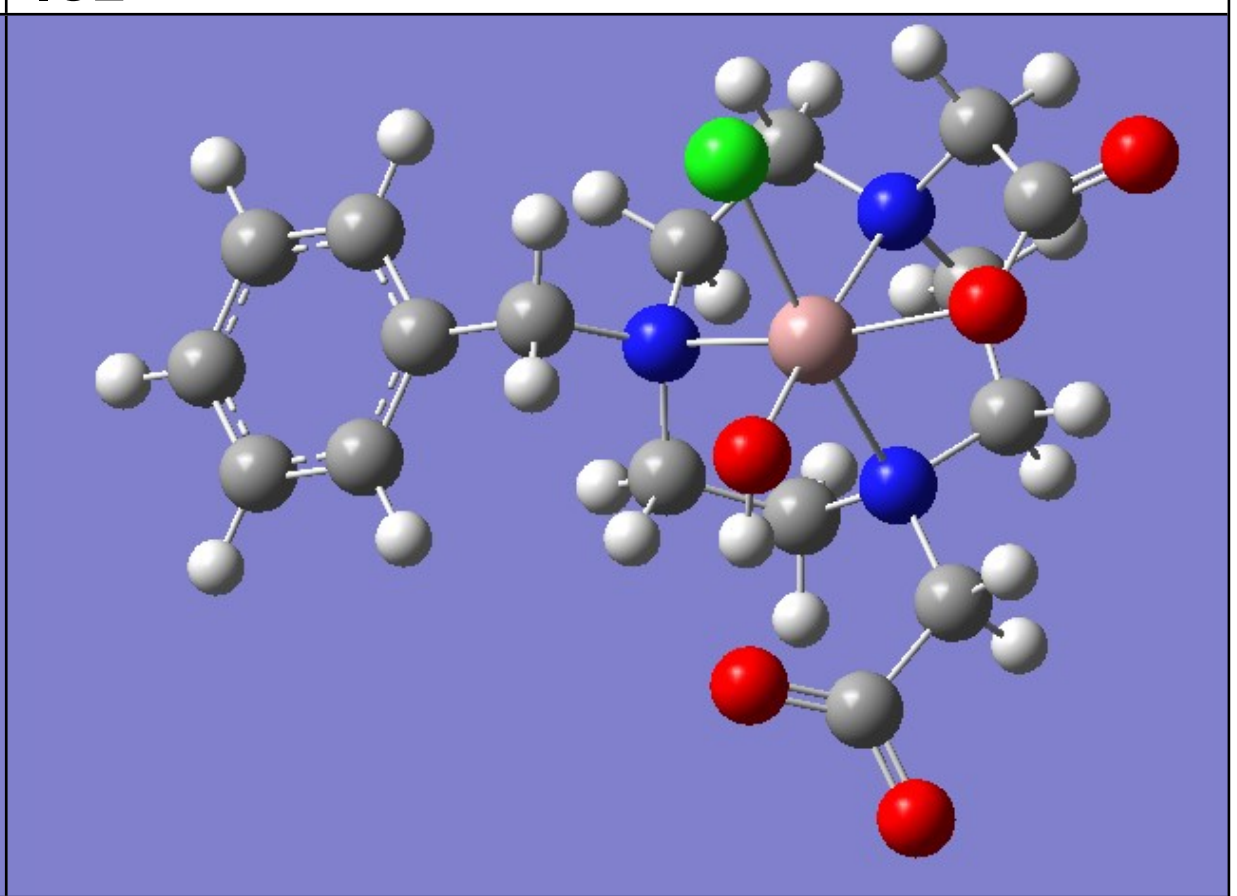
**TS2**	**PC**
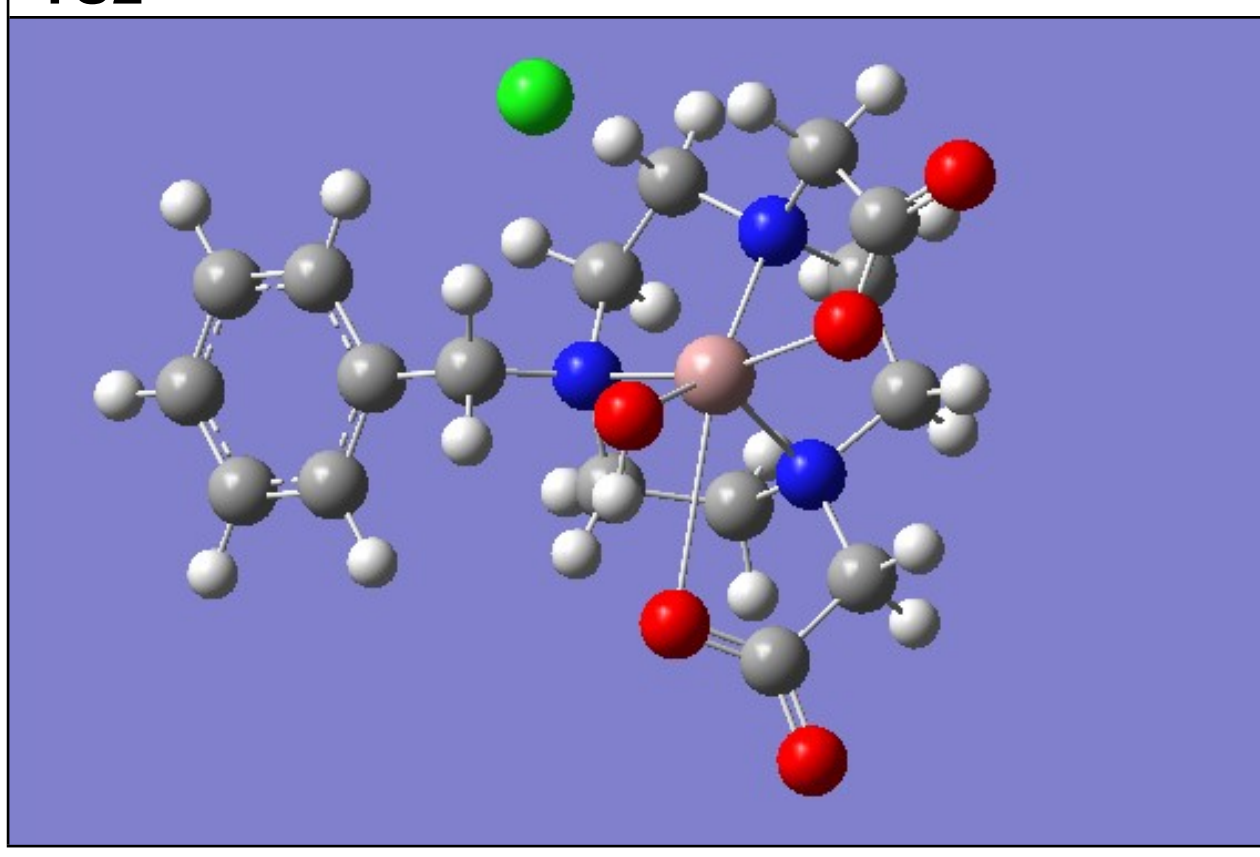	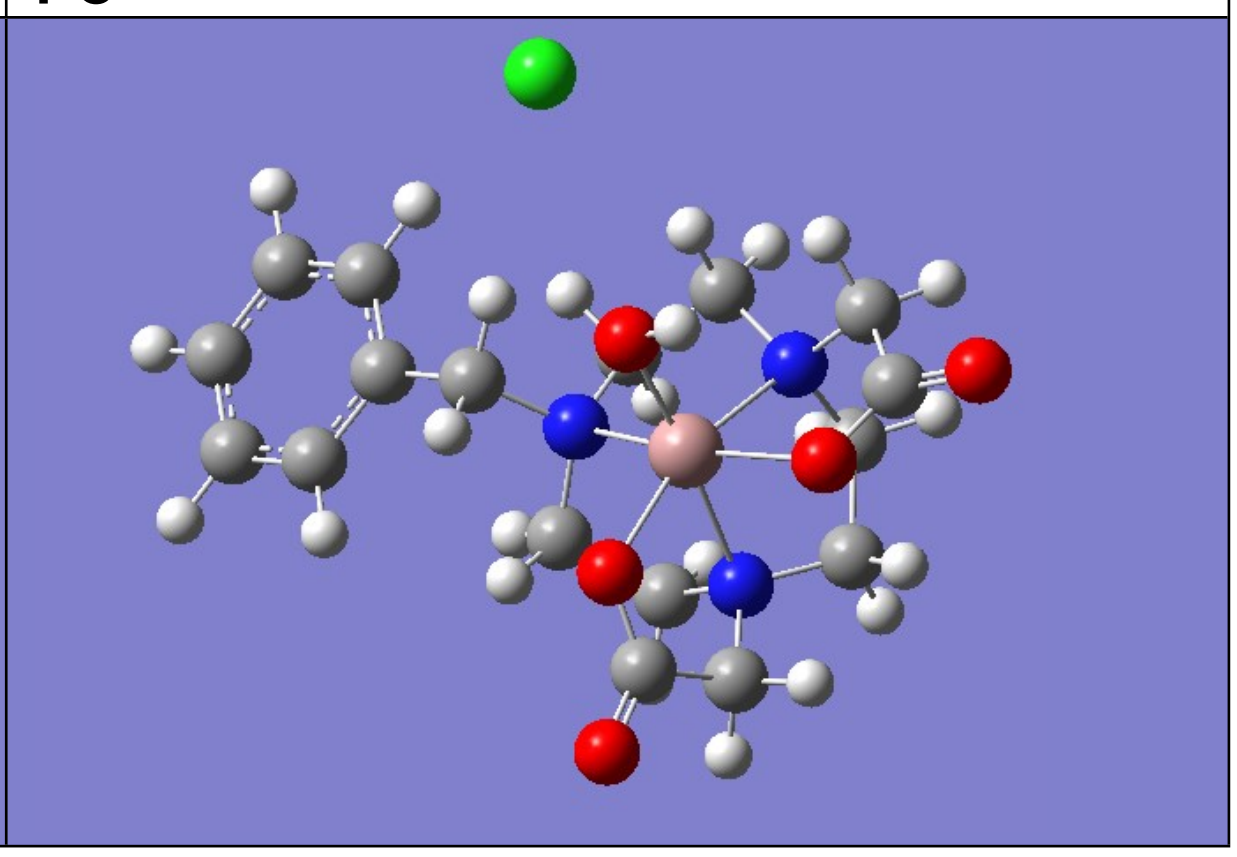

[Ga(OH)(L)]	
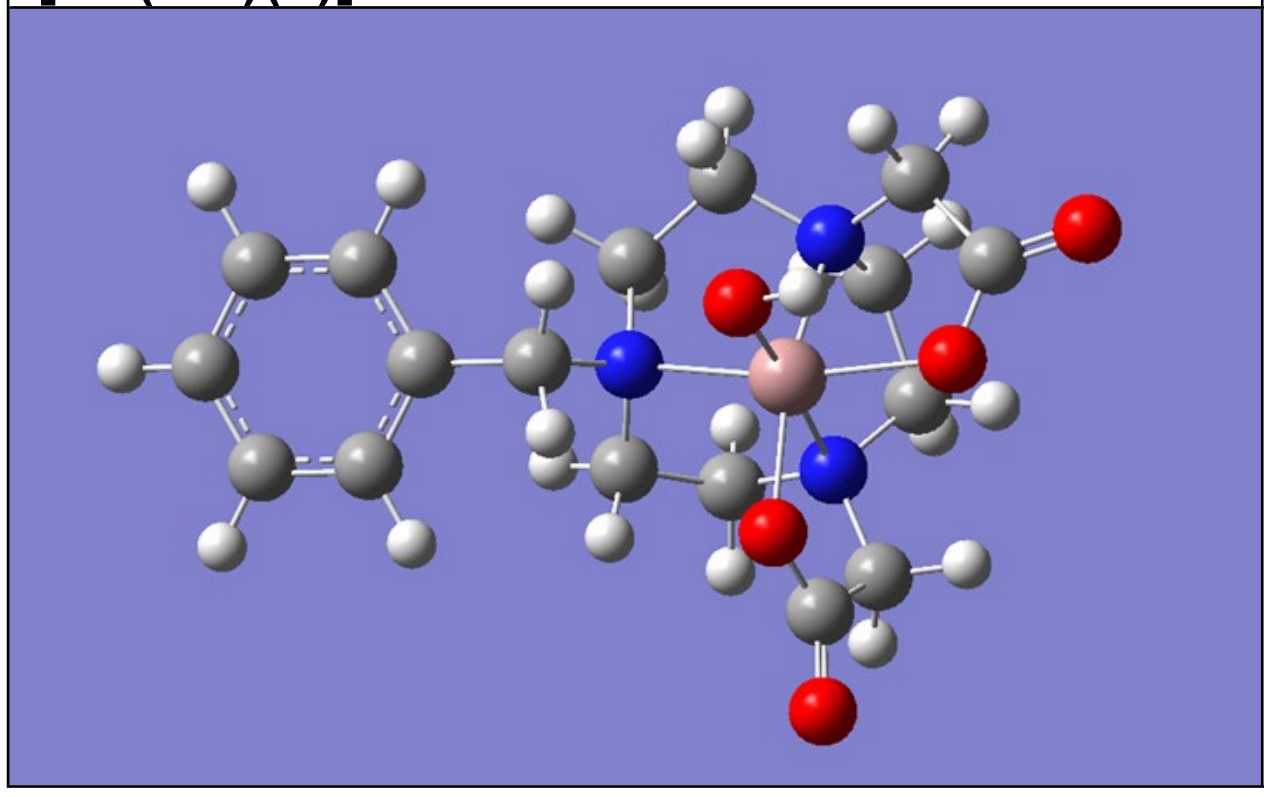	

3c) Solution-phase calculations: Charge Density Table (Mulliken Analysis)

Mulliken charges of [GaCl(L)] + OH^- geometries around Ga centre. In solution/6-311++G(d,p)

	[GaCl(L)]	RC	TS1	IC1	ICts	IC2	TS2	PC	[Ga(OH)(L)]
**Ga**	1.46647	1.488601	1.190715	1.486764	1.253152	1.594911	1.721856	2.118776	2.113961
**Cl**	-0.54546	-0.53474	-0.52368	-0.72291	-0.6297	-0.53069	-0.86474	-0.86325	--X--
**$O_{(OH)}$**	--X--	-1.16489	-1.04419	-0.8492	-0.81917	-0.94326	-0.80828	-0.86325	-0.99077
**$O_{(2)}$**	-0.52157	-0.51712	-0.50677	-0.47651	-0.50852	-0.5321	-0.4621	-0.62043	-0.61784
**$O_{(3)}$**	-0.49942	-0.46472	-0.35022	-0.43663	-0.45117	-0.4994	-0.52048	-0.60904	-0.6222
**$N_{(4)}$**	-0.10288	-0.07186	-0.27689	0.008855	-0.07917	-0.04386	-0.00571	-0.10006	-0.0923
**$N_{(5)}$**	-0.32072	-0.31597	-0.12174	-0.30807	-0.0284	-0.2974	-0.2152	-0.28965	-0.28602
**$N_{(14)}$**	-0.17032	-0.15883	0.138928	-0.22733	0.026524	-0.2356	-0.16907	-0.2234	-0.25973

Atom numbers for N atoms and carboxylate atoms around Ga vary slightly between geometries, reported values are the equivalent positions, with the carboxylate oxygens being the closest to the metal centre.

4. Supplementary data for [AlCl(L)] + OH^{-} → [Al(OH)(L)] + Cl^{-} reaction

4a) Solution-phase calculations: Geometry Tables

Note: Cl/OH-Al-$N_{(trans)}$ values are taken as the closest to 180º, Al-$N_{(trans)}$ is the N atom trans to Cl. Al-O taken as the shortest Al-O distance from each carboxylate arm.

	[AlCl(L)]	RC	TS1	IC1	ICts	IC2	TS2	PC	[Al(OH)(L)]
Al-Cl	2.27408	2.27490	2.24239	2.37591	2.37097	2.37431	4.00451	5.16302	--X--
Al-OH	--X--	5.63882	2.75391	1.80454	1.79228	1.79397	1.77779	1.81606	1.81708
Al-O	1.92511<br>1.88389	1.92723<br>1.88270	2.14053<br>1.94901	1.93011<br>3.59548	1.92733<br>3.87183	1.93433<br>3.72431	1.91929<br>2.91999	1.94701<br>1.90633	1.94241<br>1.90255
Al-N	2.11584<br>2.07135	2.11785<br>2.07492	2.18783<br>2.27780	2.13919<br>2.12279	2.13325<br>2.25730	2.14402<br>2.17216	2.06427<br>2.12152	2.08609<br>2.10583	2.08962<br>2.10834
Al-$N_{(trans)}$	2.09744	2.09834	2.09375	2.17166	2.12733	2.14014	2.01702	2.13088	2.12968
N-Al-O	165.87812<br>166.27117	166.29654<br>165.85533	150.32725<br>142.52256	162.50141<br>129.97777	161.69124<br>138.33473	161.04108<br>134.40829	163.23236<br>152.14740	165.10929<br>163.12957	163.24033<br>165.27897
Cl-Al-OH	--X--	131.25689	88.03942	95.13735	91.36671	92.80218	69.44110	44.74567	--X--
Cl-Al-$N_{(trans)}$	178.55368	178.24599	175.46616	170.72558	171.19043	171.74092	158.08352	133.07643	--X--
OH-Al-$N_{(trans)}$	--X--	121.10960	149.26890	174.68130	176.95892	177.38068	139.67399	176.04309	175.69857
**Energy**	**-1830.249307**	**-1906.233077**	**-1906.199309**	**-1906.250898**	**-1906.237369**	**-1906.252884**	**-1906.230801**	**-1906.266052**	**-1445.827475**
**Energy G**	**-1829.909922**	**-1905.887892**	**-1905.853760**	**-1905.901757**	**-1905.888318**	**-1905.904152**	**-1905.884183**	**-1905.920423**	**-1445.476984**

[AlCl(L)] + OH^{-} = -1830.249307 + -75.980355 = **E = -1906.229662** & G = -1905.898115 au.
[Al(OH)(L)] + Cl^{-} = -1445.827475 + -460.434655 **= E = -1906.26213** & G = -1905.926664 au.

4b) Solution-phase calculations: Image of each stationary point

[AlCl(L)]	RC
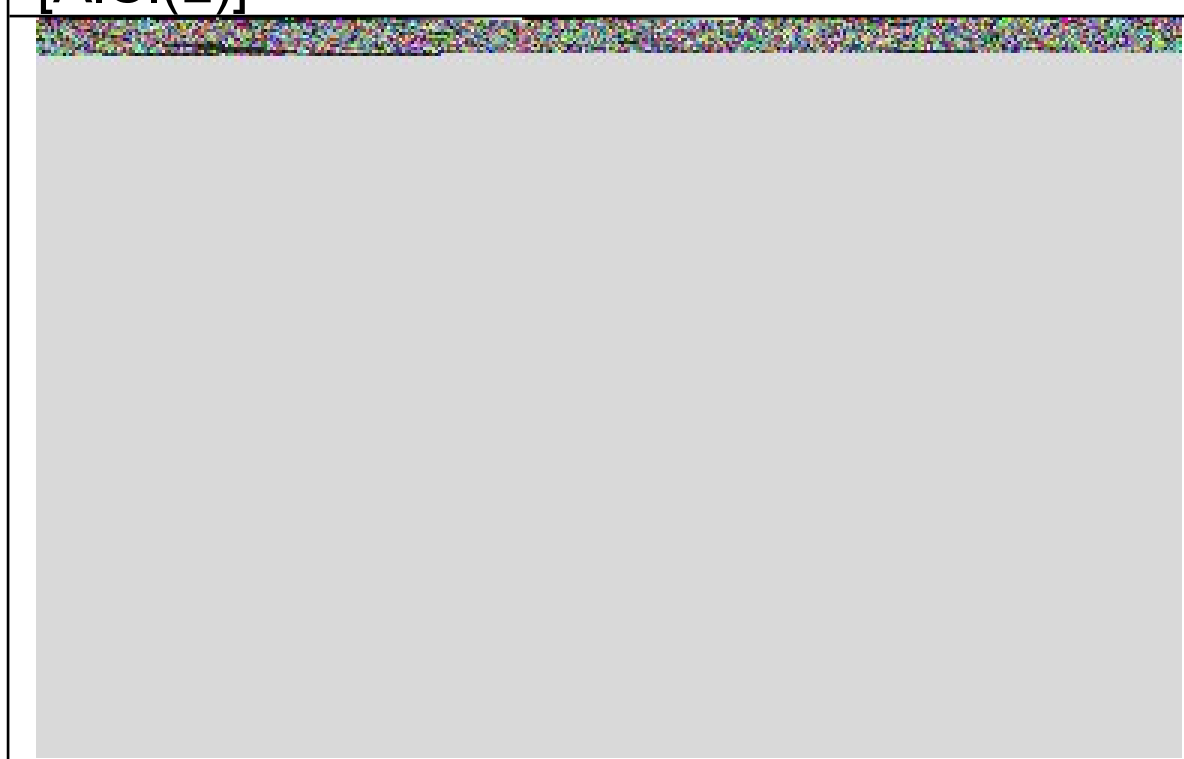	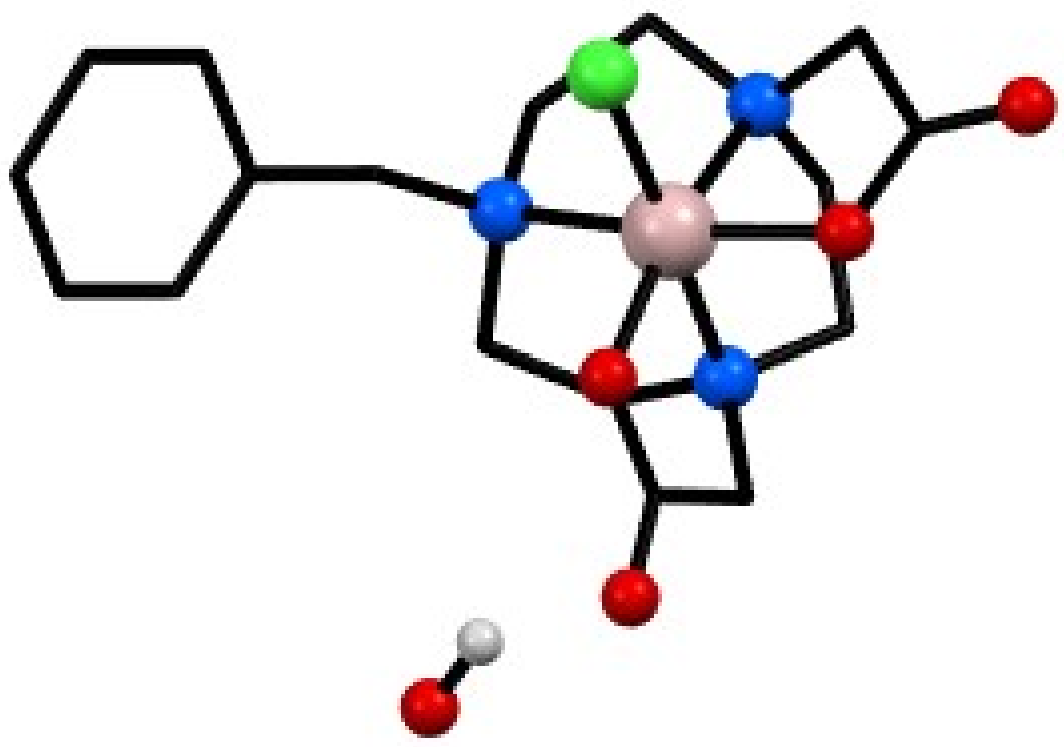
TS1	IC1

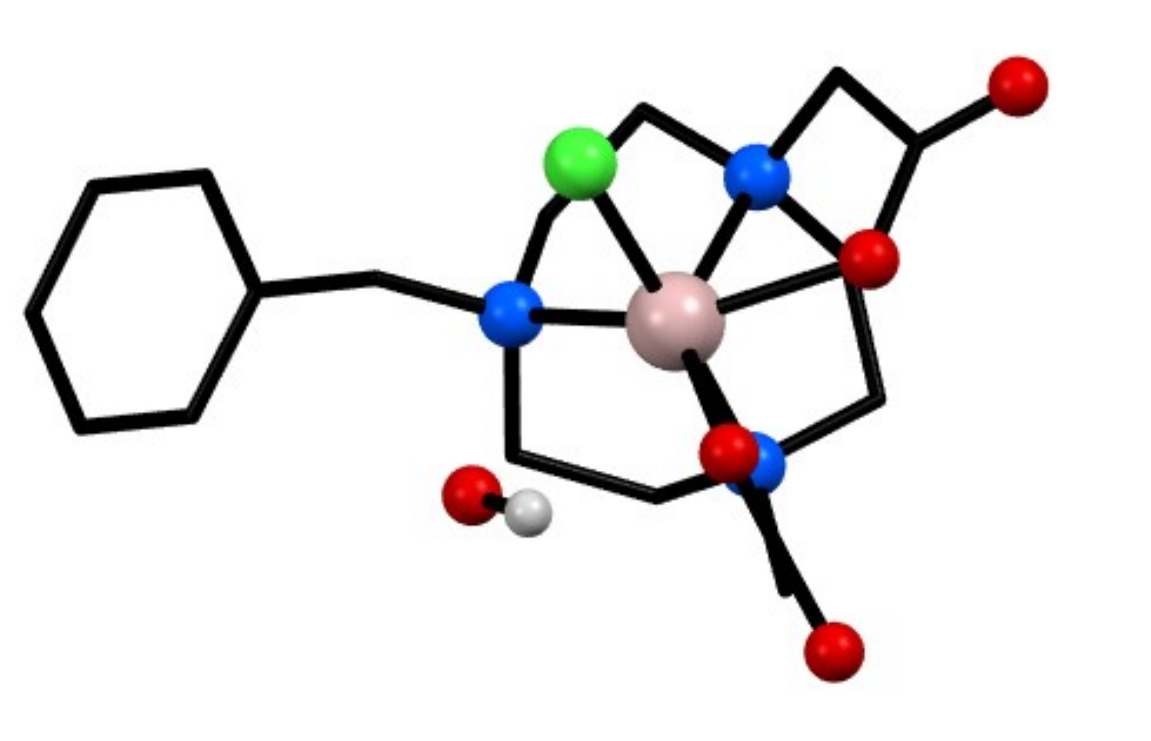	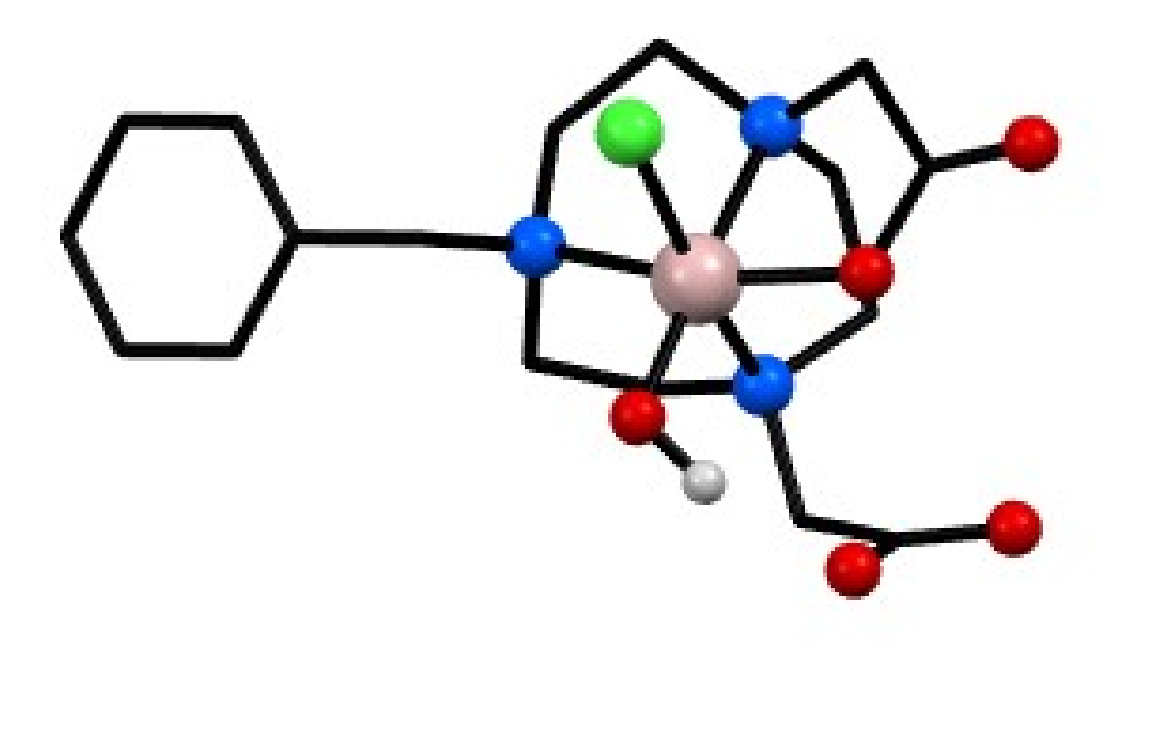
ICts	IC2
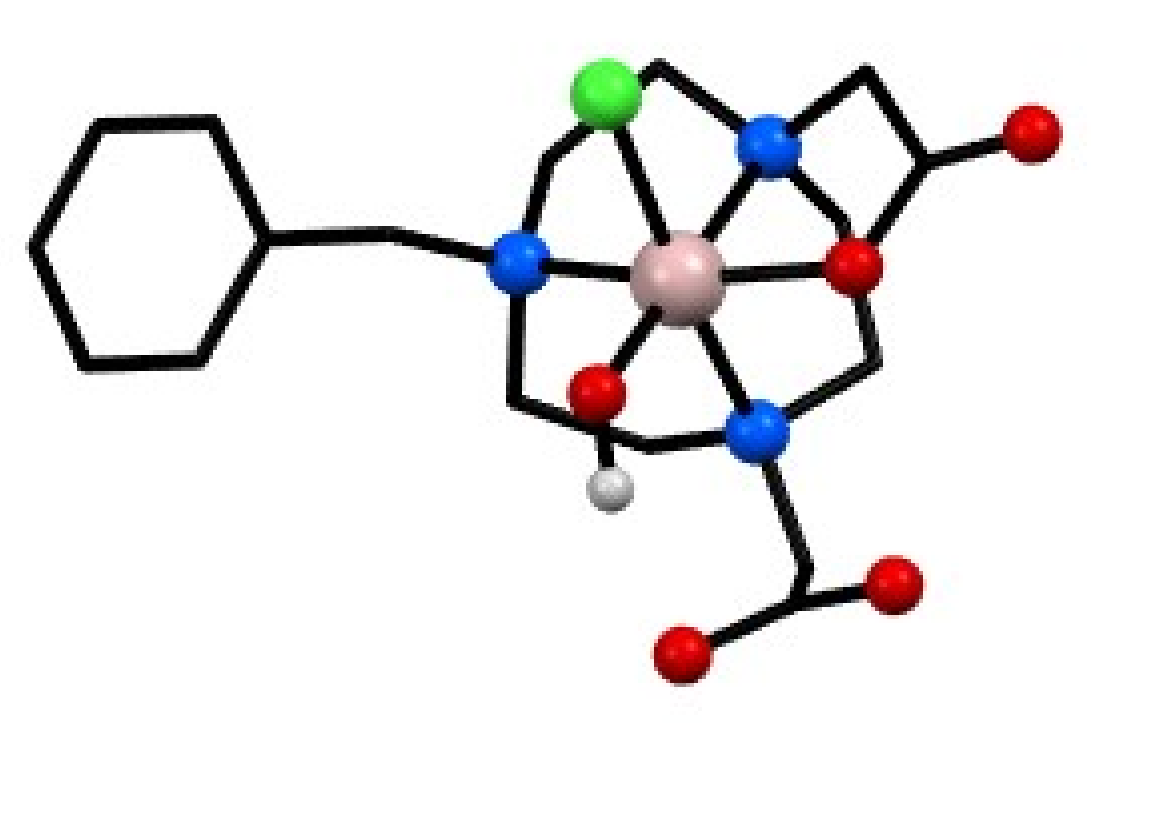	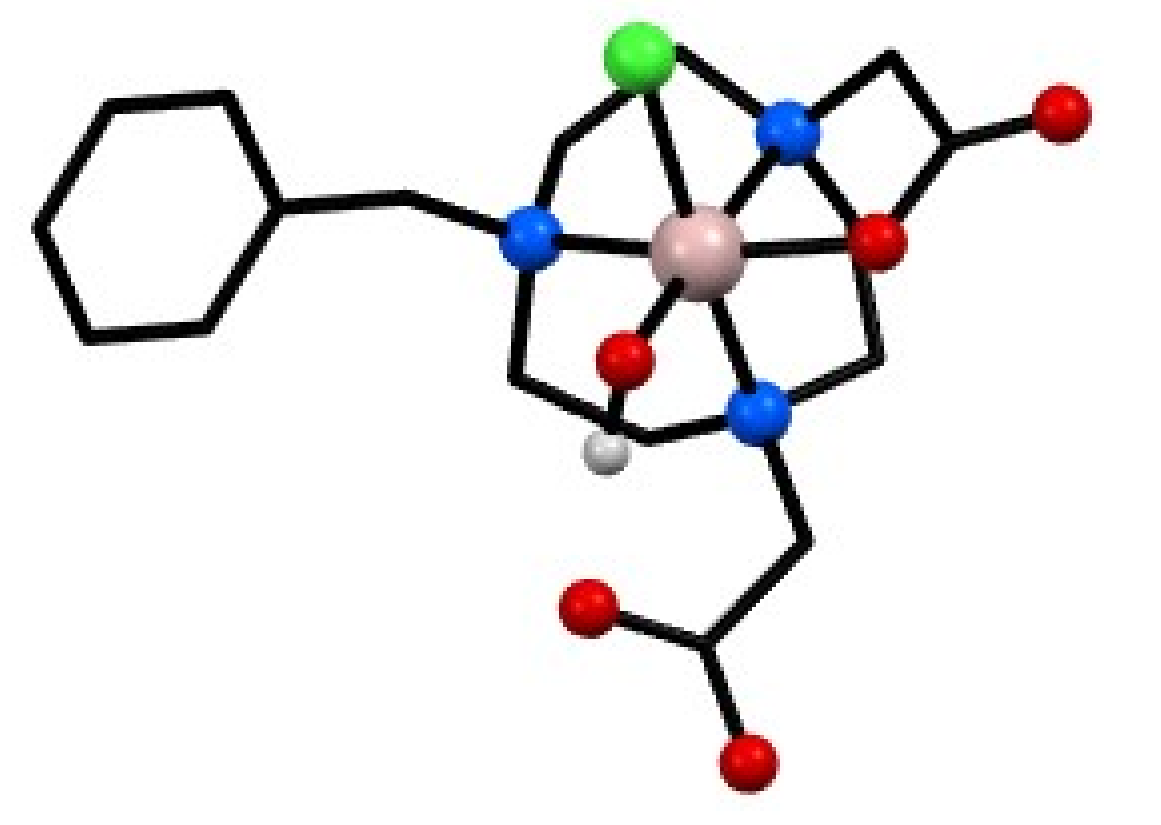
TS2	PC
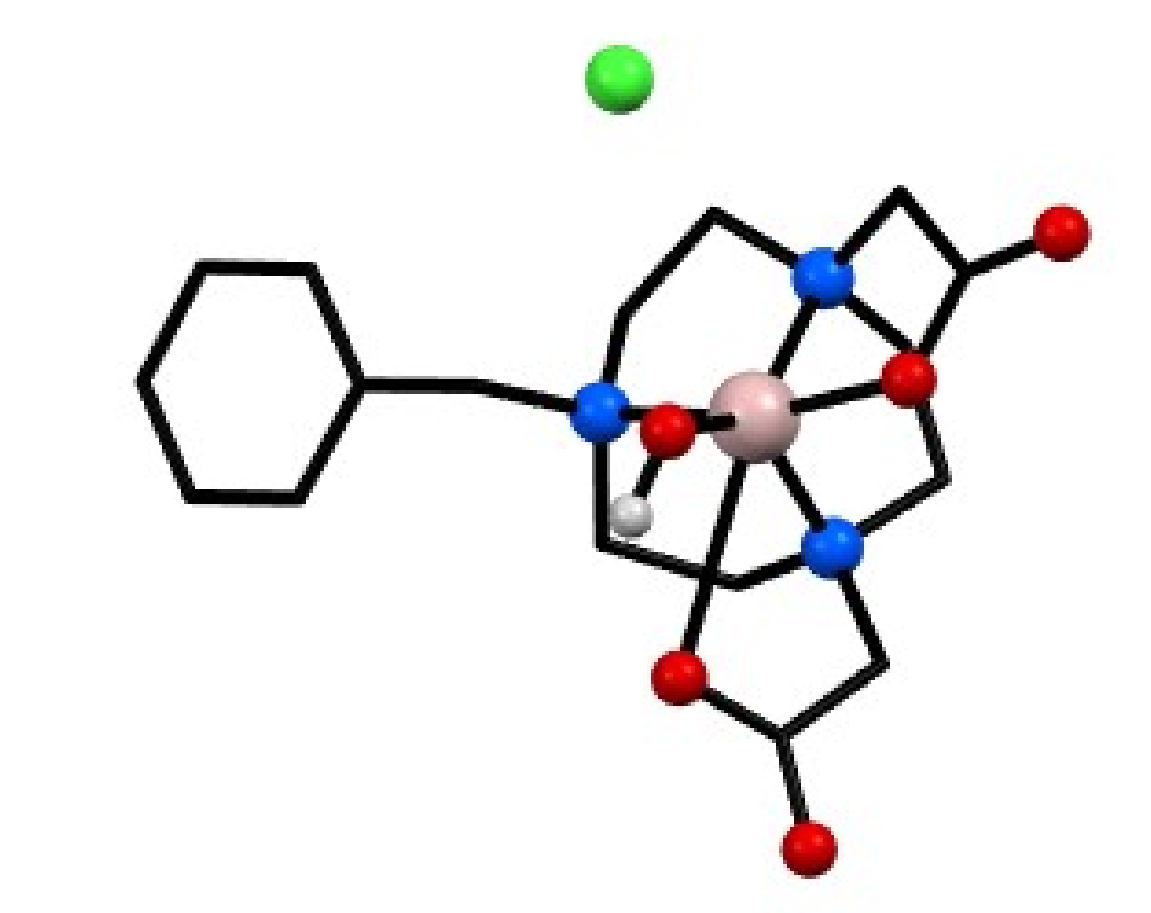	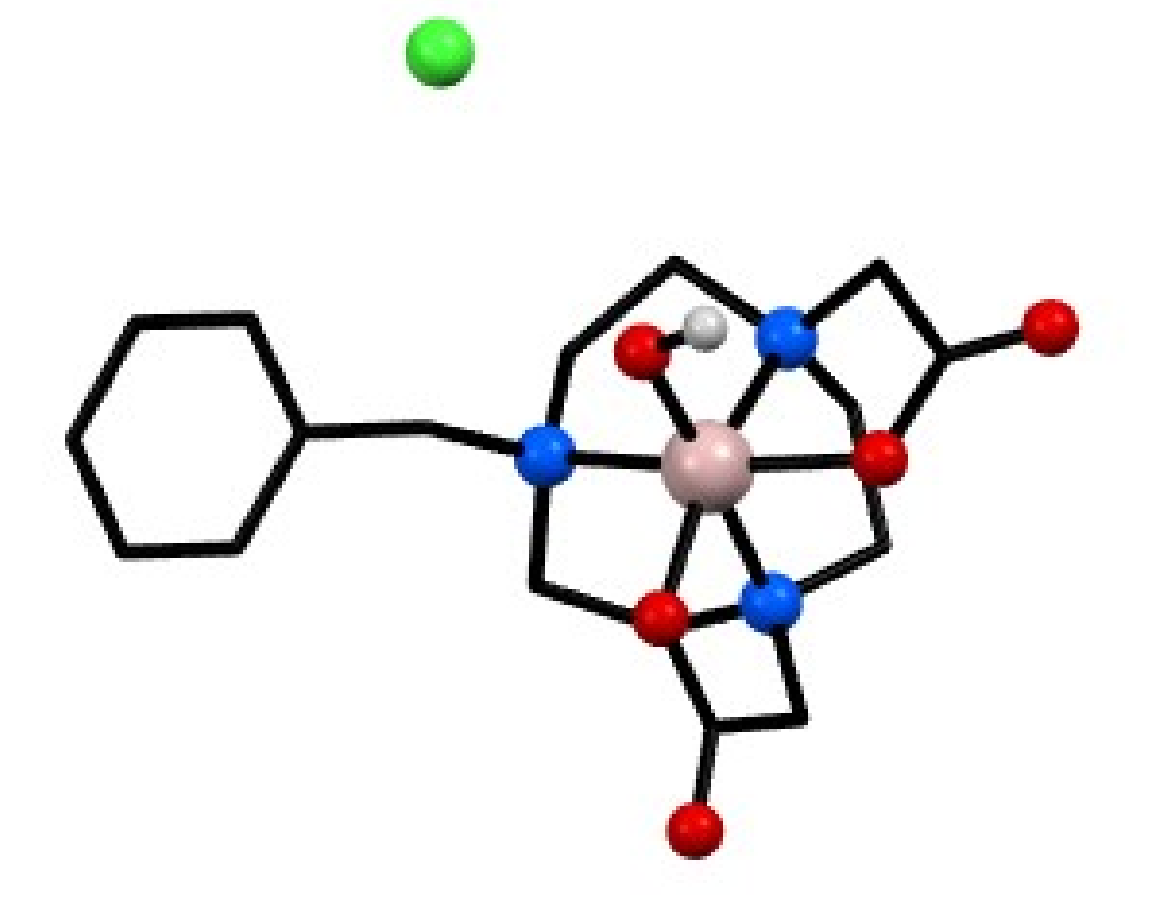
[Al(OH)(L)]	
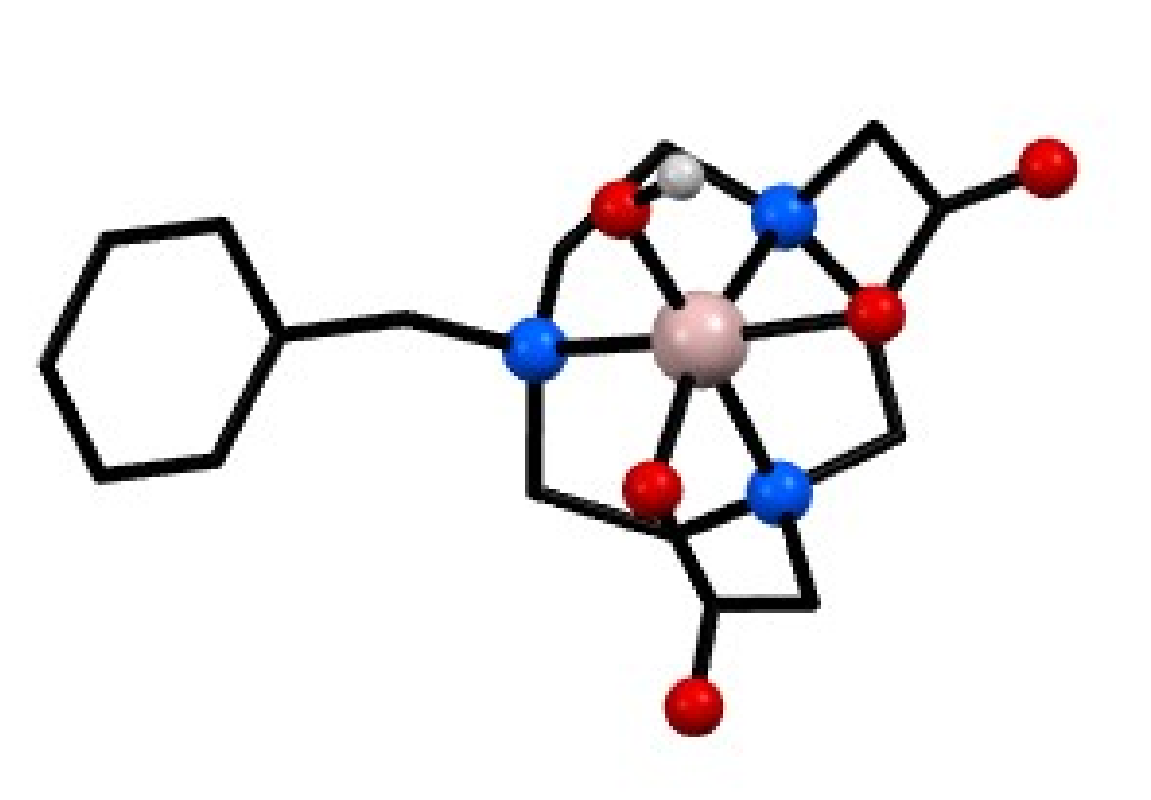	

**[AlCl(L)]**	**RC**
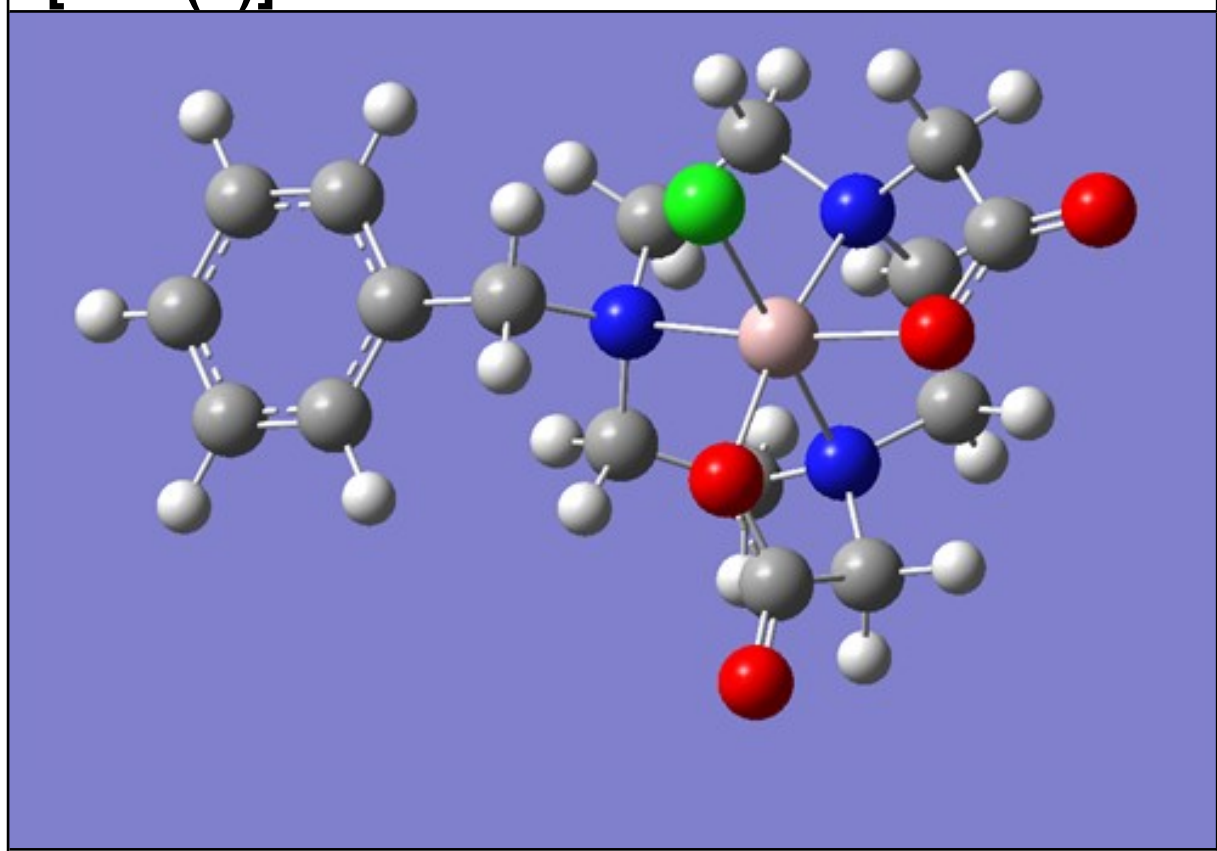	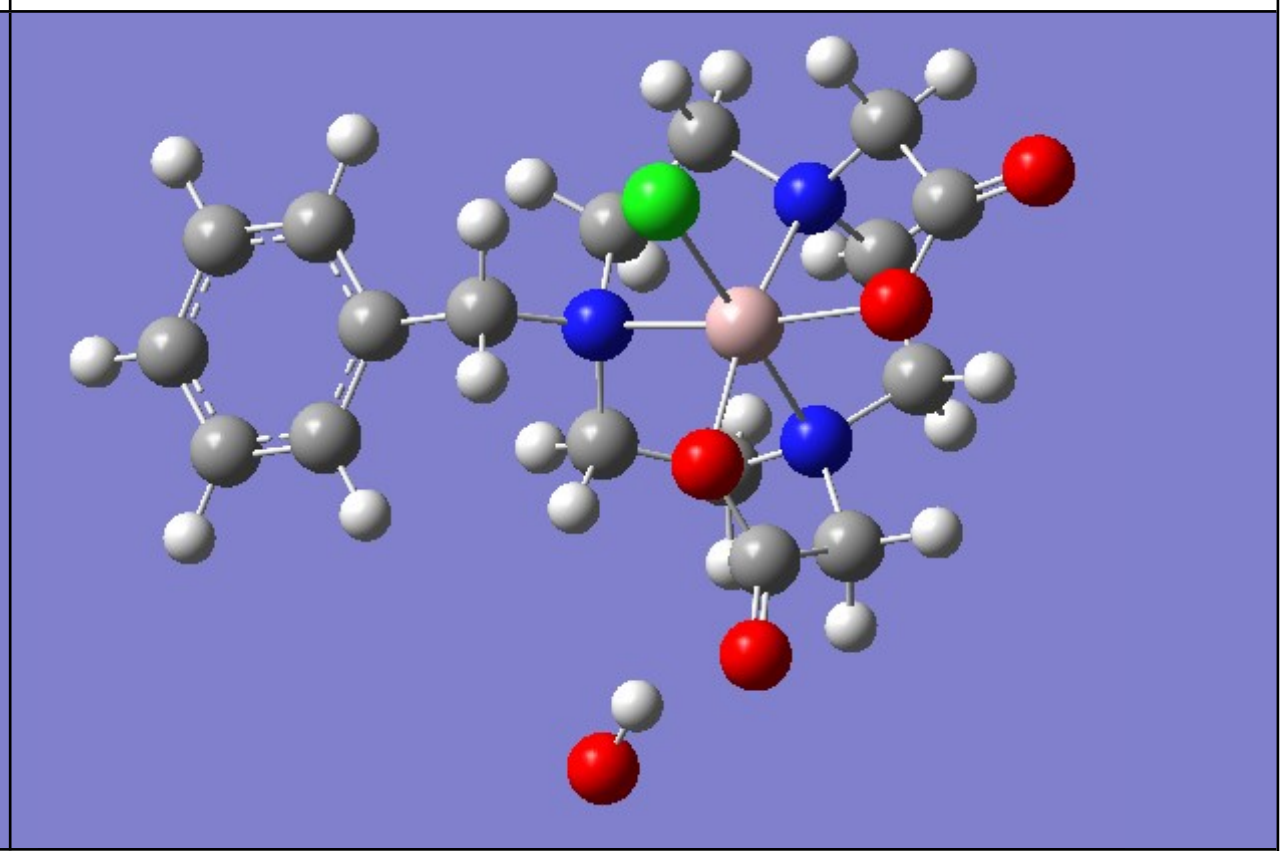
**TS1**	**IC1**
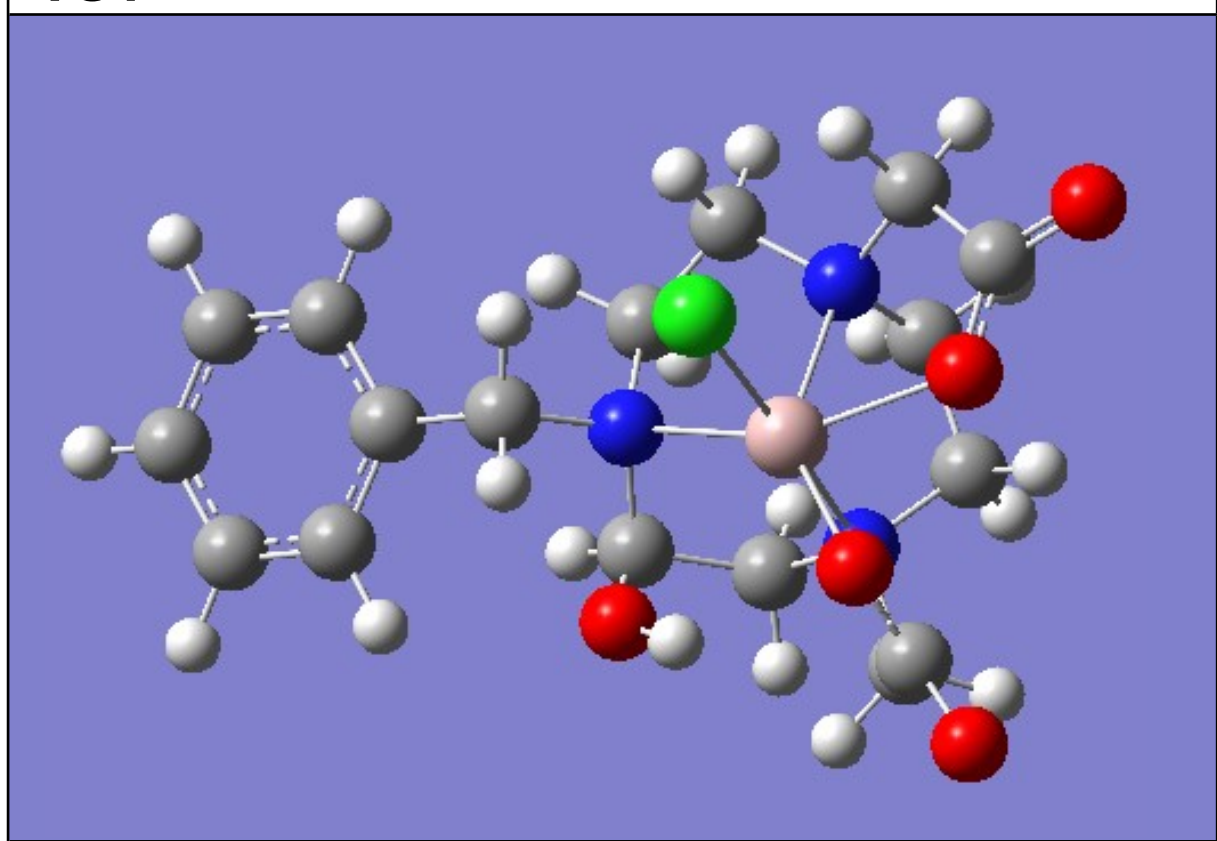	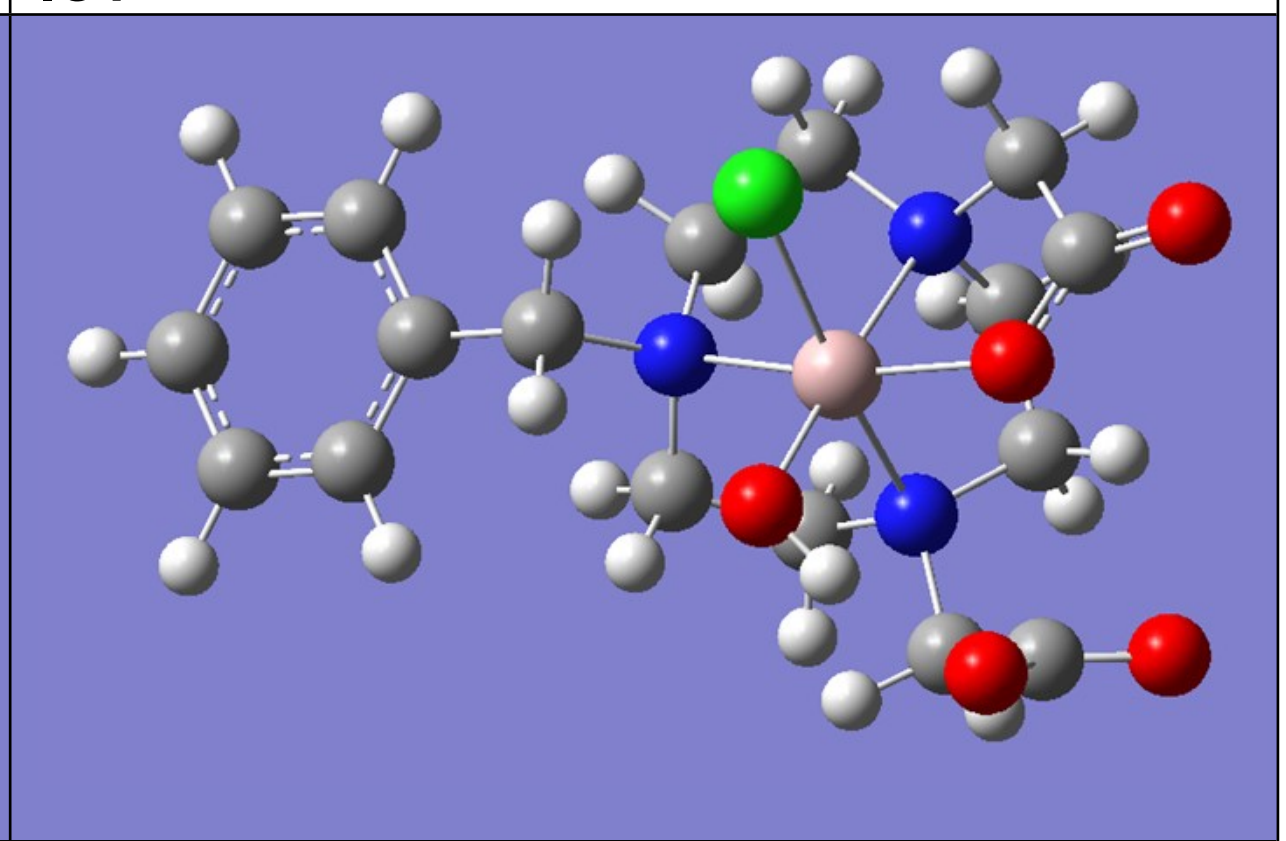
**ICts**	**IC2**
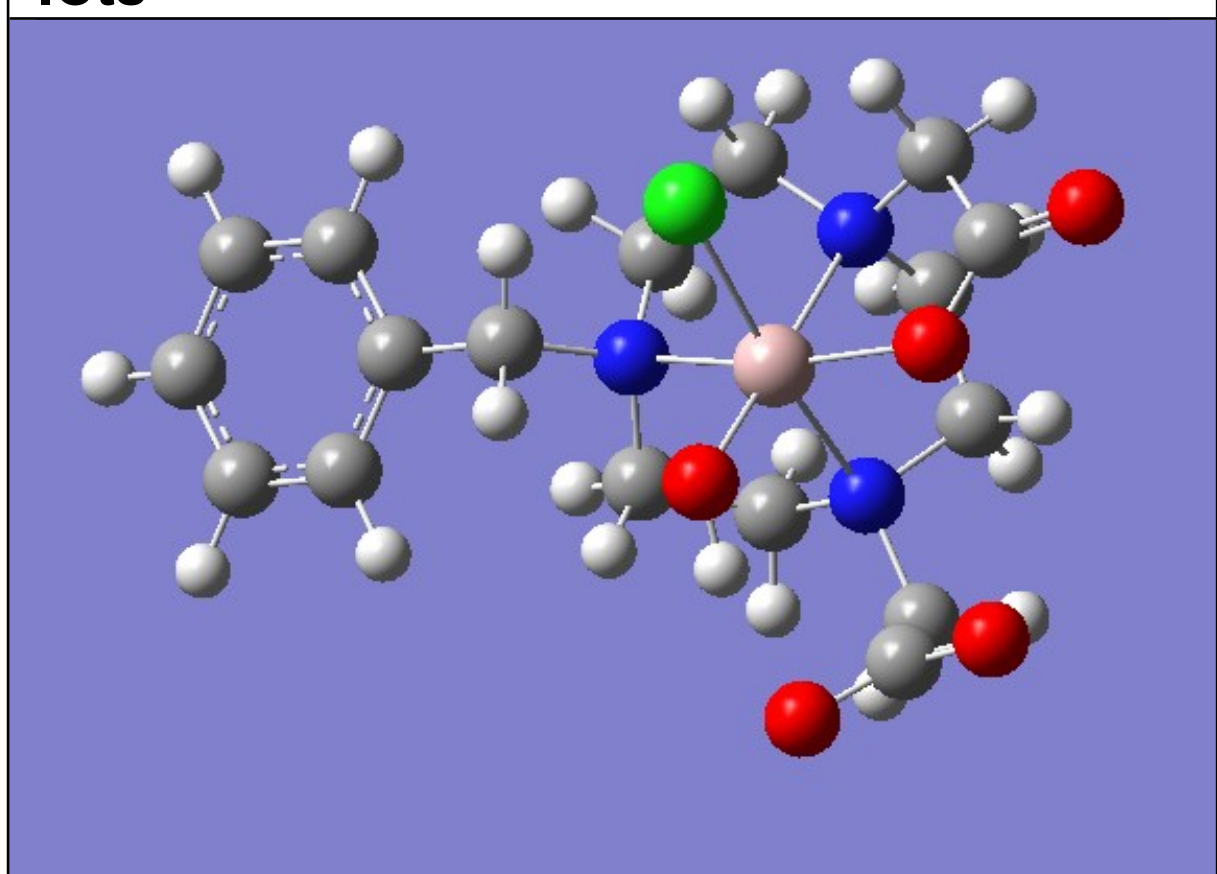	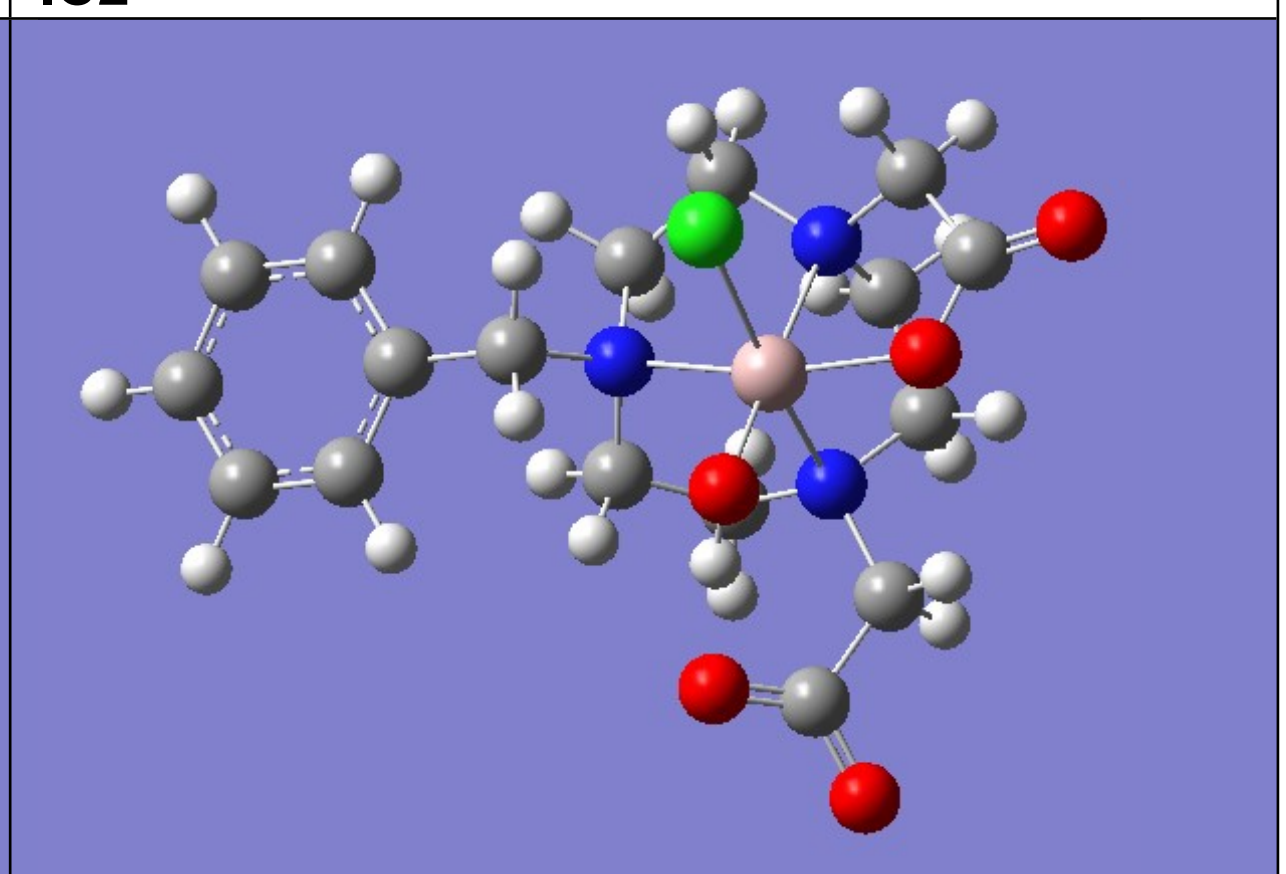
**TS2**	**PC**

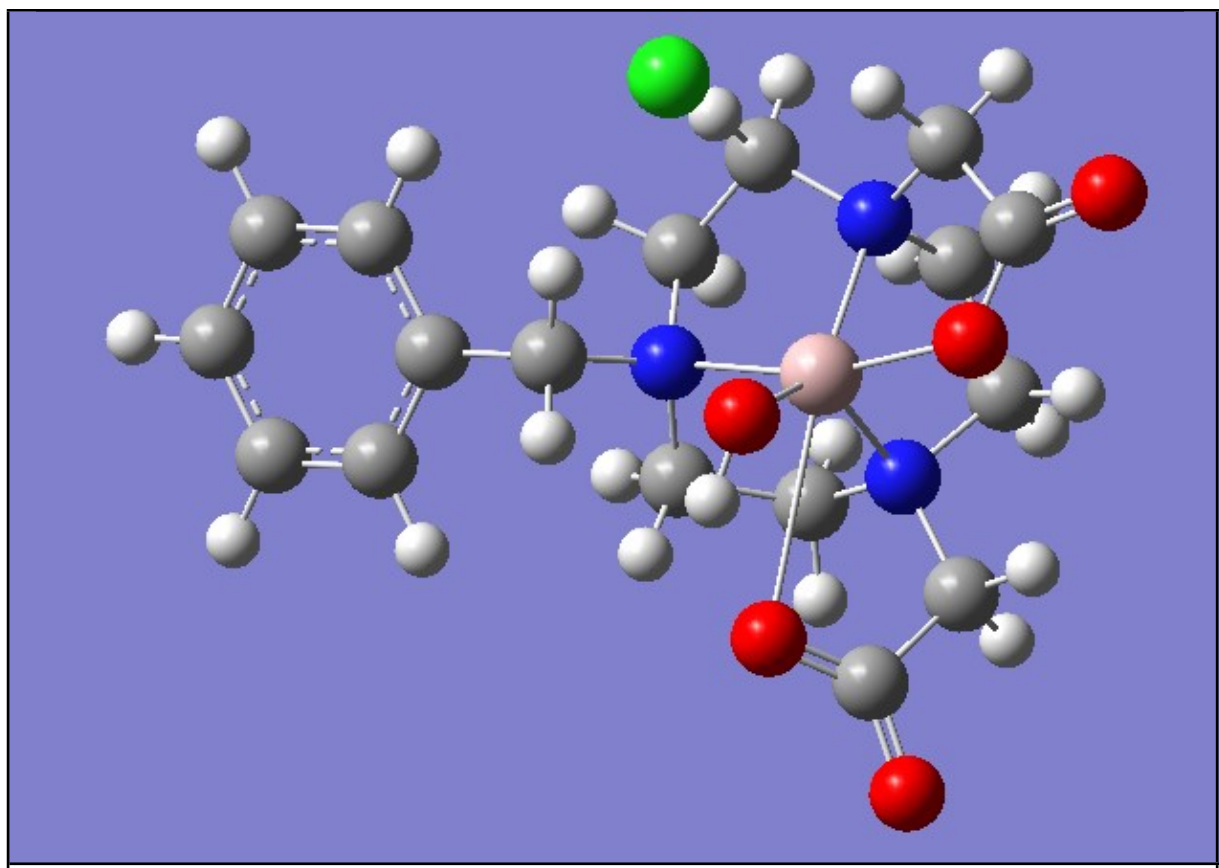

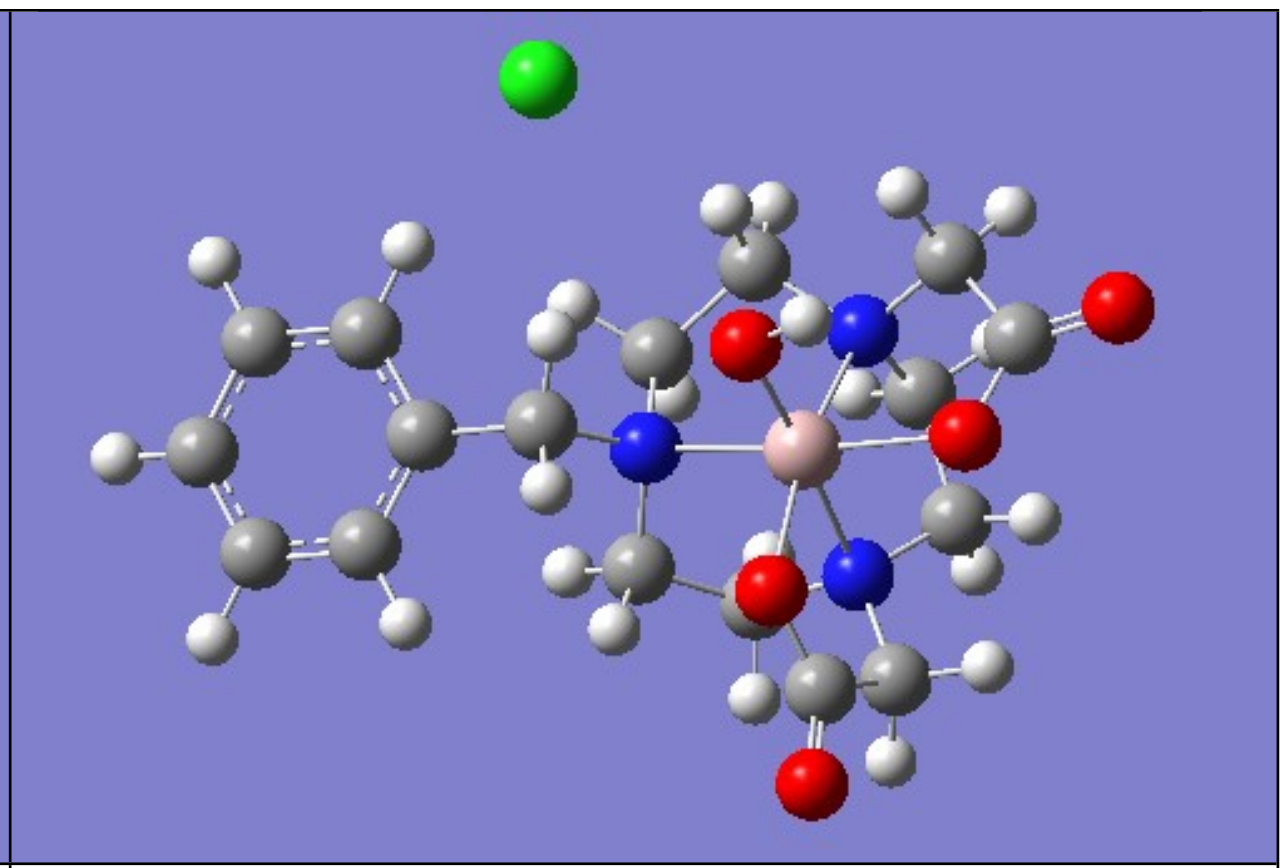

[Al(OH)(L)]

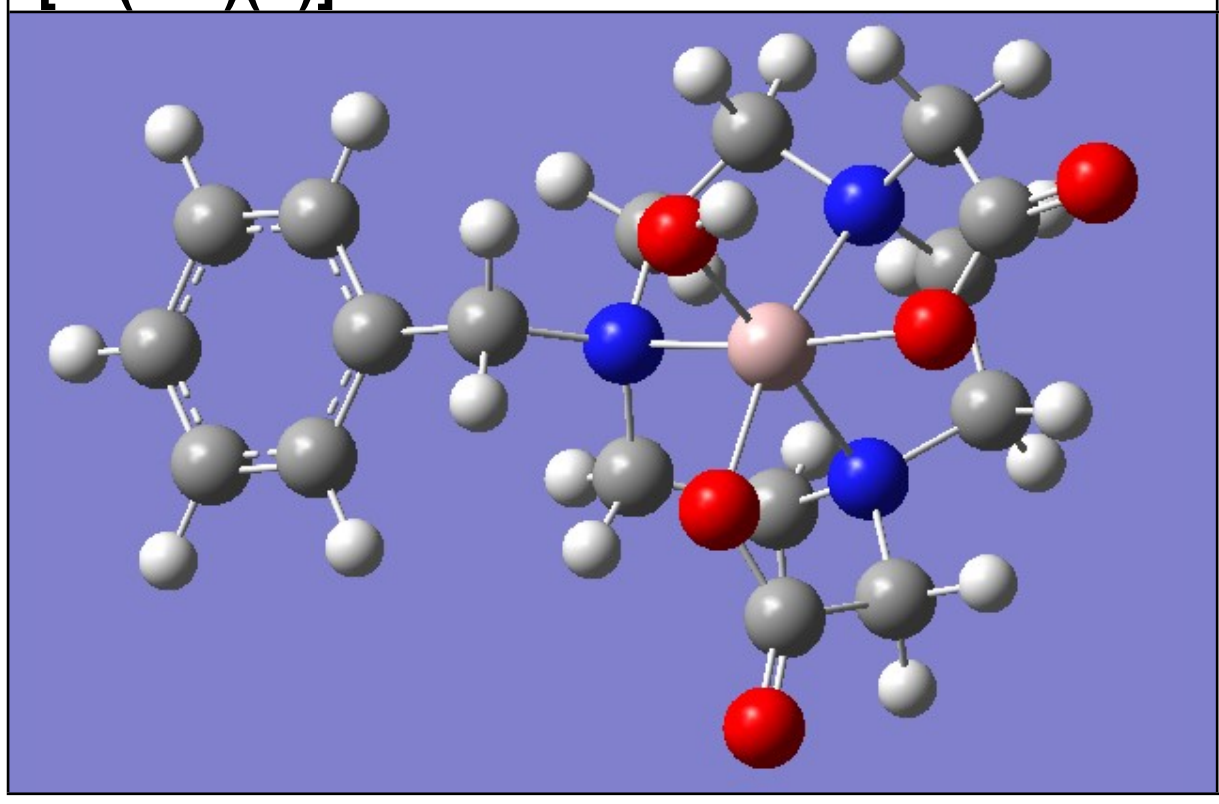

4c) Solution-phase calculations: Charge Density Table (Mulliken Analysis)

Mulliken charges of [AlCl(L)] + OH^- geometries around Al centre. In solution/6-311++G(d,p)

	[AlCl(L)]	RC	TS1	IC1	ICts	IC2	TS2	PC	[Al(OH)(L)]
**Al**	1.19341	1.155207	1.26607	1.896606	1.699081	1.769515	-0.64785	1.684862	1.625694
**Cl**	-0.73261	-0.70291	-0.67282	-0.98733	-0.78314	-0.65430	-0.86620	-0.87951	--X--
**O**$_{(OH)}$	*--X--*	-1.17868	-1.07320	-0.86026	-0.86179	-0.91034	-0.70698	-1.03723	-1.06371
**O**$_{(2)}$	-0.49589	-0.50040	-0.51387	-0.36478	-0.39528	-0.46979	-0.33121	-0.61584	-0.61027
**O**$_{(3)}$	-0.46445	-0.43212	-0.30516	-0.43021	-0.48197	-0.48942	-0.50834	-0.57931	-0.58859
**N**$_{(4)}$	-0.14800	-0.13652	-0.24235	-0.03788	0.04446	-0.12338	0.282821	-0.04026	-0.03992
**N**$_{(5)}$	-0.30794	-0.29999	-0.18453	-0.30311	-0.28326	-0.30715	-0.16029	-0.16708	-0.18222
**N**$_{(14)}$	-0.09577	-0.05134	0.23709	-0.18617	-0.08079	-0.10094	0.094585	-0.07496	-0.13903

Atom numbers for N atoms and carboxylate atoms around Ga vary slightly between geometries, reported values are the equivalent positions, with the carboxylate oxygens being the closest to the metal centre.

5. Supplementary data for [GaF(L)] + OH^{-} → [Ga(OH)(L)] + F^{-} reaction

5a) Solution-phase calculations: Geometry Tables

Note: F/OH-Ga-$N_{(trans)}$ values are taken as the closest to 180º, Ga-$N_{(trans)}$ is the N atom trans to Cl. Ga-O taken as the shortest Ga-O distance from each carboxylate arm.

	**[GaF(L)]**	**RC**	**TS1**	**IC1**	**ICts**	**IC2**	**TS2**	**PC**	**[Ga(OH)(L)]**
Ga-F	--X--	1.87578	1.85815	1.92230	1.88595	1.92382	3.19385	5.58351	--X--
Ga-OH	1.87615	5.53944	2.86448	1.88531	1.93538	1.87694	1.84933	1.89507	1.89525
Ga-O	1.98074<br>2.02988	1.98043<br>2.03441	2.03154<br>2.30603	2.05632<br>3.58710	2.07783<br>3.81080	2.05834<br>3.68275	2.03597<br>3.25980	2.06499<br>2.00559	2.05846<br>2.00667
Ga-N	2.14594<br>2.12196	2.14569<br>2.11878	2.24044<br>2.19004	2.16744<br>2.17134	2.17358<br>2.26798	2.18333<br>2.17611	2.09500<br>2.14560	2.14039<br>2.15105	2.14370<br>2.1412
$GaN_{(trans)}$	2.14836	2.14583	2.14046	2.24026	2.14979	2.23348	2.07885	2.18181	2.17869
N-GaO	164.70717<br>165.13779	164.92312<br>164.89358	151.96280<br>139.66084	161.04573<br>129.43157	158.85867<br>136.90018	160.10554<br>134.13973	164.11642<br>146.04879	161.55877<br>163.11082	161.52024<br>163.08875
F-GaOH	--X--	126.89655	82.13582	96.19263	96.47995	94.07671	75.43339	55.03730	--X--
F-$GaN_{(trans)}$	--X--	176.39656	176.53843	167.46127	163.13342	168.16580	157.09546	124.69316	--X--
OH-$GaN_{(trans)}$	176.24347	124.68801	153.75410	175.63193	168.31365	176.74034	145.55480	175.66047	175.72329
**Energy**	**-3152.410228**	**-3228.394028**	**-3228.370613**	**-3228.413307**	**-3228.400175**	**-3228.415363**	**-3228.379310**	**-3228.401974**	**-3128.371685**
**Energy G**	**-3152.074653**	**-3228.052972**	**-3228.023779**	**-3228.065608**	**-3228.053898**	**-3228.068562**	**-3228.035839**	**-3228.058100**	**-3128.026033**

[GaF(L)] + OH^{-} = -3152.410228 + -75.980355 = **E = -3228.390583** & G = -3228.062846 au

[Ga(OH)(L)] + F^{-} = -3128.371685 + -100.027891 = **E = -3228.399576** & G = -3228.068082 au

5b) Solution-phase calculations: Image of each stationary point

[GaF(L)]	RC
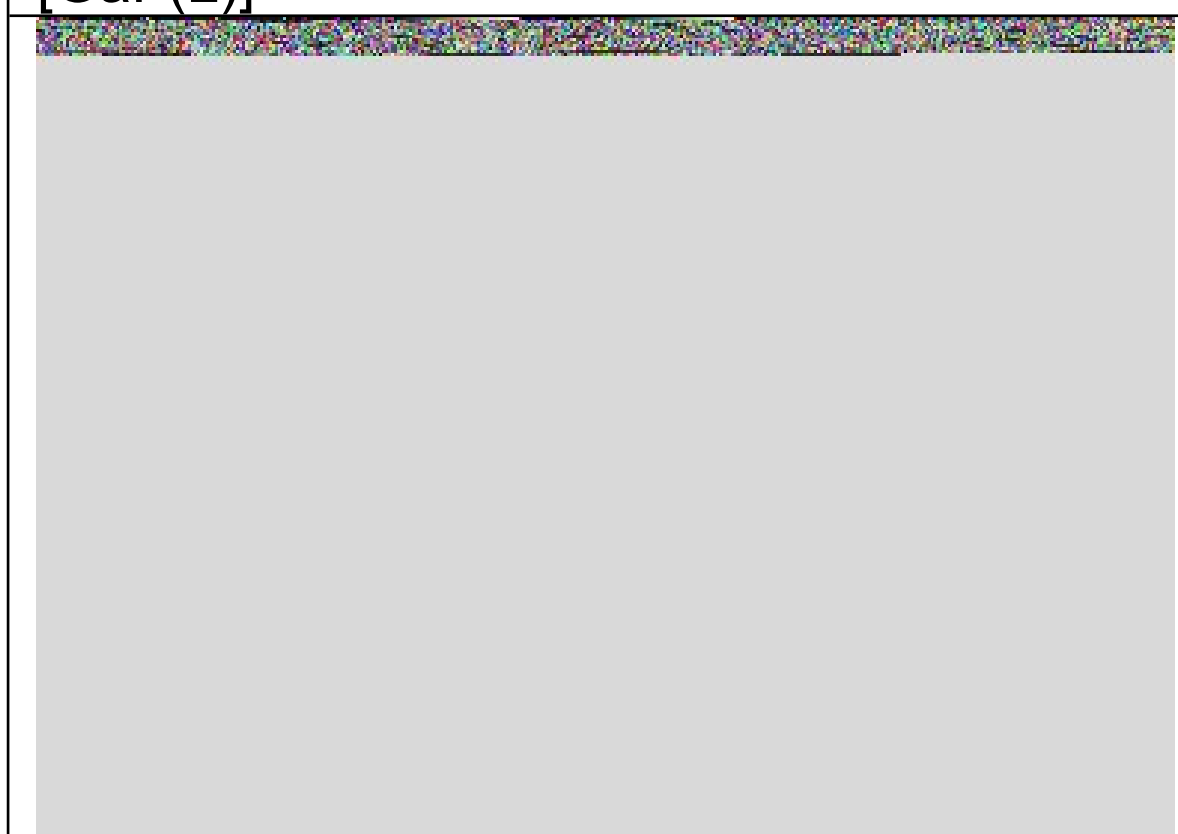	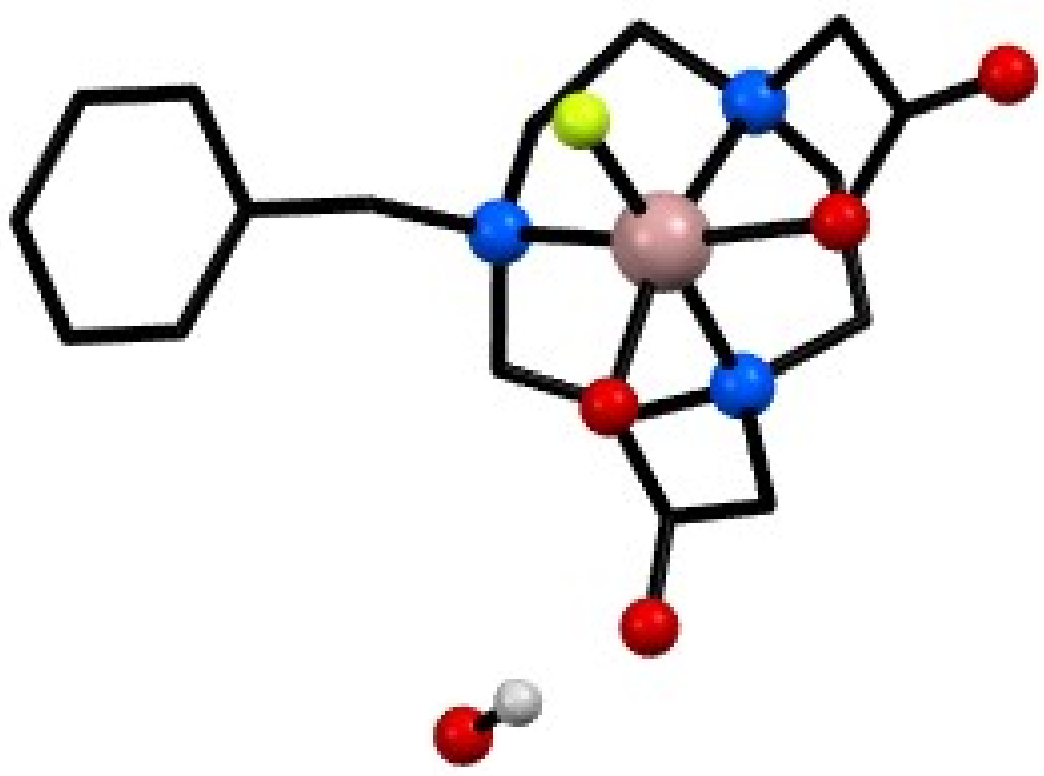
TS1	IC1

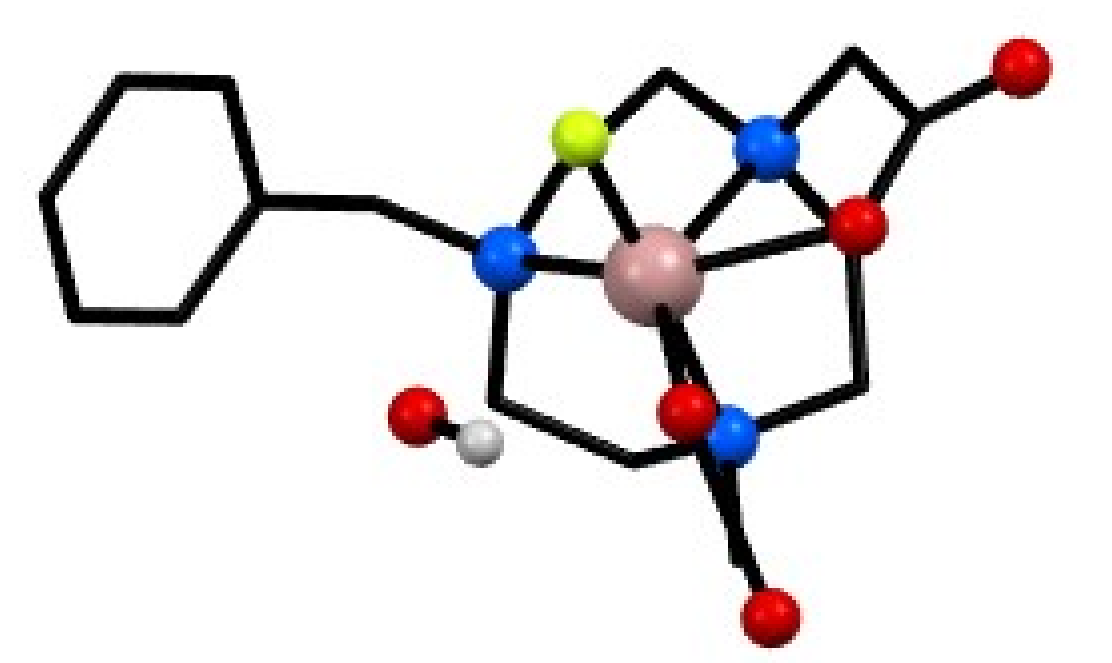	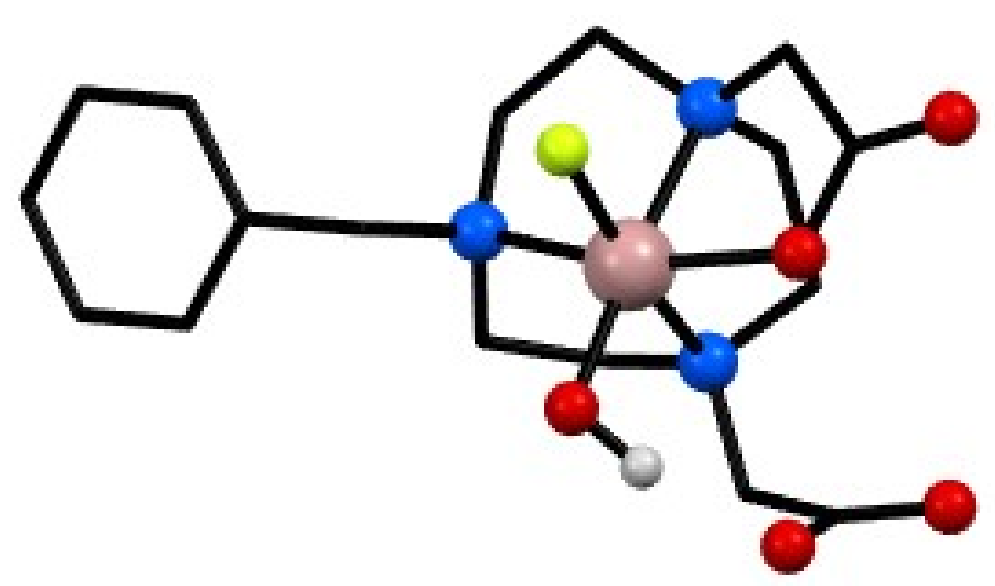
ICts	IC2
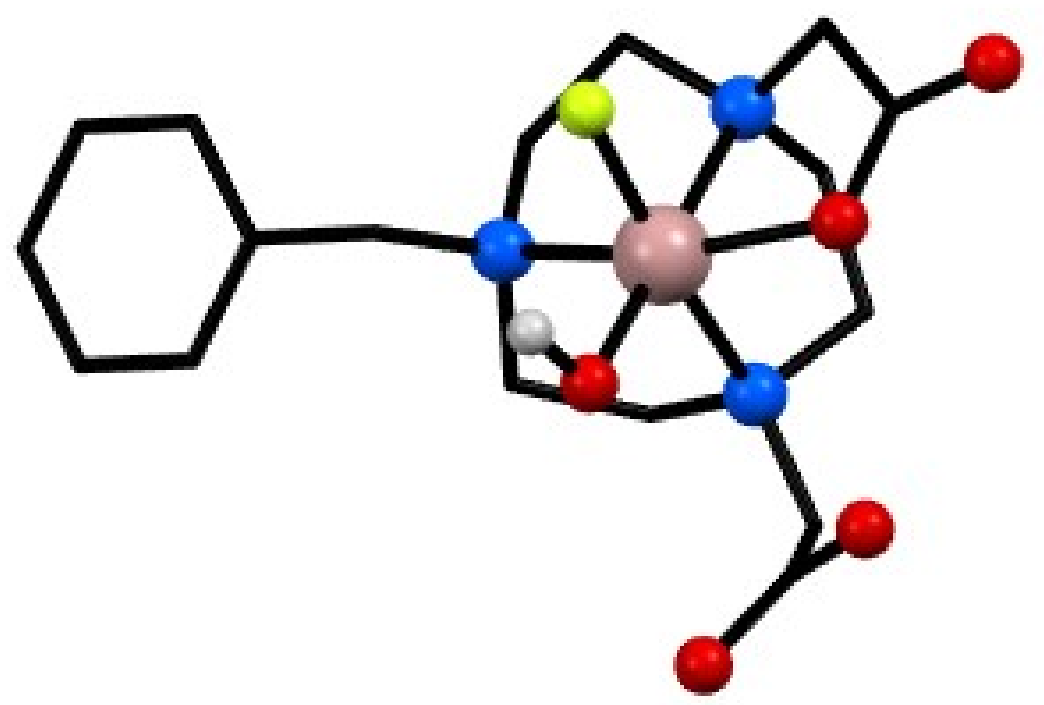	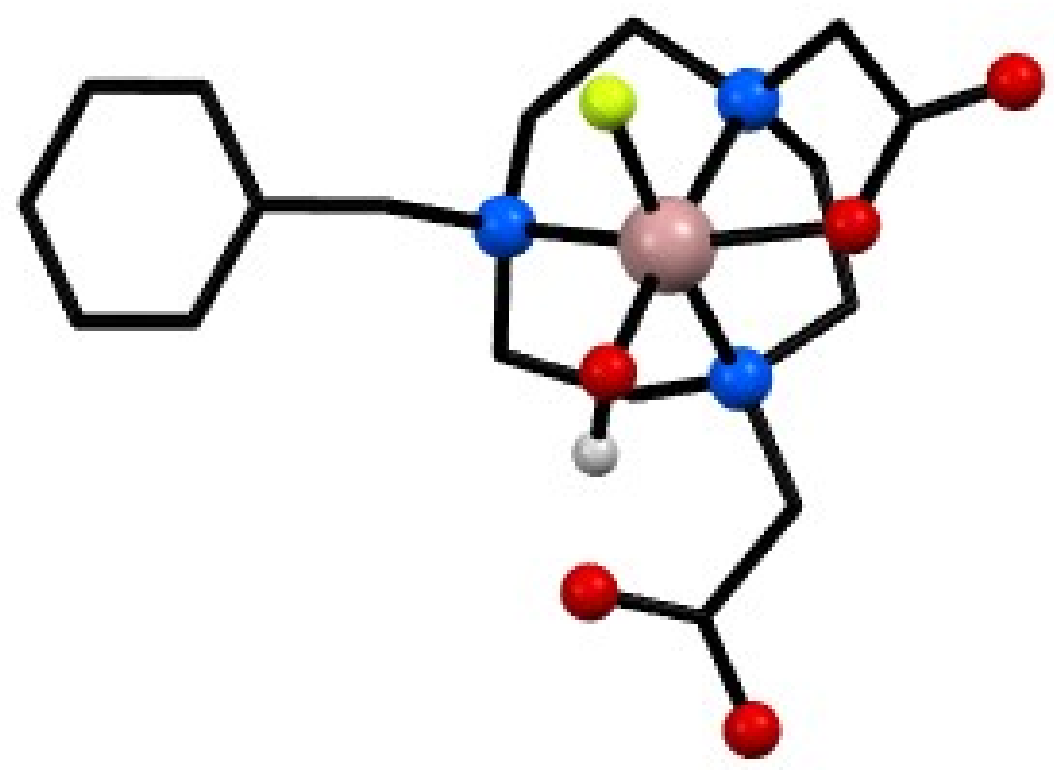
TS2	PC
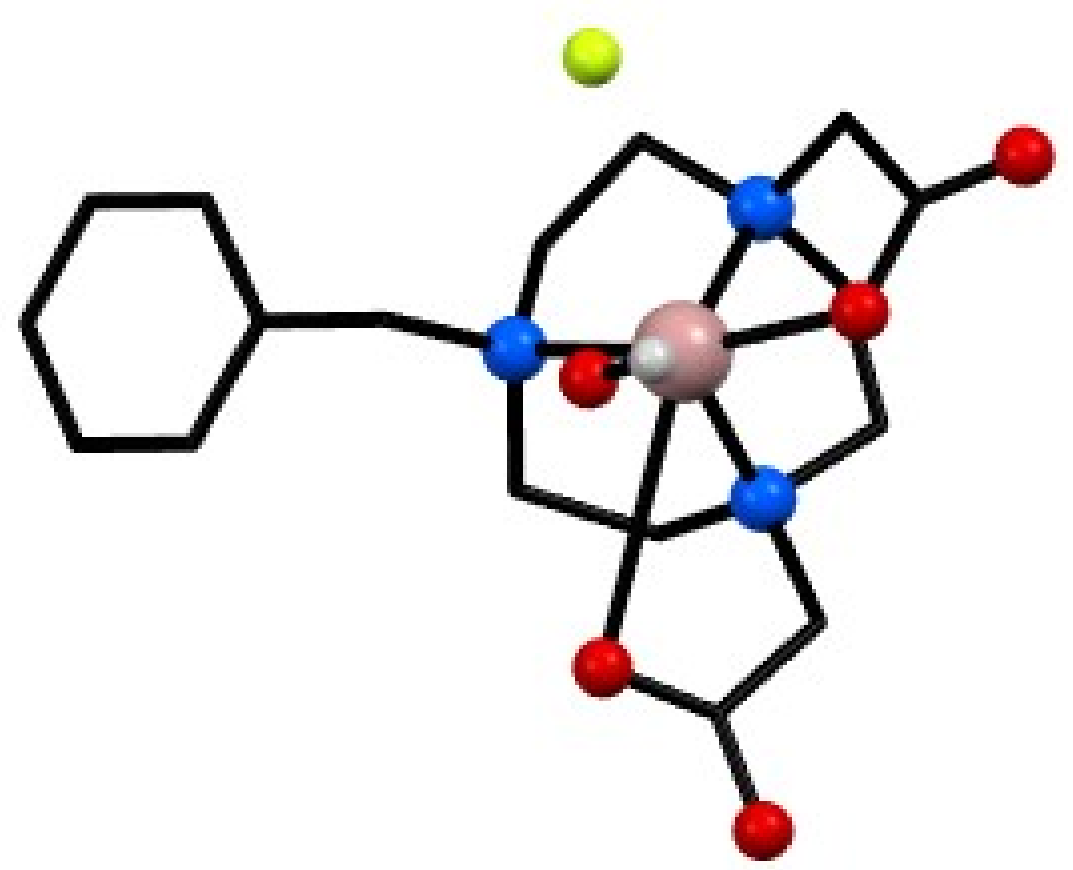	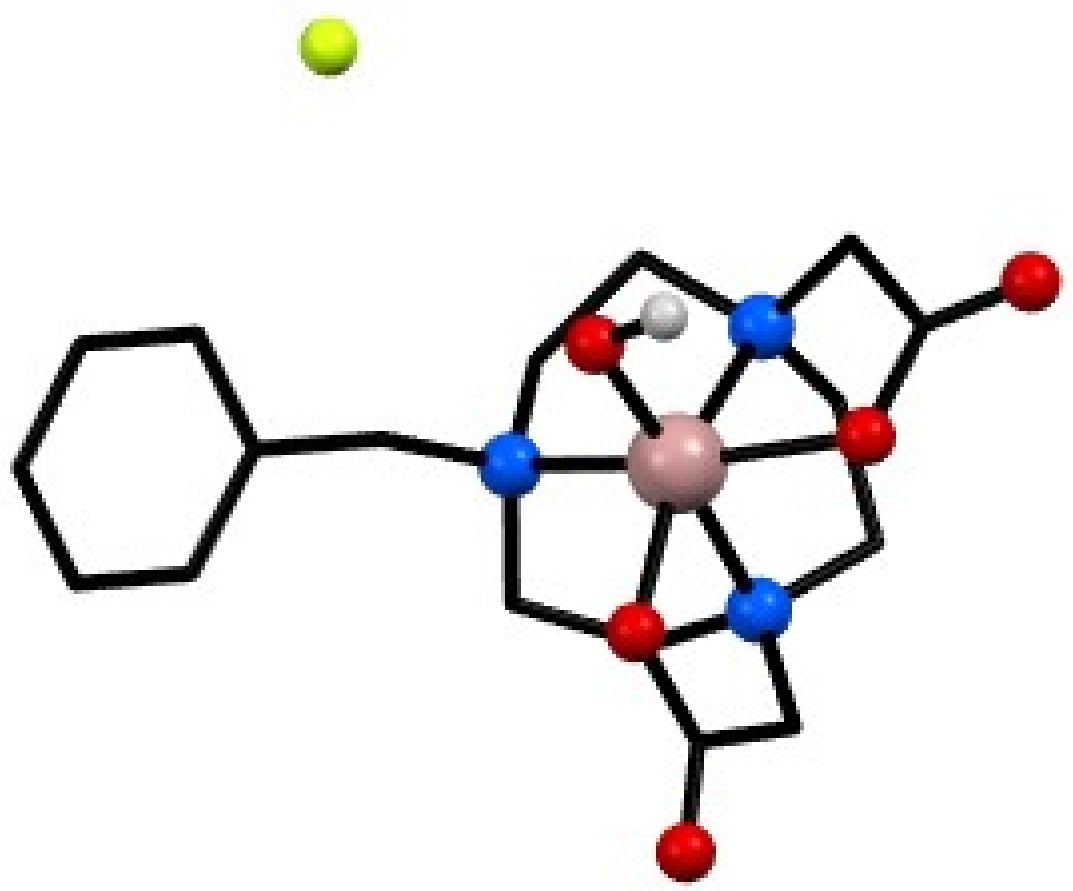
[Ga(OH)(L)]	
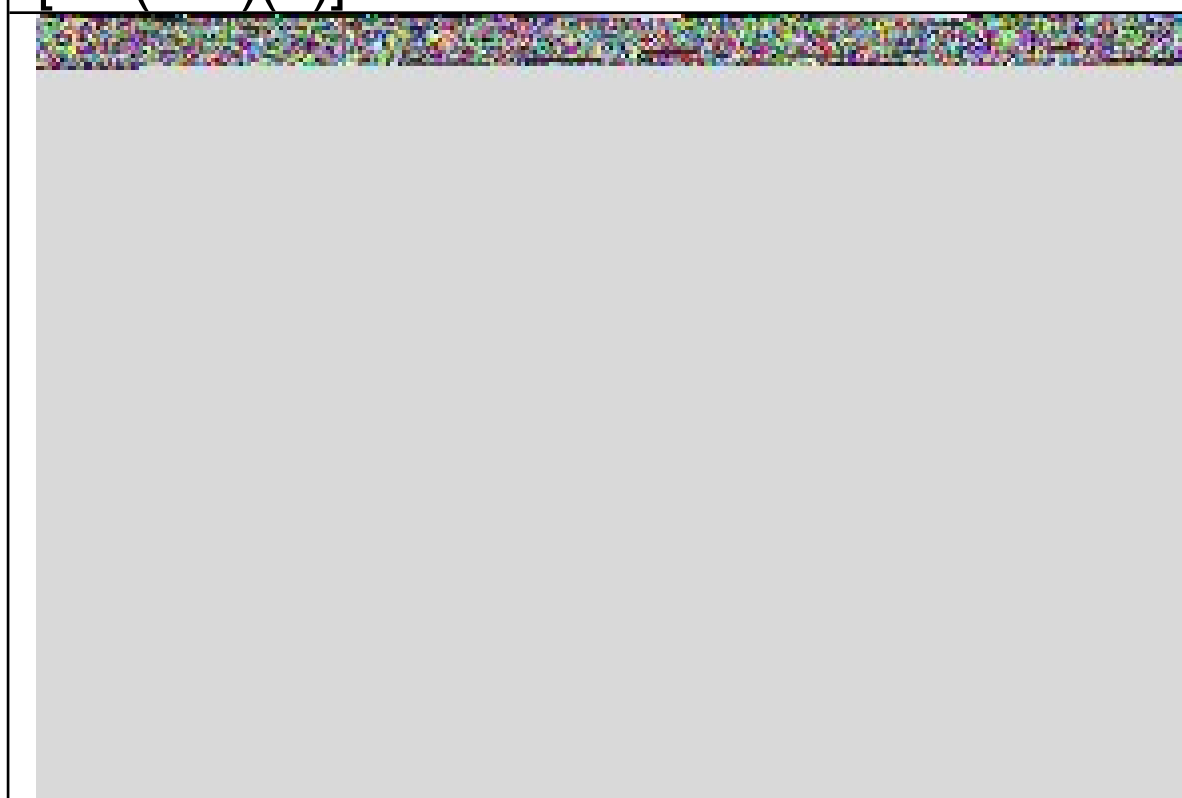	

[GaF(L)] | RC

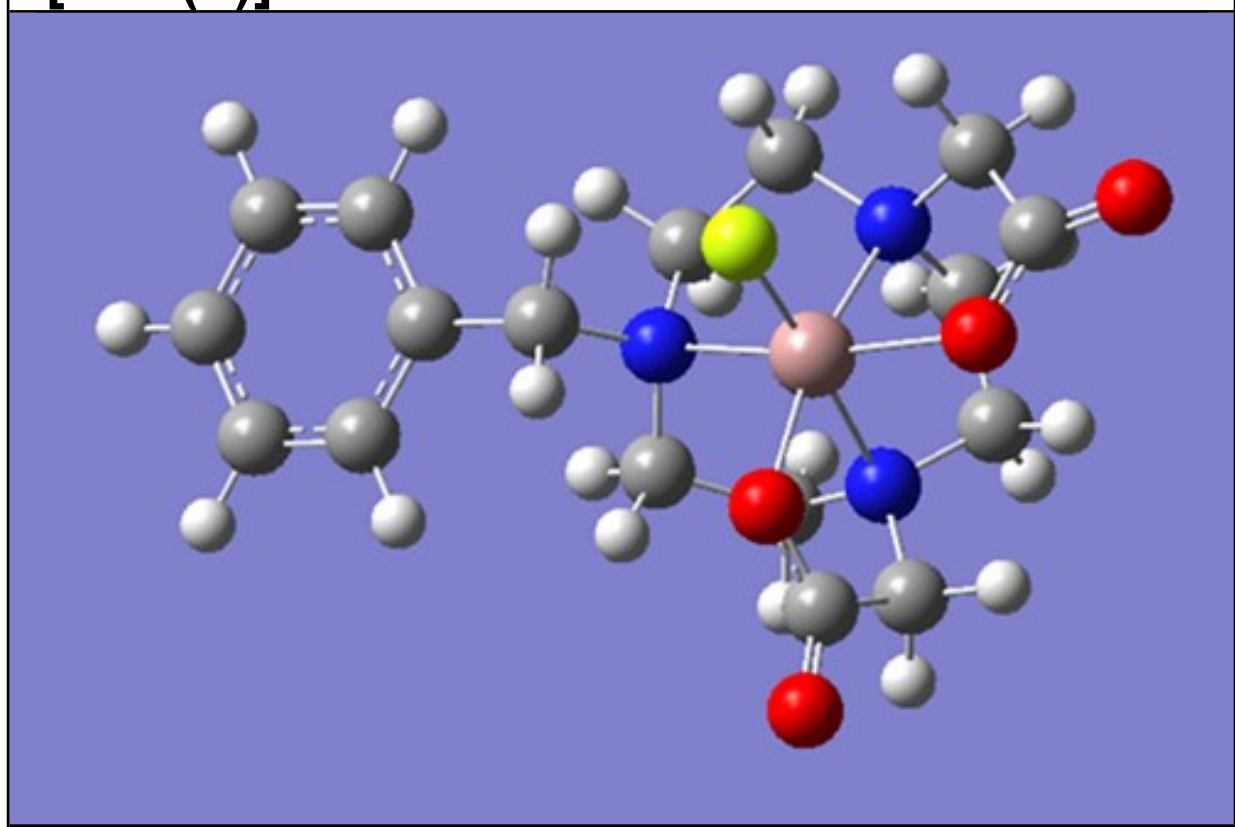

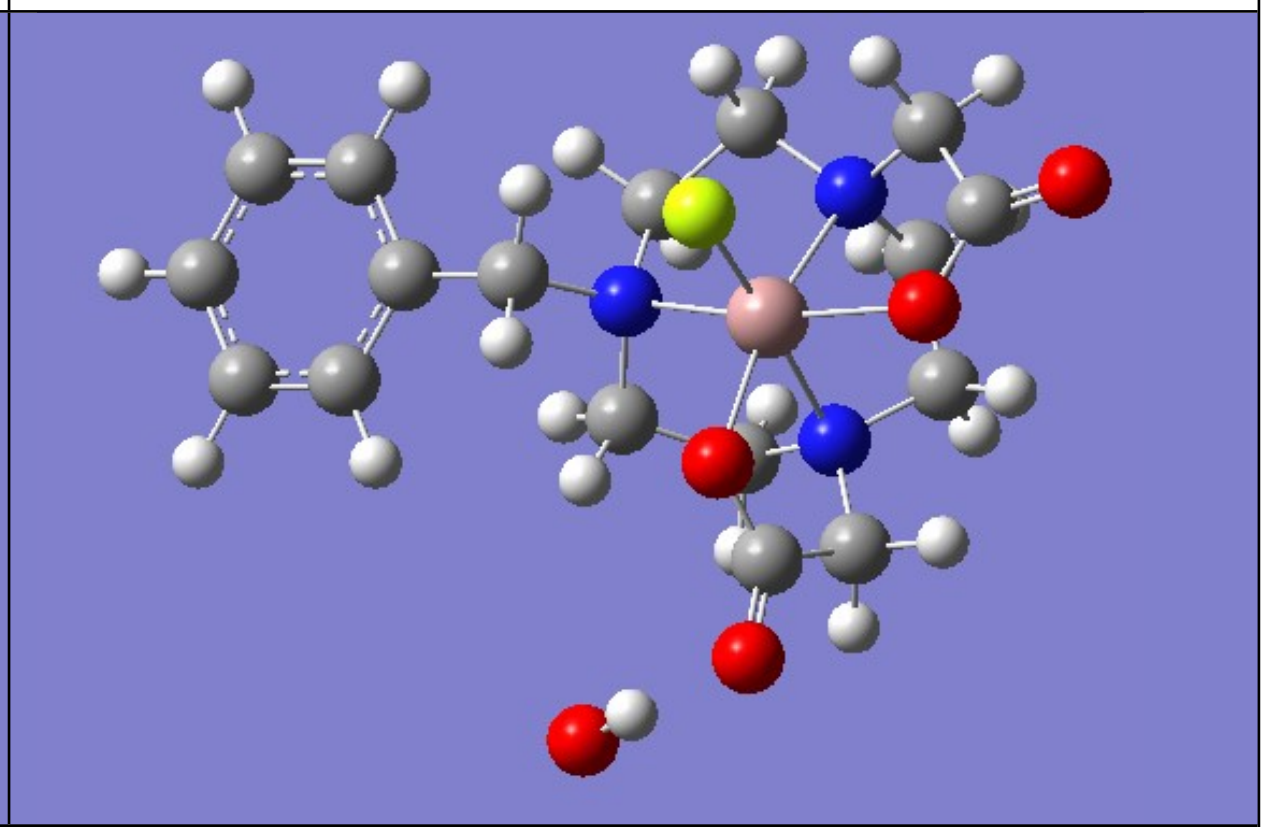

TS1 | IC1

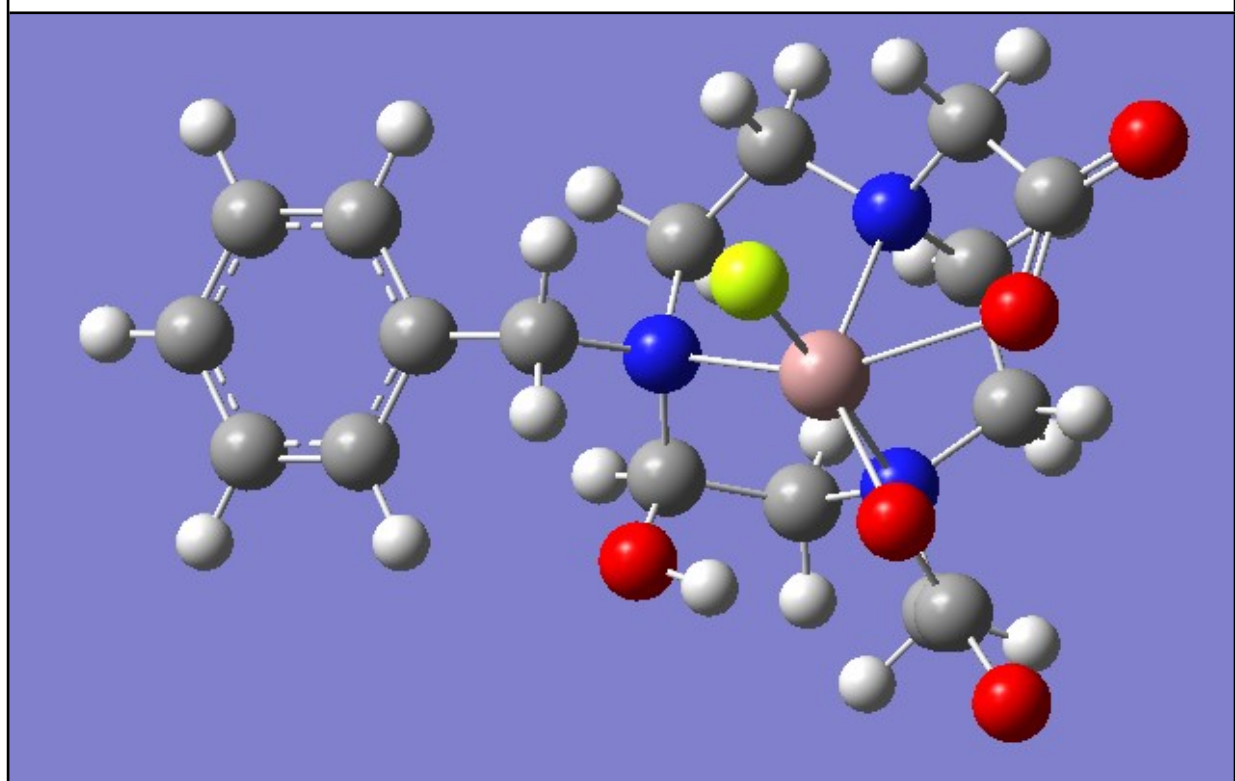

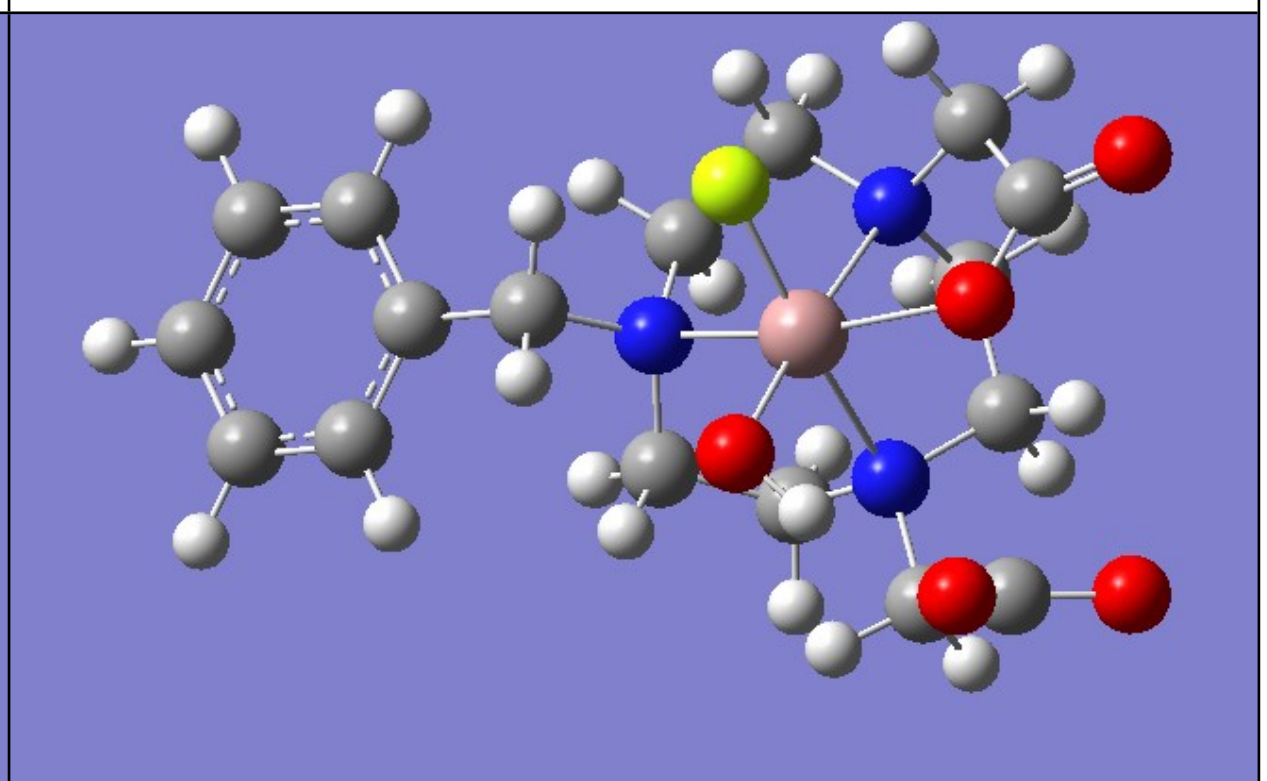

ICts | IC2

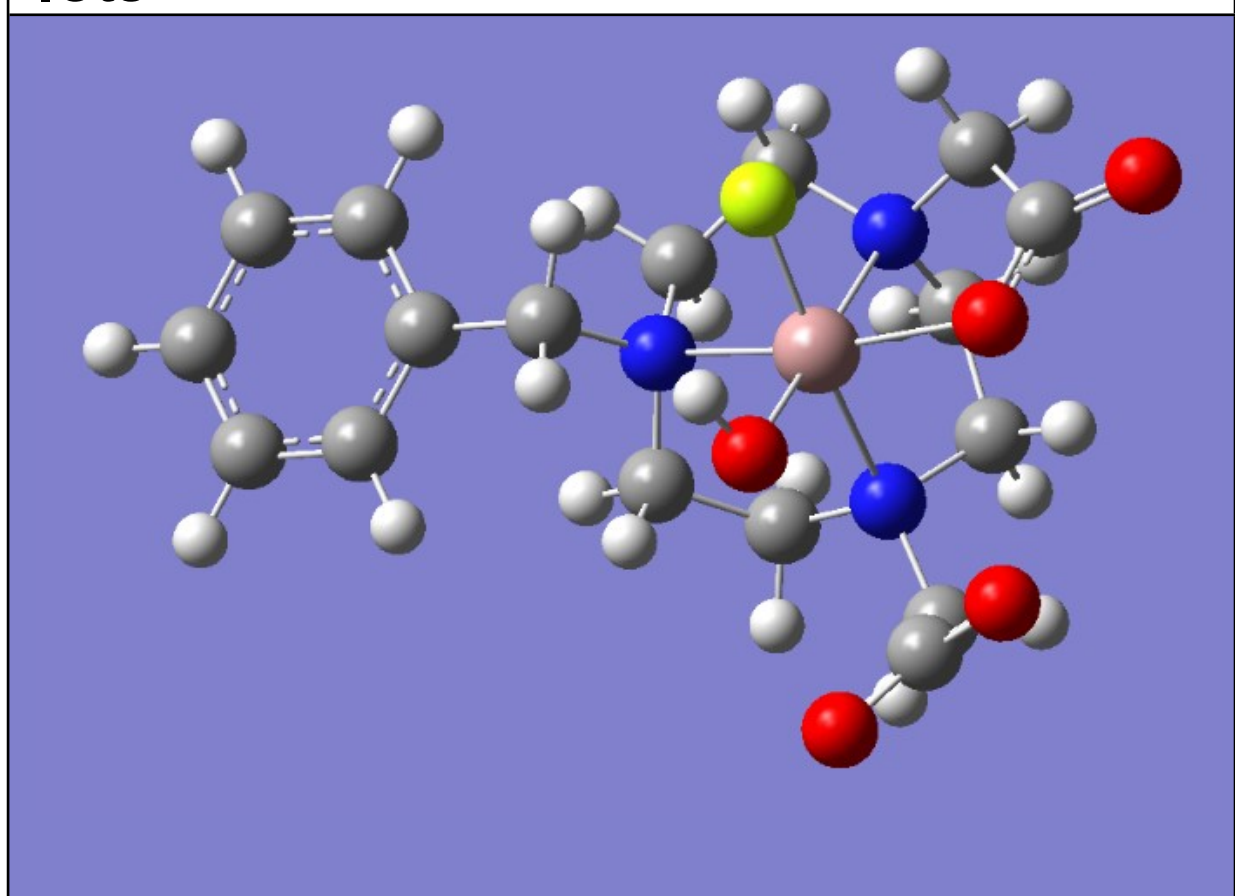

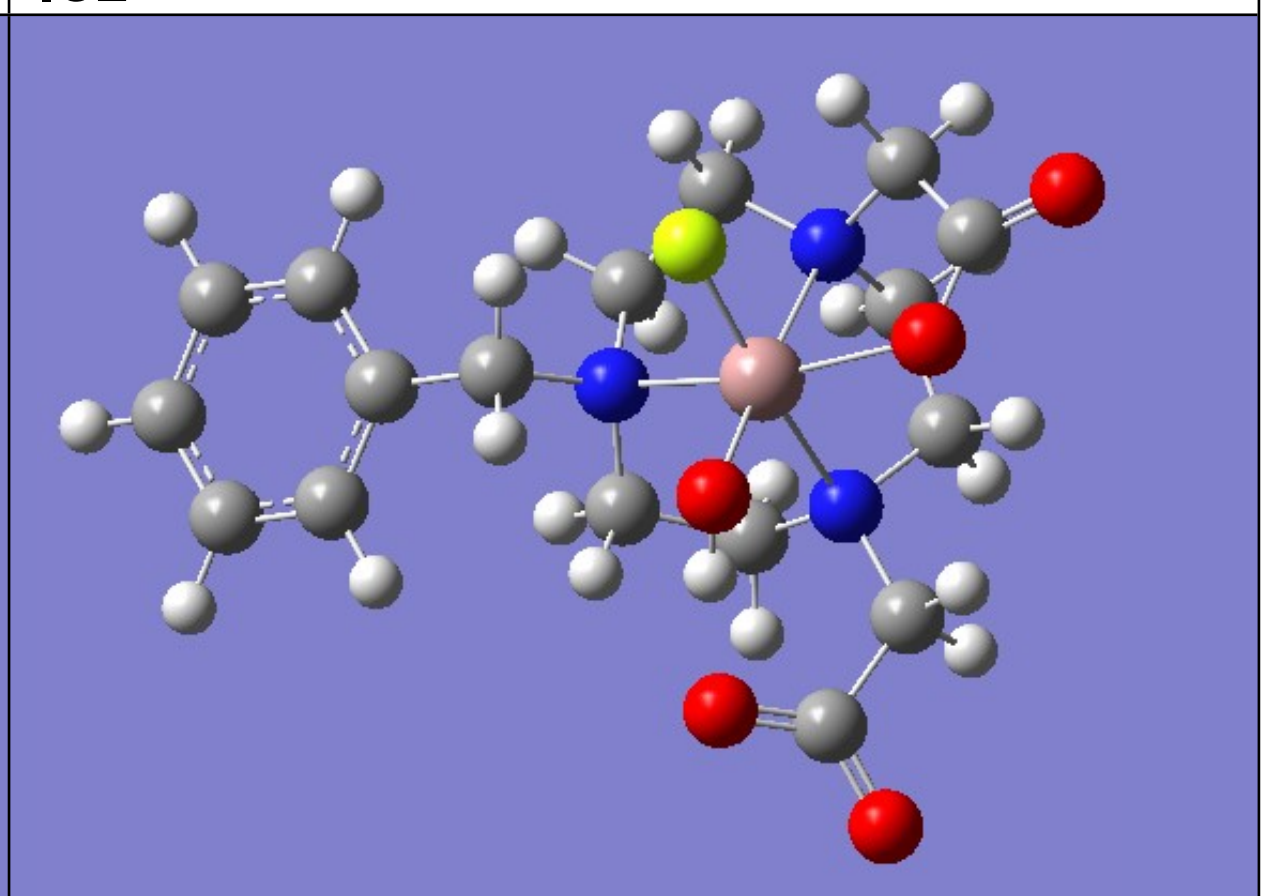

TS2 | PC

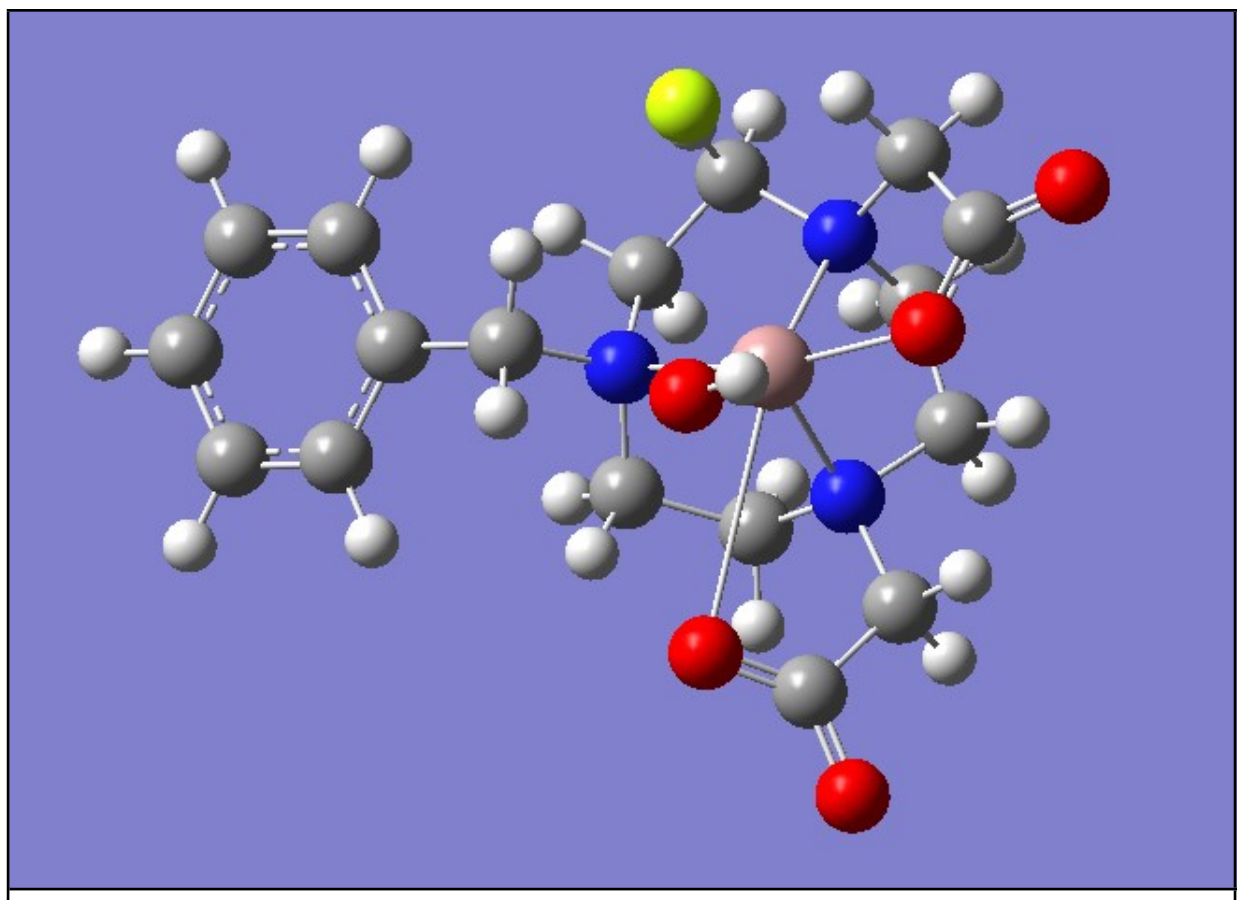	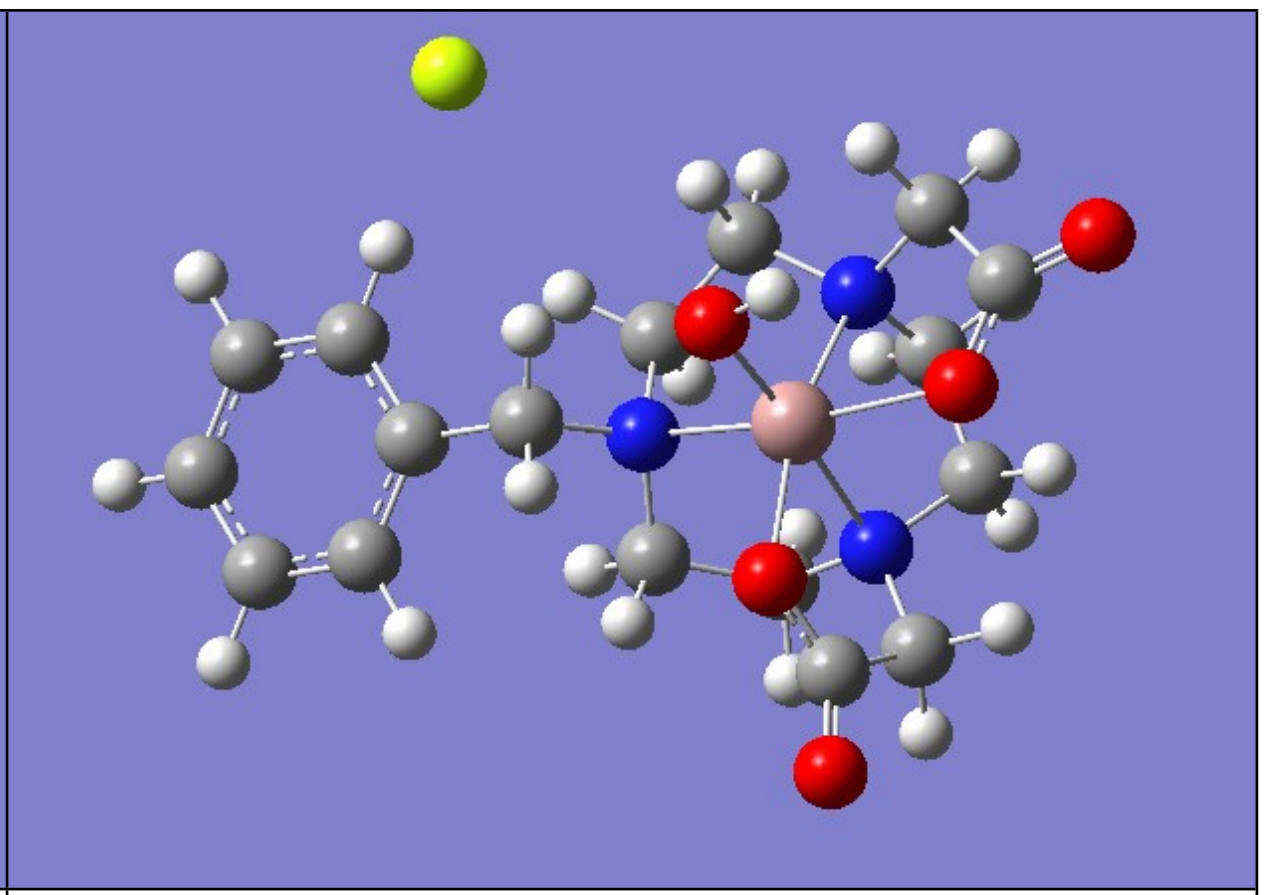
**[Ga(OH)(L)]**	
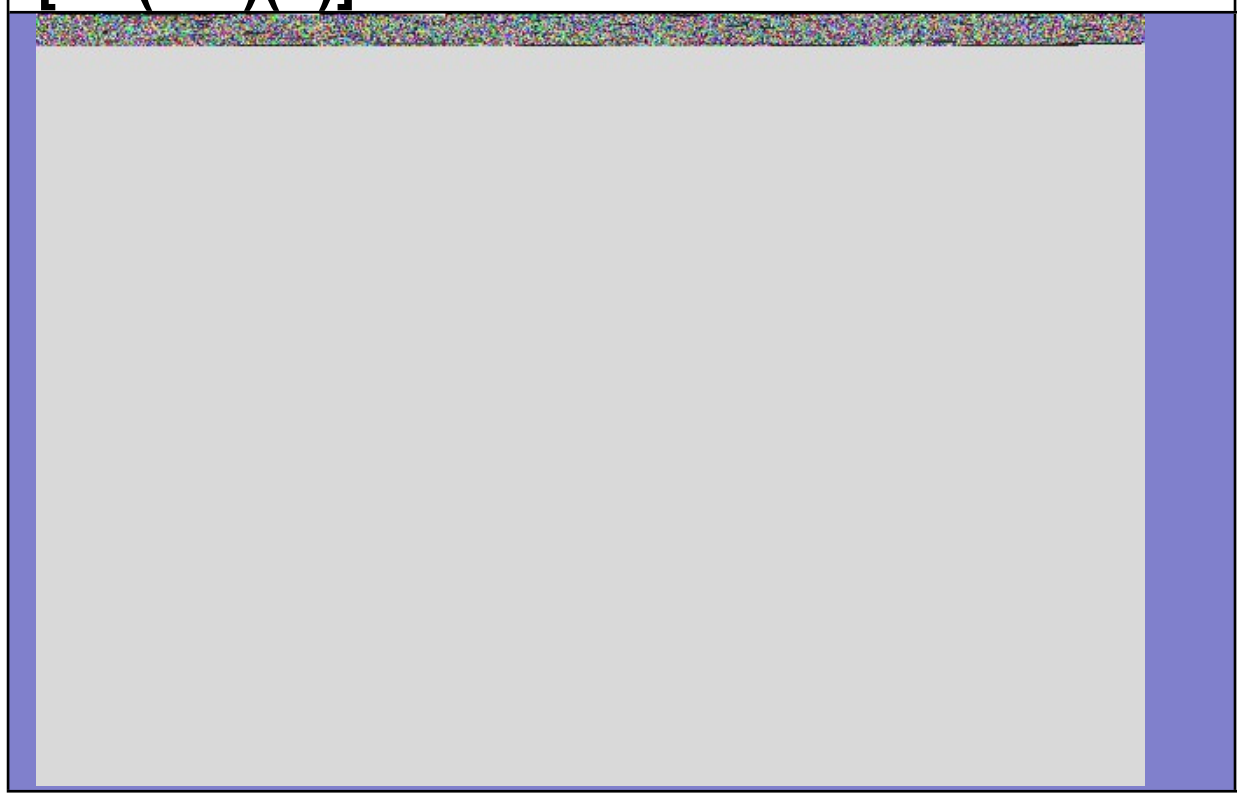	

5c) Solution-phase calculations: Charge Density Table (Mulliken Analysis)

Mulliken charges of [GaF(L)] + OH^- geometries around Ga centre. In solution/6-311++G(d,p)

	**[GaF(L)]**	**RC**	**TS1**	**IC1**	**ICts**	**IC2**	**TS2**	**PC**	**[Ga(OH)(L)]**
**Ga**	2.232263	2.193926	1.947249	2.076107	1.982391	2.249453	1.733027	2.112474	2.113961
**F**	-0.66119	-0.6586	-0.62391	-0.69388	-0.65539	-0.69602	-0.80737	-0.88815	--X--
**$O_{(OH)}$**	--X--	-1.16415	-1.12197	-1.03571	-1.04041	-1.07255	-0.84927	-0.98659	-0.99077
**$O_{(1)}$**	-0.65119	-0.64884	-0.59835	-0.60093	-0.5207	-0.63034	-0.50357	-0.61641	-0.61784
**$O_{(2)}$**	-0.64002	-0.59321	-0.49513	-0.45186	-0.56217	-0.50542	-0.51287	-0.4182	-0.6222
**$N_{(3)}$**	-0.14683	-0.06824	-0.33386	-0.10723	-0.11413	-0.08512	-0.03501	-0.09908	-0.0923
**$N_{(4)}$**	-0.32237	-0.32269	-0.2209	-0.07933	-0.05223	-0.06315	-0.10873	-0.2876	-0.28602
**$N_{(13)}$**	-0.25288	-0.22315	0.009785	-0.18264	-0.03522	-0.23879	-0.21987	-0.22078	-0.25973

Atom numbers for N atoms and carboxylate atoms around Ga vary slightly between geometries, reported values are the equivalent positions, with the carboxylate oxygens being the closest to the metal centre.

6. Supplementary data for [AlF(L)] + $OH^{-} \rightarrow$ [Al(OH)(L)] + F^{-} reaction

6a) Solution-phase calculations: Geometry Tables

Note: F/OH-Al-N$_{(trans)}$ values are taken as the closest to 180º, Al-N$_{(trans)}$ is the N atom trans to Cl. Al-O taken as the shortest Al-O distance from each carboxylate arm.

	**[AlF(L)]**	**RC**	**TS1**	**IC1**	**ICts**	**IC2**	**TS2**	**PC**	**[Al(OH)(L)]**
Al-F	--X--	1.76742	1.75397	1.80241	1.80217	1.80305	3.28914	5.57919	--X--
Al-OH	1.76736	5.62090	2.71895	1.81042	1.79946	1.80132	1.78419	1.81620	1.81708
Al-O	1.92933<br>1.88779	1.93083<br>1.88761	2.12306<br>1.96108	1.94517<br>3.61452	1.94082<br>3.87899	1.95024<br>3.72756	1.92131<br>2.84854	1.94501<br>1.90351	1.94241<br>1.90255
Al-N	2.10434<br>2.06779	2.06760<br>2.10225	2.19118<br>2.26849	2.12549<br>2.12596	2.12607<br>2.12304	2.14106<br>2.12866	2.06264<br>2.12239	2.08723<br>2.10649	2.08962<br>2.10834
AlN$_{(trans)}$	2.10244	2.09887	2.09596	2.19230	2.27967	2.19155	2.04258	2.12985	2.12968
N-Al-O	166.57613<br>166.06697	166.56272<br>166.21838	150.49191<br>134.52408	162.67725<br>128.78384	161.84123<br>137.63839	161.40159<br>133.92295	151.82602<br>164.23157	165.35395<br>163.29657	163.24033<br>165.27897
F-AlOH	--X--	129.21308	82.83239	97.76969	94.13877	95.60416	70.72595	51.99987	--X--
F-Al-N$_{(trans)}$	--X--	176.37375	173.28499	167.50853	167.65057	168.43439	155.49015	125.01291	--X--
OH-Al-N$_{(trans)}$	176.14123	122.39049	150.16265	174.42631	177.93502	176.92842	139.38255	175.83553	175.69857
**Energy**	**-1469.872386**	**-1545.856334**	**-1545.821952**	**-1545.872722**	**-1545.859566**	**-1545.874754**	**-1545.822800**	**-1545.857728**	**-1445.827475**
**Energy G**	**-1469.532764**	**-1545.510902**	**-1545.474501**	**-1545.521326**	**-1545.508630**	**-1545.523591**	**-1545.473016**	**-1545.509389**	**-1445.476984**

[AlF(L)] + OH^{-} = -1469.872389 + -75.980355 = **E= -1545.852744** & G = -1545.520957 au
[Al(OH)(L)] + F^{-} = -1445.827475 + -100.027891 = **E = -1545.855366** & G = -1545.519033 au

6b) Solution-phase calculations: Image of each stationary point

[AlF(L)]	RC
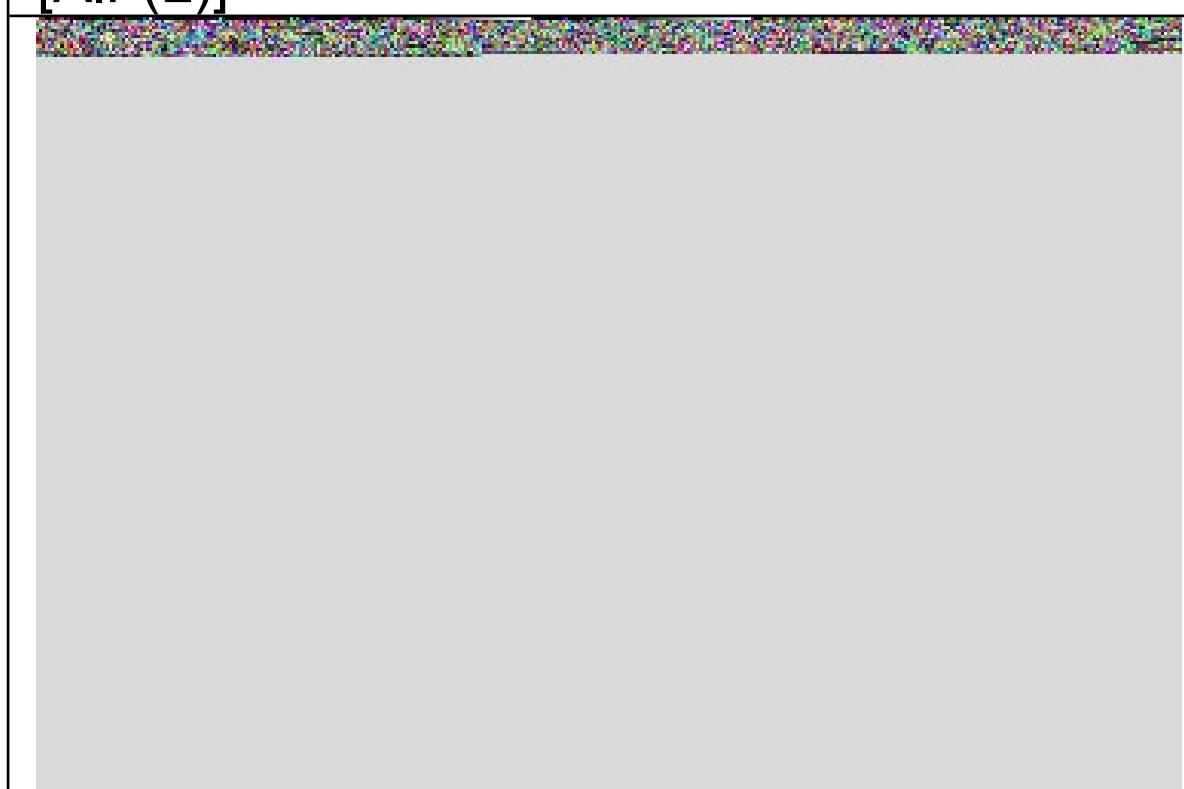	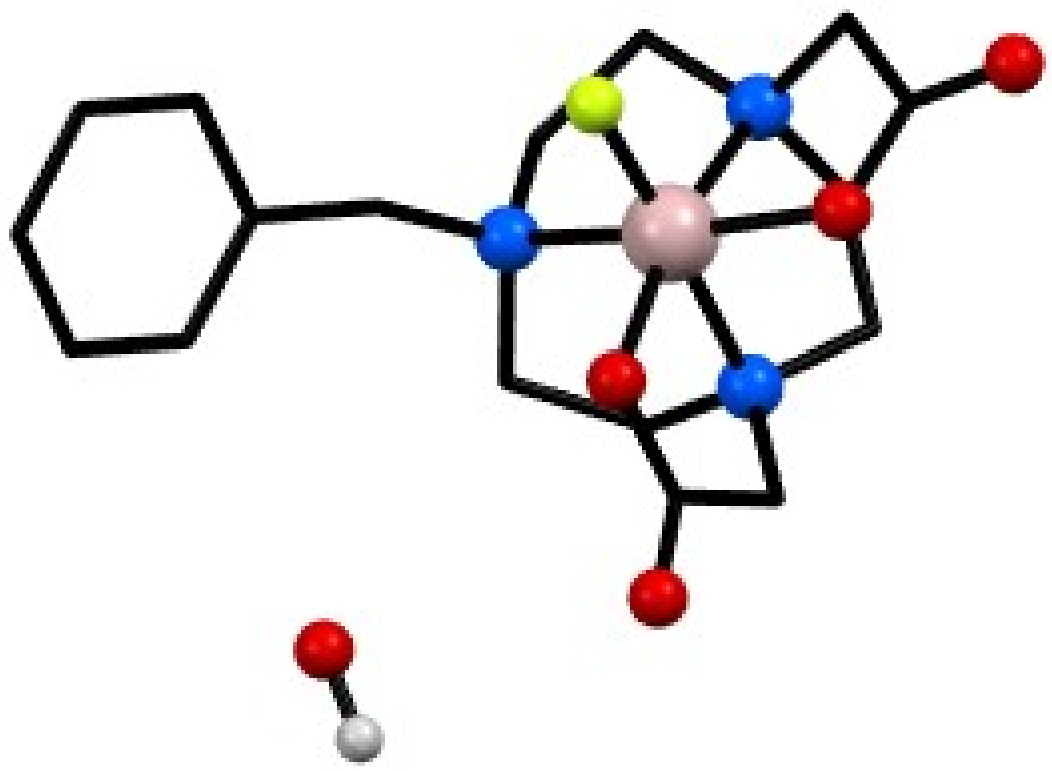
TS1	IC1

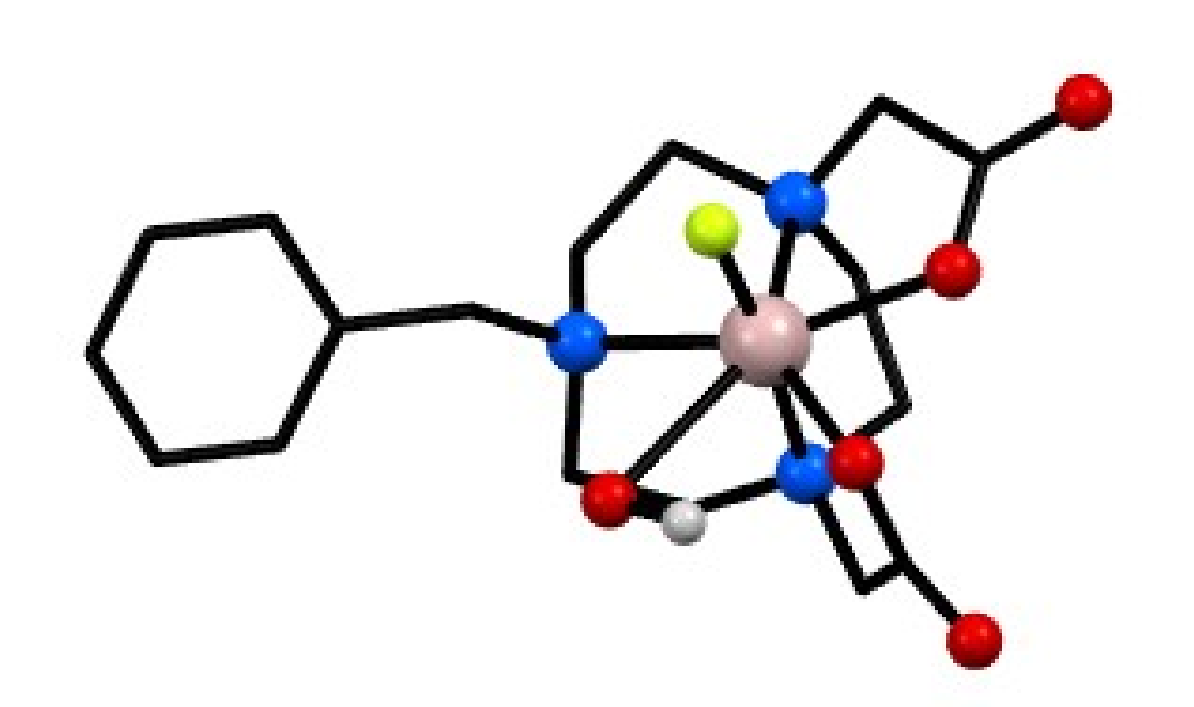	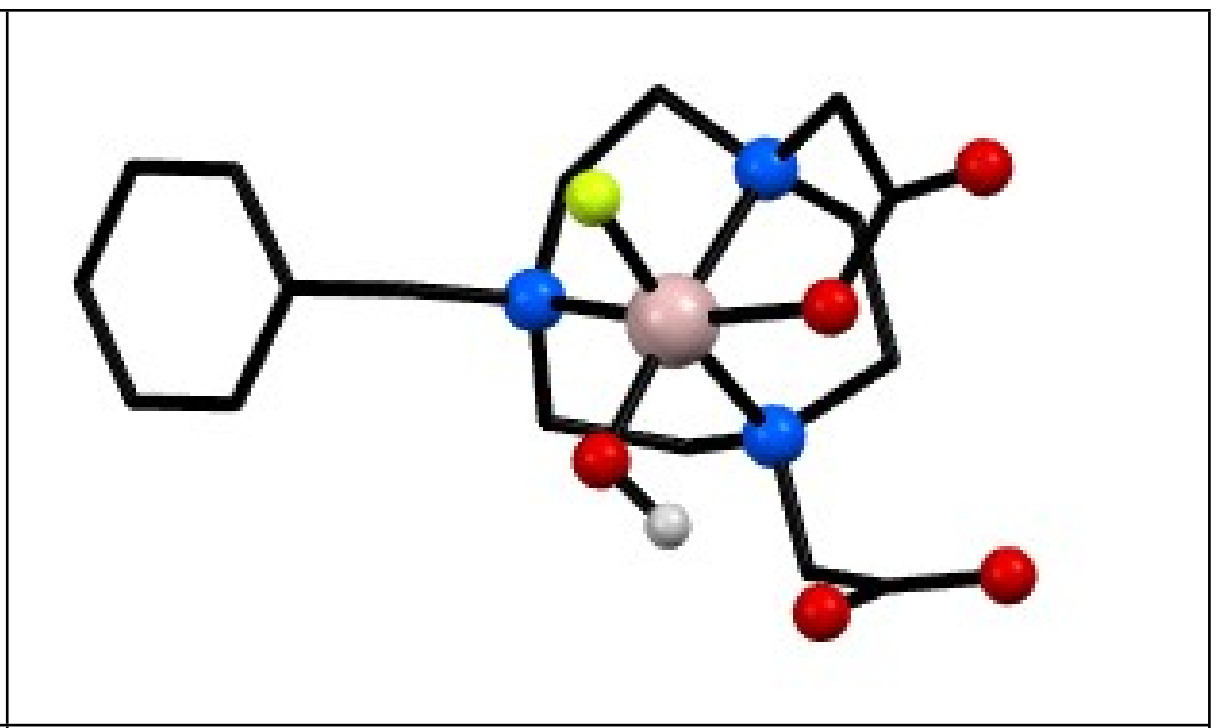
ICts	IC2
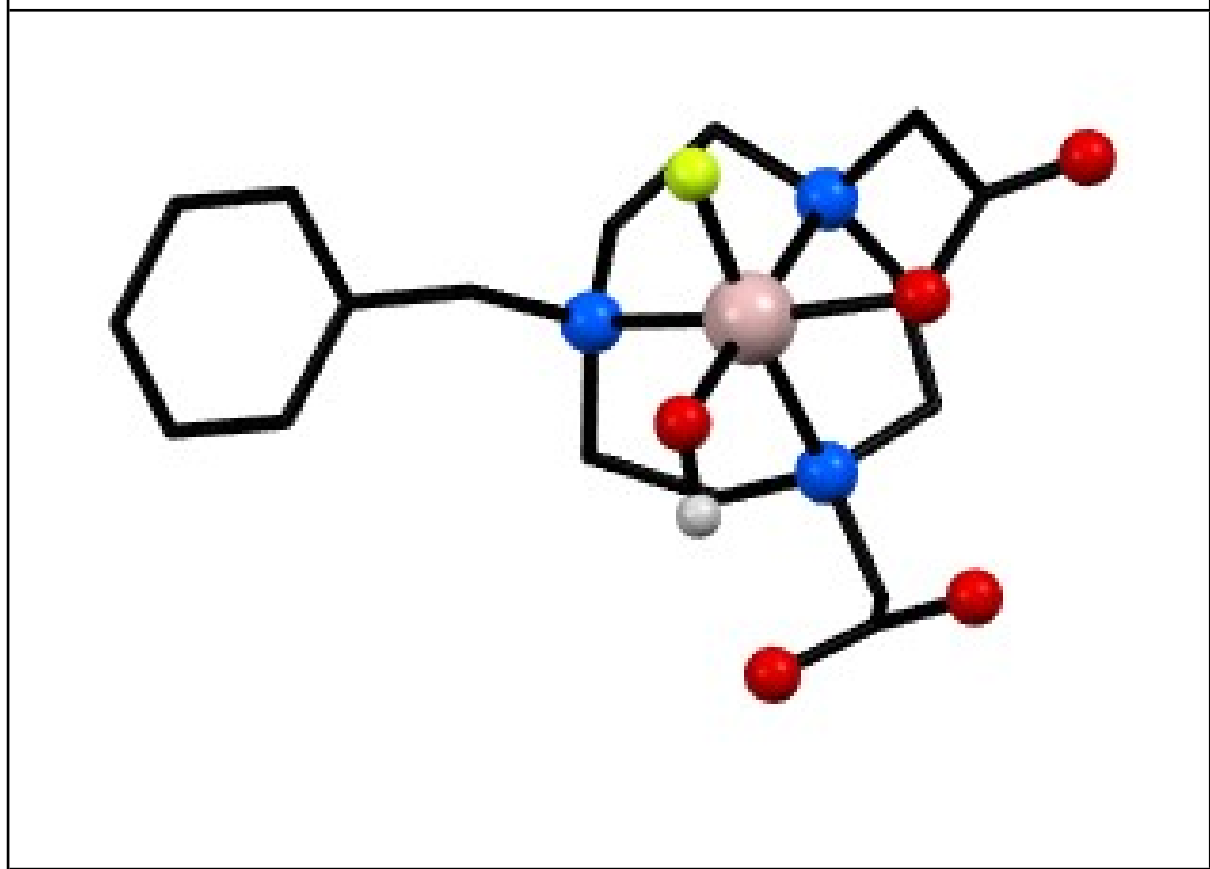	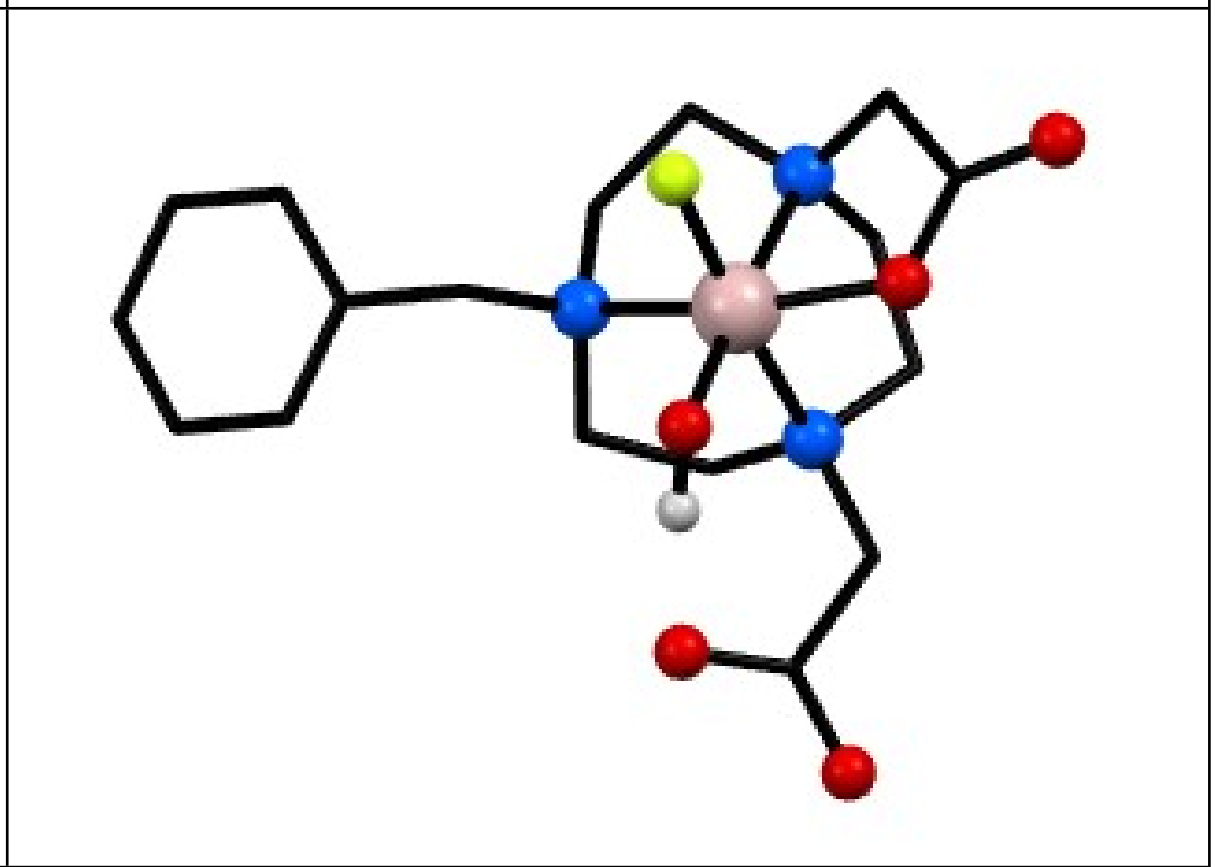
TS2	PC
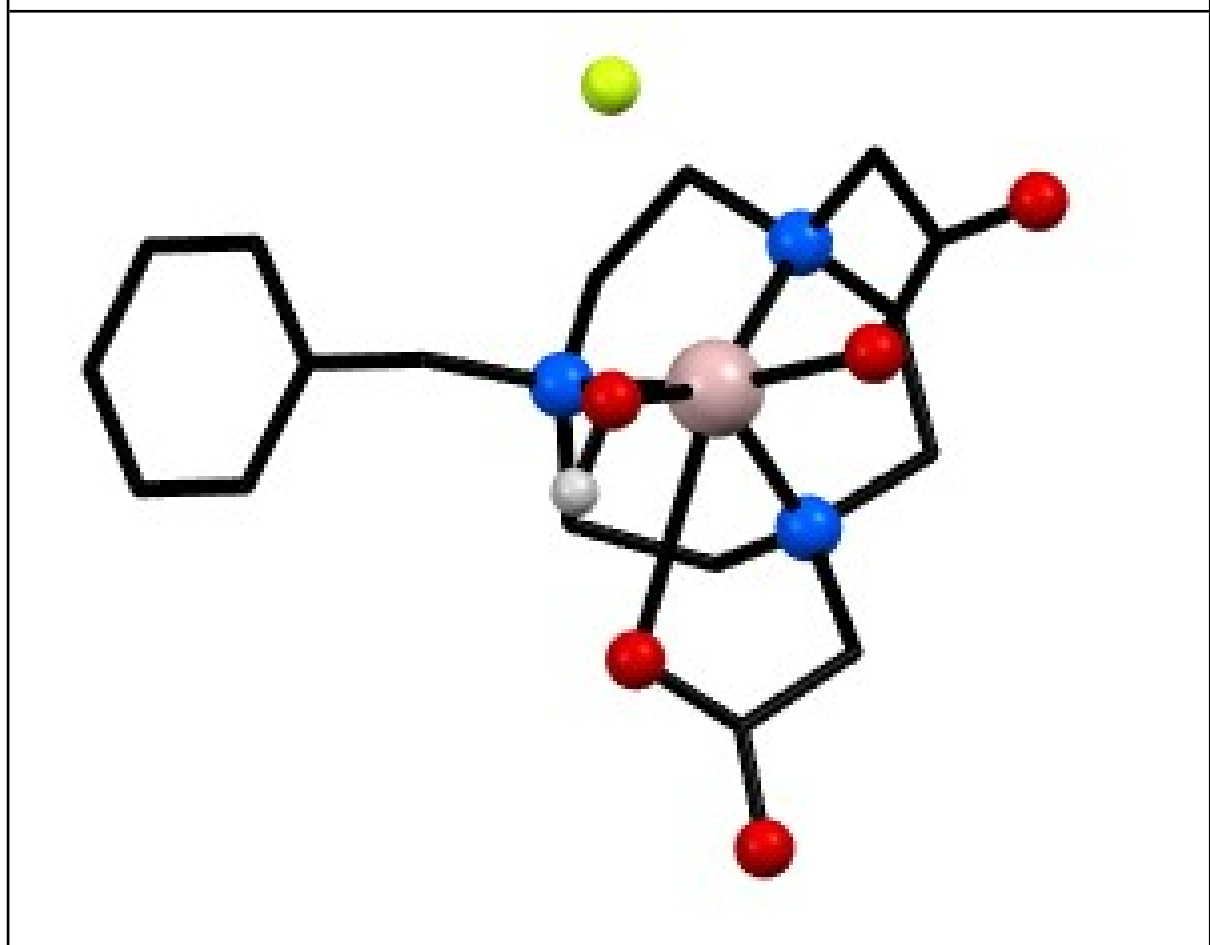	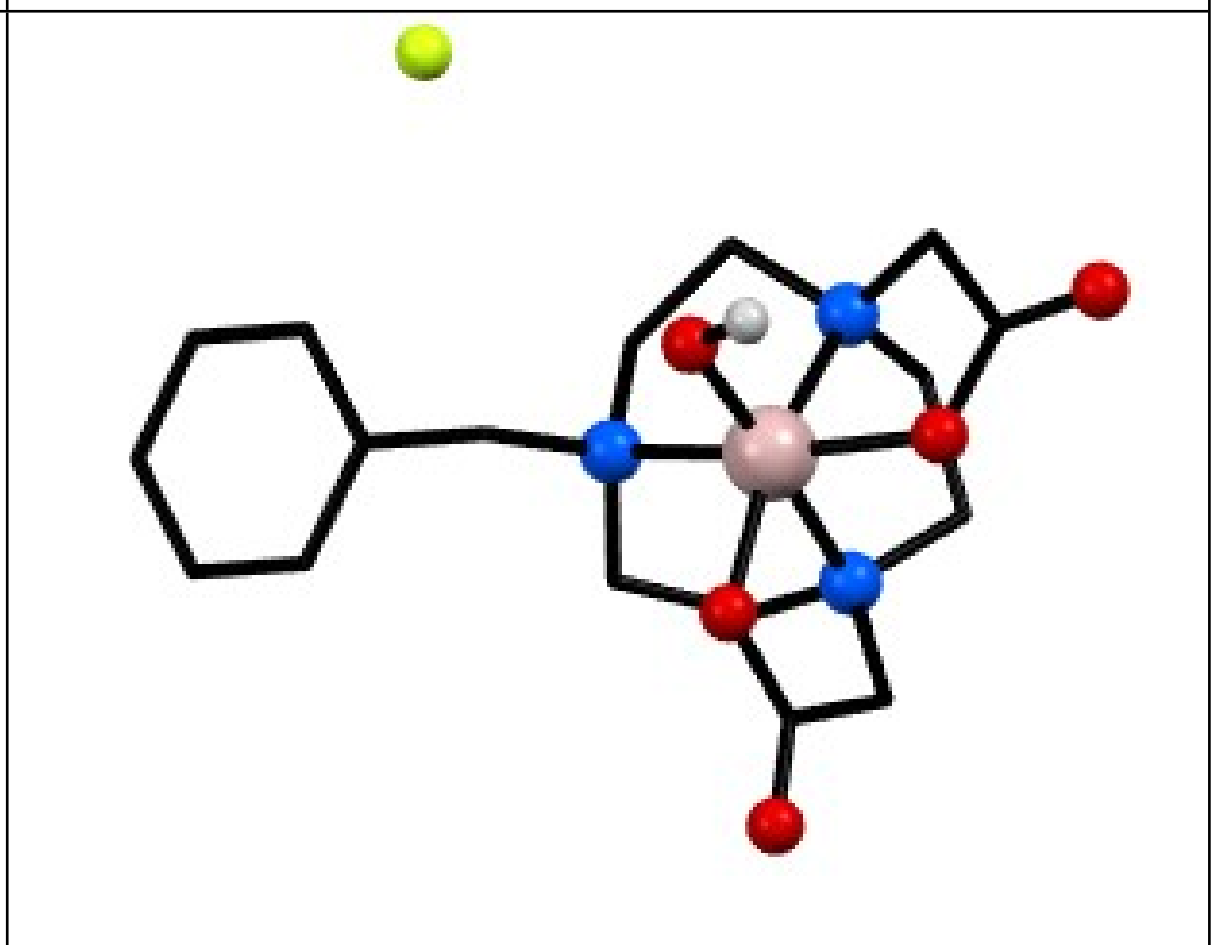
[Al(OH)(L)]	
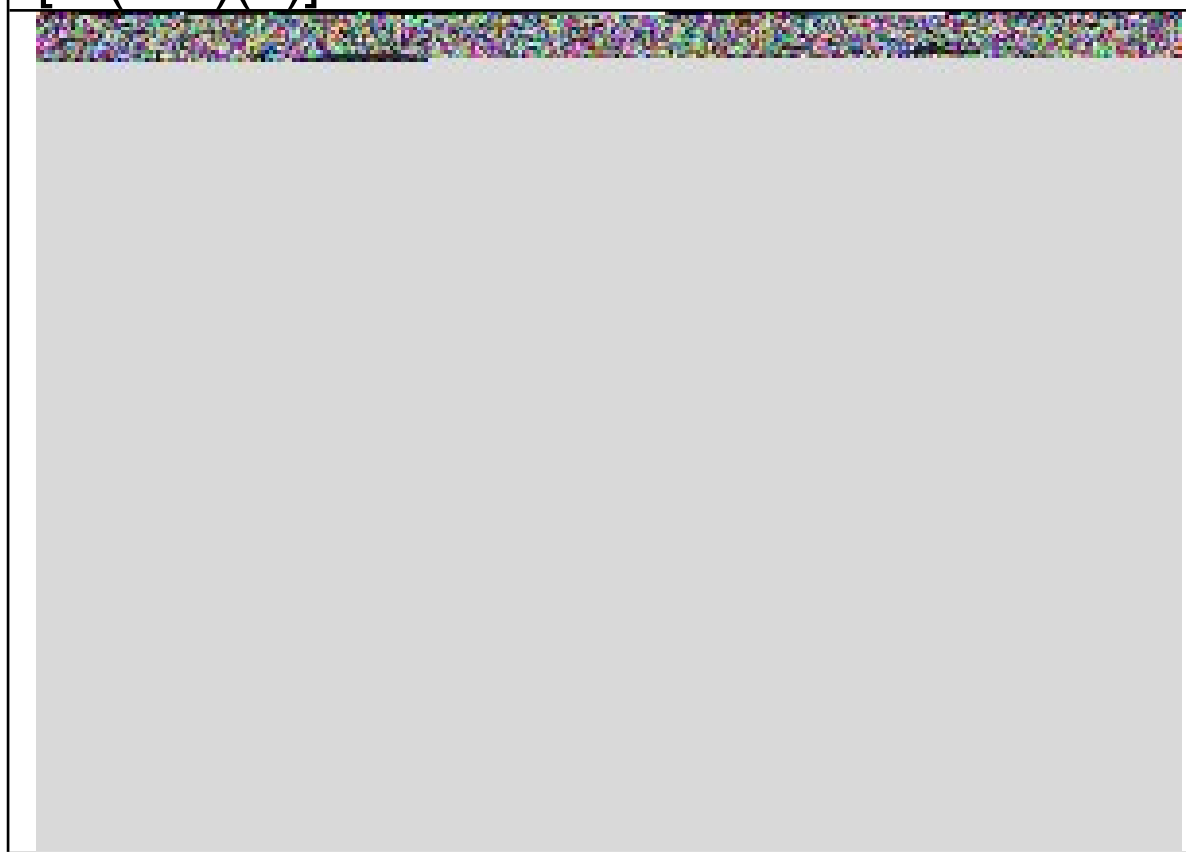	

[AlF(L)]	RC
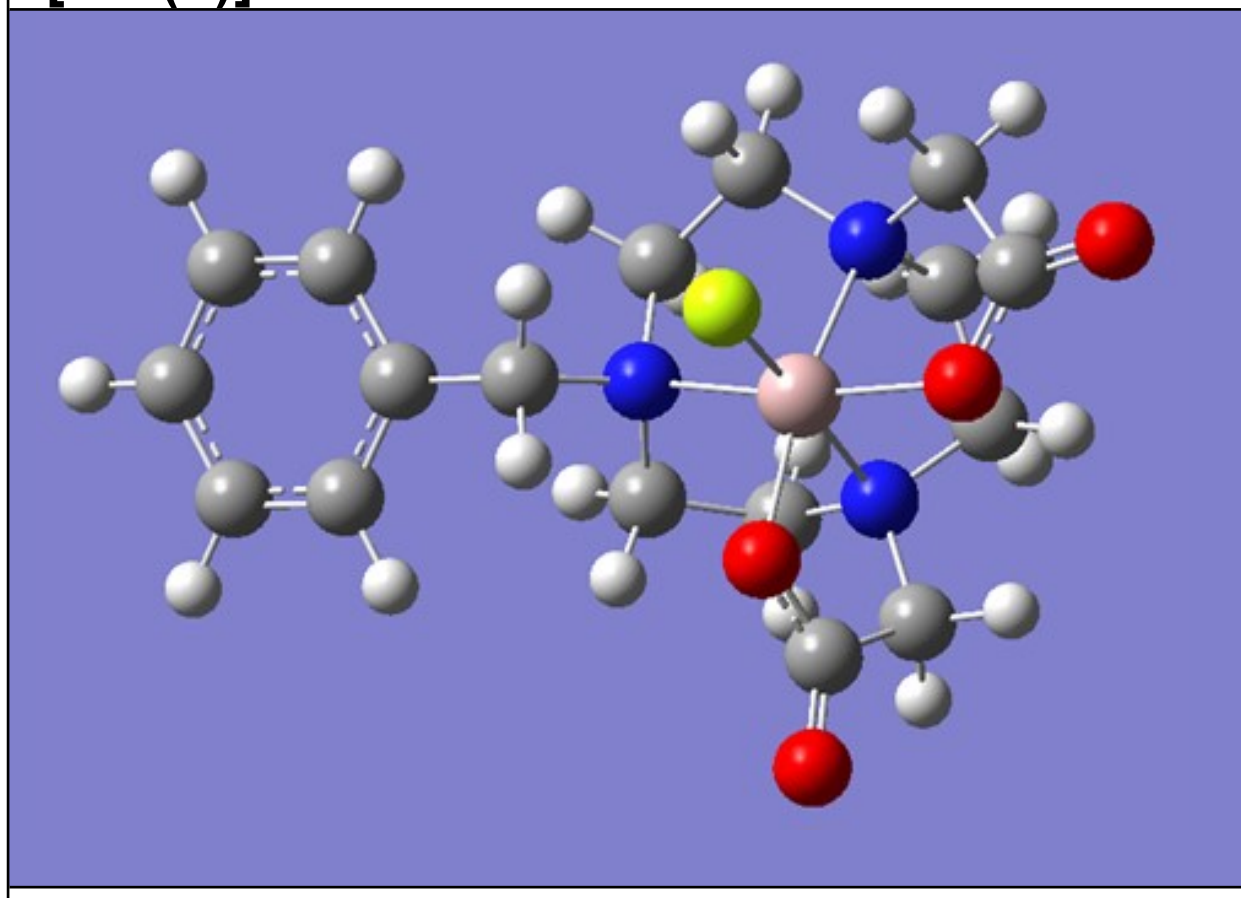	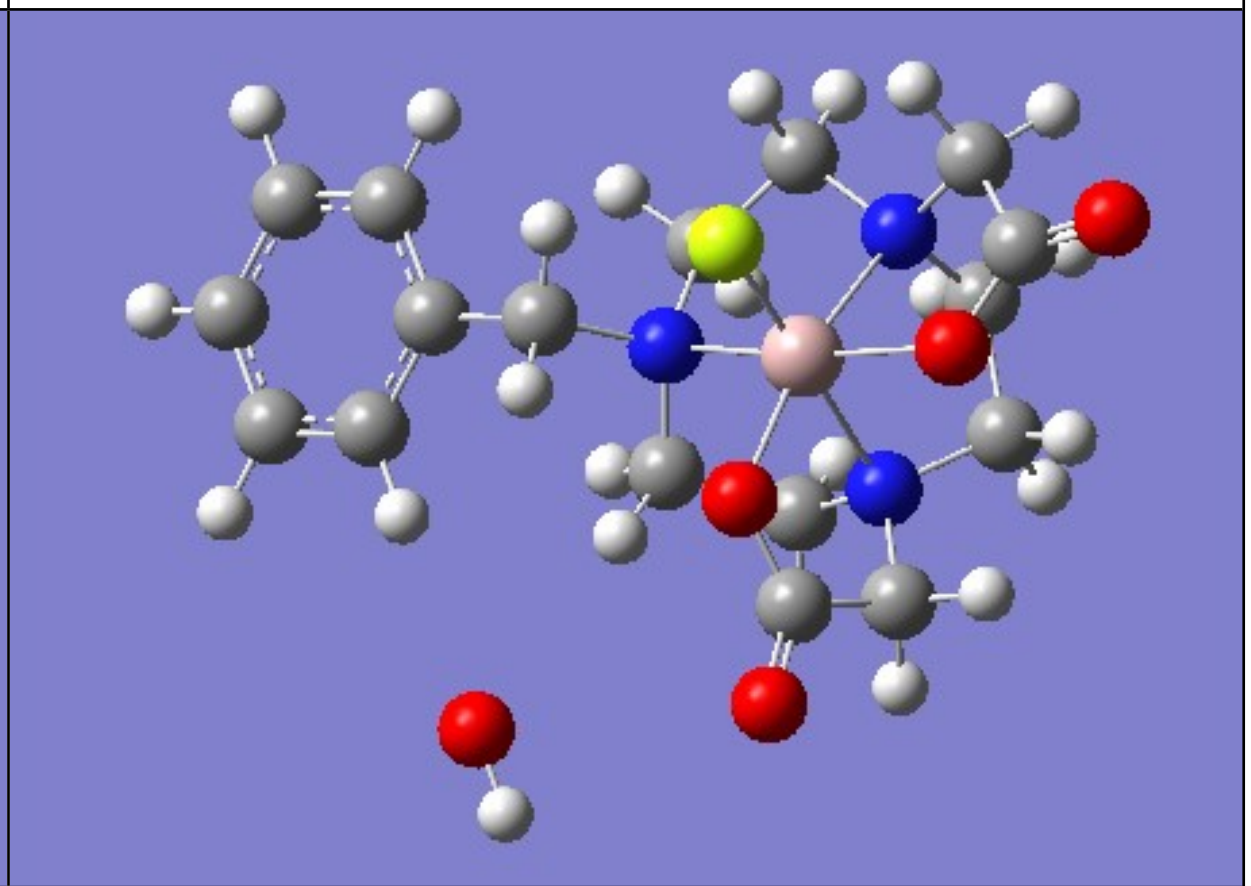
TS1	IC1
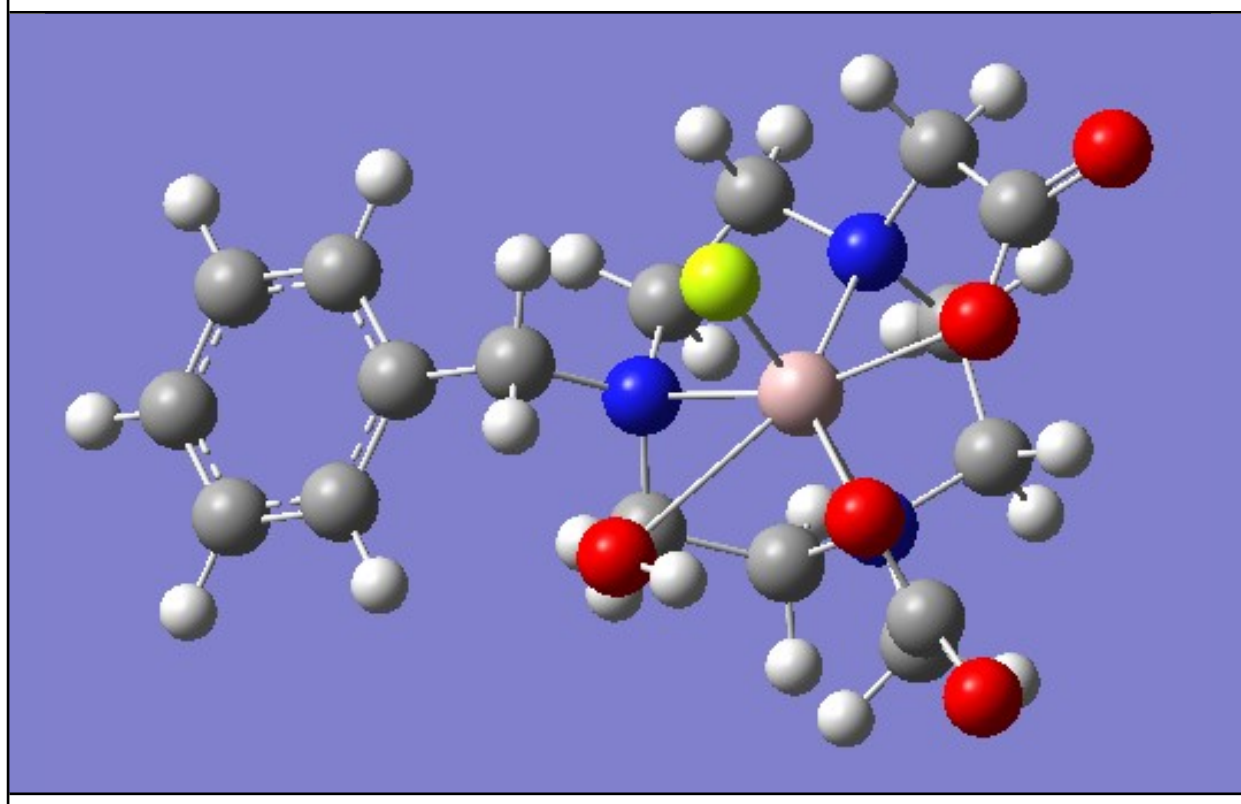	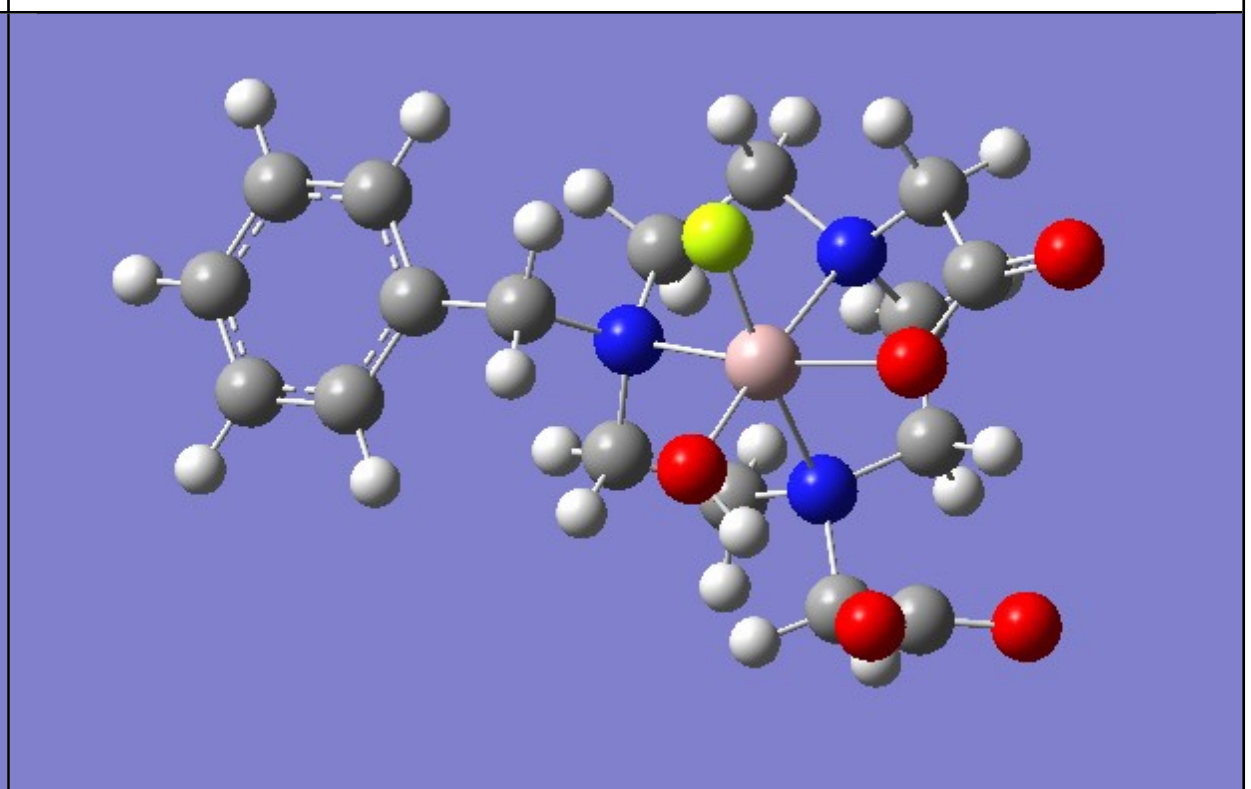
ICts	IC2
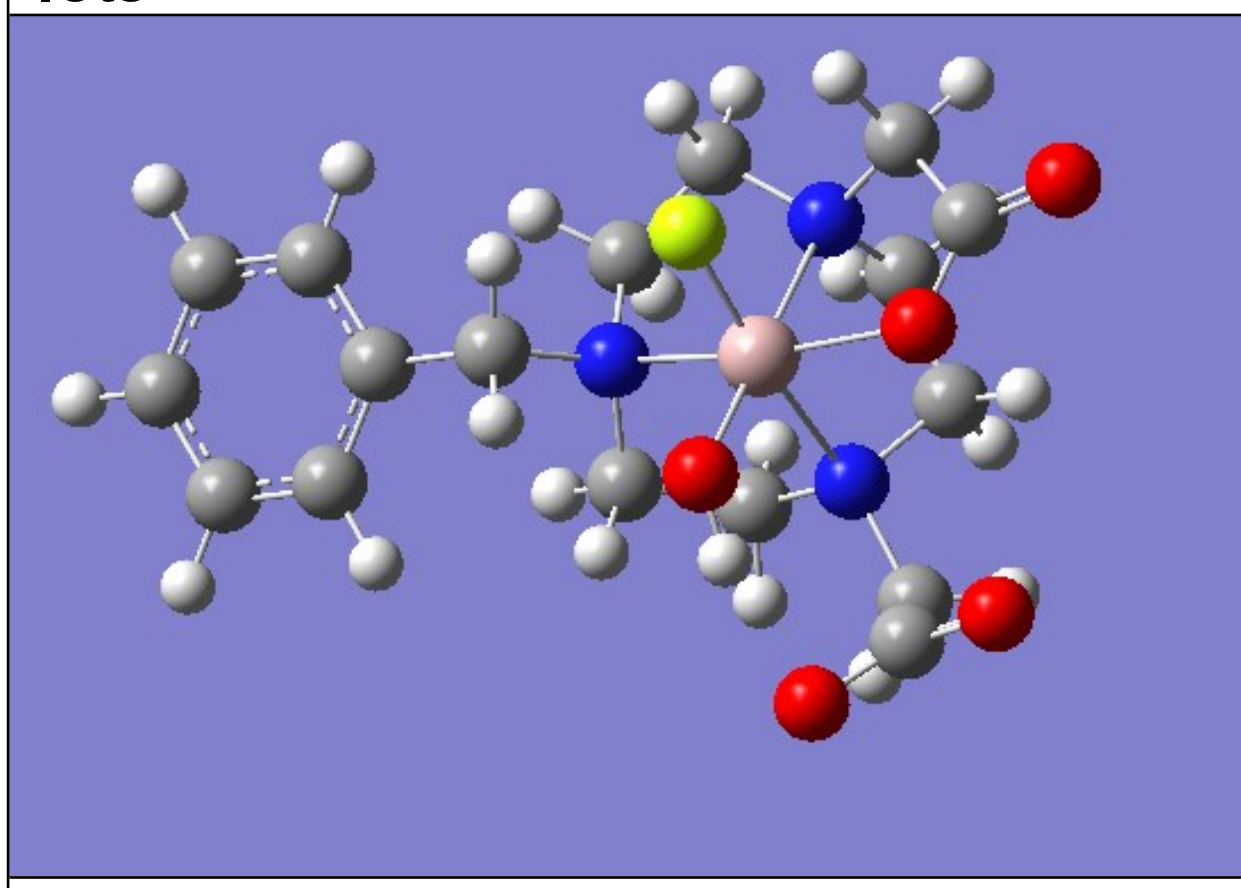	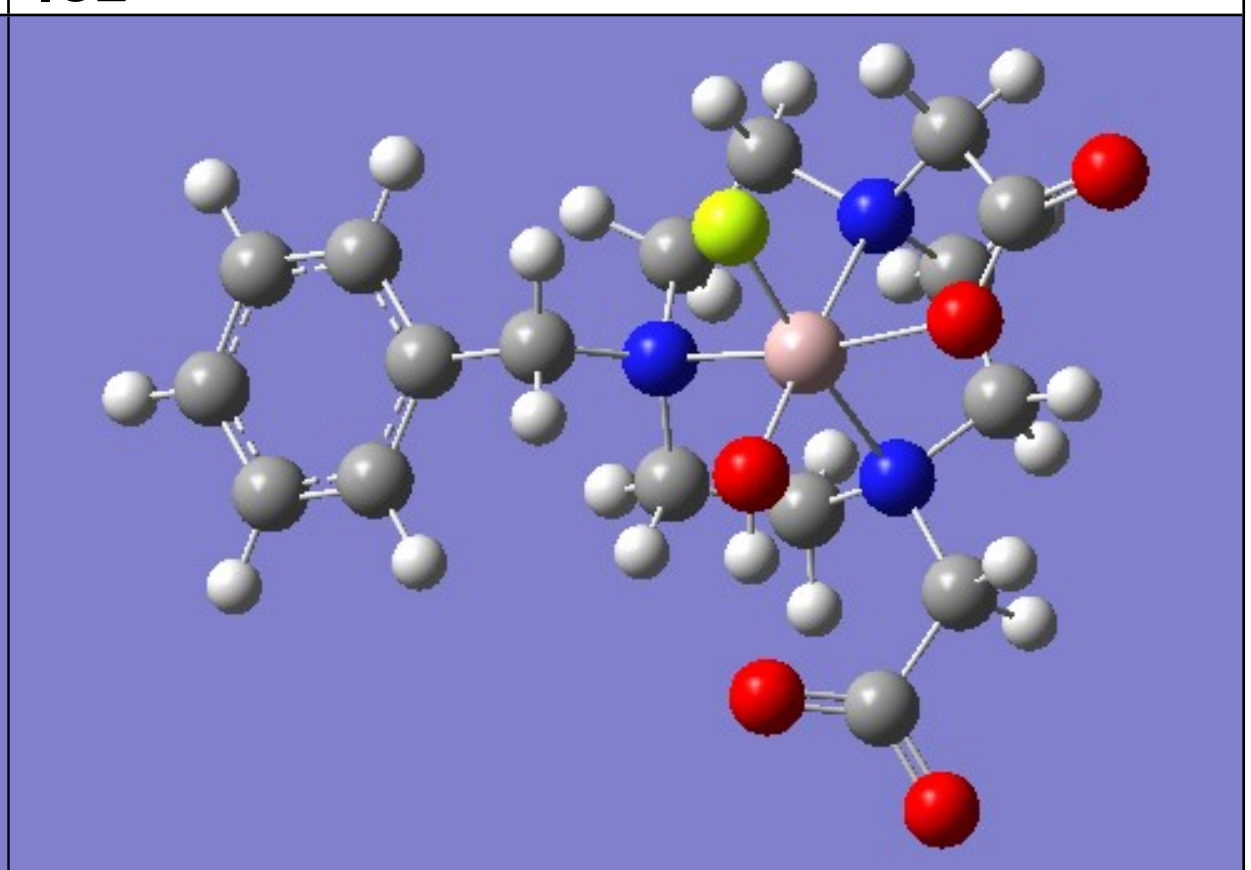
TS2	PC

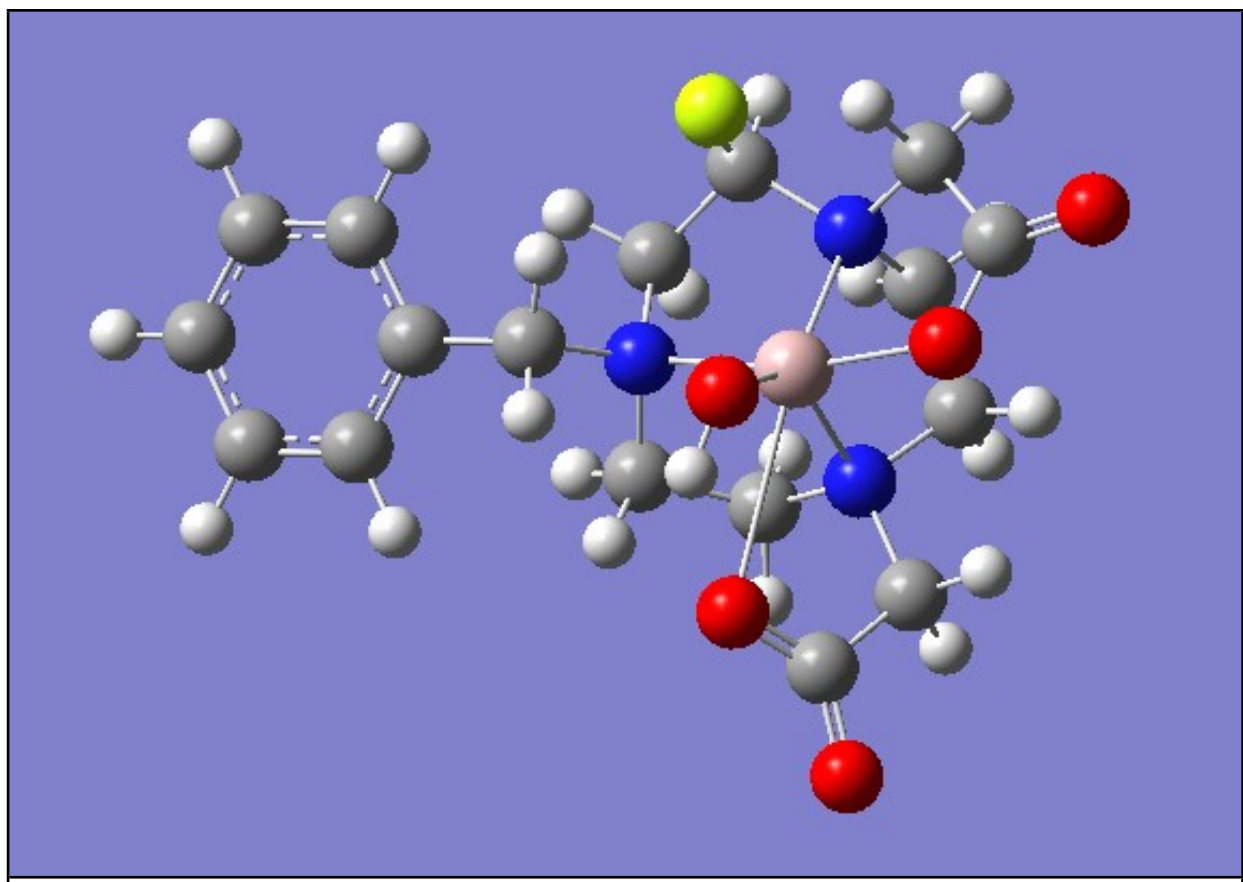

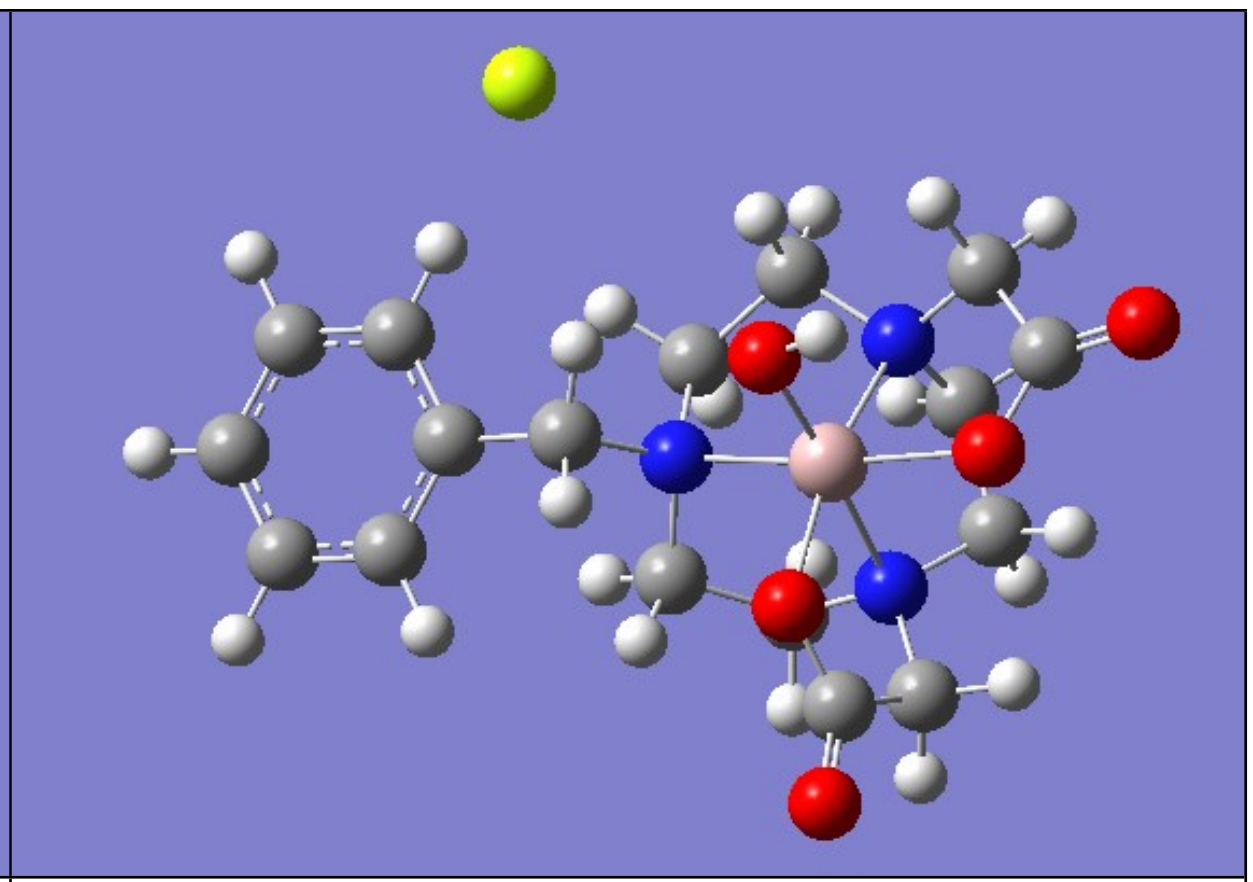

[Al(OH)(L)]

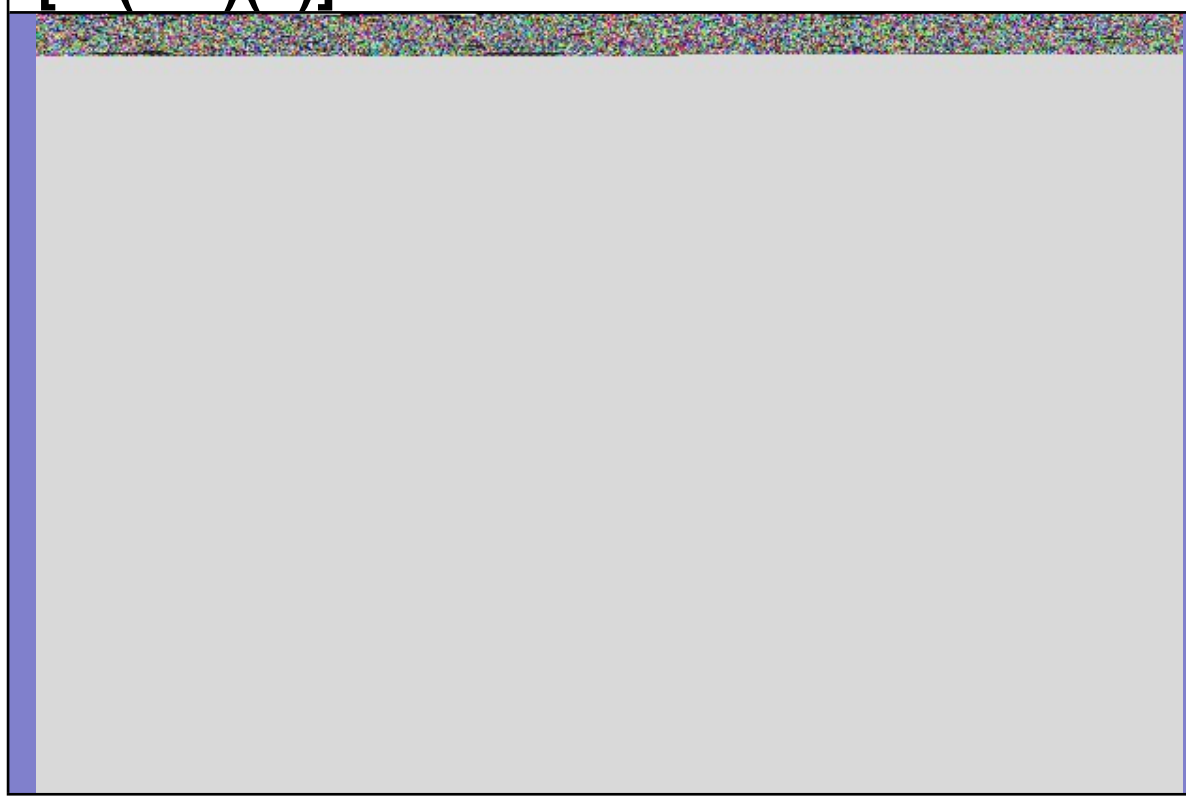

6c) Solution-phase calculations: Charge Density Table (Mulliken Analysis)

Mulliken charges of [AlF(L)] + OH^- geometries around Al centre. In solution/6-311++G(d,p)

	[AlF(L)	RC	TS1	IC1	ICTS	IC2	TS2	PC	[Al(OH)(L)]
**Al**	1.42251	1.438199	1.657457	1.152342	1.333625	1.499751	-0.19966	1.660081	1.625694
**F**	--X--	-0.63073	-0.57488	-0.63381	-0.64878	-0.65511	-0.78089	-0.88771	--X--
**$O_{(OH)}$**	-0.63826	-1.17745	-1.09877	-1.03697	-1.06205	-1.07435	-0.72521	-1.05898	-1.06371
**$O_{(1)}$**	-0.63221	-0.62893	-0.56661	-0.54918	-0.57571	-0.61251	-0.34751	-0.6105	-0.61027
**$O_{(2)}$**	-0.63514	-0.59603	-0.43108	-0.44433	-0.4878	-0.49349	-0.52668	-0.58817	-0.58859
**$N_{(3)}$**	-0.07760	-0.00763	-0.22769	0.044535	0.07353	0.008295	0.255217	-0.03803	-0.03992
**$N_{(4)}$**	-0.26659	-0.26948	-0.17751	-0.06863	-0.03074	-0.06811	0.02188	-0.17634	-0.18222
**$N_{(13)}$**	-0.15275	-0.08953	0.157242	-0.05502	-0.11146	-0.13736	-0.00018	-0.10574	-0.13903

Atom numbers for N atoms and carboxylate atoms around Al vary slightly between geometries, reported values are the equivalent positions, with the carboxylate oxygens being the closest to the metal centre.

7. Comparison of the geometries between [MX(L)] systems

7a) RC geometries

**RC**		
[GaCl(Bn-NODA) – [GaF(Bn-NODA)]	[GaCl(Bn-NODA)] – [Ga(OH)(Bn-NODA)]	[GaF(Bn-NODA)] – [Ga(OH)(Bn-NODA)]
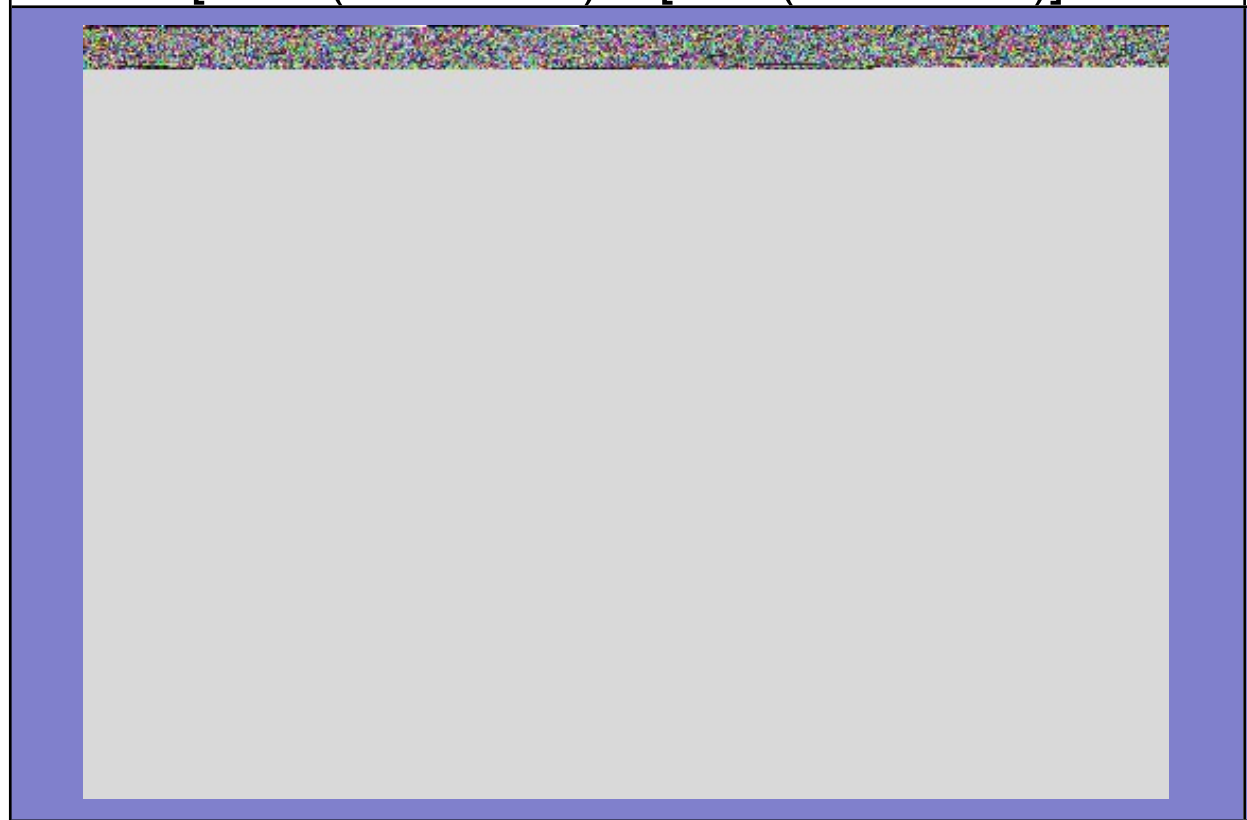	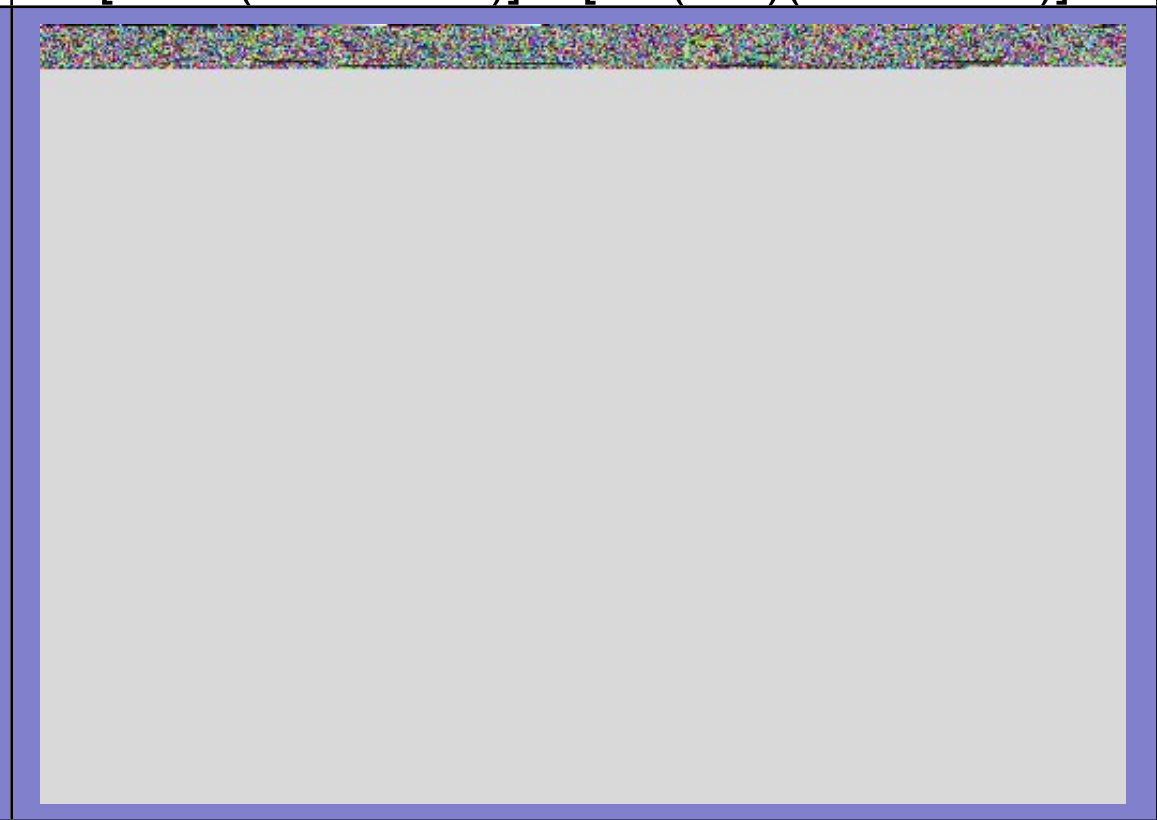	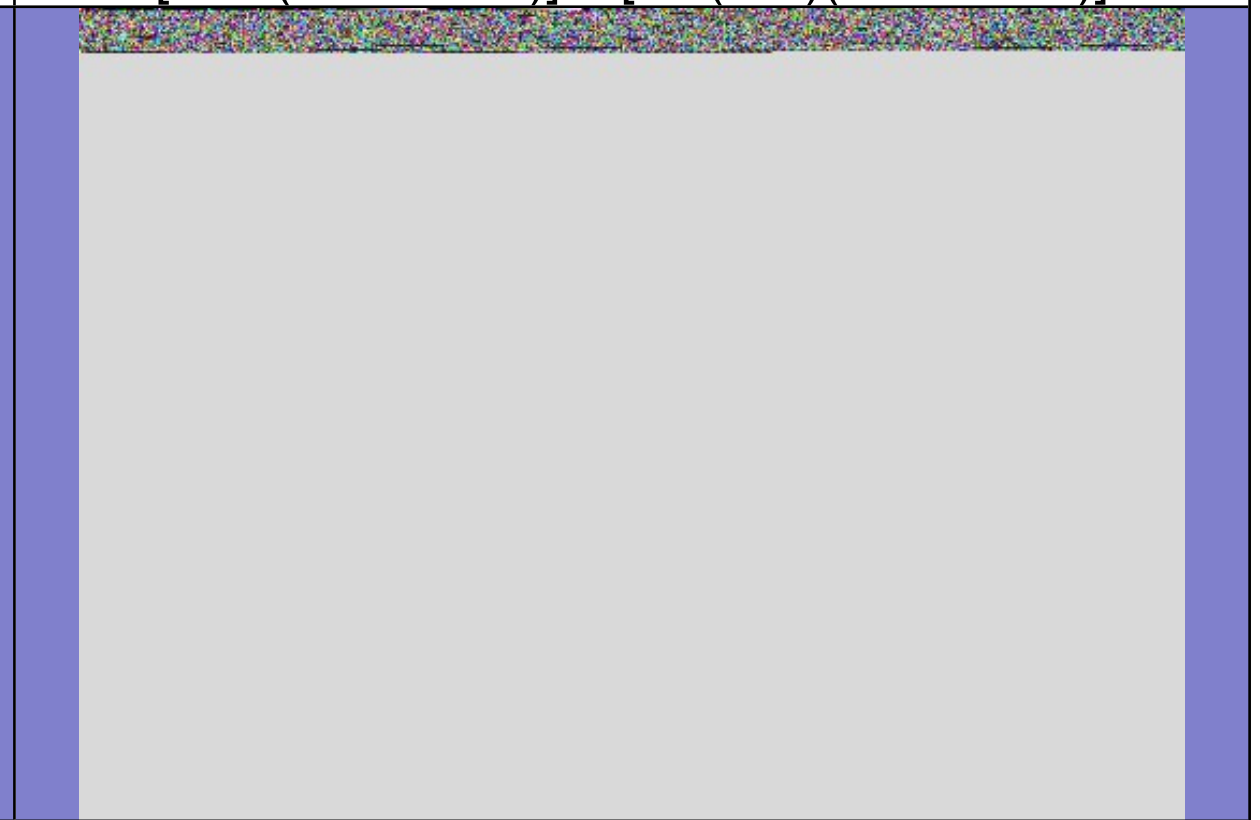
[AlCl(Bn-NODA)] – [AlF(Bn-NODA)]	[AlCl(Bn-NODA)] – [AlOH(Bn-NODA)]	[AlF(Bn-NODA)] – [Al(OH)(Bn-NODA)]
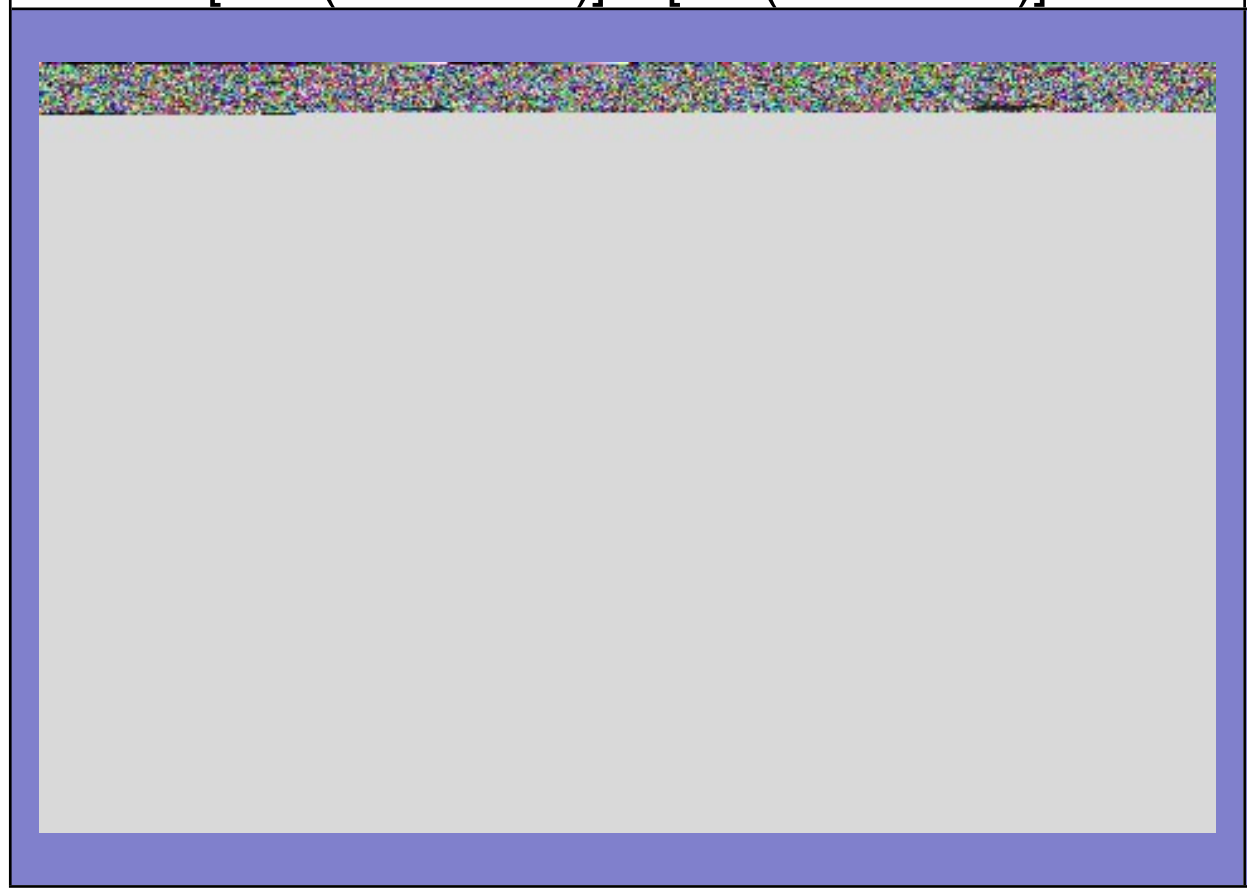	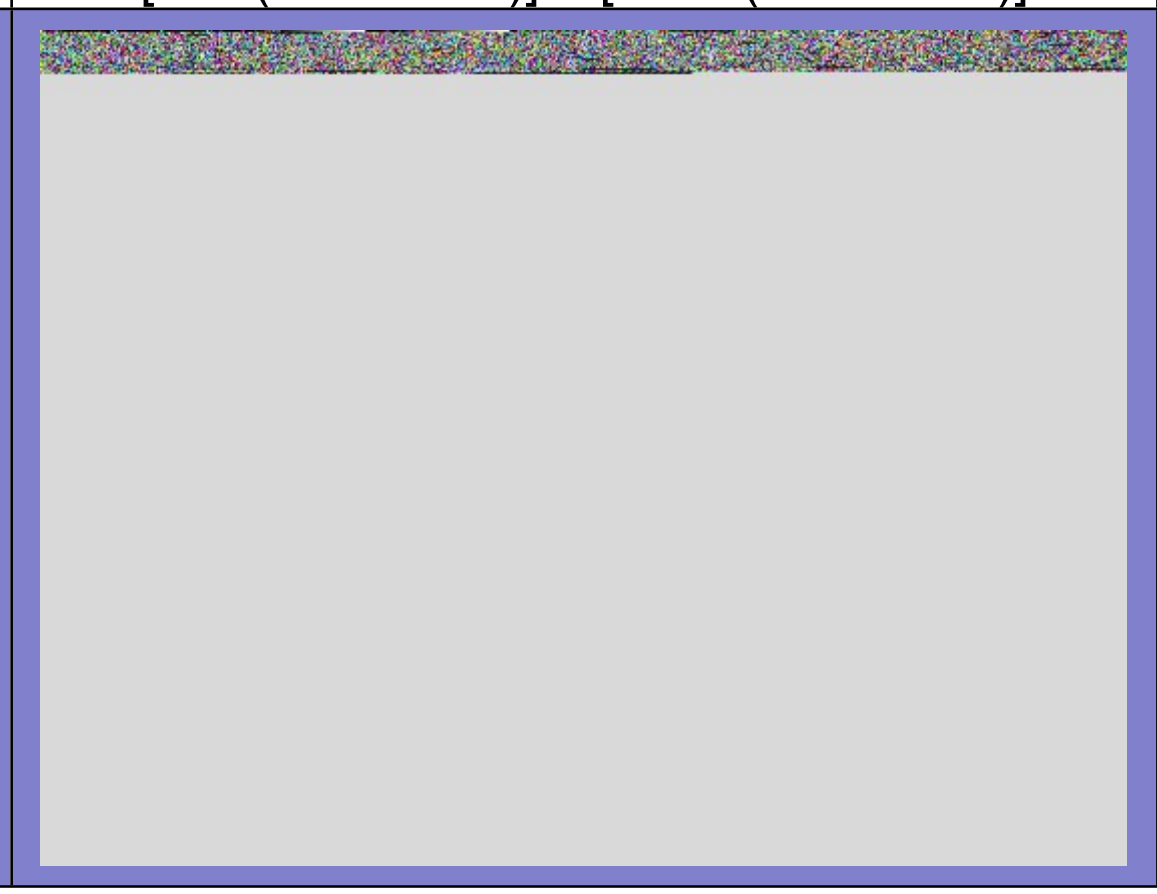	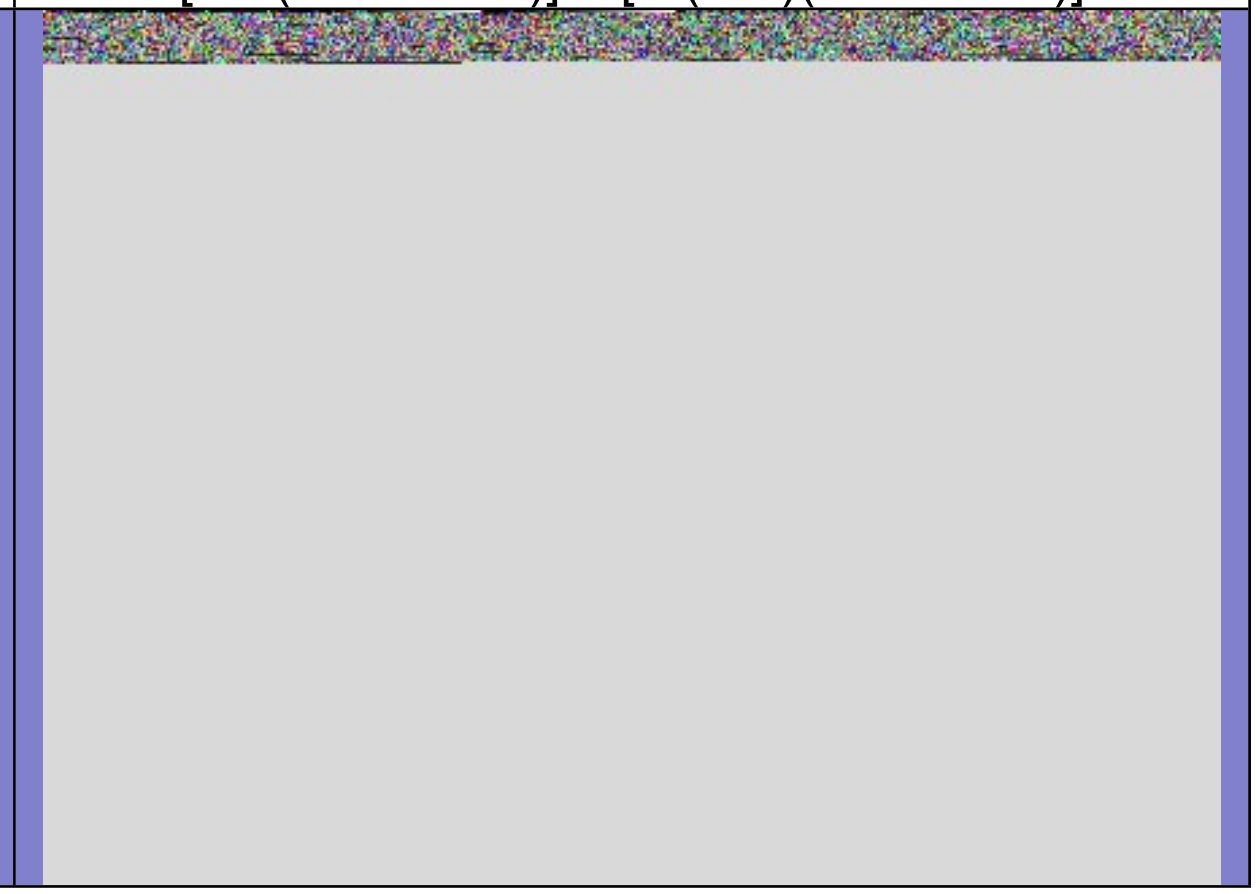

7b) TS1 geometries

TS1		
[GaCl(Bn-NODA)] – [GaF(Bn-NODA)]	[GaCl(Bn-NODA)] – [Ga(OH)(Bn-NODA)]	[GaF(Bn-NODA)] – [Ga(OH)(Bn-NODA)]
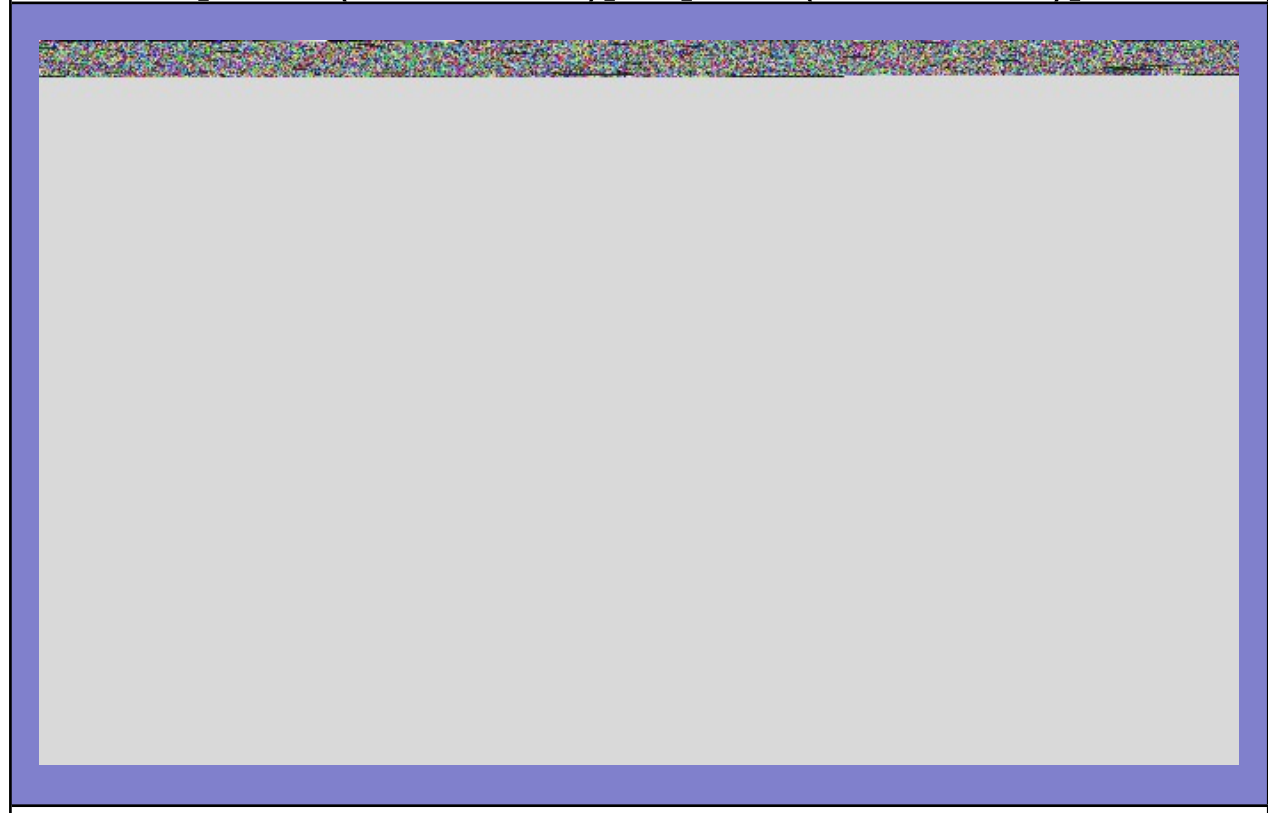	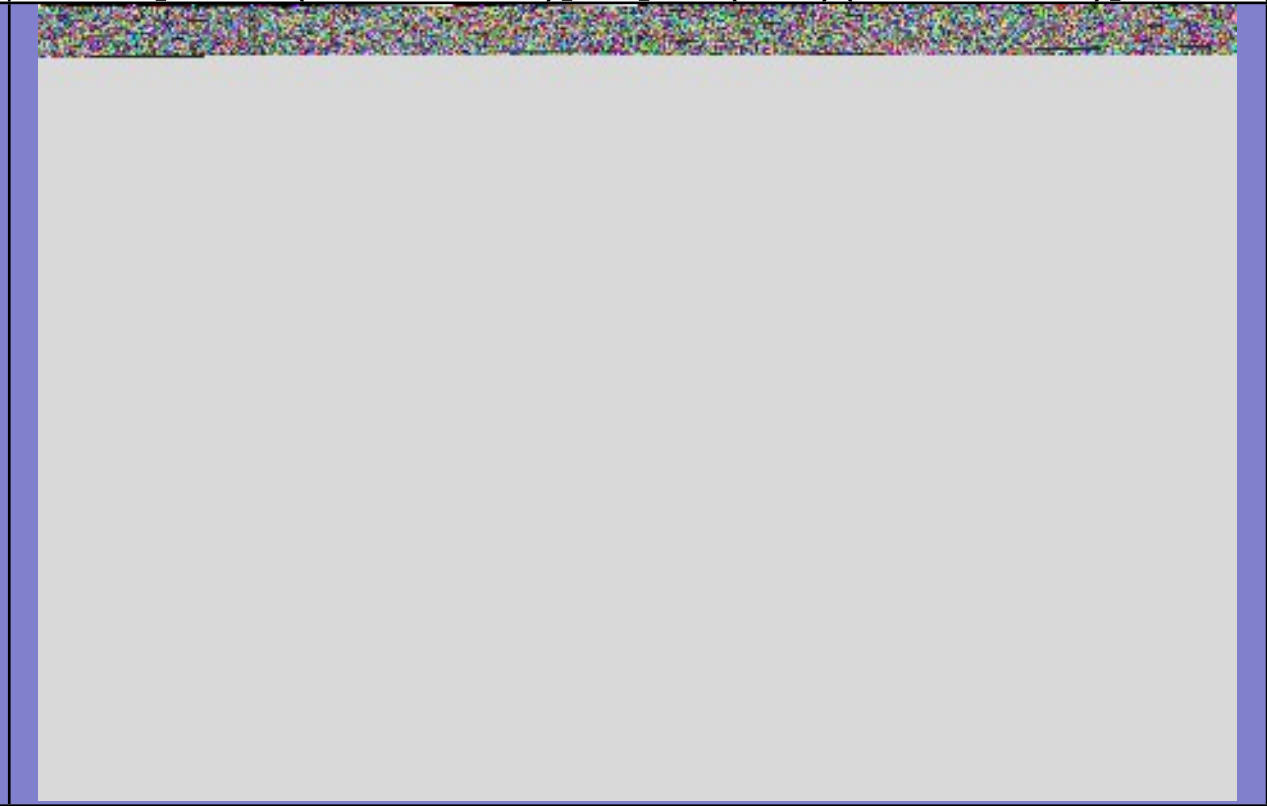	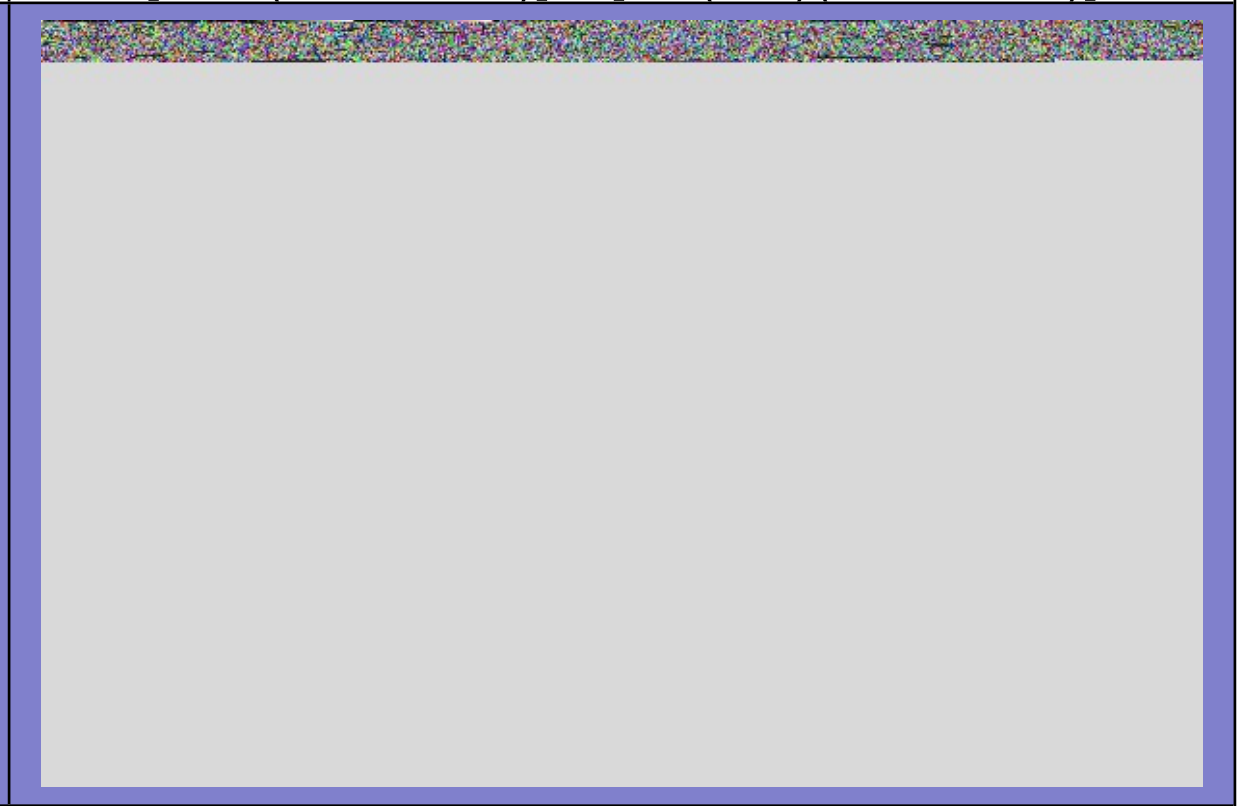
[AlCl(Bn-NODA)] – [AlF(Bn-NODA)]	[AlCl(Bn-NODA)] – [Al(OH)(Bn-NODA)]	[AlF(Bn-NODA)] – [Al(OH)(Bn-NODA)]
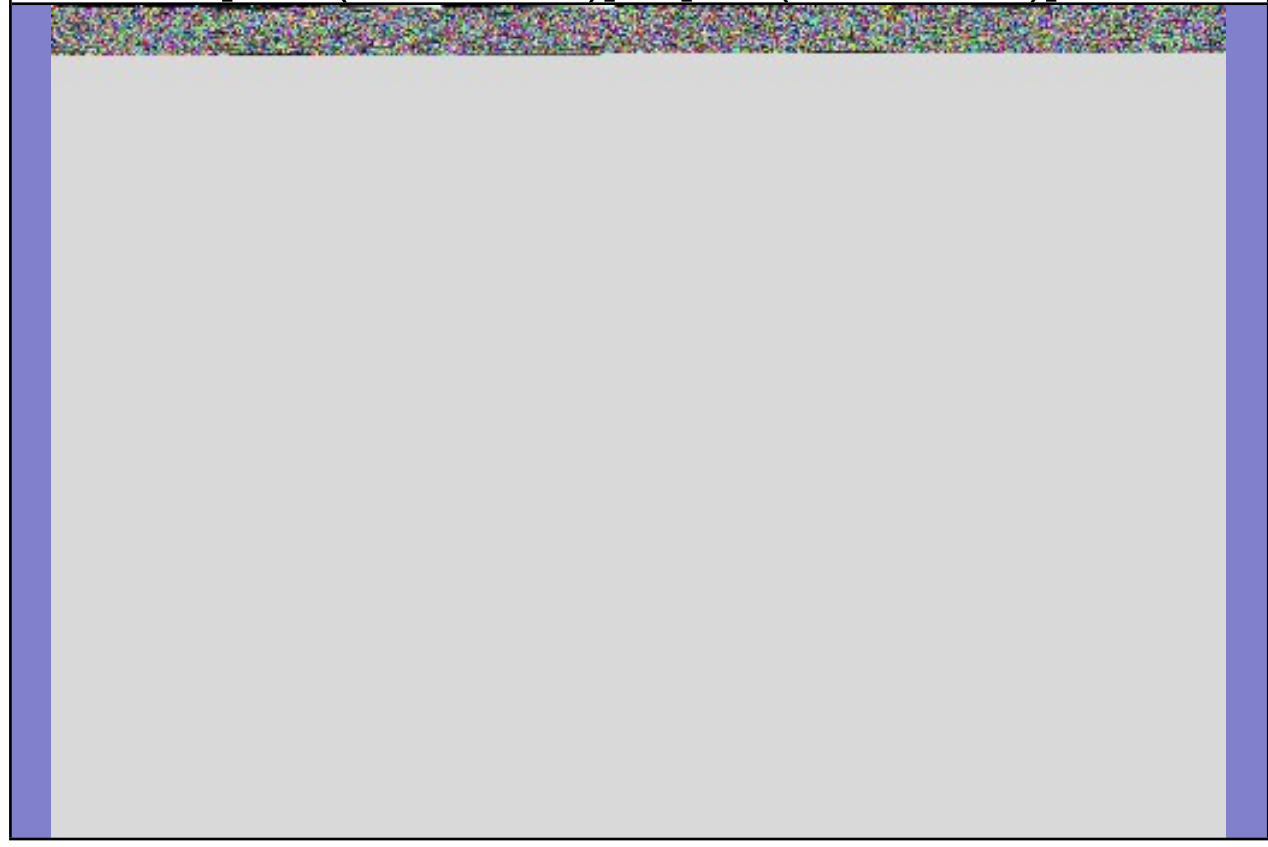	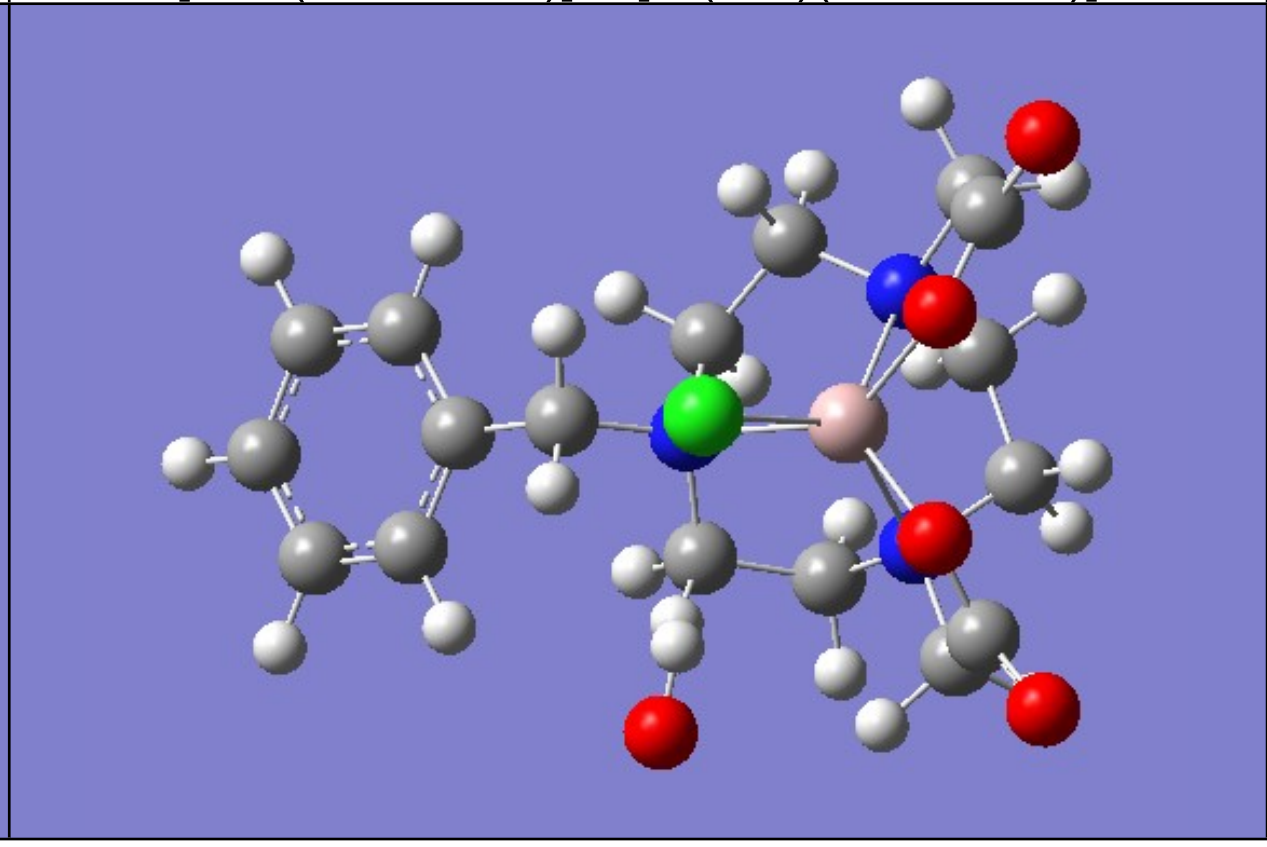	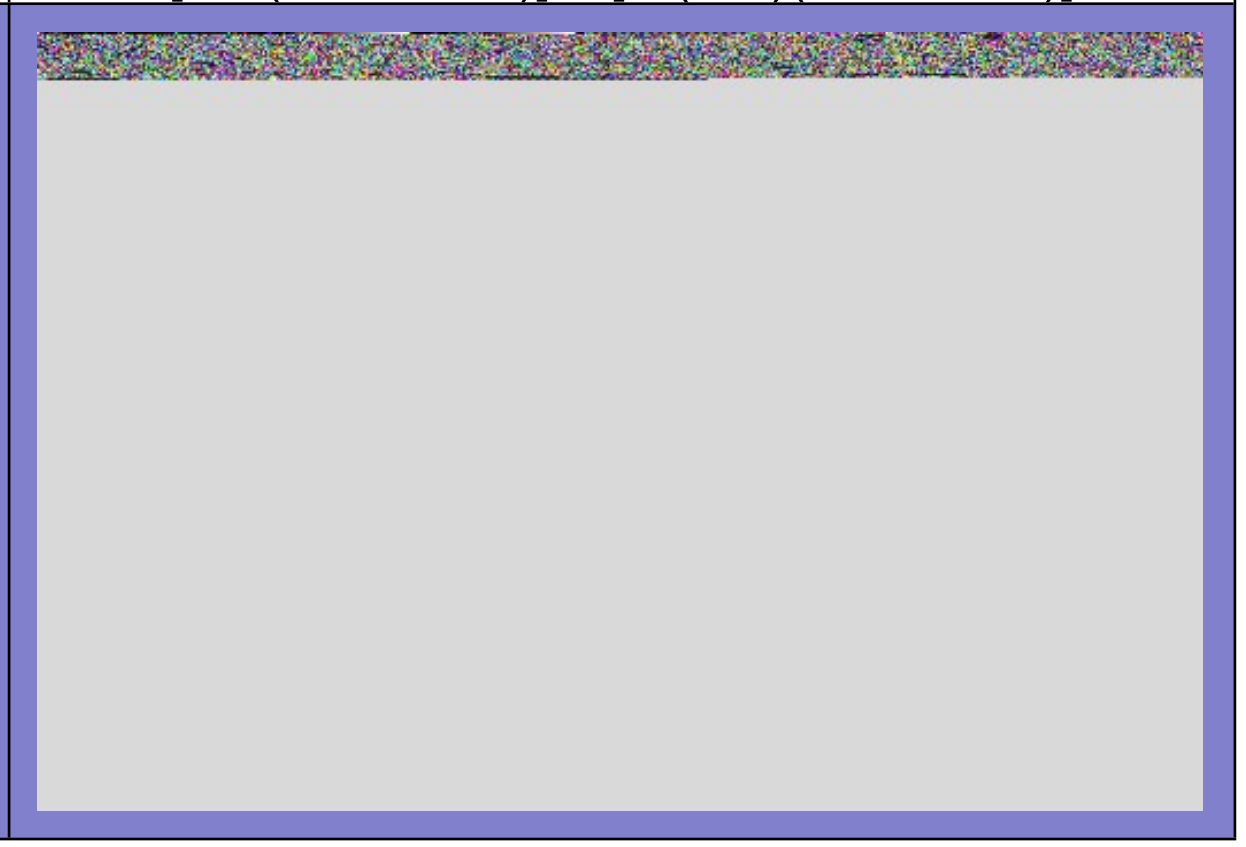

7c) IC1 geometries

IC1		
[GaCl(Bn-NODA)] – [GaF(Bn-NODA)]	[GaCl(Bn-NODA)] – [Ga(OH)(Bn-NODA)]	[GaF(Bn-NODA)] – [Ga(OH)(Bn-NODA)]
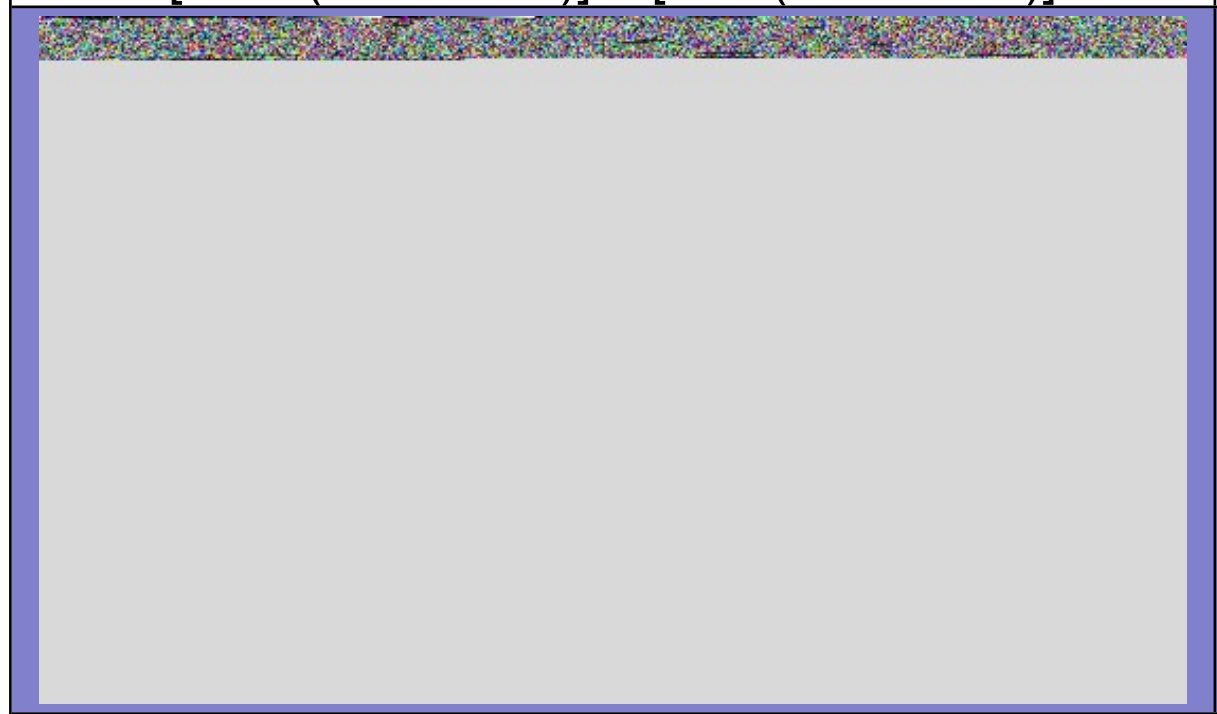	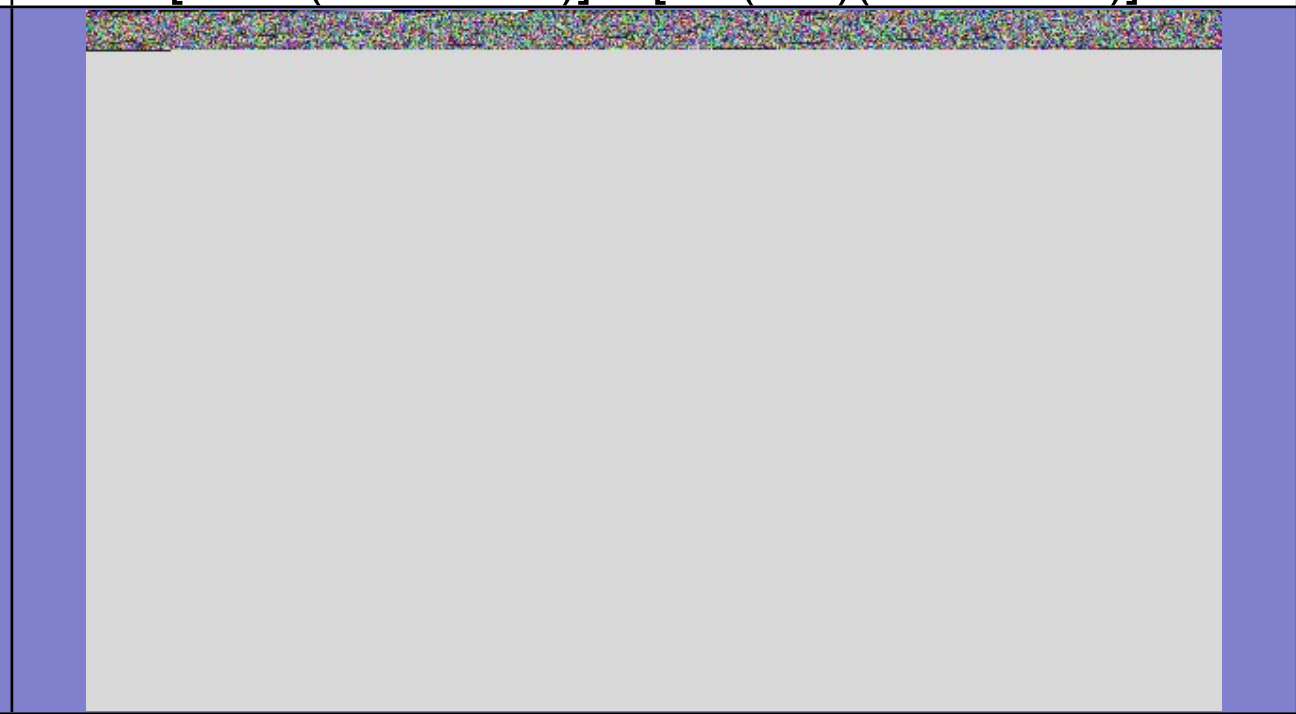	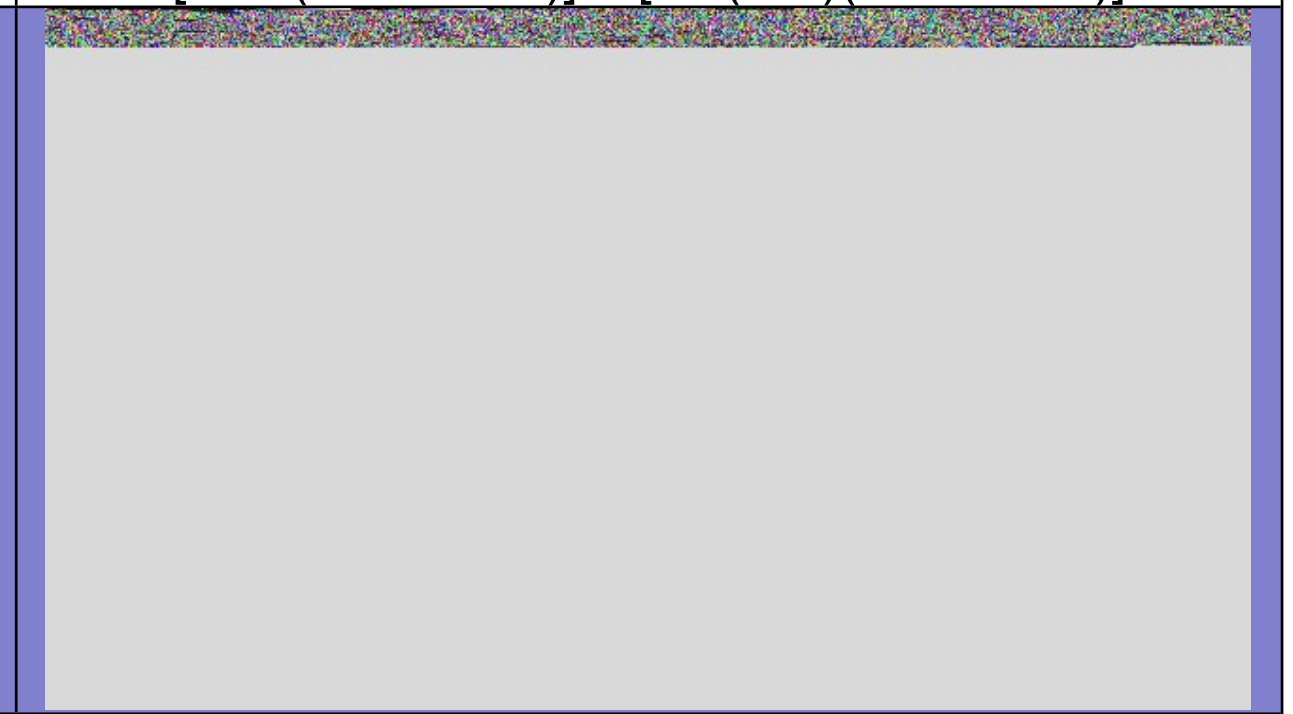
[AlCl(Bn-NODA)] – [AlF(Bn-NODA)	[AlCl(Bn-NODA)] – [Al(OH)(Bn-NODA)]	[AlF(Bn-NODA)] – [Al(OH)(Bn-NODA)]
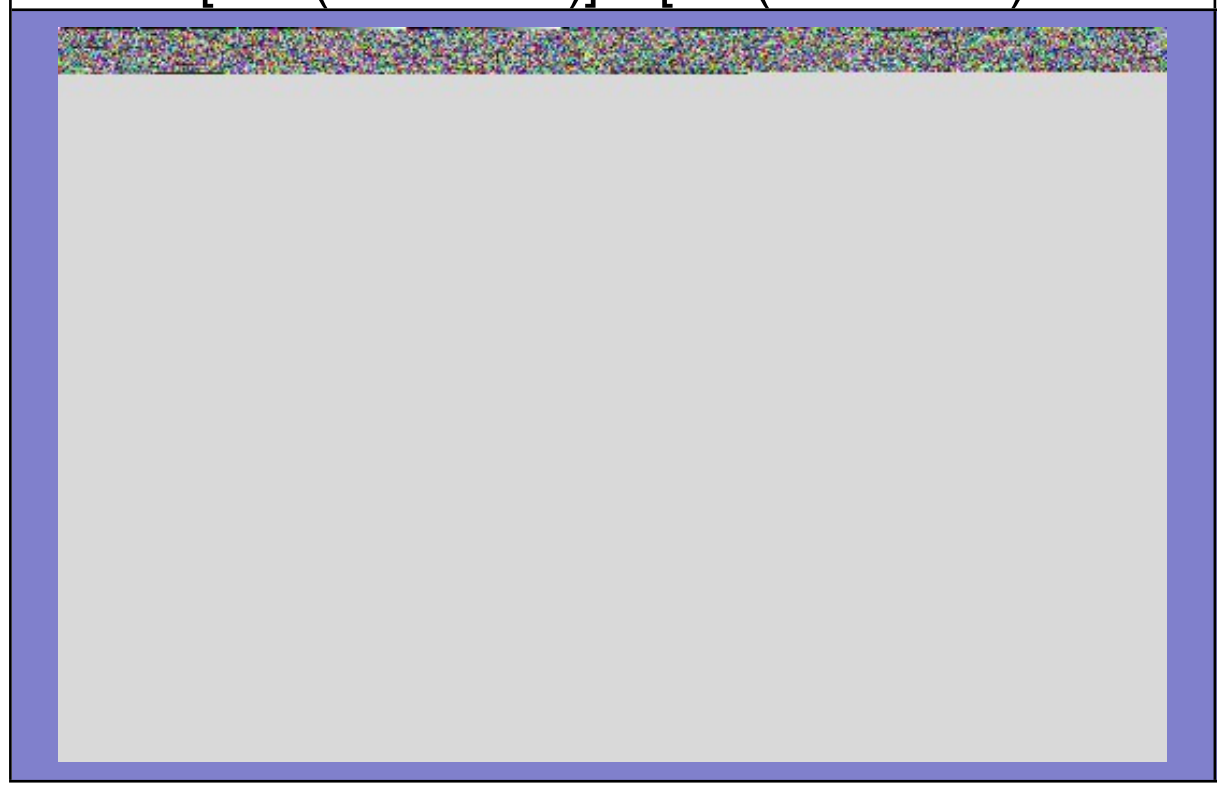	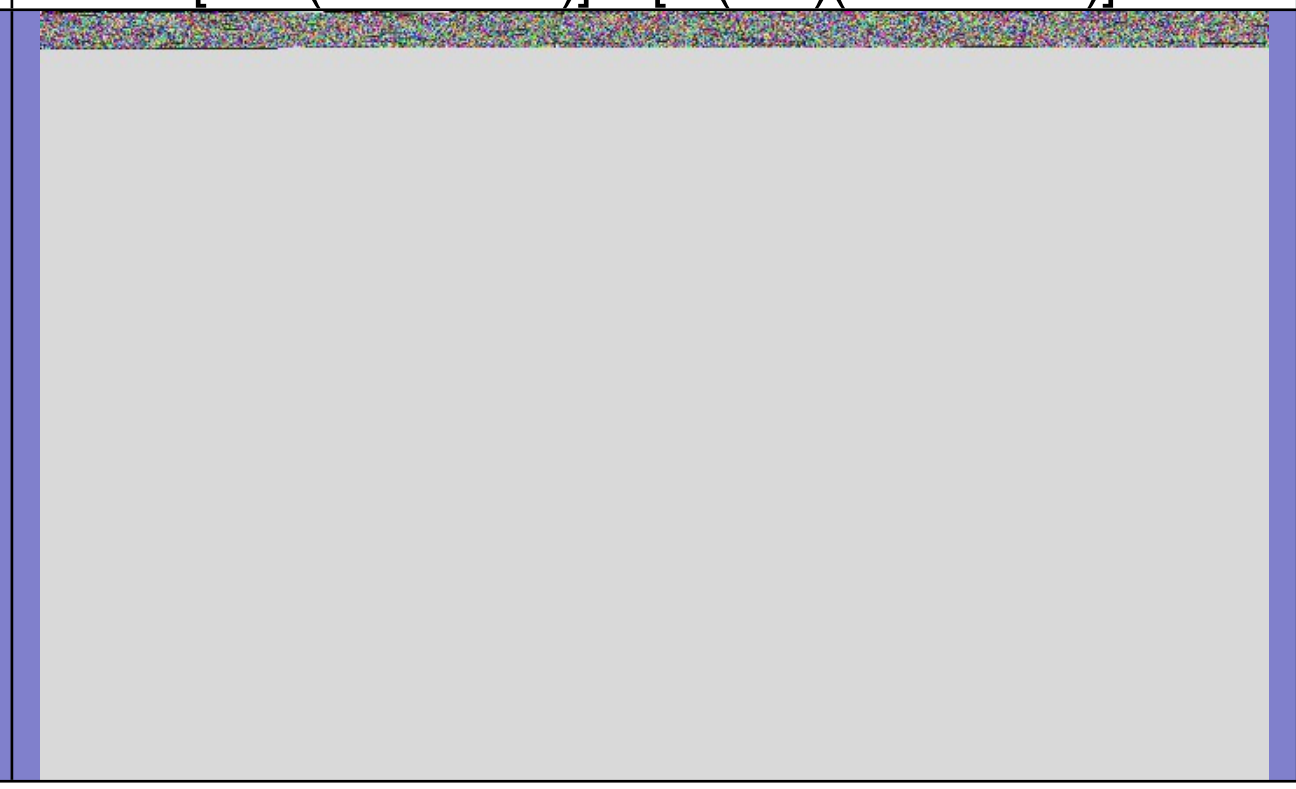	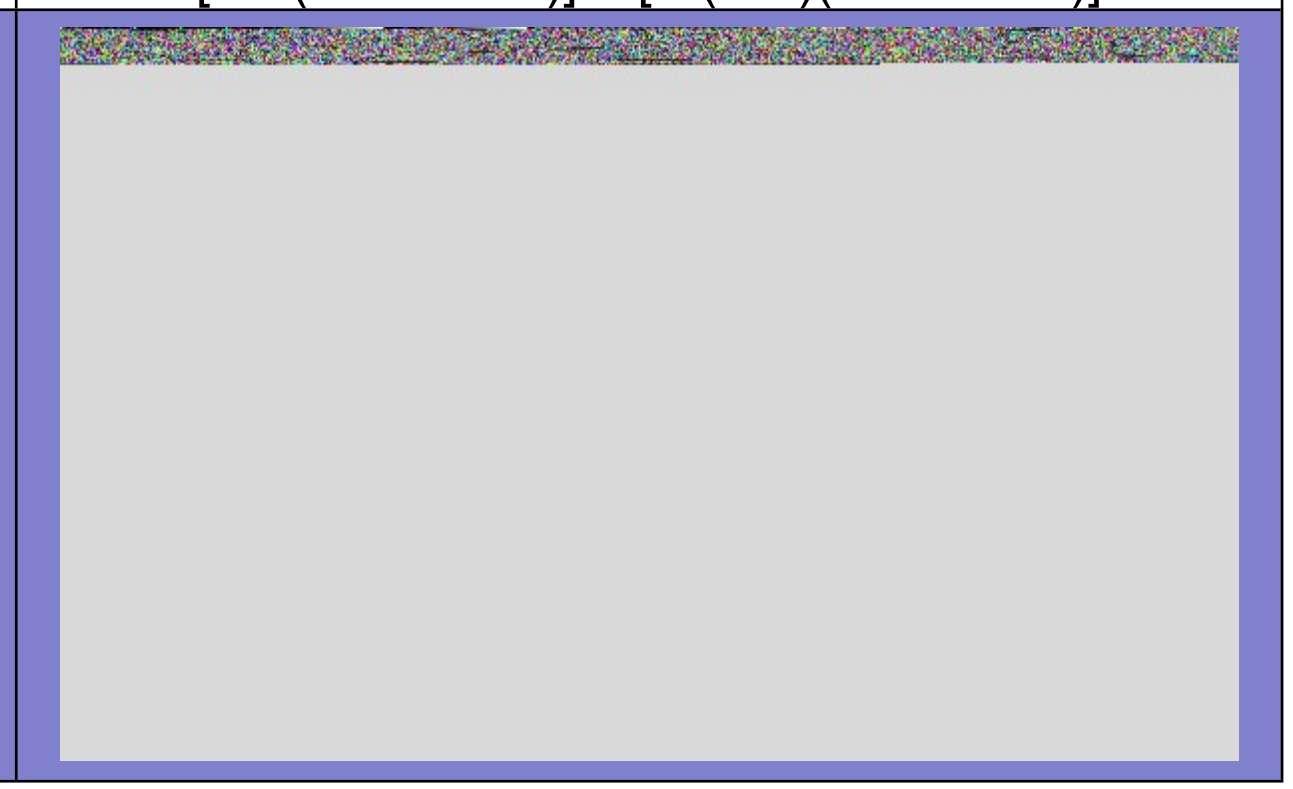

7d) ICts geometries

ICTS		
[GaCl(Bn-NODA)] – [GaF(Bn-NODA)]	[GaCl(Bn-NODA)] – [Ga(OH)(Bn-NODA)]	[GaF(Bn-NODA)] – [Ga(OH)(Bn-NODA)]
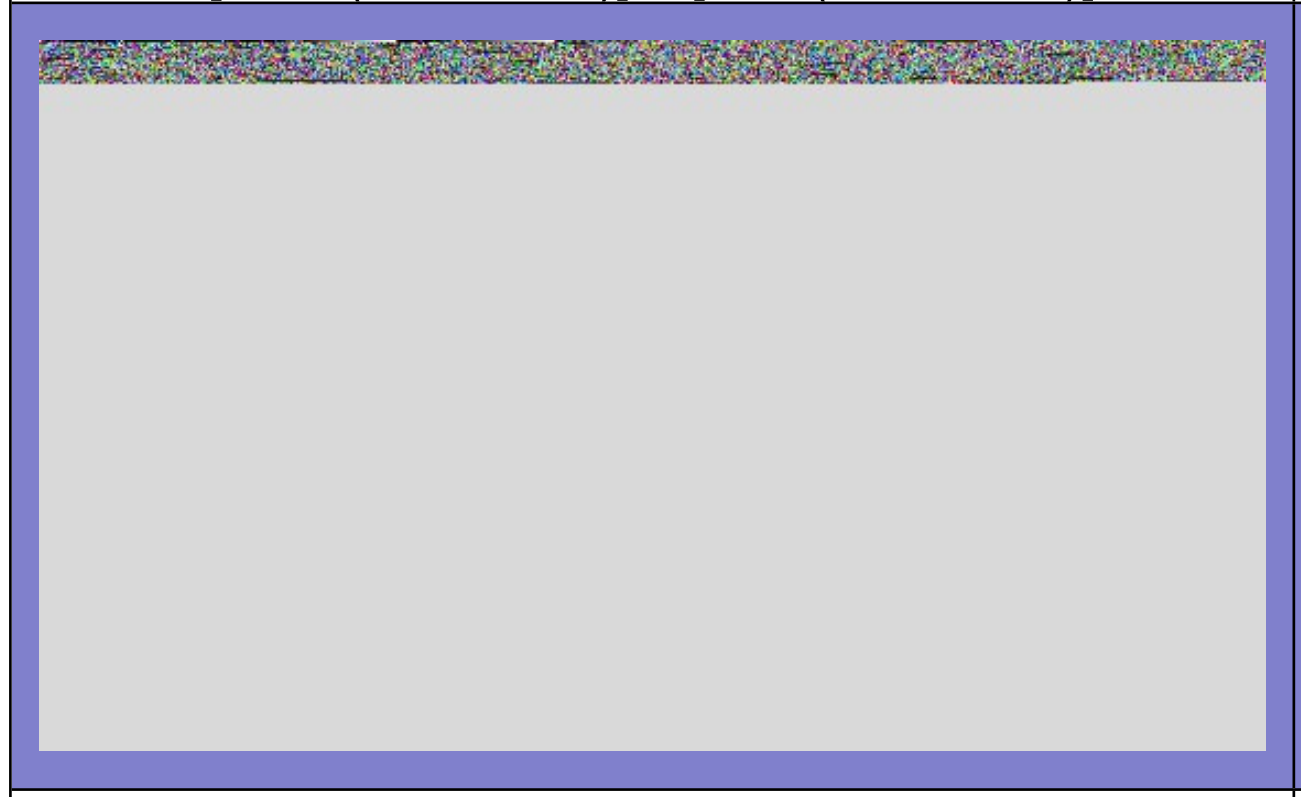	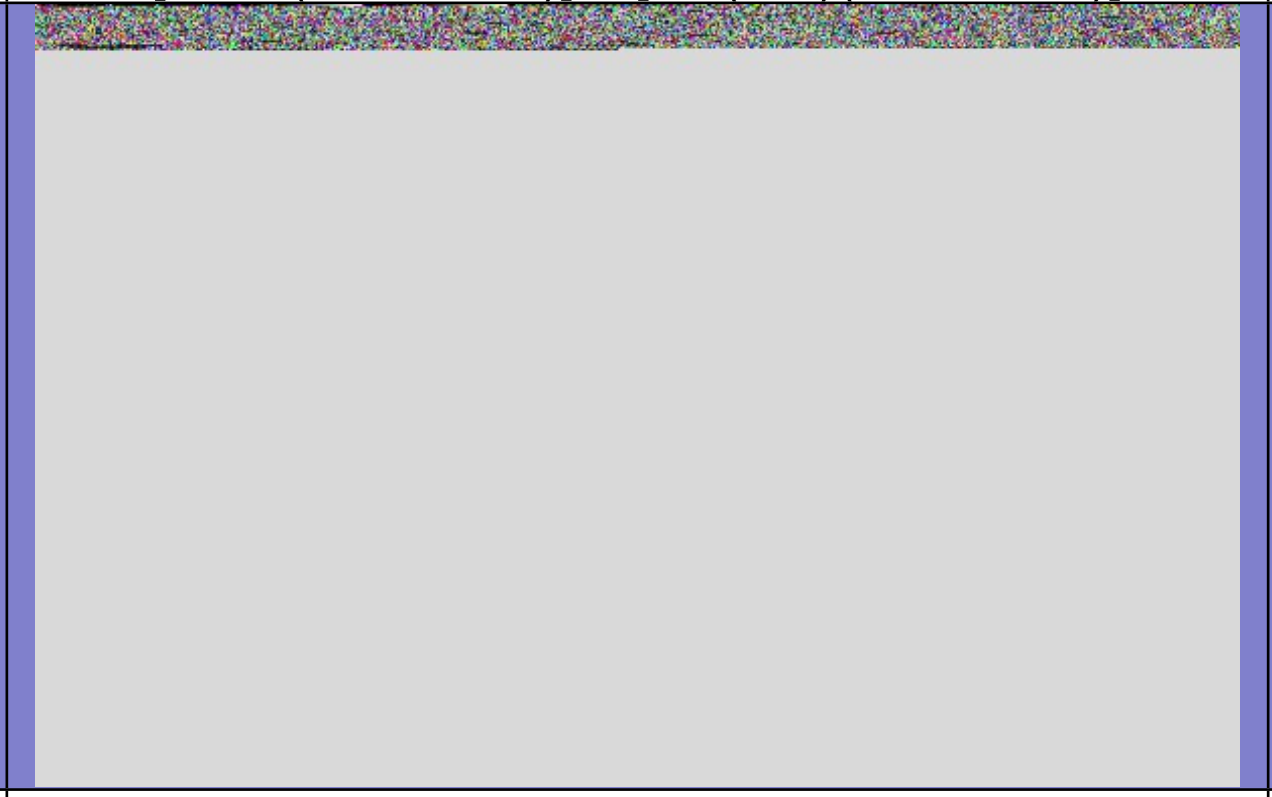	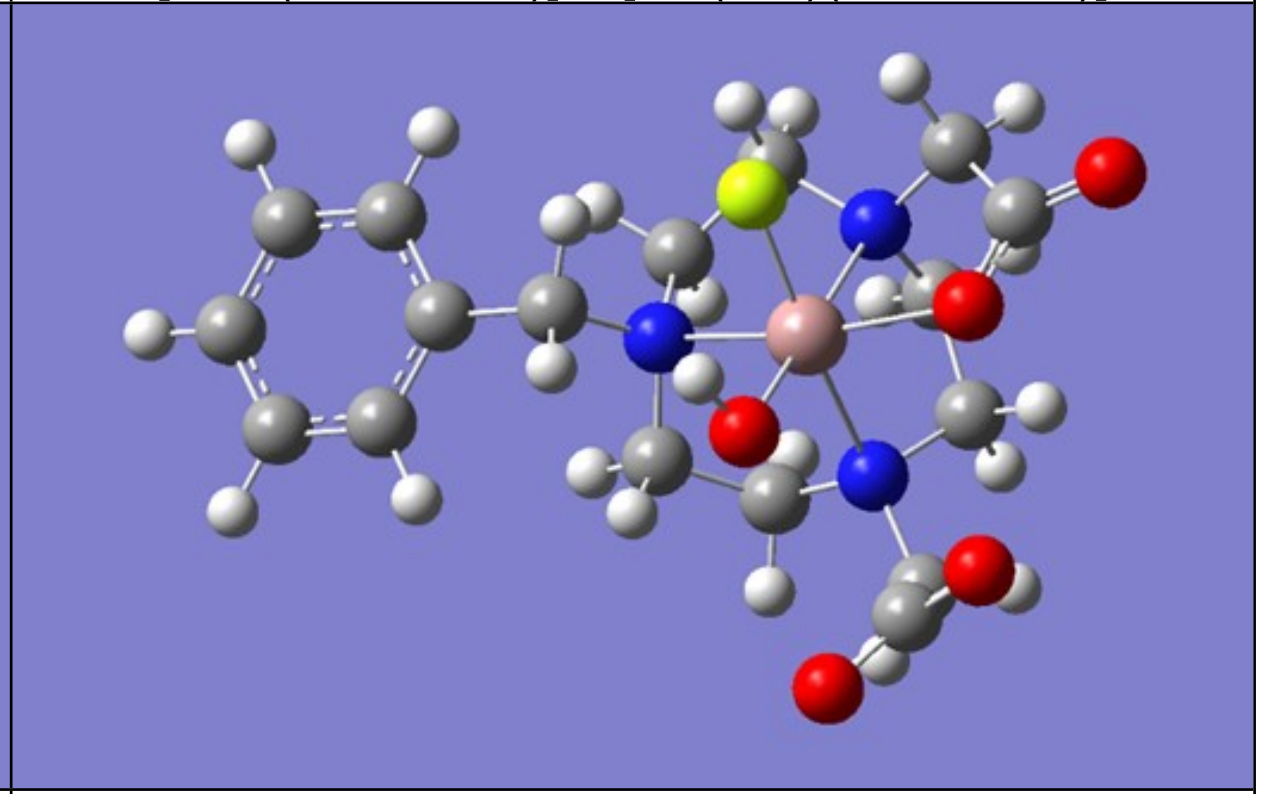
[AlCl(Bn-NODA)] – [AlF(Bn-NODA)]	[AlCl(Bn-NODA)] – [Al(OH)(Bn-NODA)]	[AlF(Bn-NODA)] – [Al(OH)(Bn-NODA)]
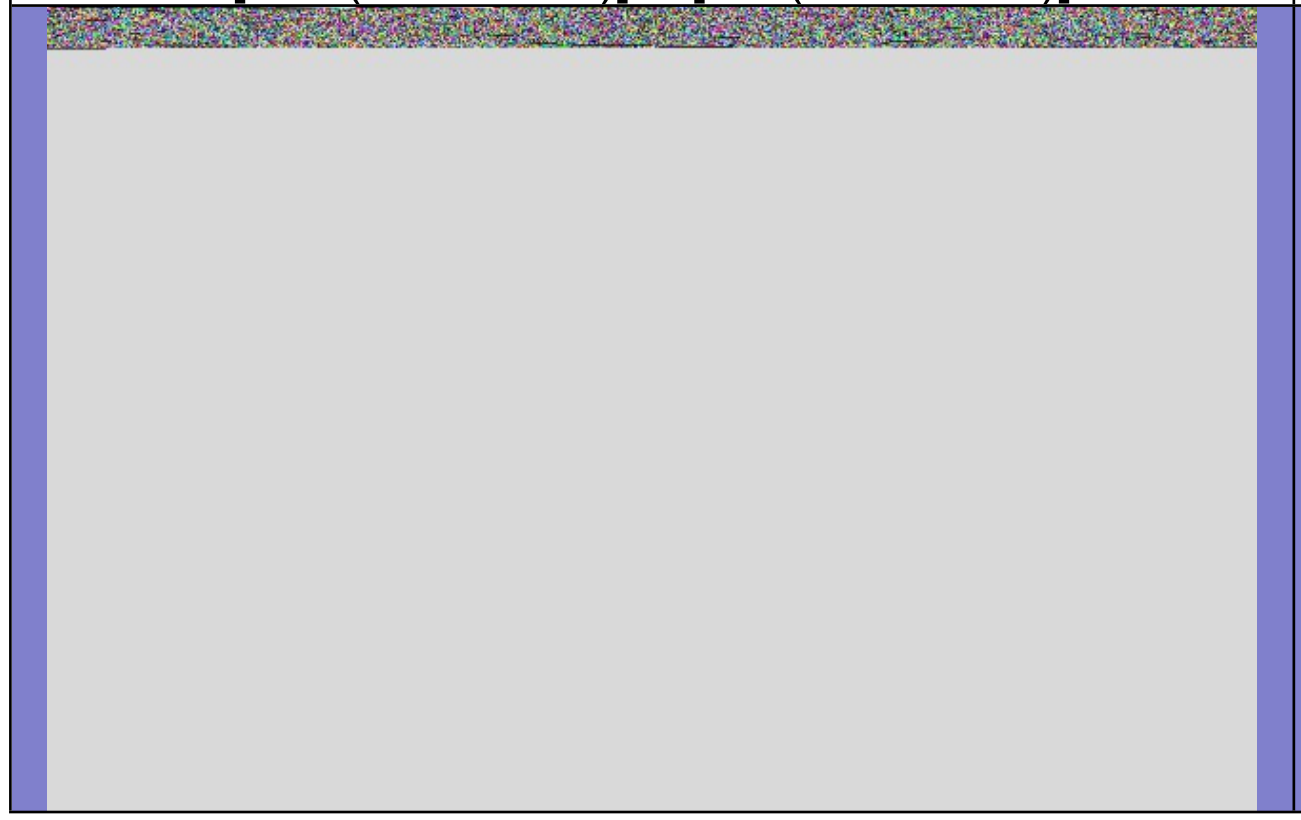	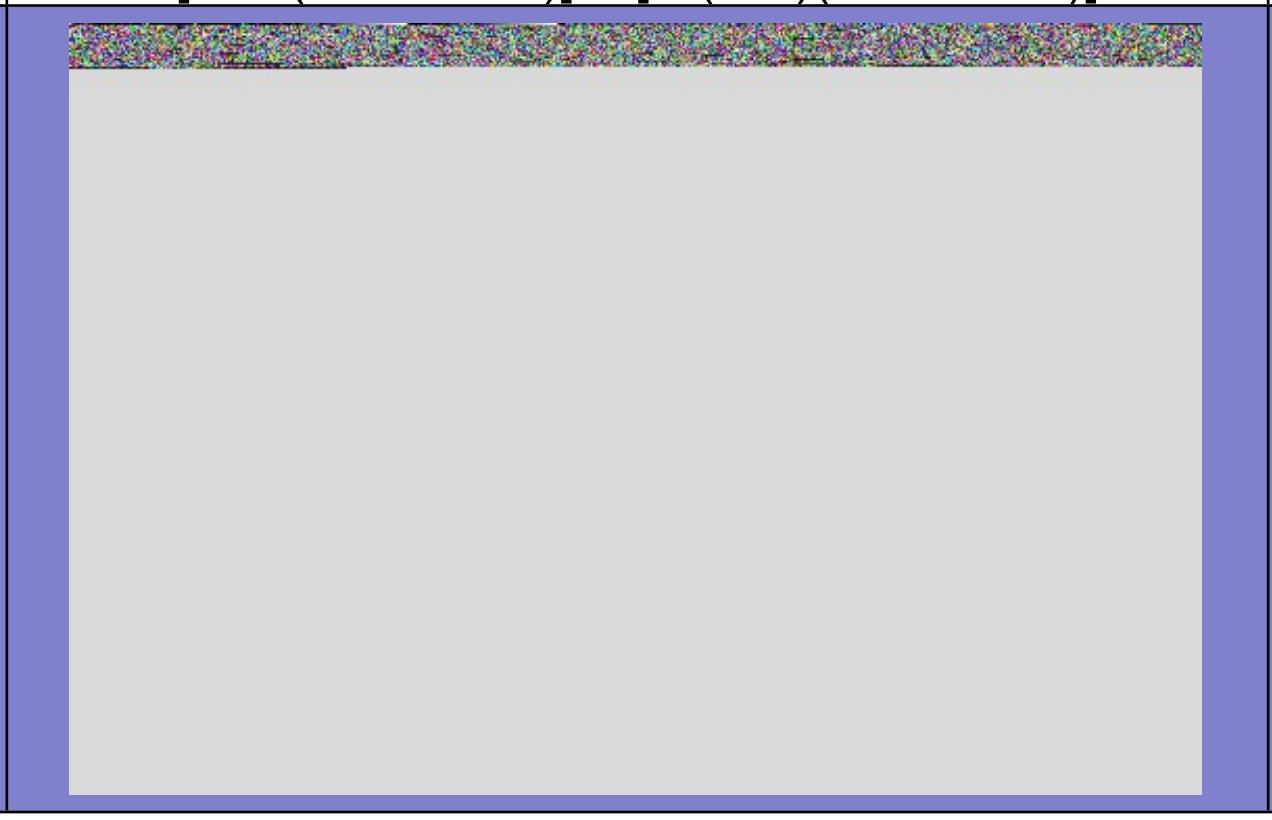	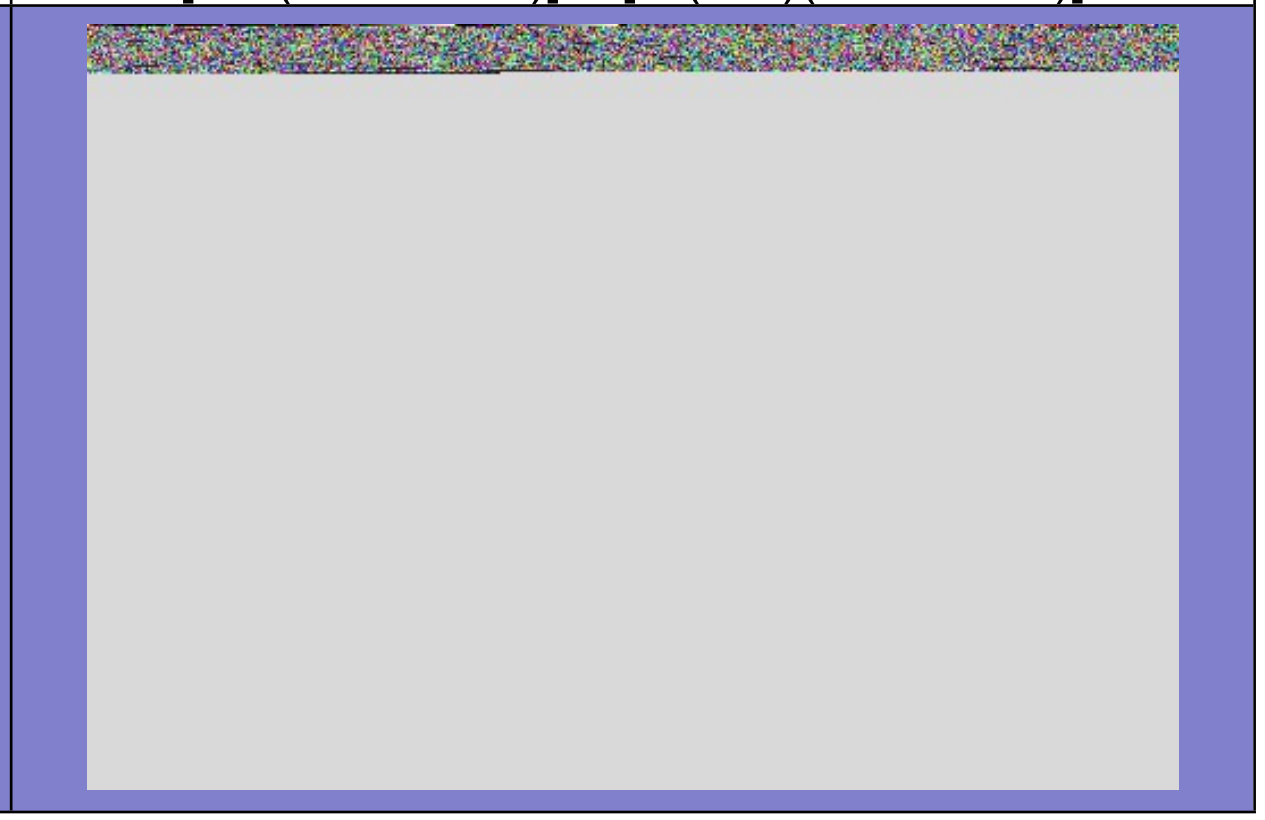

7e) IC2 geometries

IC2		
[GaCl(Bn-NODA)] – [GaF(Bn-NODA)]	[GaCl(Bn-NODA)] – [Ga(OH)(Bn-NODA)]	[GaF(Bn-NODA)] – [Ga(OH)(Bn-NODA)]
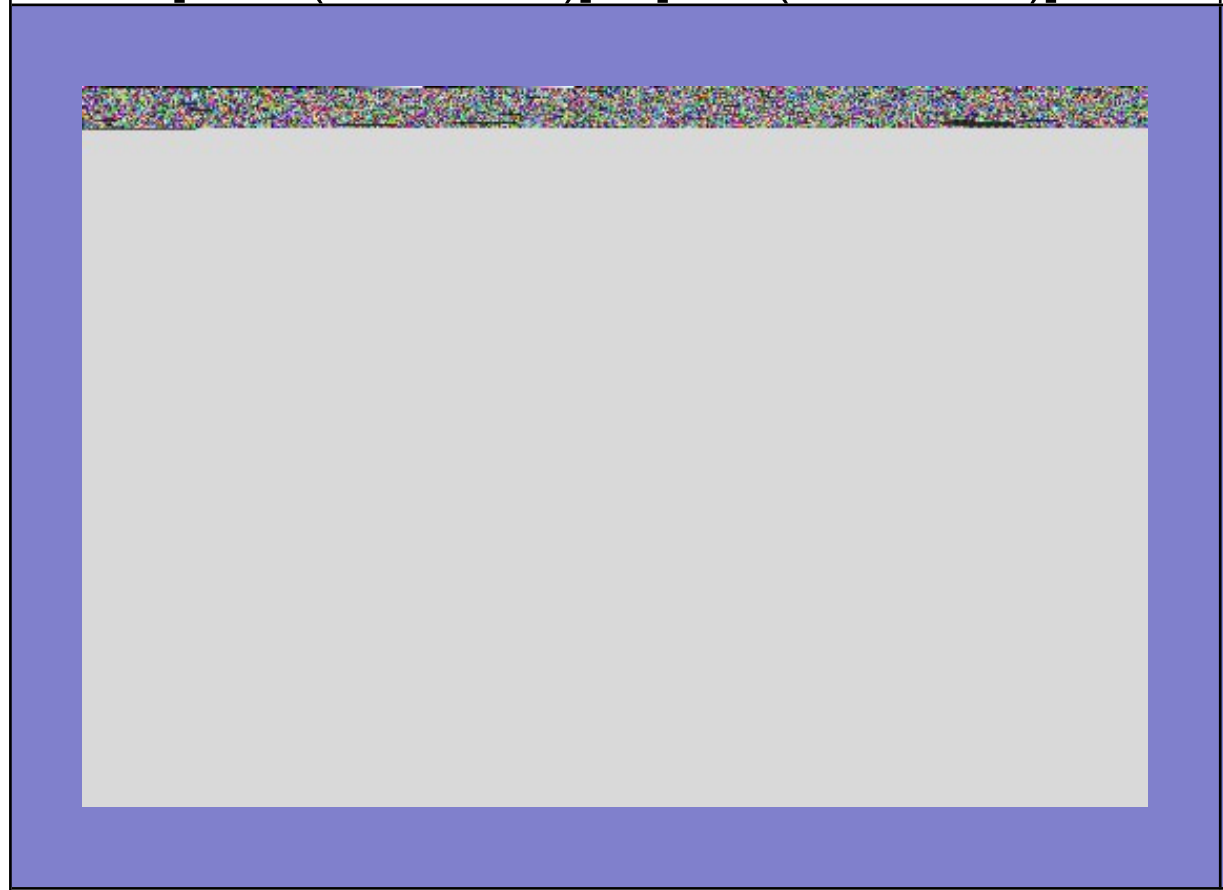	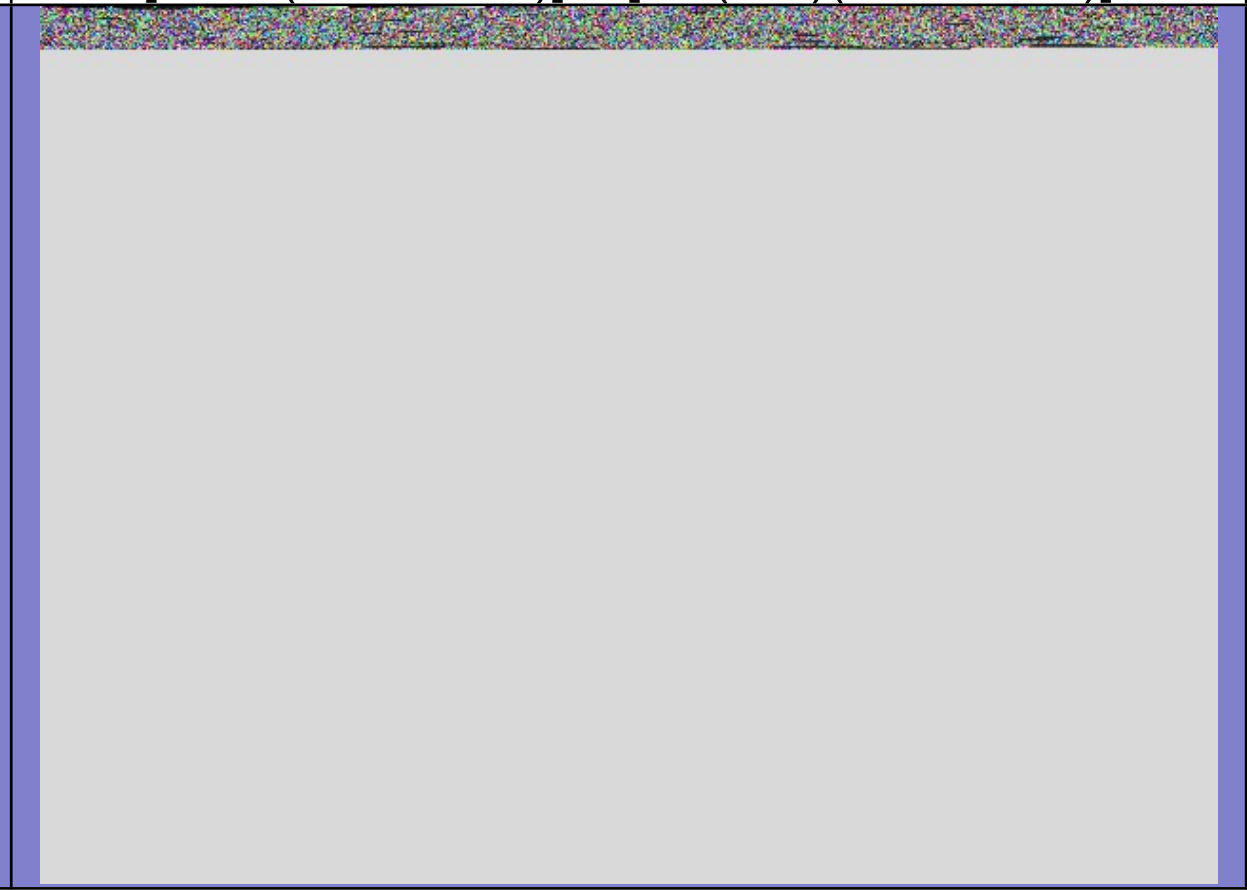	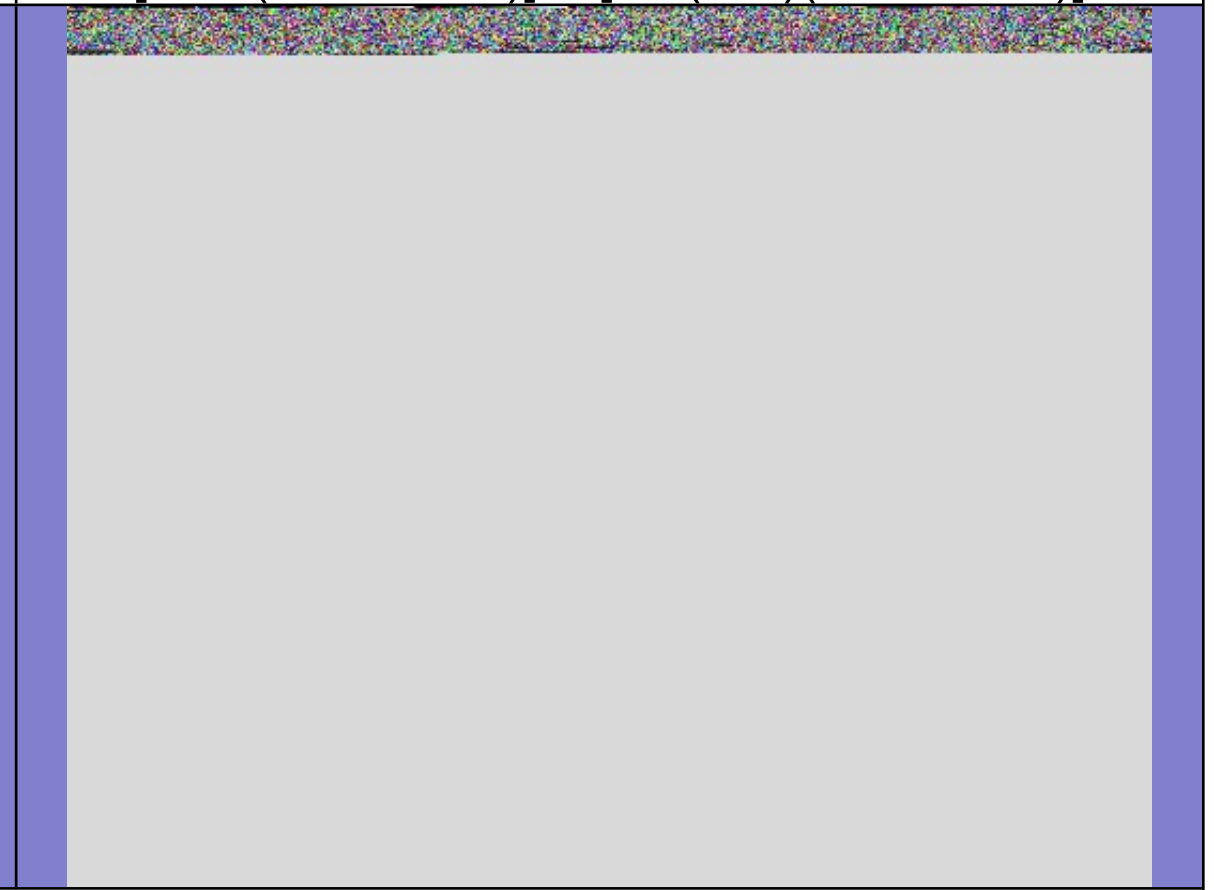
[AlCl(Bn-NODA)] – [AlF(Bn-NODA)]	[AlCl(Bn-NODA)] – [Al(OH)(Bn-NODA)]	[AlF(Bn-NODA)] – [Al(OH)(Bn-NODA)]
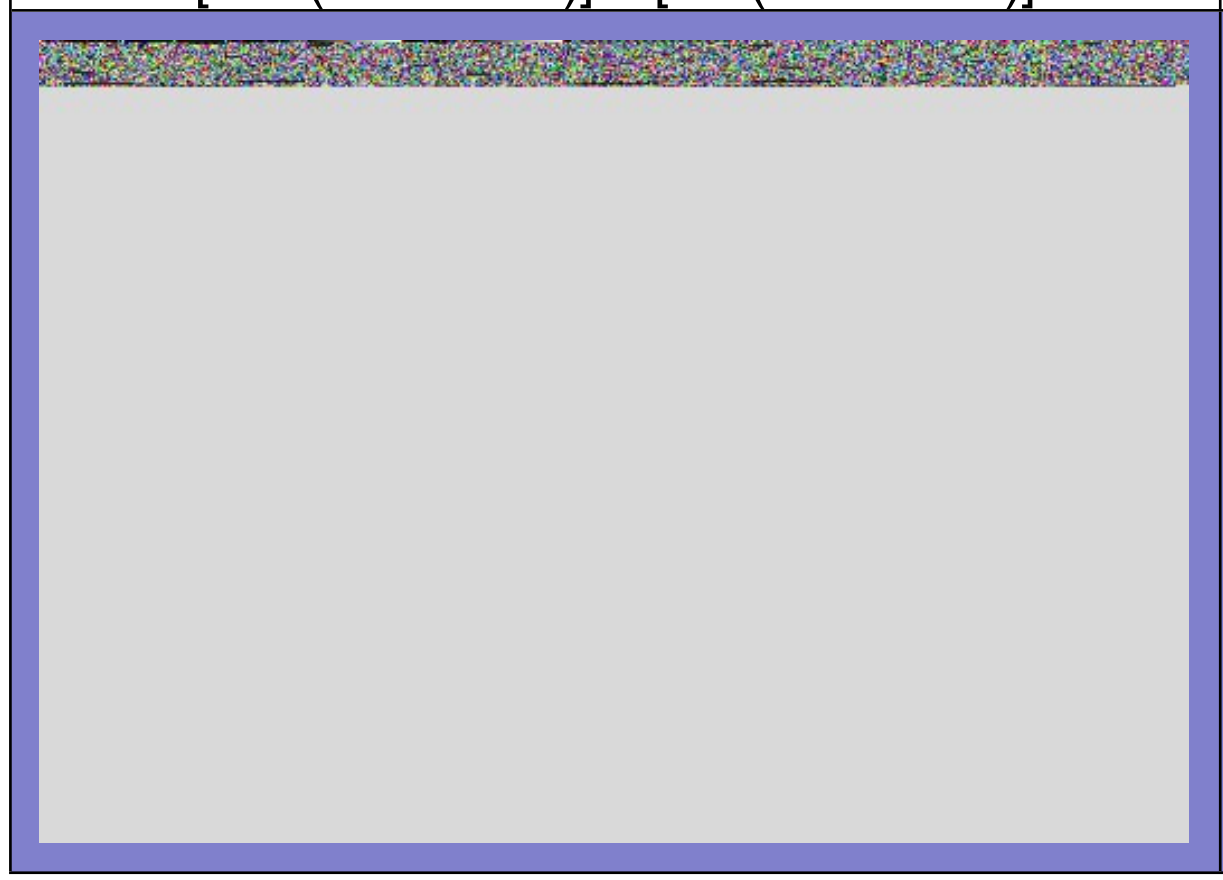	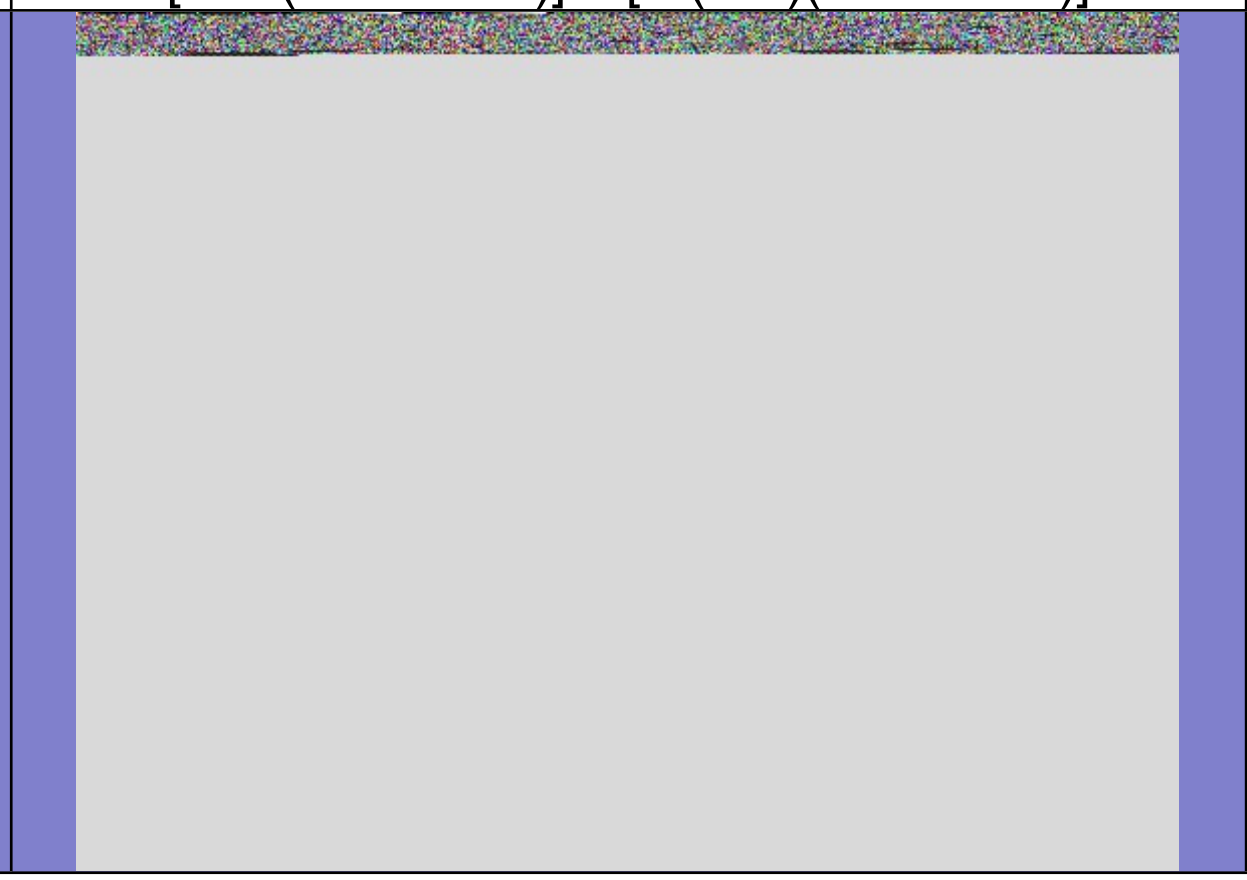	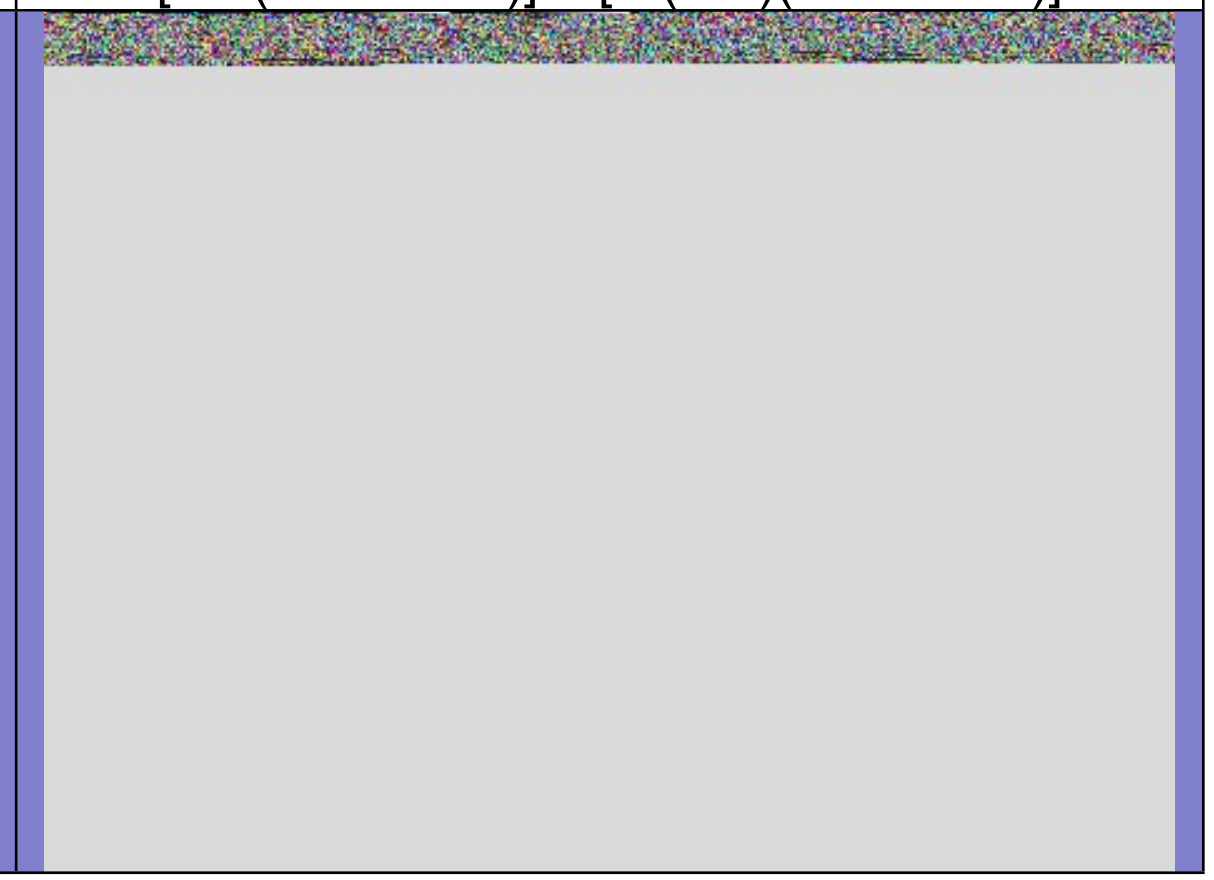

7f) TS2 geometries

**TS2**		
[GaCl(Bn-NODA)] – [GaF(Bn-NODA)]	[GaCl(Bn-NODA)] – [Ga(OH)(Bn-NODA)]	[GaF(Bn-NODA)] – [Ga(OH)(Bn-NODA)]
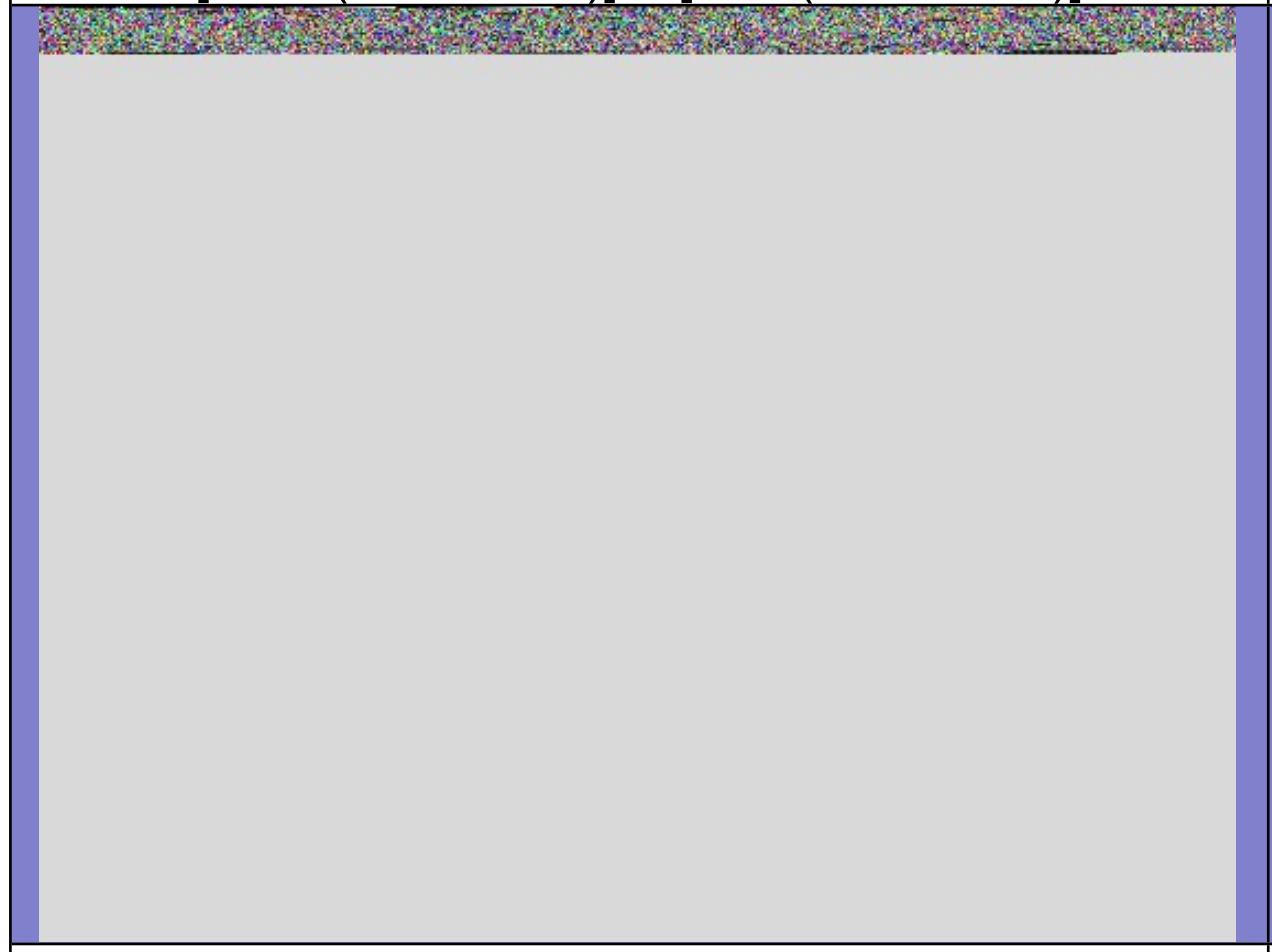	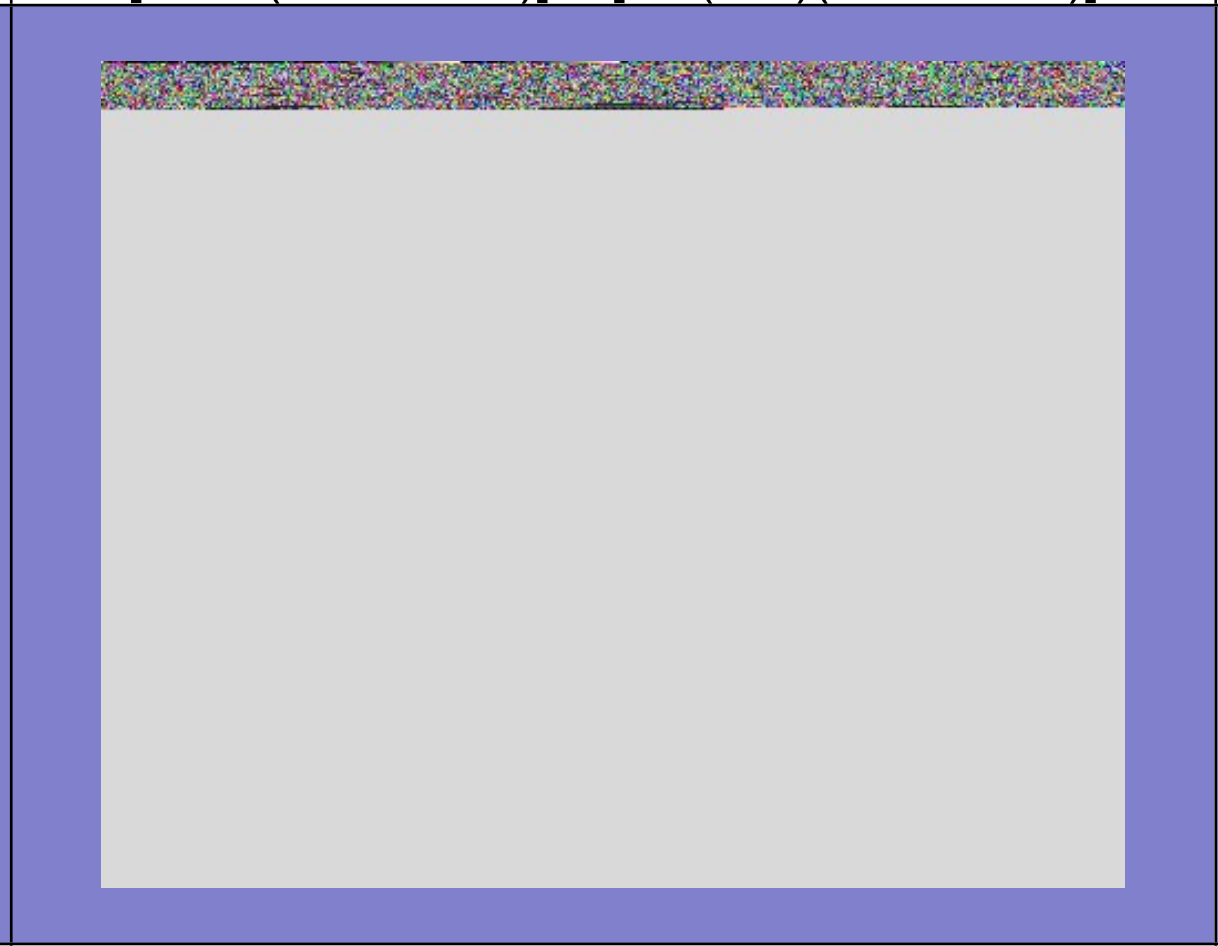	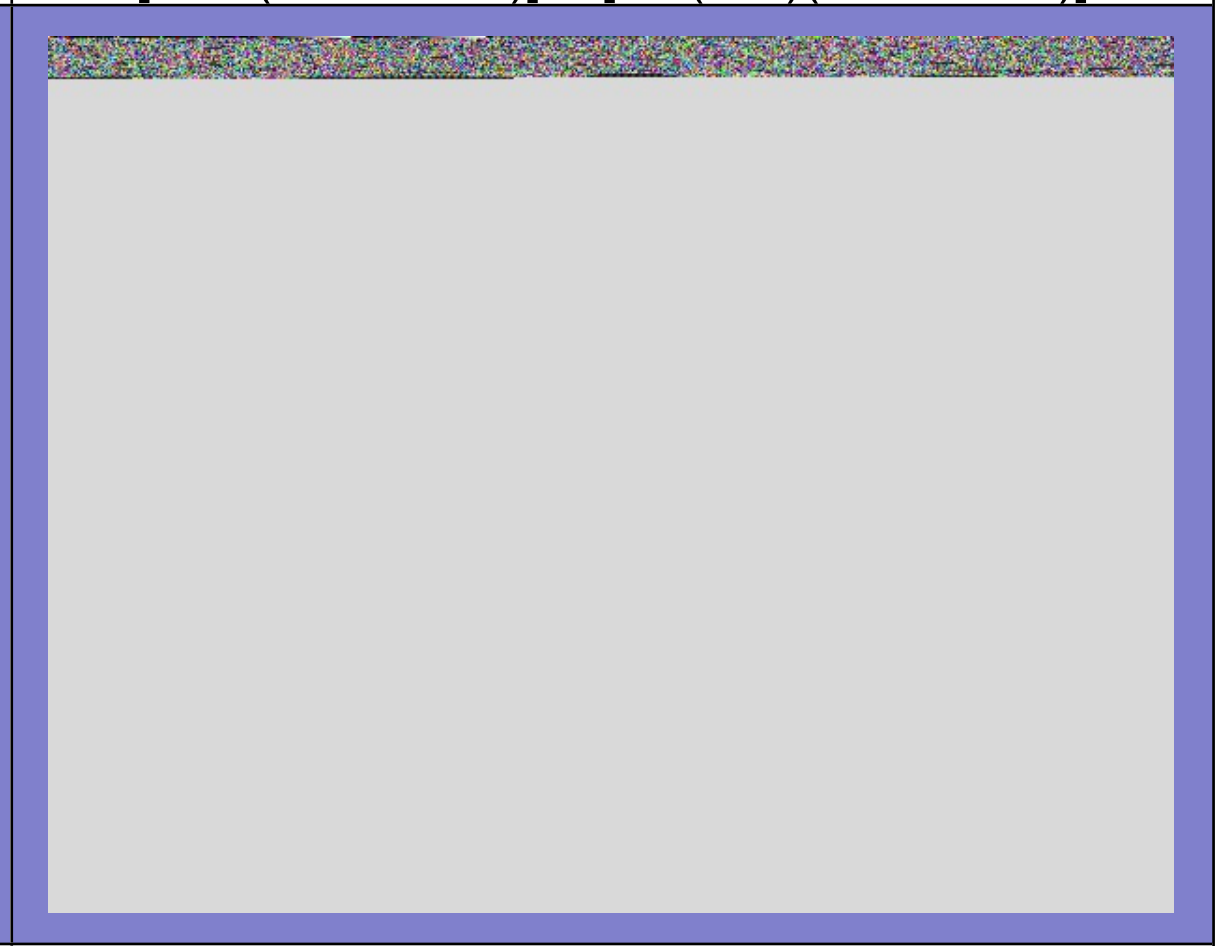
[AlCl(Bn-NODA)] – [AlF(Bn-NODA)]	[AlCl(Bn-NODA)] – [Al(OH)(Bn-NODA)]	[AlF(Bn-NODA)] – [Al(OH)(Bn-NODA)]
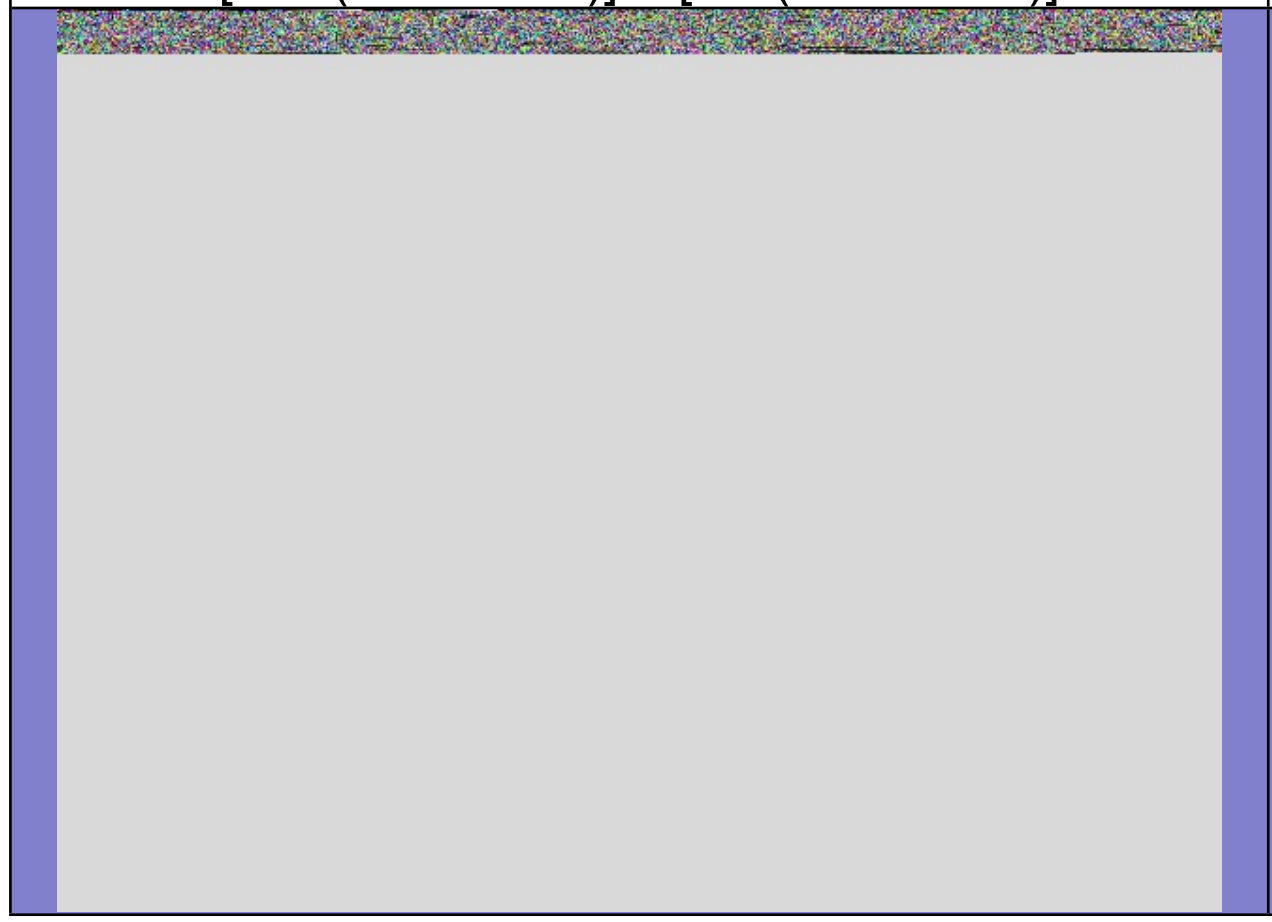	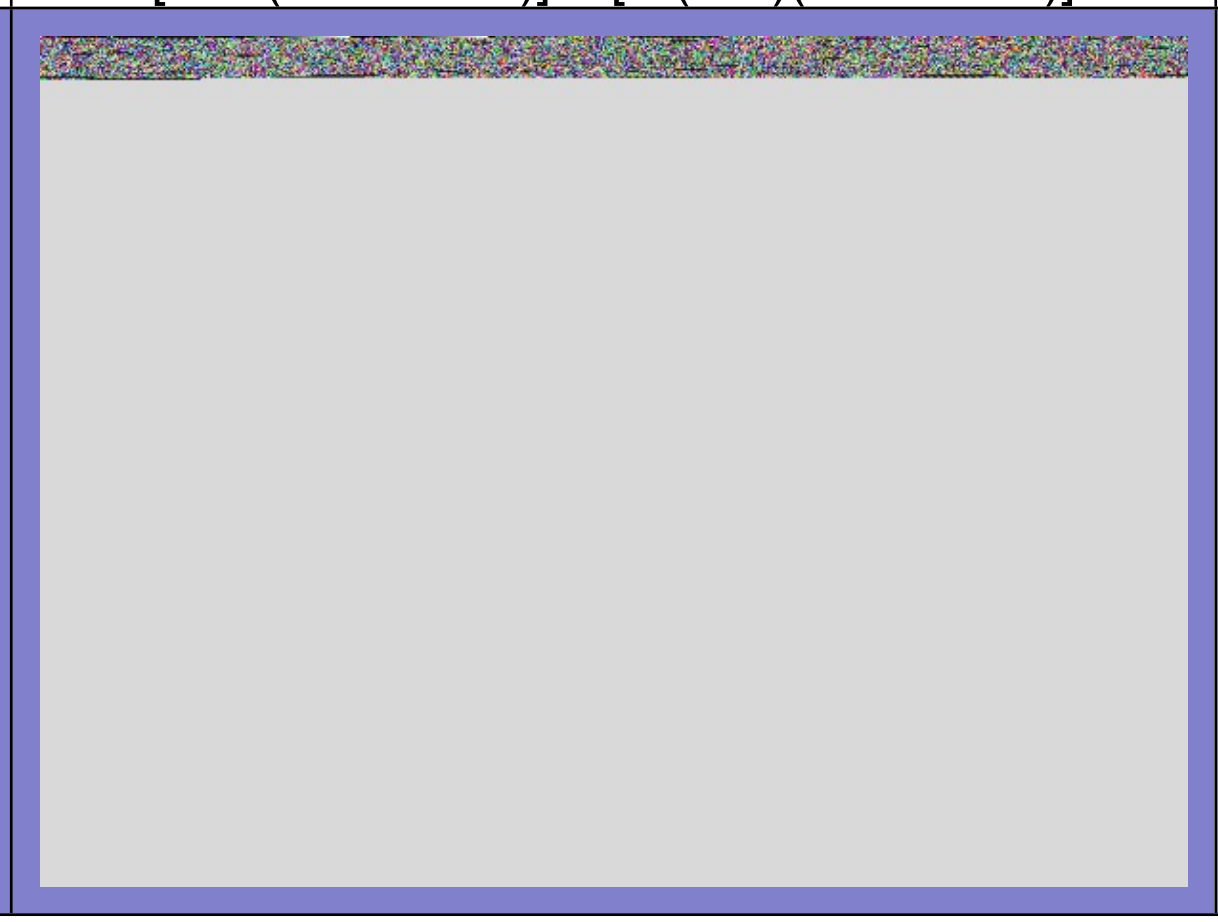	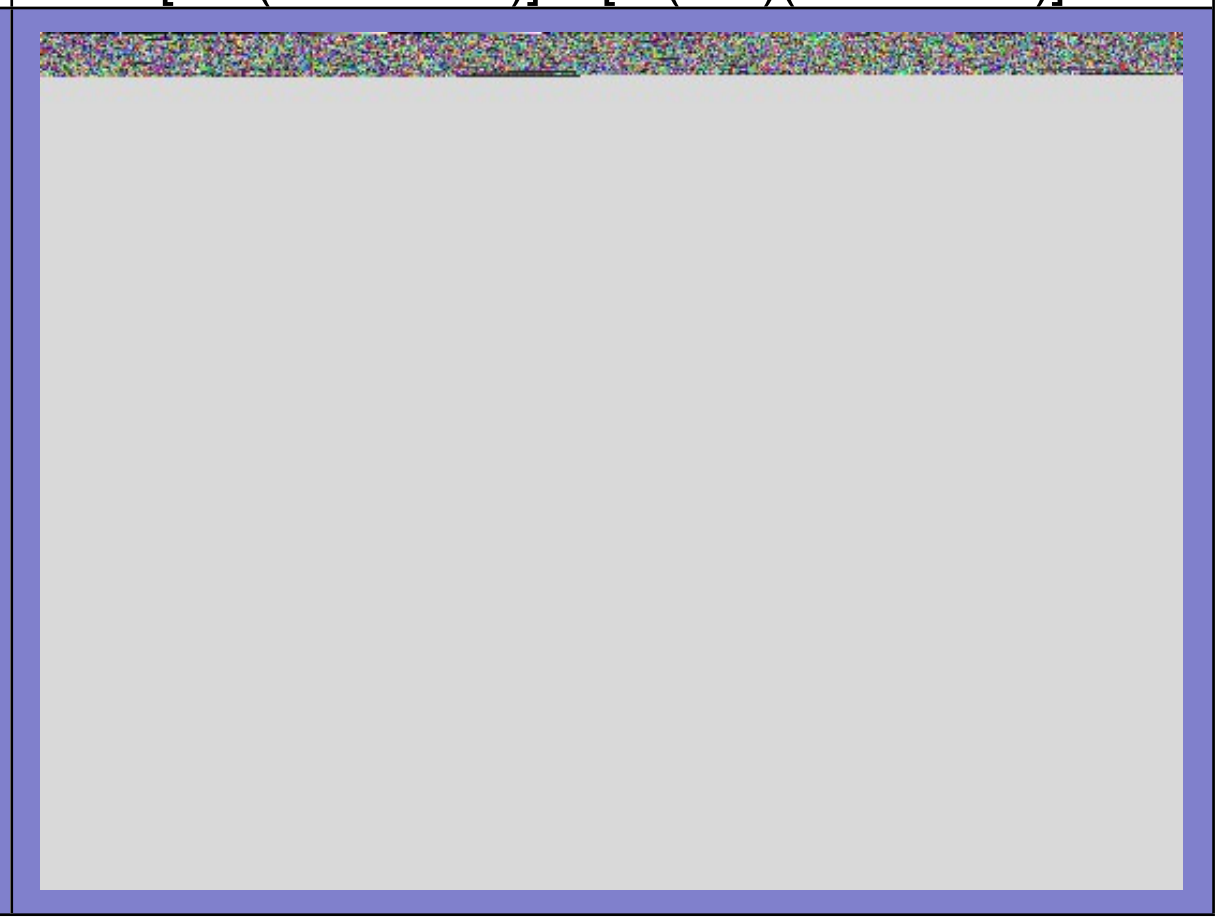

7g) PC geometries

PC		
[GaCl(Bn-NODA)] – [GaF(Bn-NODA)]	[GaCl(Bn-NODA)] – [Ga(OH)(Bn-NODA)]	[GaF(Bn-NODA)] – [Ga(OH)(Bn-NODA)]
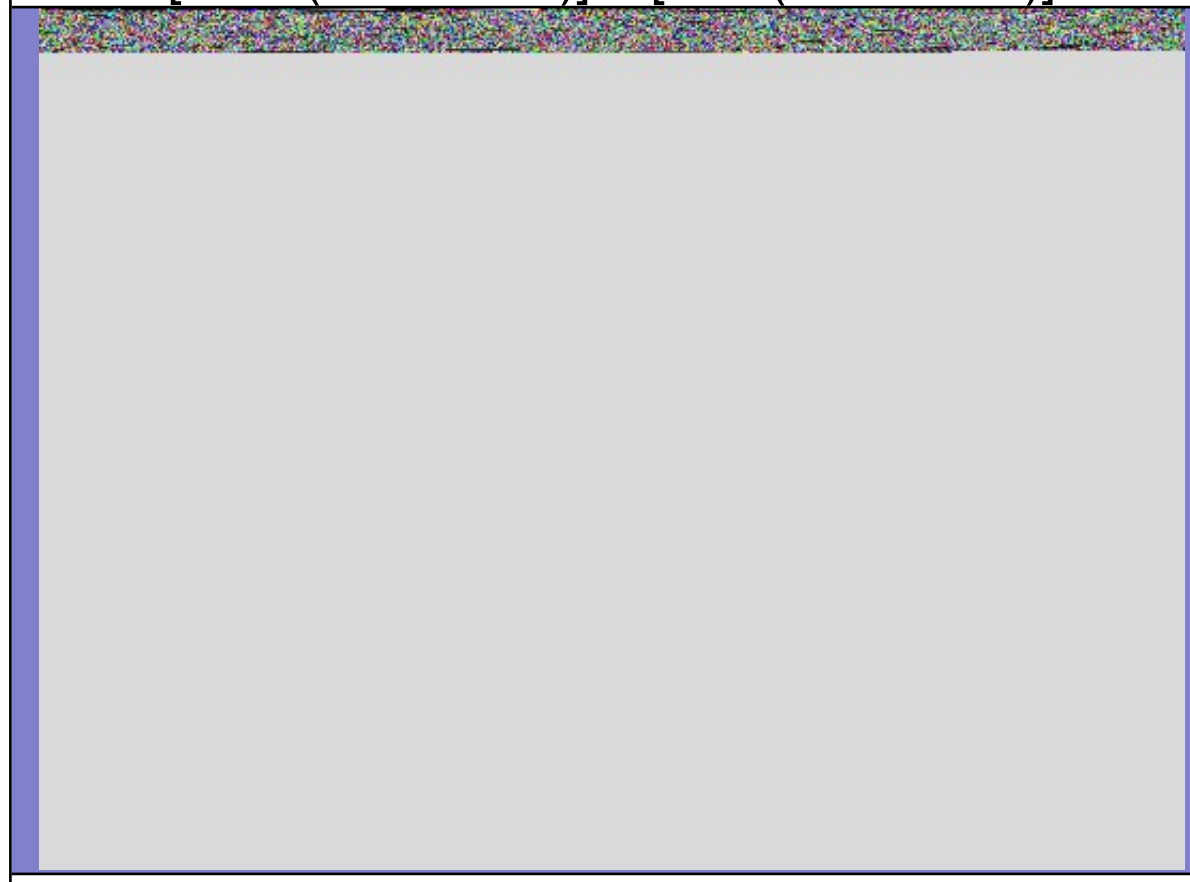	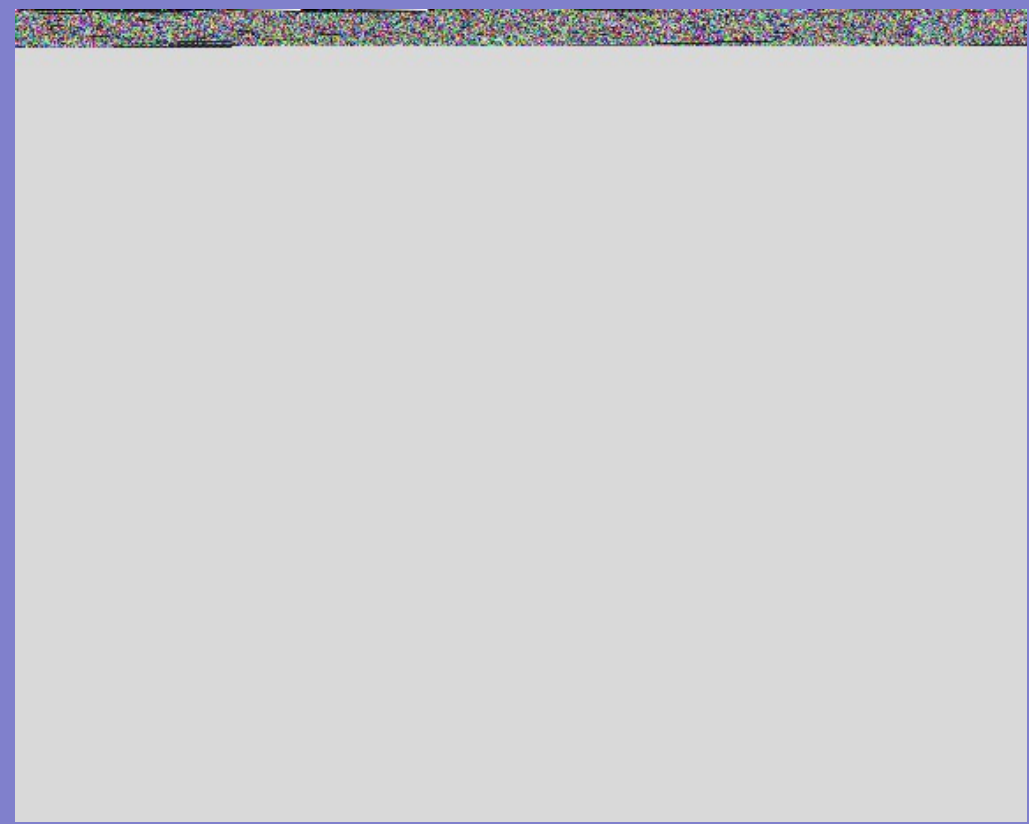	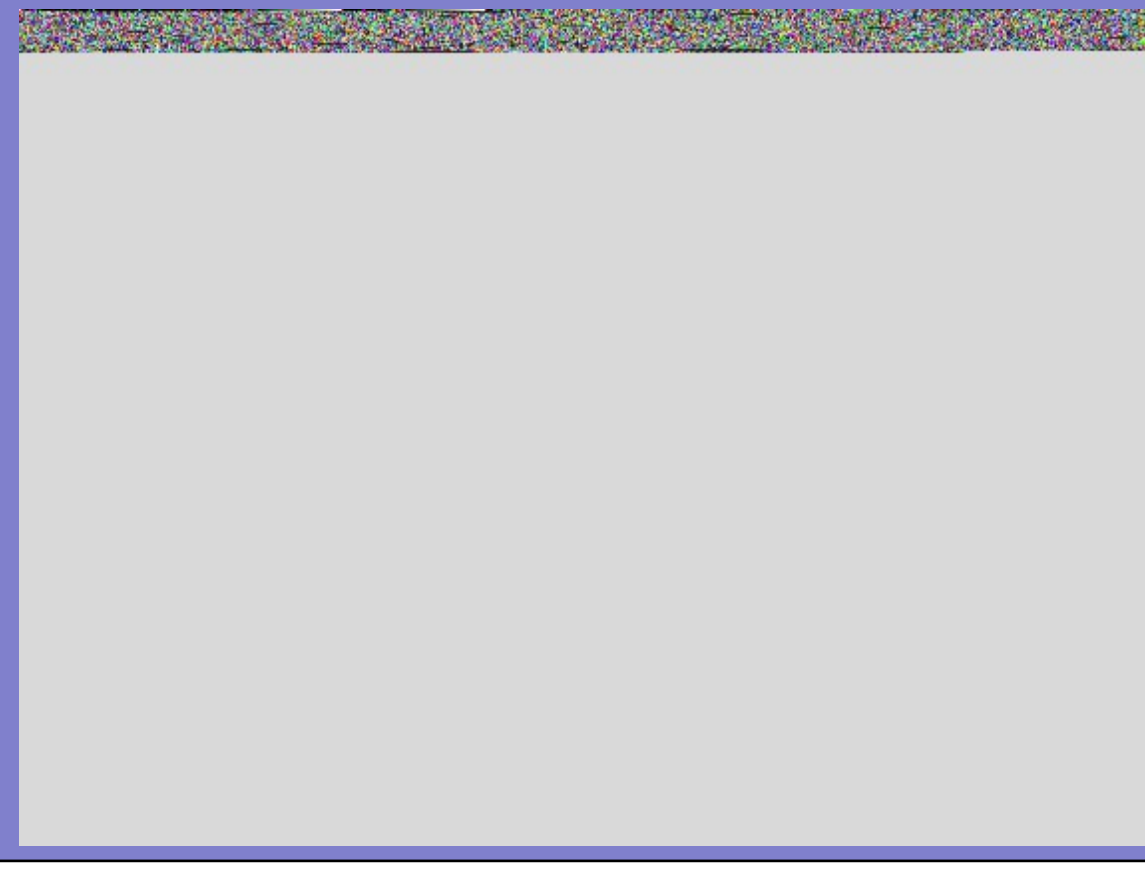
[AlCl(Bn-NODA)] – [AlF(Bn-NODA)]	[AlCl(Bn-NODA)] – [Al(OH)(Bn-NODA)]	[AlF(Bn-NODA)] – [Al(OH)(Bn-NODA)]
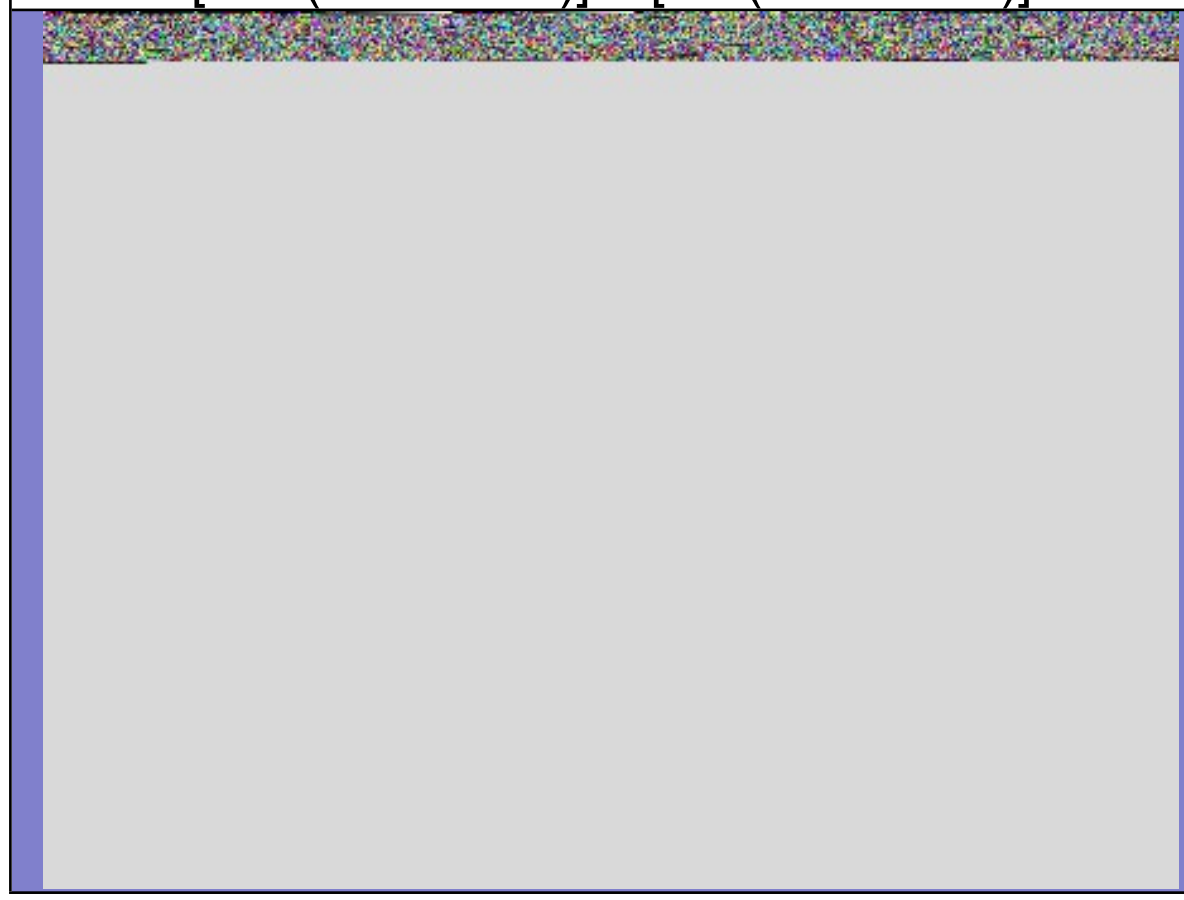	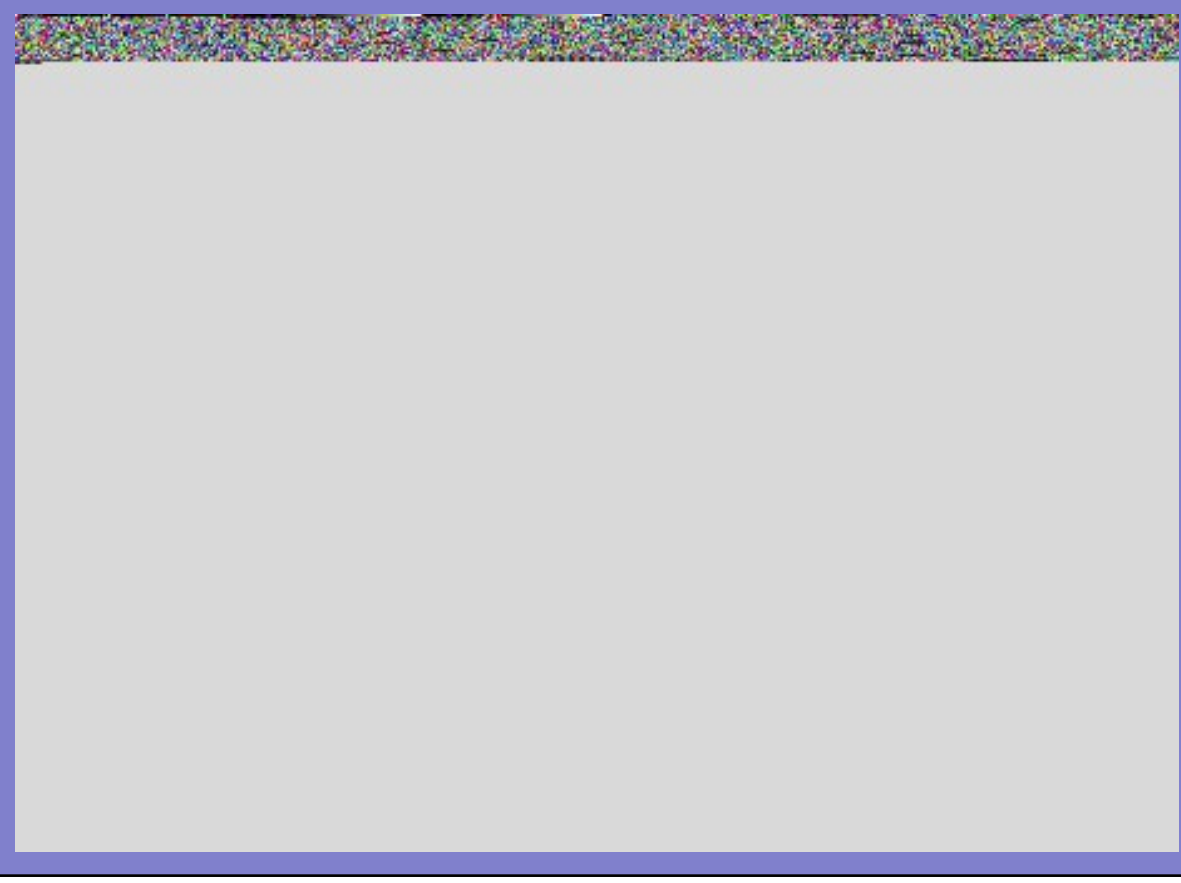	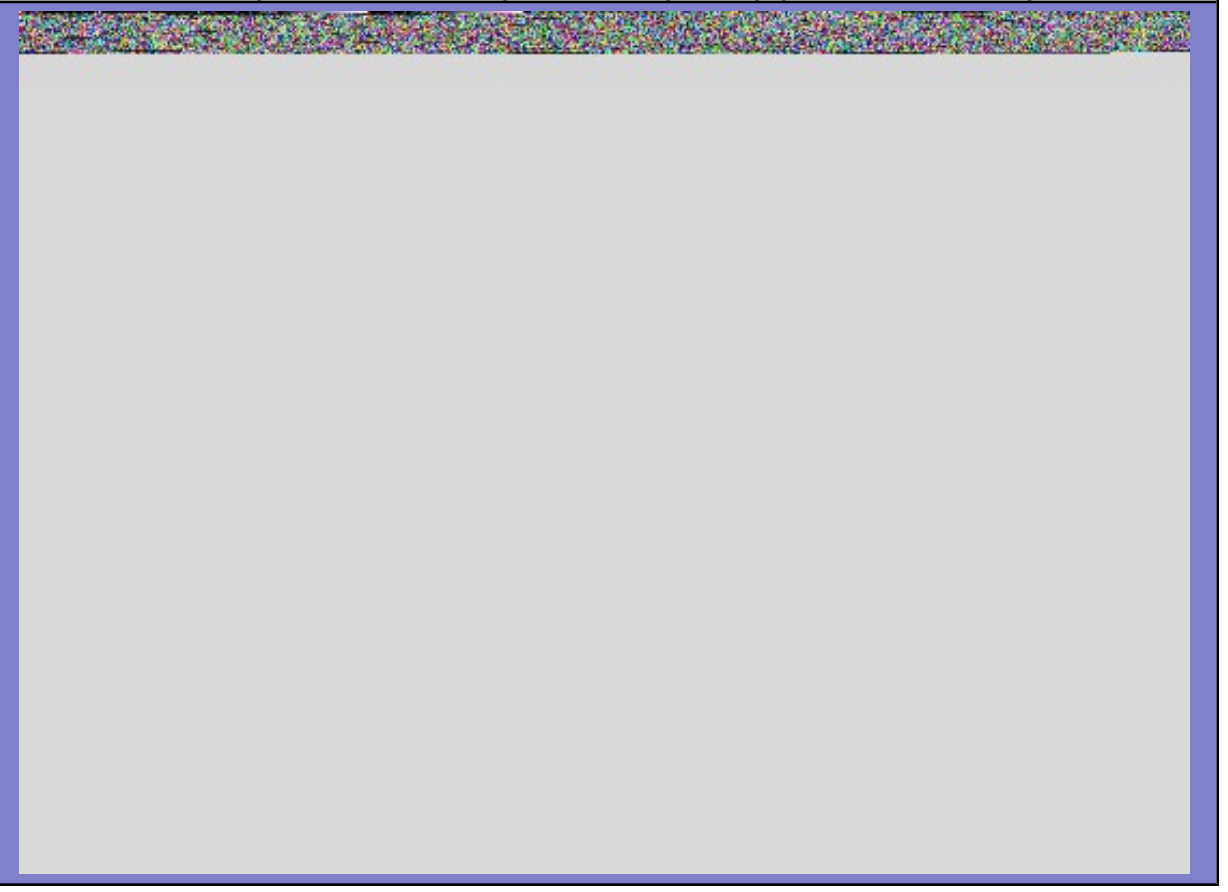

8. Summary for M-X Bond Lengths and M, X Charge Densities

Summary on the changes in M-X Bond Lengths and Mulliken Charge Densities for the Atoms (X) Close to Ga or Al (X= F and/or Cl), O (2 values), N (3 values) for the stationary points located for the six reactions investigated

8a) Reaction F^- + [MCl(L)], L=Bn-NODA

(i) M=Ga

For the **gas-phase** case the Ga-N_{trans} bond increases in length as the reaction proceeds from 2.24 Å in [GaCl(L)] to 2.96 Å in the TS **ICts**. It then reduces to 2.22 Å in [GaF(L)]. The other two Ga-N bonds show a similar change with maximum values at **IC1**, with the two Ga-O bond lengths being approximately constant throughout the reaction. The ionic complex **IC1** Ga can be viewed as 5-coordinate with Ga-F, Ga-Cl, Ga-N_{trans} and two Ga-O bonds as the two other Ga-N distances are all greater than the sum of the van der Waals radii of Ga and N of 3.42 Å[52]) (at 3.99 Å, and 4.14 Å; Ga-N_{trans} is 2.26 Å). They decrease to less than the sum of the van der Waals radii after **IC1**. The O atoms bonded to Ga show an increase in negative charge from [GaCl(L)] to [GaF(L), but a decrease from [GaCl(L)] to the ionic complex **IC1** and then an increase to [GaF(L)]. Similarly, the N atoms show a decrease of negative charge from [GaCl(L)] to the ionic complex **IC1**. The negative charge then increases from **IC1** to **ICts**, **IC2**, **TS2**, **PC** and [GaF(L). The overall negative charge on the N atoms is lower in [GaF(L)] than [GaCl(L)].

In the **solvated** case different changes in bond lengths are seen. The three Ga-N bonds show only small changes throughout the reaction whereas one Ga-O bond increases significantly from 2.04 Å in [GaCl(L)] to 4.33 Å in the ionic complex **IC1** (the sum of van der Waals radii for Ga and O is 3.39 Å.[52] It then reduces to 2.23 Å in **ICts**, and remains approximately constant at 2.03 Å in **IC2**, **TS2**, **PC** and the products. The second Ga-O bond which has been approximately constant at 1.99 Å, increases to 2.37 Å in **ICts**, and 4.07 Å in **IC2**. It then reduces to 3.19 Å in **TS2**, 1.99 Å in the PC and 1.98 Å in [GaF(L)]. In the ionic complexes, **IC1** and **IC2**, Ga can therefore be regarded as 6-coordinate with one Ga-F bond, one Ga-Cl bond, three Ga-N bonds and one Ga-O bond. The negative charges on the oxygen atoms remain approximately constant throughout showing a slight increase at the **PC** and the [GaF(L)] product. The negative charges on the nitrogen atoms also show a small increase from [GaCl(L)] to [GaF(L)], but on going from [GaCl(L)] they decrease first, to a minimum at **ICts** for two N atoms and at **IC1** for the other N atom, before increasing to [GaF(L)].

(ii) M=Al

As in the M = Ga solvated case, as the reaction proceeds the Al-Cl bond gets longer and the Al-F bond gets shorter. Also, the overall trend in the charge on the halogen atoms is that the negative charge on Cl increases and the negative charge of F decreases as the reaction proceeds. Also, as in the F^- + [GaCl(L)] solvated case, the three Al-N bonds show only small changes throughout the reaction whereas larger changes are observed in one of the Al-O bonds which increases from 1.88 Å in [AlCl(L)] to 2.06 Å in **IC1**, to 2.25 Å in **ICts**, to 4.07 Å in **IC2**. It then decreases to 3.04 Å in **TS2** and to 1.89 Å in [AlF(L)]. As the sum of van der Waals radii in Al and O is 3.36 Å,[52] in **IC2** Al can be regarded as 6-coordinate with one Al-F, one Al-Cl, three Al-N bonds and one Al-O bond, whereas in **TS1**, **IC1**, **ICts** and **TS2** the Al is 7-coordinate with two Al-O bonds and one Al-F, one Al-Cl, three Al-N bonds. As in

the F^- + [GaCl(L)] solvated case, the negative charges on the oxygen atoms remain approximately constant showing a slight increase at the **PC** and the [AlF(L)] product. The negative charges on the nitrogen atoms show a similar trend to the F^- + [GaCl(L)] case, showing an overall decrease from [AlF(L)] to [AlF(L)].

8b) Reaction OH^- + [MCl(L)], L=Bn-NODA

(i) M=Ga

For the 1-step process (reaction **4c**) the overall reaction profile shows the same stationary points as seen in Figures 2 and S1e (see Figure 5 and Table 4). Reaction (**4c**) has an overall ΔE= -15.1 kcal mol^{-1} ($\Delta G^{\phi}_{f,298K}$ = -13.5 kcal mol^{-1}). The corresponding values for F^- + [GaCl(L)] are -9.4 kcal mol^{-1} (-10.2 kcal mol^{-1}). As observed previously for the other 1-step reactions, the main potential barrier for F^- + [GaCl(L)] reaction (**4c**) occurs at **TS1** ΔE = +12.2 kcal mol^{-1}($\Delta G^{\phi}_{f,298K}$ = 21.7 kcal mol^{-1}). **TS1** for F^- + [GaCl(L)] is at ΔE= +11.4 kcal mol^{-1} ($\Delta G^{\phi}_{f,298K}$ = +18.4 kcal mol^{-1}). The Ga-X distances and Mulliken charges on the atoms bonded to Ga for the stationary points located on the reaction surface (Figure 5) are listed earlier in the SI. Similar trends are observed as seen for the OH^-+ [AlCl(L)] and the F^-+ [GaCl(L)] reactions. One Ga-O bond increases to greater than the sum of the van der Waals radii of Ga and O (3.39 Å[52]) at **IC1**. It then decreases to below this value at **ICts** and increases to greater than 3.70 Å at **IC2**. It has lower values in **TS2**, **PC** and [Ga(OH)(L)]. The other Ga-O bond remains approximately constant throughout the reaction.

(ii) M=Al

As the OH^-+ [AlCl(L)] reaction (**4c**) proceeds, the Al-Cl bond gets longer and the Al-OH bond gets shorter. The negative charge on Cl increases and the negative charge on the O atom of O-H decreases. As observed previously for F^-+ [AlCl(L)], the 3 Al-N bond lengths change only slightly throughout the reaction, but of the two Al-O bonds (involving O atoms from the carboxylate groups) one increases in length to 3.59, 3.87, 3.72 Å in **IC1**, **ICts**, **IC2**, from 1.88 Å in [AlCl(L)], before decreasing to 2.92 Å in **TS2**, 1.91 Å in **PC** and 1.90 Å in [Al(OH)(L)]. The other Al-O bond remains approximately constant at 1.92 Å throughout the reaction. Therefore, in **IC1**, **ICts**, and **IC2** the Al is effectively 6-coordinate. This elongation of one Al-O bond in **IC1**, **ICts** and **IC2** to greater than the sum of the atomic van der Waals radii of 3.36 Å[52] is more pronounced than in the F^- + [AlCl(L)] reaction where only significant elongation of one Al-O bond to beyond the sum of the atomic van der Waals radii occurs in **IC2**. The negative charges on the nitrogen atoms bonded to Al show a similar trend as in the F^- + [AlCl(L)] case, showing an overall decrease from [AlCl(L)] to [Al(OH)(L)]. Again, as in the F^- + [AlCl(L)] case, the negative charges on the oxygen atoms of the carboxylate groups bonded to Al remain approximately constant, showing a slight increase at the **PC** and [Al(OH)(L)] product. The Al-X bond distances and computed Mulliken charges on these atoms are listed earlier in the SI section and a schematic potential energy diagram for this reaction is shown in Figure 5.

8c) Reaction OH^- + [MF(L)] for M=Ga and Al, L=Bn-NODA

Very similar changes in bond lengths and atomic charge densities were observed for these reactions as observed for the other reactions (**4c**). In each case, the negative charge on the F atom increases and the negative charge on the O atom of the O-H group decreases

as the reaction proceeds. The negative charges on the other oxygen atoms show only small changes and the charges on the N atoms decrease steadily throughout the reaction. The Ga-N bond lengths remain approximately constant throughout the reaction. Of the three Ga-O bond distances, in each case one increases to above the sum of the van der Waals radii for **IC1**, **ICts**, and **IC2**, whereas the others remain lower and approximately constant. Schematic potential energy diagrams for the OH^- + [AlF(L)] and OH^- + [GaF(L)] reactions are shown in Figure 6.

9. Atoms in Molecules – Summary for [MCl(L)] + F^- → [MF(L)] + Cl^-

AIM analysis was undertaken for the reaction of [MCl(L)] + F^- → [MF(L)] + Cl^- in both the gas phase and in solution for M = Ga, and in solution for M = Al

~ Gaussian numbering used throughout – AIM results from multiwfn program ~

Atoms in molecules is based upon a topological analysis of the electron density (ρ) of the molecular system. It identifies a bond critical point (BCP) in a bond, which is a saddle point along the gradient path ∇ρ(r) (*Laplacian of electron density*) connecting two local electron ρ(r) density maxima. From the values of ρ(r) (*Density of all electrons*) and $\nabla^2\rho(r)$ at the BCPs, it is possible to determine the nature of a bond.[44,45]

9a) Gas-phase Calculations – Gallium Reaction Complex (**RC**)

Table for Hydrogen Bond Strengths using RC - BP86-D3/6-311++G(d,p) MULTIWFN outputs—using calculated PEDs (Potential Energy Densities, V(r))

MultiWfn identifies 112 CPS.

System has 50 Atoms, 54 "formal bonds" and 112 CPs

i.e. expect 8 "non-bonded interactions" 3 Cl···H, 1 C···H, 1 O···H, 2 H···F, 1 H···H

CP No.	V(r)/au	V(r) /kcal mol^{-1}	Gaussian Atom numbering	Bond Type	
73	-0.009453082993	-5.931899137	H30-Cl2	H—Cl	Non-bonded interactions
58	-0.006323866823	-3.968286344	H46-Cl2	H—Cl	
62	-0.005760920182	-3.615031993	H49-Cl2	H—Cl	
107	-0.07265942927	-45.59448024	24H-F50	H—F	
102	-0.02595775840	-16.28873932	11H-F50	H—F	
63	-0.005031738350	-3.157463485	42H-26C	H—C	
79	-0.008445544923	-5.299659452	22H-43H	H—H	
104	-0.006853251571	-4.300480289	18H-8O	H—O	
109	-0.2250012671	-141.190456924	C23-C40	C-C	known bonds
110	-0.2685365139	-168.509242579	C23-H24	C-H	
111	-0.2954341278	-185.387753735	C23-H25	C-H	
80	-0.07407973512	-46.48573562	1Ga-2Cl	Ga-Cl	Bonds around Ga
93	-0.07464692096	-46.8416147	1Ga-N5	Ga-N	
78	-0.07446001438	-46.7243292	1Ga-N6	Ga-N	
85	-0.04600531127	-28.8687469	1Ga-N15	Ga-N	
86	-0.1332786708	-83.6335654	1Ga-O3	Ga-O	
98	-0.1565462787	-98.2341988	1Ga-O8	Ga-O	

CP	Atom No. A	Atom No. B	Bond Density ρ	$\nabla^2 \rho$	A-B distance Å	Formal Bond?	Sum of vdW radii[45] Å	Computed Mayer Bond Order
73	H30	Cl2	0.01657330566	0.05406714831	2.50773	N	2.95	0.26396013
58	H46	Cl2	0.01206428601	0.03665279870	2.70118	N	2.95	0.19507842
62	H49	Cl2	0.01056691041	0.03412026467	2.81048	N	2.95	0.07455839
107	H24	F50	0.07016896203	0.1752517700	1.52352	N	2.67	0.27254073
102	H11	F50	0.03174338115	0.1092319558	1.87900	N	2.67	0.16051203
63	H42	C26	0.009799514540	0.03211354894	2.59877	N	2.9	0.13513114
79	H22	H43	0.01439300955	0.05057857815	2.01624	N	2.4	0.02006657
104	H18	O8	0.01072781871	0.03560257897	2.57053	N	2.72	-0.00087102
109	C23	C40	0.2268031885	-0.6505161060	1.51390	Y	3.40	0.90143118
110	H24	C23	0.2511848883	-0.5612788734	1.17688	Y	2.80	0.74246029
111	H25	C23	0.2671274583	-0.8498860188	1.10315	Y	2.80	1.00616677
80	Ga1	Cl2	0.06753882118	0.1097495098	2.32807	Y	3.62	0.13287805
93	Ga1	N5	0.06558348973	0.1371116184	2.19395	Y	3.42	0.19023857
78	Ga1	N6	0.06494404240	0.1401033664	2.19386	Y	3.42	0.22466590
85	Ga1	N15	0.04738343080	0.09691366972	2.35125	Y	3.42	0.11315426
86	Ga1	O3	0.09127912588	0.3579705335	1.95060	Y	3.39	1.09111989
98	Ga1	O8	0.1019852284	0.4250057665	1.90535	Y	3.39	0.78461969

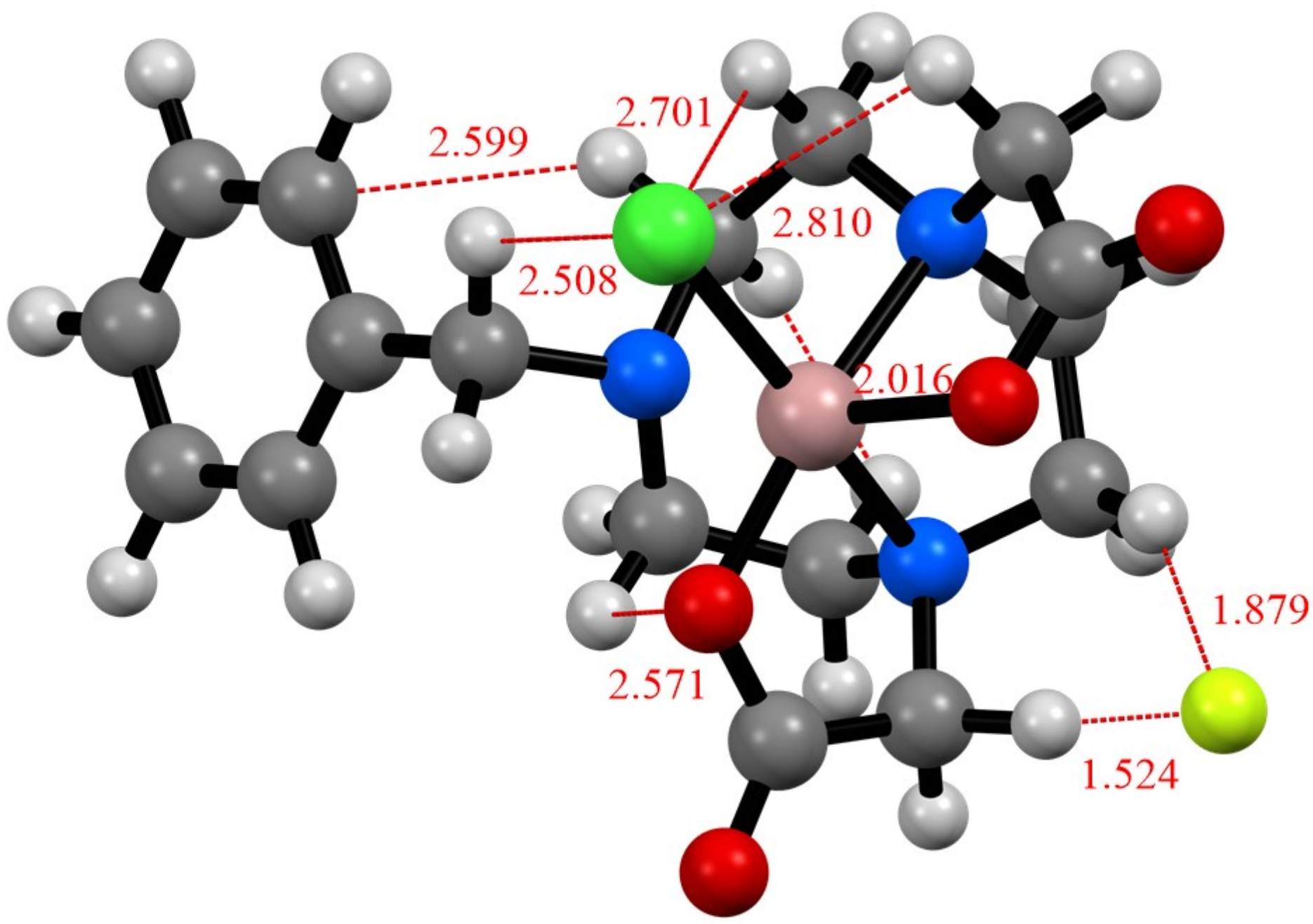

9b) Gas-phase Calculations – Gallium Transition State 1 (**TS1**)

Table for Hydrogen Bond Strengths using RC - BP86-D3/6-311++G(d,p)
MULTIWFN outputs—using calculated PEDs (Potential Energy Densities, V(r))
MultiWfn identifies 113 CPS.
System has 50 Atoms, 54 "formal bonds" and 113 CPs
i.e. expect 9 "non-bonded interactions" 2 Cl···H, 1 O···F, 2 C···H, 1 H···F, 2 H···H

CP No.	V(r)/au	V(r) /kcal mol^{-1}	Gaussian Atom numbering	Bond Type	
74	-0.008970084288	-5.628812873	H30-Cl2	H—Cl	Non-bonded interactions
63	-0.007252598910	-4.551074527	H46-Cl2	H—Cl	
108	-0.05910765801	-37.09061539	H24-F50	H—F	
103	-0.03622627052	-22.73232796	H50-H11	H—H	
107	-0.005048111103	-3.167737543	H18-C40	H—C	
97	-0.01576612753	-9.893394393	O3-F50	O—F	
59	-0.004912434571	-3.082599234	H42-C26	H—C	
99	-0.01154891366	-7.247052736	Ga1-F50	Ga-F	
77	-0.009407753157	-5.903454235	H22-H43	H—H	
111	-0.2625046445	-164.724186576	C23-C40	C-C	known bonds
110	-0.2650562362	-166.325334884	C23-H24	C-H	
112	-0.2942571880	-184.649212702	C23-H25	C-H	
83	-0.07185368884	-45.08887049	Ga1-Cl2	Ga-Cl	Bonds around Ga
80	-0.06737047992	-42.2755825	Ga1-N5	Ga-N	
95	-0.04508068823	-28.2885376	Ga1-N6	Ga-N	
85	-0.01084594975	-6.80593108	Ga1-N15	Ga-N	
86	-0.1354108884	-84.9715512	Ga1-O3	Ga-O	
102	-0.1502334640	-94.2728508	Ga1-O8	Ga-O	

CP	Atom No. A	Atom No. B	Bond Density ρ	$\nabla^2 \rho$	A-B distance Å	Formal Bond?	Sum of vdW radii[45] Å	Computed Mayer Bond Order
74	H30	Cl2	0.01599261771	0.05182385638	2.51947	N	2.85	0.23609522
63	H46	Cl2	0.01381129544	0.04189842081	2.60848	N	2.85	0.31049324
108	H24	F50	0.05786539731	0.1841248518	1.60962	N	2.57	0.21968343
103	H50	H11	0.04032597323	0.1379326083	1.76943	N	2.20	0.20823530
107	H18	C40	0.009046837597	0.03232707842	2.56889	N	2.80	0.02396119
97	O3	F50	0.01844036178	0.07211847274	2.58173	N	2.99	-0.04882975
59	H42	C26	0.009579077266	0.03136134560	2.61536	N	2.80	0.11645195
99	Ga1	F50	0.01676231323	0.04233146583	2.78460	N	3.34	0.09396380
77	H22	H43	0.01617820400	0.05565676835	1.94605	N	2.20	0.02766192
111	C23	C40	0.2486905956	-0.5519032365	1.52110	Y	3.40	0.90173994
110	C23	H24	0.2533032263	-0.7973982567	1.13437	Y	2.80	0.81307659
112	C23	H25	0.2671757164	-0.8516659221	1.10301	Y	2.80	0.99166652
80	Ga1	N5	0.06190375051	0.1239596391	2.35130	Y	3.42	0.23554183
83	Ga1	Cl2	0.06634700896	0.1079080805	2.33385	Y	3.62	0.67465648

85	Ga1	N15	0.01827494938	0.04091610894	2.86439	Y	3.42	0.02548271
86	Ga1	O3	0.09315176564	0.3632722678	1.94111	Y	3.39	0.83542711
95	Ga1	N6	0.04823033193	0.08888199118	2.21999	Y	3.42	0.28280781
102	Ga1	O8	0.09980321972	0.4079211017	1.91147	Y	3.39	0.74996009

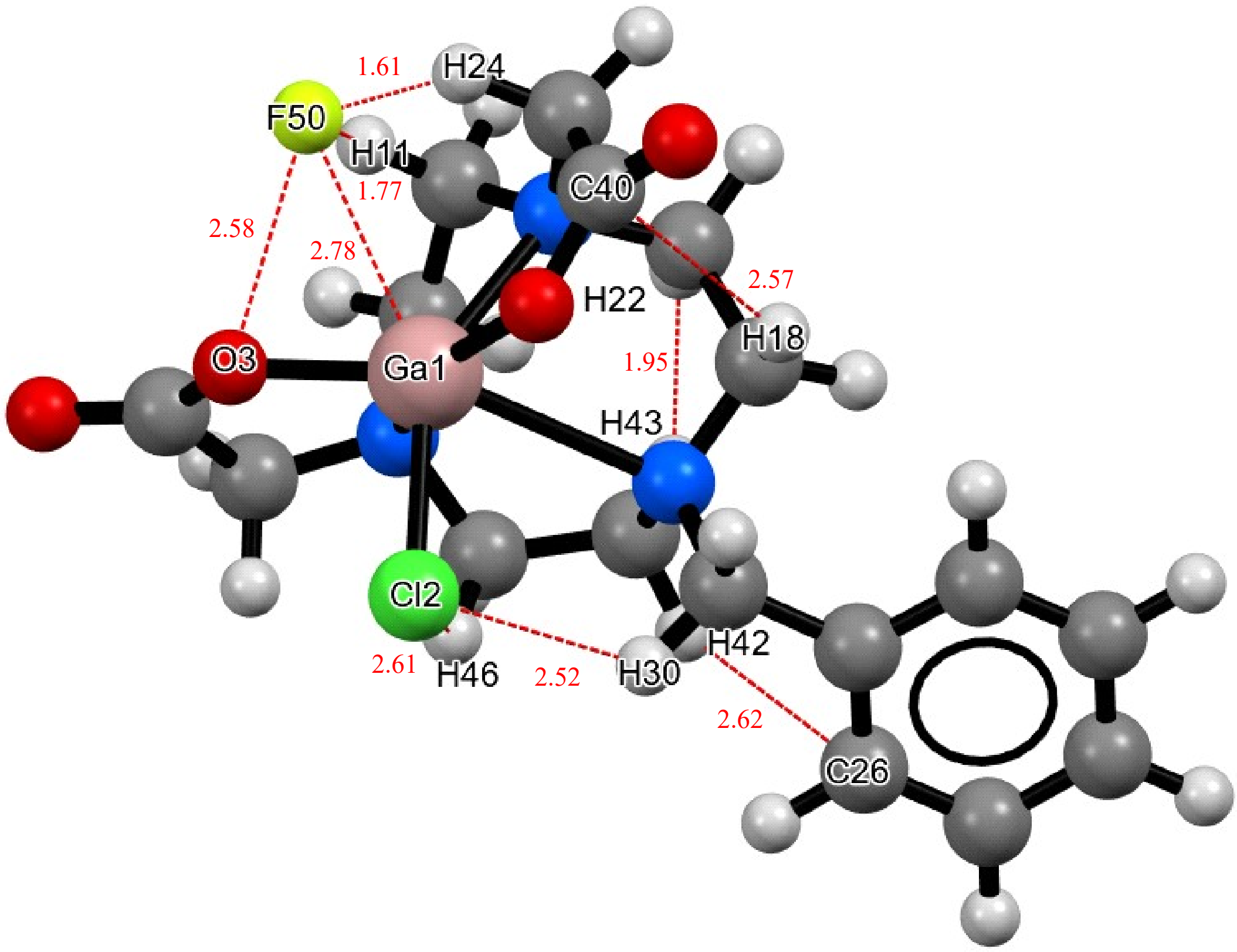

9c) Gas-phase Calculations – Gallium Ionic Complex 1 (**IC1**)

Table for Hydrogen Bond Strengths using IC1 /BP86-D3 6-311++G(d,p) GAS PHASE MULTIWFN outputs—using calculated PEDs (Potential Energy Densities, V(r))

MultiWfn identifies 109 CPS.

System has 50 Atoms, 53 "formal bonds" and 109 CPs

i.e. expect 6 "non-bonded interactions" 1 Cl···H, 2 O···H, 1 H···H, 1 F···O, 1 N···C

CP No.	V(r)/au	V(r) /kcal mol^{-1}	Gaussian Atom numbering	Bond Type	
60	-0.00577436	-3.62340873	49H – 2Cl	Cl—H	Non-bonded interactions
70	-0.00852601	-5.35006965	43H – 37H	H—H	
92	-0.00856500	-5.37453485	15N – 40C	N—C	
100	-0.00826289	-5.18496463	29H – 4O	O—H	
103	-0.00499092	-3.13180392	18H – 4O	O—H	
107	-0.01139687	-7.15153335	50F – 24H	F—O	
106	-0.23782015	-149.23214582	23C—40C	C—C	known bonds
108	-0.29634057	-185.95370893	23C—24H	C—H	
109	-0.29145834	-182.89010697	23C—25H	C—H	
81	-0.05983254	-37.54491660	6N-1Ga	Ga-N	Bo nd
82	-0.09254764	-58.07364634	2Cl-1Ga	Ga-Cl	

84	-0.10812458	-67.84817251	3O-1Ga	Ga-O	s around Ga
89	-0.13096033	-82.17760670	8O-1Ga	Ga-O	
97	-0.16815596	-105.51786365	1Ga-50F	Ga-F	

Note: only 1 Ga-N bond

CP	Atom No. A	Atom No. B	Bond Density ρ	$\nabla^2 \rho$	A-B distance Å	Formal Bond?	Sum of vdW radii[45] Å	Computed Mayer Bond Order
60	49H	2Cl	0.01105520	0.03498286	2.74961	N	2.85	0.15444099
70	43H	37H	0.01533728	0.05095560	1.94835	N	2.20	0.02475487
92	15N	40C	0.01313261	0.04546615	2.87108	N	3.25	0.01294480
100	29H	4O	0.01381597	0.04502729	2.30501	N	2.62	-0.00325750
103	18H	4O	0.00834571	0.02799775	2.65062	N	2.62	-0.03487610
107	50F	24H	0.01607620	0.05435949	2.21924	N	2.57	0.03437087
106	23C	40C	0.23825515	-0.50682474	1.54995	Y	3.40	0.76802079
108	23C	24H	0.27201519	-0.89295774	1.10204	Y	2.80	1.00607075
109	23C	25H	0.26741896	-0.85553406	1.10695	Y	2.80	1.05895520
81	6N	1Ga	0.05698905	0.11302051	2.25930	Y	3.42	0.13567107
82	2Cl	1Ga	0.07871716	0.13846374	2.24648	Y	3.62	0.84020470
84	3O	1Ga	0.07988204	0.28007485	2.01045	Y	3.39	0.75924610
89	8O	1Ga	0.08926075	0.36301358	1.94720	Y	3.39	0.53358837
97	50F	1Ga	0.10211246	0.55720216	1.84525	Y	3.34	0.96502892

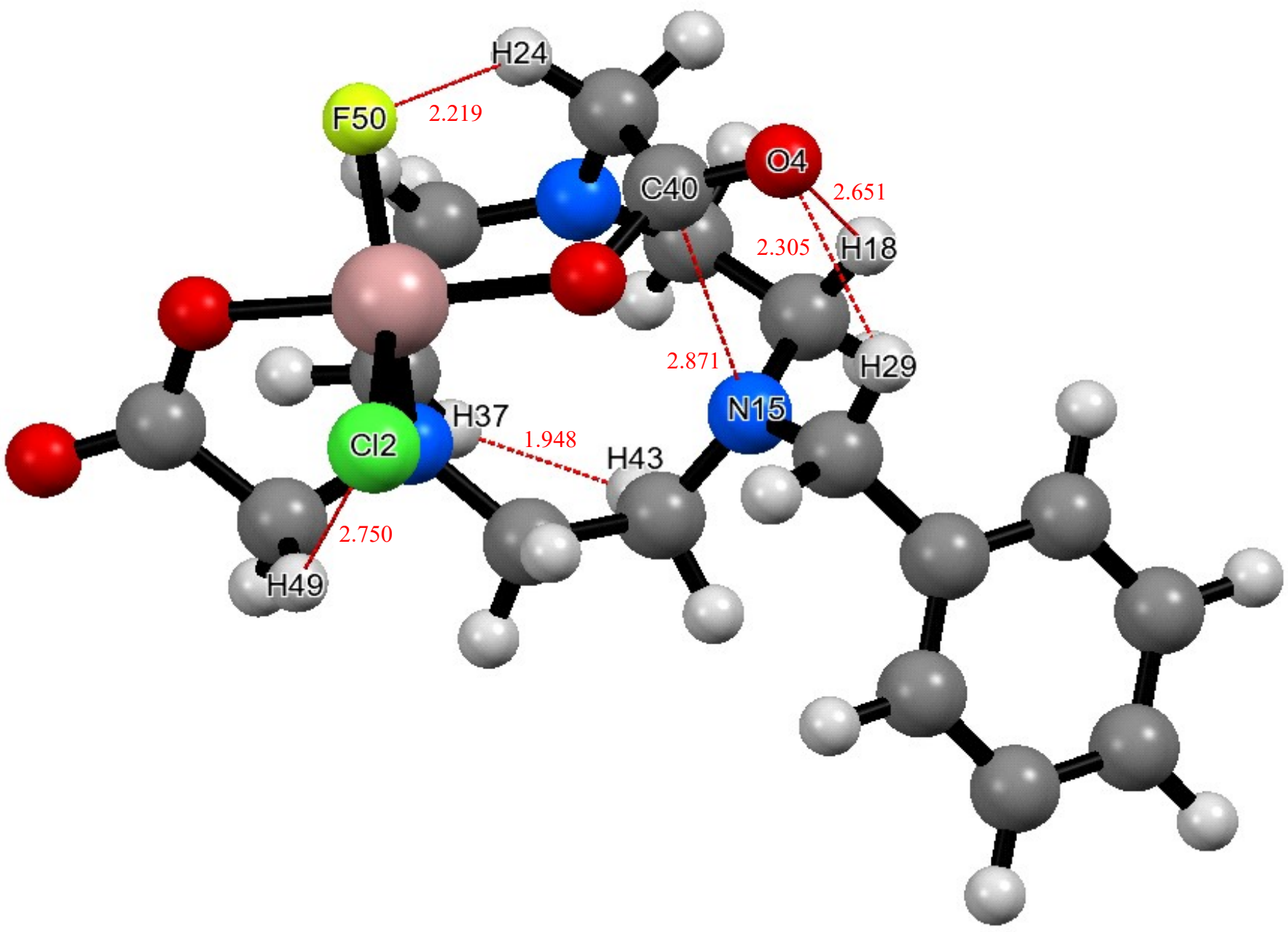

9d) Gas-phase Calculations – Gallium Ionic Complex transition state (**ICts**)

Table for Hydrogen Bond Strengths using ICts /Bp86-D3 6-311++G(d,p) GAS PHASE MULTIWFN outputs—using calculated PEDs (Potential Energy Densities, V(r))

MultiWfn identifies 112 CPS.

System has 50 Atoms, 54 "formal bonds" and 112 CPs

i.e. expect 8 "non-bonded interactions" 3 Cl···H, 2 O···H, 1 H···H, 2 C···H

CP No.	V(r)/au	V(r) /kcal mol^{-1}	Gaussian Atom numbering	Bond Type	
54	-0.01259099	-7.90084538	46H – 2Cl	Cl—H	Non-bonded interactions
58	-0.00804751	-5.04981228	49H – 2Cl	Cl—H	
63	-0.00545817	-3.42499880	42H – 26C	C—H	
68	-0.00981713	-6.16025106	30H – 2Cl	Cl—H	
80	-0.00856195	-5.37262069	37H – 22H	H—H	
87	-0.00582222	-3.65344385	12C – 11H	C—H	
104	-0.00516330	-3.23996811	3O – 24H	O—H	
106	-0.01271845	-7.98082584	18H – 8O	O—H	
108	-0.29469793	-184.92294888	23C—24H	C—H	known bonds
110	-0.24518356	-153.85268139	23C—40C	C—C	
111	-0.28600400	-179.46751119	23C—25H	C—H	
77	-0.05857927	-36.75849016	2Cl—1Ga	Ga-Cl	Bonds around Ga
78	-0.00893452	-5.60641268	6N—1Ga	Ga-N	
86	-0.02937581	-18.43331823	15N—1Ga	Ga-N	
88	-0.14100910	-88.48321006	3O—1Ga	Ga-O	
94	-0.17025109	-106.83256023	1Ga—50F	Ga-F	
102	-0.11626046	-72.95343984	8O—1Ga	Ga-O	

CP	Atom A	Atom B	Bond Density ρ	$\nabla^2 \rho$	A-B distance/ Å	Formal Bond?	Sum of vdW radii[45] Å	Computed Mayer Bond Order
54	46H	2Cl	0.02076612	0.06528959	2.38400	N	2.95	0.22902693
58	49H	2Cl	0.01484483	0.04574320	2.55984	N	2.95	0.14559553
63	42H	26C	0.01037857	0.03494212	2.56819	N	2.9	0.06339127
68	30H	2Cl	0.01754818	0.05464336	2.45195	N	2.95	0.24063758
80	37H	22H	0.01476735	0.05116680	1.99462	N	2.4	0.03022164
87	11H	12C	0.00999066	0.03659067	2.52761	N	2.9	-0.00559091
104	24H	3O	0.00789528	0.02897395	2.66738	N	2.72	-0.02154964
106	18H	8O	0.01873430	0.06909968	2.23935	N	2.72	-0.03846705
108	23C	24H	0.26988491	-0.87895948	1.10291	Y	2.9	1.00152683
110	23C	40C	0.24193439	-0.52667294	1.53980	Y	3.4	0.90722467
111	23C	25H	0.26402748	-0.83197609	1.11175	Y	2.9	0.98337625
77	2Cl	1Ga	0.05864419	0.09024728	2.39892	Y	3.62	0.59307300
78	6N	1Ga	0.01592312	0.03601286	2.95745	Y	3.42	0.15570789
86	15N	1Ga	0.03716084	0.06859887	2.48190	Y	3.42	0.11595801
88	3O	1Ga	0.09364173	0.39660681	1.92438	Y	3.39	0.47484039

94	50F	1Ga	0.10352239	0.55990780	1.84198	Y	3.34	0.31377883
102	8O	1Ga	0.08235983	0.31738425	1.97942	Y	3.39	1.02724166

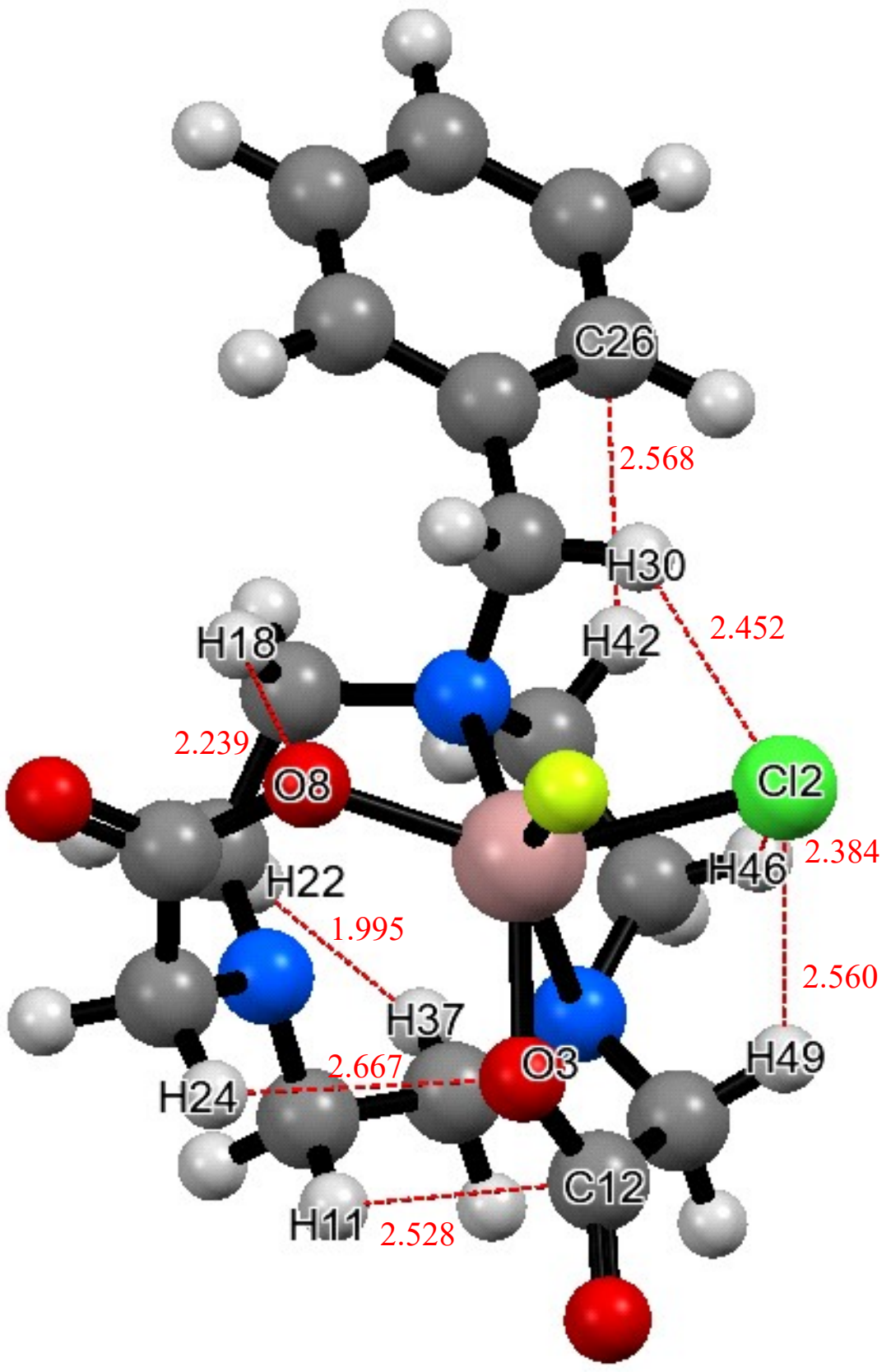

9e) Gas-phase Calculations – Gallium Ionic Complex 2 (**IC2**)

Table for Hydrogen Bond Strengths using IC2 /Bp86-D3 6-311++G(d,p) GAS PHASE MULTIWFN outputs—using calculated PEDs (Potential Energy Densities, V(r))

MultiWfn identifies 113 CPS.

System has 50 Atoms, 55 “formal bonds” and 113 CPs

i.e. expect 8 “non-bonded interactions” 3 Cl···H, 1 O···H, 1 H···H, 1 F···H, 2 C···H

CP No.	V(r)/au	V(r) /kcal mol^{-1}	Gaussian Atom numbering	Bond Type	
54	-0.01447811	-9.08501217	46H—2Cl	Cl—H	Non-bonded interactions
57	-0.00927590	-5.82062426	49H—2Cl	Cl—H	
67	-0.00702618	-4.40892858	2Cl—30H	Cl—H	
81	-0.00807158	-5.06491522	37H—22H	H—H	
84	0.03006989	18.86885390	12C—11H	C—H	
92	-0.01062617	-6.66792356	29H—50F	F—H	
95	-0.00546426	-3.42882261	13C—17H	C—H	
107	-0.01062617	-6.66792356	18H—8O	O—H	
109	-0.29543965	-185.38837943	23C—24H	C—H	known bonds
110	-0.24781662	-155.50493037	23C—40C	C—C	
112	-0.28568927	-179.27001887	23C—25H	C—H	
75	-0.04564762	-28.64388112	2Cl—1Ga	Ga-Cl	Bond s aroun
77	-0.00935059	-5.86749347	6N—1Ga	Ga-N	
85	-0.06246371	-39.19597996	15N—1Ga	Ga-N	

87	-0.14485278	-90.89512158	3O—1Ga	Ga-O	d Ga
91	-0.16862073	-105.80951008	1Ga—50F	Ga-F	
98	-0.00967920	-6.07369885	5N—1Ga	Ga-N	
103	-0.10333722	-64.84410317	8O—1Ga	Ga-O	

CP	Atom A	Atom B	Bond Density ρ	$\nabla^2 \rho$	A-B Distance / Å	Formal Bond?	Sum of vdW radii[45] Å	Computed Mayer Bond Order
54	46H	2Cl	0.02317432	0.07093214	2.31979	N	2.95	0.32617888
57	49H	2Cl	0.01666439	0.05116997	2.50423	N	2.95	0.16604075
67	2Cl	30H	0.01373727	0.04136317	2.58505	N	2.95	0.20267053
81	37H	22H	0.01367336	0.04860599	2.04529	N	2.4	0.02568651
84	12C	11H	0.00825694	-0.00454289	2.61470	N	2.9	-0.01615418
92	29H	50F	0.01489194	0.05280006	2.30753	N	2.67	0.02340955
95	13C	17H	0.01026754	0.03544265	2.62409	N	2.9	0.04591670
107	18H	8O	0.01847066	0.06544011	2.23781	N	2.72	0.01903035
109	23C	24H	0.26968841	-0.87679720	1.10264	Y	2.9	0.99578314
110	23C	40C	0.24312947	-0.53157927	1.53688	Y	3.4	1.00586963
112	23C	25H	0.26346357	-0.82822678	1.11195	Y	2.9	0.95146627
75	2Cl	1Ga	0.04952886	0.07774275	2.48950	Y	3.62	0.62693307
77	6N	1Ga	0.01645853	0.03682022	2.93406	Y	3.42	0.16306817
85	15N	1Ga	0.05896616	0.11618510	2.23750	Y	3.42	0.06763517
87	3O	1Ga	0.09565738	0.40635718	1.91713	Y	3.39	0.65297909
91	50F	1Ga	0.10302024	0.55220835	1.84469	Y	3.34	0.87729119
98	5N	1Ga	0.01612116	0.03990165	2.93644	Y	3.42	0.04768123
103	8O	1Ga	0.07560080	0.27820077	2.01465	Y	3.39	0.22835456

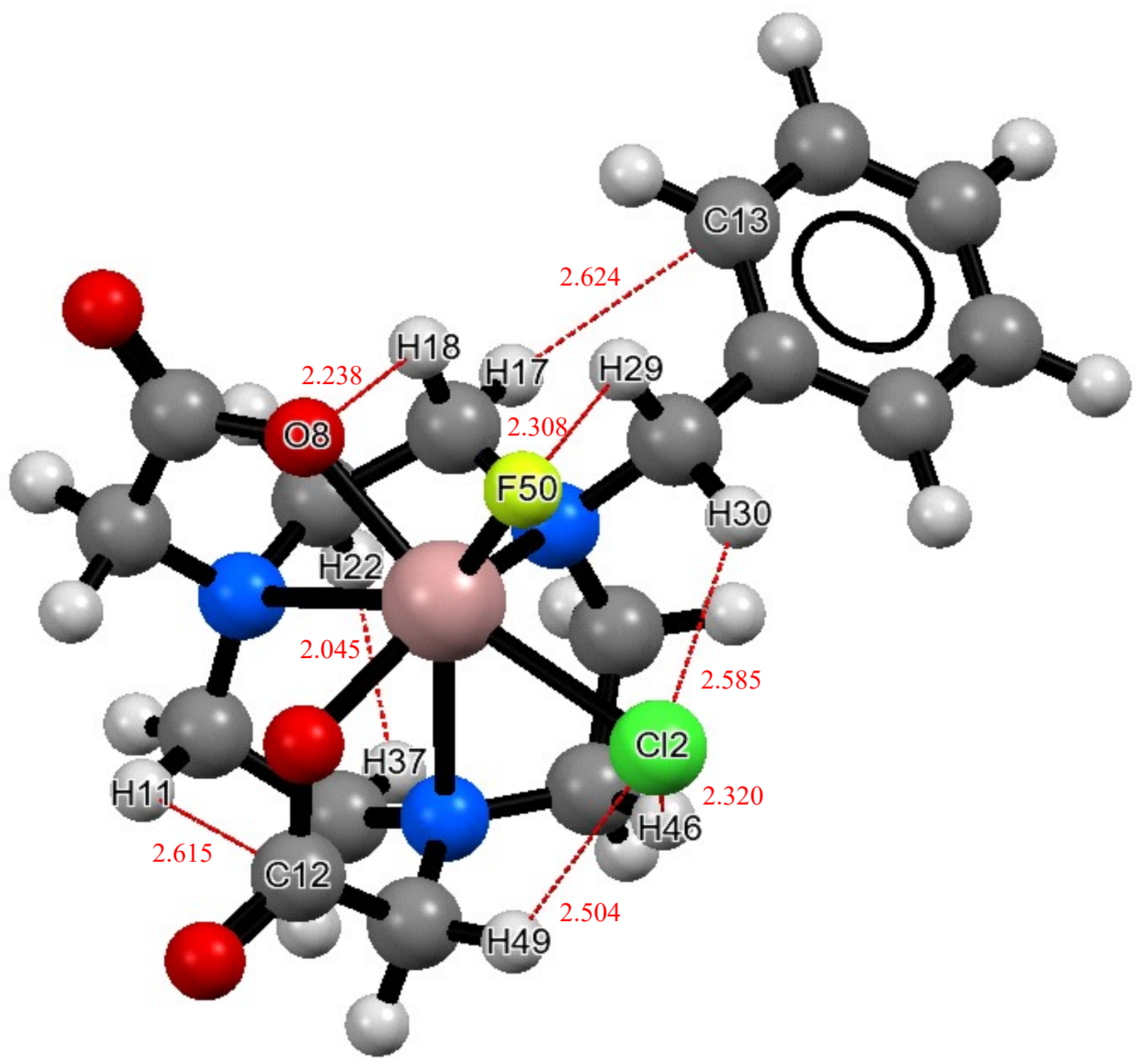

9f) Gas-phase Calculations – Gallium Transition State 2 (**TS2**)

Table for Hydrogen Bond Strengths using TS2 /Bp86-D3 6-311++G(d,p) MULTIWFN outputs—using calculated PEDs (Potential Energy Densities, V(r))

MultiWfn identifies 113 CPS. System has 50 Atoms, 54 “formal bonds” and 113 CPs (Ga-Cl not being formal bond)

i.e. expect 9 “non-bonded interactions” 3 Cl···H, 1 O···H, 1 Ga···Cl, 1 F···H, 1 H···H, 2 C···H

CP No.	V(r)/au	V(r) /kcal mol^{-1}	Gaussian Atom numbering	Bond Type	
53	-0.017882	-11.220926	46H-2Cl	H-Cl	Non-bonded interactions
55	-0.011074	-6.948715	49H-2Cl	H-Cl	
64	-0.008383	-5.260091	2Cl-30H	H-Cl	
74	-0.015792	-9.909322	1Ga-2Cl	Ga-Cl	
82	-0.008166	-5.124101	37H-22H	H-H	
84	-0.005045	-3.165906	12C-11H	C-H	
90	-0.009900	-6.212476	50F-29H	H-F	
95	-0.004996	-3.134772	13C-17H	C-H	
107	-0.011965	-7.507848	18H-8O	O-H	
112	-0.287846	-180.623056	23C—25H	C—H	known bonds
111	-0.247995	-155.616896	23C—40C	C—C	
109	-0.295576	-185.473709	23C—24H	C—H	
77	-0.01721372475	-10.8017672	1Ga-6N	Ga-N	Bonds around Ga
85	-0.07057563774	-44.2868479	1Ga-15N	Ga-N	
87	-0.1460540638	-91.6502395	1Ga-3O	Ga-O	

89	-0.177839	-111.594064	1Ga—50F	Ga-F	
96	-0.01803060318	-11.3143658	1Ga-5N	Ga-N	
102	-0.09793173959	-61.453048	1Ga-8O	Ga-O	

CP	Atom A	Atom B	Bond Density ρ	$\nabla^2 \rho$	A-B distance Å	Formal Bond?	Sum of vdW radii[45] Å	Computed Mayer Bond Order
53	46H	2Cl	0.0270955	0.0791296	2.23946	N	2.95	0.38872637
55	49H	2Cl	0.0195672	0.0574536	2.40805	N	2.95	0.23419441
64	2Cl	30H	0.0162936	0.0472185	2.48304	N	2.95	0.20377155
74	1Ga	2Cl	0.0255373	0.0479714	2.84699	N	3.62	0.26074520
82	37H	22H	0.0138738	0.0490552	2.03565	N	2.40	0.02781277
84	12C	11H	0.0088468	0.0325229	2.59407	N	2.9	-0.01911593
90	50F	29H	0.0136103	0.0503212	2.38202	N	2.67	0.01515260
95	13C	17H	0.0093374	0.0328778	2.68431	N	2.9	0.03615112
107	18H	8O	0.0179416	0.0629060	2.25671	N	2.72	0.00681421
109	23C	24H	0.2699711	-0.8783848	1.10241	Y	2.9	1.00249609
111	23C	40C	0.2430812	-0.5306499	1.53724	Y	3.4	0.98817103
112	23C	25H	0.2648844	-0.8380086	1.10957	Y	2.9	0.95617397
77	1Ga	6N	0.0260148	0.0532988	2.68440	Y	3.42	0.15830323
85	1Ga	15N	0.0639781	0.1295773	2.19794	Y	3.42	0.07211333
87	1Ga	3O	0.0966100	0.4064778	1.91631	Y	3.39	0.65030122
89	1Ga	50F	0.1067176	0.5861026	1.83033	Y	3.34	0.87614830
96	1Ga	5N	0.0257264	0.0576437	2.67428	Y	3.42	0.03644712
102	1Ga	8O	0.0733729	0.2575517	2.03425	Y	3.39	0.25170428

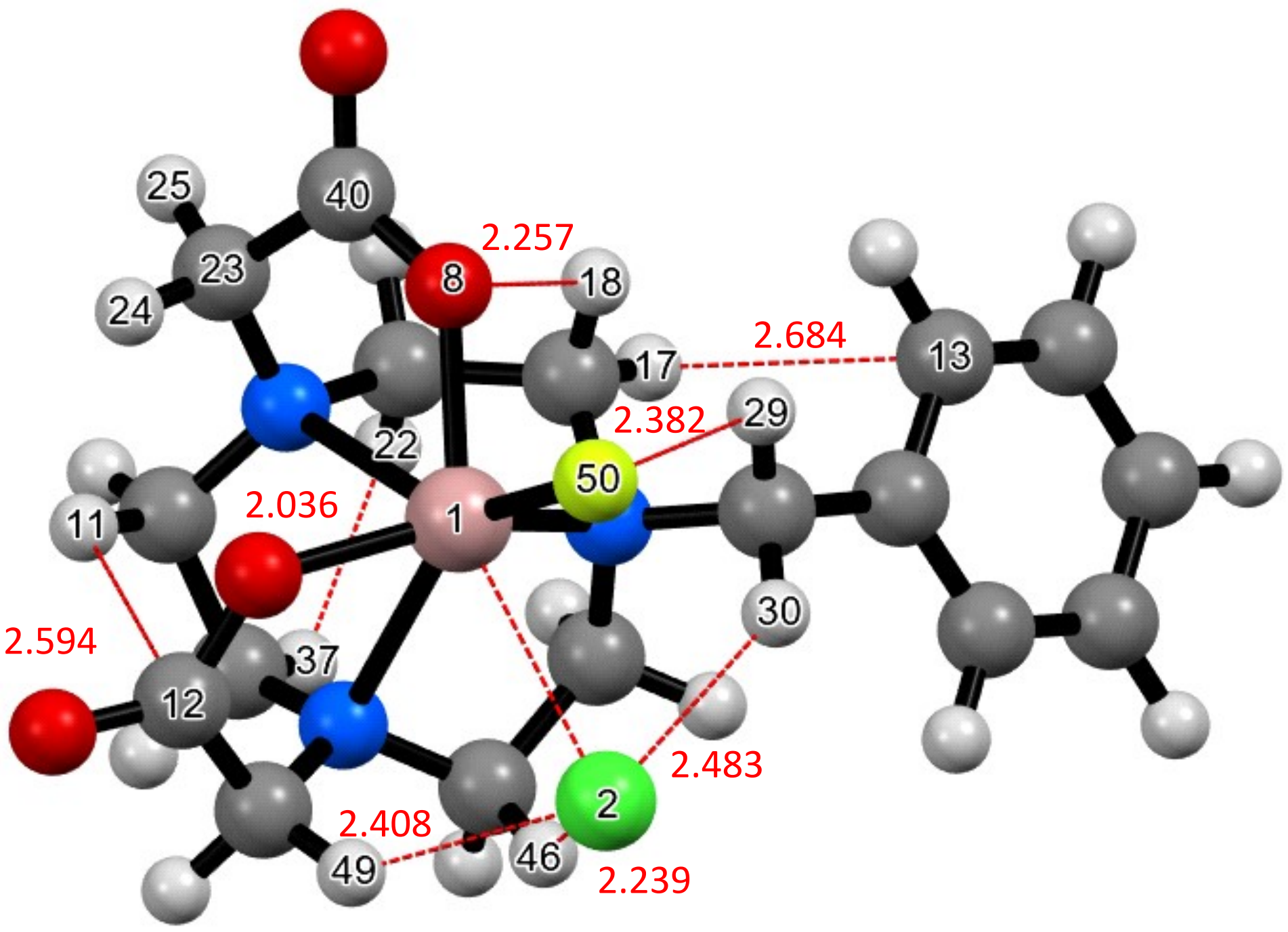

9g) Gas-phase Calculations – Gallium Product Complex (**PC**)

Table for Hydrogen Bond Strengths using PC /Bp86-D3 6-311++G(d,p) gas phase MULTIWFN outputs—using calculated PEDs (Potential Energy Densities, V(r))

MultiWfn identifies 109 CPS. System has 50 Atoms, 54 "formal bonds" and 109 CPs i.e. expect 5 "non-bonded interactions" 2 O···H, 1 H···H, 2 Cl···H

CP No.	V(r)/au	V(r) /kcal mol^{-1}	Gaussian Atom numbering	Bond Type	
59	-0.007567714365	-4.74874076	18H – 8O	O-H	Non-bonded interactions
76	-0.009016159818	-5.65764029	11H-3O	O-H	
101	-0.005865149129	-3.68038108	42H- 27H	H -H	
107	-0.01290898930	-8.10039079	42H-2Cl	H-Cl	
109	-0.008771416198	-5.50406366	27H-2Cl	H-Cl	
52	-0.2935125321	-184.179114	23C—25H	C—H	known bonds
53	-0.2356546216	-147.873275	23C—40C	C—C	
54	-0.2961599277	-185.840355	23C—24H	C—H	
84	-0.1725877932	-108.29884	1Ga—50F	Ga-F	Bonds around Ga
72	-0.06684792030	- 41.9476716	1Ga-N5	Ga-N	
86	-0.08294989875	-52.051808	1Ga-6N	Ga-N	
79	-0.07797101871	-48.927516	1Ga-15N	Ga-N	
78	-0.1178573939	- 73.9565754	1Ga-3O	Ga-O	
70	-0.1327401845	- 83.2956604	1Ga-8O	Ga-O	

CP	Atom A	Atom B	Bond Density ρ	$\nabla^2 \rho$	A-B distance Å	Formal Bond?	Sum of vdW radii[45] Å	Computed Mayer Bond Order
59	8O	18H	0.01153713488	0.03933545787	2.53420	N	2.72	-0.00653930
76	3O	11H	0.01361435070	-	2.44022	N	2.72	-0.00955364
101	27H	42H	0.01093627367	0.03536820696	2.04975	N	2.4	0.03004426
107	2Cl	42H	0.02277567847	0.06164312511	2.31818	N	2.95	0.81668508
109	2Cl	27H	0.01773122329	0.04746115943	2.42693	N	2.95	0.52803982
52	23C	25H	0.2694310298	-0.08694417703	1.10318	Y	2.9	0.97979630
53	23C	40C	0.2363922028	-0.4965798426	1.55196	Y	3.4	0.93247013
54	23C	24H	0.2714714303	-0.8888763989	1.10142	Y	2.9	1.00155868
84	1Ga	50F	0.1036475316	0.5678296267	1.84138	Y	3.62	0.68506237
72	1Ga	5N	0.05973720857	0.1284245687	2.23484	Y	3.42	0.17602926
86	1Ga	6N	0.06935753979	0.1565830455	2.16075	Y	3.42	0.21987081
79	1Ga	15N	0.06738858071	0.1446967429	2.17875	Y	3.42	0.11659640
78	1Ga	O3	0.08383204416	0.3095388662	1.98990	Y	3.39	0.52230256
70	1Ga	O8	0.09044706184	0.3593982712	1.95219	Y	3.39	0.54264634

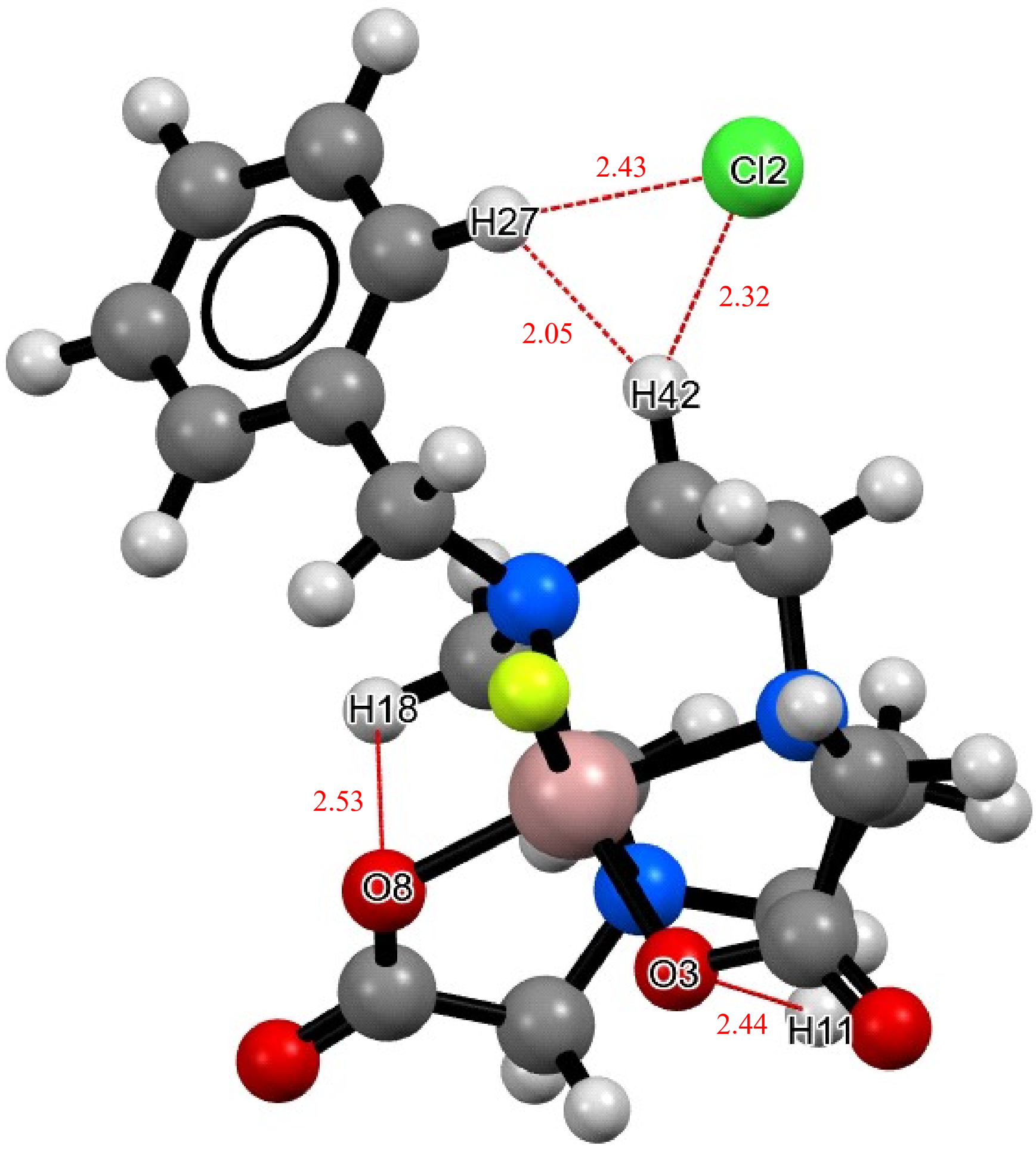

9h) Solution-phase Calculations – Gallium Reaction Complex (**RC**)

Table for Hydrogen Bond Strengths using RC - BP86-D3/6-311++G(d,p) SMD=water MULTIWFN outputs—using calculated PEDs (Potential Energy Densities, V(r))

MultiWfn identifies 110 CPS.

System has 50 Atoms, 54 “formal bonds” and 110 CPs

i.e. expect 6 “non-bonded interactions” 1 Cl···H, 1 O···H, 1 C···H, 2 H···F, 1 F···O

CP No.	V(r)/au	V(r) /kcal mol^{-1}	Gaussian Atom numbering	Bond Type	
59	-0.005300	-3.325975	26C—42H	C—H	Non-bonded interactions
75	-0.007164	-4.495194	2Cl—30H	Cl—H	
94	-0.008065	-5.061077	3O—11H	O—H	
103	-0.013489	-8.464524	50F—14H	F—H	
104	-0.012670	-7.950556	50F—18H	F—H	
110	-0.001287	-0.807438	50F—8O	F—O	
107	-0.295964	-185.717410	23C—25H	C—H	known bonds
106	-0.251789	-157.997598	23C—40C	C—C	
105	-0.294516	-184.808790	23C—24H	C—H	
83	-0.078469	-49.239298	1Ga—2Cl	Ga-Cl	Bonds around Ga
77	-0.091855	-57.638963	6N-1Ga	Ga-N	
82	-0.083259	-52.245070	15N-1Ga	Ga-N	
96	-0.085809	-53.845142	5N-1Ga	Ga-N	
92	-0.097264	-61.033056	3O-1Ga	Ga-O	
101	-0.120355	-75.523075	4O-1Ga	Ga-O	

Note: No Ga-F bond found

CP	Atom A	Atom B	Bond Density ρ	$\nabla^2 \rho$	A-B distance Å	Formal Bond?	Sum of vdW radii[45] Å	Computed Mayer Bond Order
59	42H	26C	0.010203	0.034804	2.56036	N	2.9	0.15471143
75	30H	2Cl	0.012758	0.043357	2.66415	N	2.95	0.16822819
94	11H	3O	0.012133	0.041720	2.49660	N	2.72	0.00958116
103	14H	50F	0.019367	0.059737	2.09571	N	2.67	0.00377831
104	18H	50F	0.018345	0.056559	2.12879	N	2.67	0.04890683
110	8O	50F	0.002783	0.010052	3.65071	N	2.99	-
107	23C	25H	0.272952	-0.898527	1.53267	Y	2.9	1.01438587
106	23C	40C	0.245104	-0.542237	1.10012	Y	3.4	0.82437416
105	23C	24H	0.270986	-0.889459	1.10170	Y	2.9	0.97891048
83	1Ga	2Cl	0.0701373	0.115000	2.30980	Y	3.62	-
77	6N	1Ga	0.075247	0.171036	2.12674	Y	3.42	0.33545356
82	15N	1Ga	0.071409	0.150822	2.15510	Y	3.42	0.27105619
96	5N	1Ga	0.071760	0.158463	2.15070	Y	3.42	0.24074369
92	3O	1Ga	0.073519	0.247605	2.04872	Y	3.39	0.83761983
101	4O	1Ga	0.084829	0.320481	1.98120	Y	3.39	0.66334410

Note: No Ga-F bond found

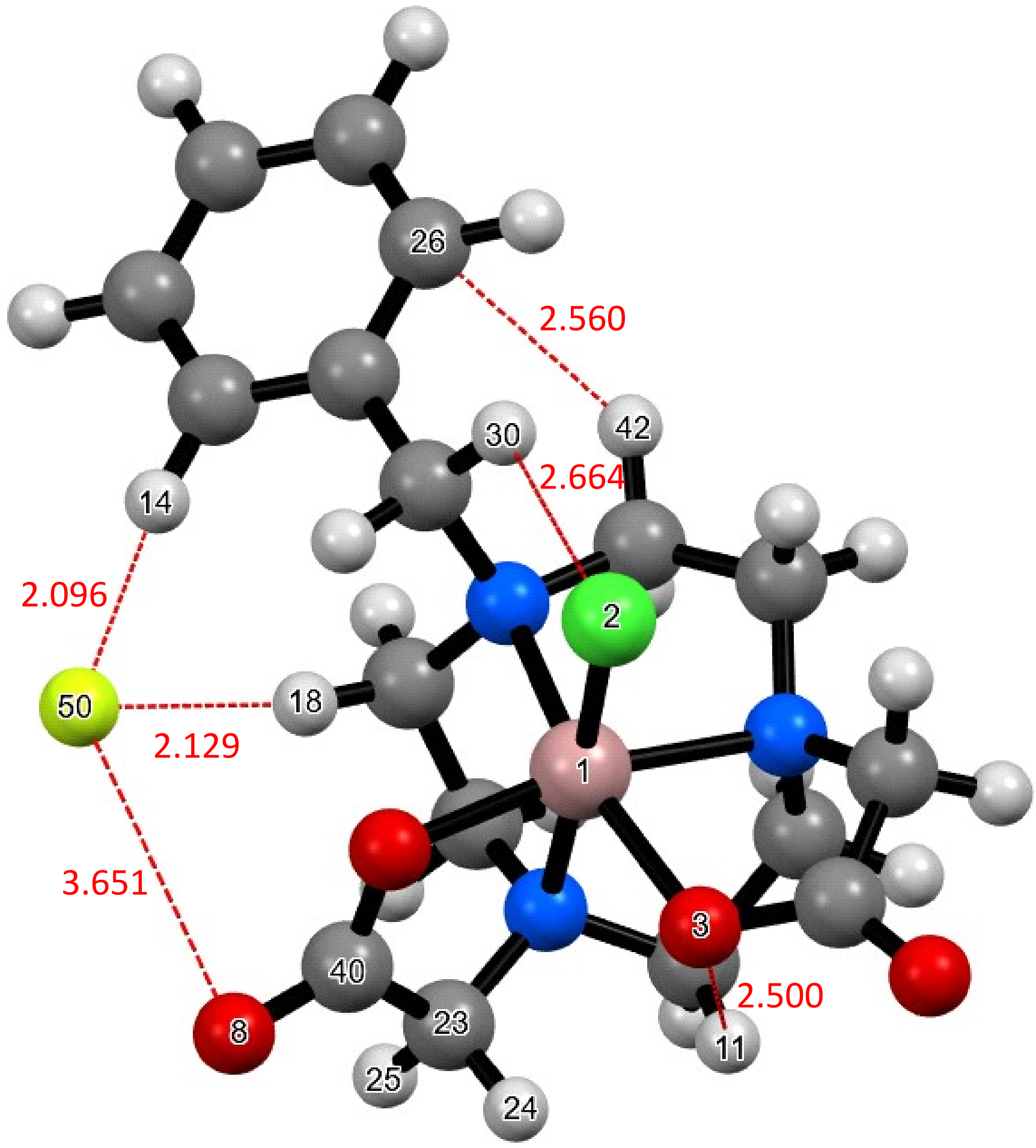
26
2.560
42
30
2.664
14
2
2.096
50
18
2.129
1
3.651
3
40
2.500
8
23
11
25
24

9i) Solution-phase Calculations – Gallium Transition State 1(**TS1**)

Table for Hydrogen Bond Strengths using TS1 /Bp86-D3 6-311++G(d,p) SMD=water MULTIWFN outputs—using calculated PEDs (Potential Energy Densities, V(r))

MultiWfn identifies 112 CPS. System has 50 Atoms, 54 "formal bonds" and 112 CPs
i.e. expect 8 "non-bonded interactions" 2 Cl···H, 1 O···H, 1 Ga···F, 1 H···H, 2 F···H, 1 O···F

CP No.	V(r)/au	V(r) /kcal mol^{-1}	Gaussian Atom numbering	Bond Type	
67	-0.004450	-2.792141	49H--2Cl	H—Cl	Non-bonded interactions
74	-0.008814	-5.530648	43H—22H	H—H	
78	-0.005902	-3.703689	30H--2Cl	H—Cl	
85	-0.008808	-5.527291	3O—11H	O—H	
103	-0.009913	-6.220163	1Ga—50F	Ga—F	
104	-0.020526	-12.880261	29H—50F	F—H	
106	-0.018700	-11.733939	18H—50F	F—H	
107	-0.012631	-7.925831	4O—50F	F—O	
111	-0.297206	-186.496675	23C—25H	C—H	known bonds
109	-0.254921	-159.962744	23C—40C	C—C	
108	-0.295515	-185.435880	23C—24H	C—H	
86	-0.086141	-54.053224	1Ga—2Cl	Ga-Cl	Bonds around Ga
77	-0.078661	-49.359710	6N—1Ga	Ga-N	
83	-0.065963	-41.391809	15N—1Ga	Ga-N	
95	-0.112044	-70.307514	5N—1Ga	Ga-N	
88	-0.034551	-21.680453	3O—1Ga	Ga-O	
99	-0.112044	-70.308591	4O—1Ga	Ga-O	

CP	Atom A	Atom B	Bond Density ρ	$\nabla^2 \rho$	A-B distance Å	Formal Bond?	Sum of vdW radii[45] Å	Computed Mayer Bond Order
67	49H	2Cl	0.008079	0.028096	2.95869	N	2.85	0.04069376
74	43H	22H	0.014528	0.054083	1.99754	N	2.20	0.00321976
78	30H	2Cl	0.010884	0.036942	2.75155	N	2.85	0.18404442
85	3O	11H	0.013797	0.045364	2.38469	N	2.62	0.02120904
103	1Ga	50F	0.014493	0.040148	2.82606	N	3.34	0.27383736
104	29H	50F	0.026124	0.089659	1.97267	N	2.57	0.02074073
106	18H	50F	0.024262	0.082598	2.01726	N	2.57	0.00894444
107	4O	50F	0.015429	0.053710	2.71108	N	2.99	-0.02523407
108 *	23C	24H	0.272153	-0.893326	1.10043	Y	2.80	1.01321120
111	23C	25H	0.272322	-0.896480	1.10005	Y	2.80	0.94440980
109 *	23C	40C	0.247080	-0.551620	1.52798	Y	3.40	0.85609282
77‡	6N	1Ga	0.068699	0.143701	2.17249	Y	3.42	0.33592703
83‡	15N	1Ga	0.062377	0.116639	2.22833	Y	3.42	0.48642837
86 †	1Ga	2Cl	0.075361	0.123818	2.27269	Y	3.62	-0.09037007
95‡	5N	1Ga	0.073480	0.155559	2.14159	Y	3.42	0.36499392
88‡	3O	1Ga	0.037059	0.093826	2.38631	Y	3.39	0.46632052
99‡	4O	1Ga	0.081561	0.294962	1.99758	Y	3.39	0.64384995

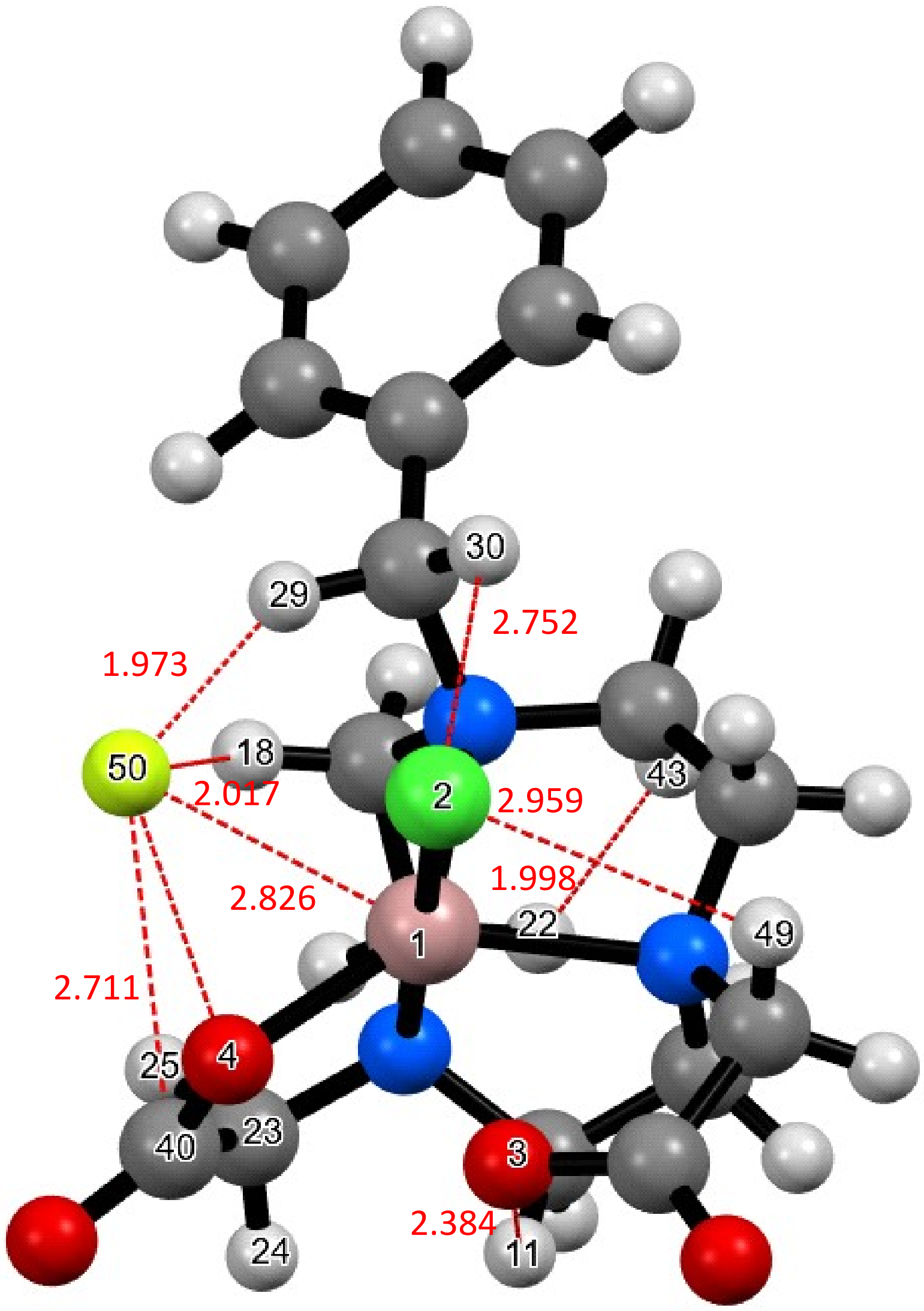

9j) Solution-phase Calculations – Gallium Ionic Complex 1 (**IC1**)

Table for Hydrogen Bond Strengths using IC1 /Bp86-D3 6-311++G(d,p) SMD=water MULTIWFN outputs—using calculated PEDs (Potential Energy Densities, V(r))

MultiWfn identifies 115 CPS.

System has 50 Atoms, 54 “formal bonds” and 115 CPs

i.e. expect 11 “non-bonded interactions” 3 Cl···H, 2 O···H, 1 C···H, 2 H···F, 1 F···C, 1 H···H, 1 O···O

CP No.	V(r)/au	V(r) /kcal mol^{-1}	Gaussian Atom numbering	Bond Type	
63	-0.00568160	-3.56520421	42H – 26C	C—H	Non-bonded interactions
64	-0.00650156	-4.07972852	49H – 2Cl	Cl—H	
65	-0.00497257	-3.12028683	46H – 2Cl	Cl—H	
69	-0.00902929	-5.66587645	37H – 43H	H—H	
75	-0.00832402	-5.22332426	36H – 3O	H—O	
79	-0.00470062	-2.94963622	30H – 2Cl	Cl—H	
86	-0.00823751	-5.16903918	3O – 11H	O—H	
89	-0.00384351	-2.41180136	3O – 4O	O—O	

101	-0.00928571	-5.82678511	29H – 50F	F—H	
107	-0.01029917	-6.46272753	18H – 50F	F—H	
111	-0.00920047	-5.77329286	50F – 23C	F—C	
115	-0.29658882	-186.10948681	23C—25H	C—H	known bonds
112	-0.24737076	-155.22515259	23C—40C	C—C	
114	-0.29455366	-184.83242159	23C—24H	C—H	
103	-0.13021826	-81.71195570	1Ga-50F	Ga-F	Bonds around Ga
80	-0.07153445	-44.88786937	6N-1Ga	Ga-N	
88	-0.08030033	-50.38845608	15N-1Ga	Ga-N	
96	-0.08793411	-55.17865705	5N-1Ga	Ga-N	
100	-0.09913031	-62.20426707	4O-1Ga	Ga-O	

(No second Ga-O bond/interaction)

CP	Atom A	Atom B	Bond Density ρ	$\nabla^2 \rho$	A-B distance Å	Formal Bond?	Sum of vdW radii[45] Å	Computed Mayer Bond Order
63	42H	26C	0.01093597	0.03733277	2.52600	N	2.80	0.18110760
64	49H	2Cl	0.01208102	0.03895562	2.67683	N	2.85	0.16340705
65	46H	2Cl	0.00909735	0.02959057	2.87077	N	2.85	0.07986662
69	37H	43H	0.01463857	0.05581567	1.99871	N	2.20	-
75	36H	3O	0.01260929	0.04165719	2.51726	N	2.62	0.00222272
79	30H	2Cl	0.00916608	0.02989350	2.84187	N	2.85	0.16785816
86	3O	11H	0.01342248	0.04257853	2.36986	N	2.62	0.00829318
89	3O	4O	0.00612046	0.02024719	3.25456	N	3.04	0.02672824
101	29H	50F	0.01297914	0.04585293	2.35344	N	2.57	-
107	18H	50F	0.01382434	0.05037647	2.36277	N	2.57	-
111	50F	23C	0.01177437	0.04702902	2.92869	N	3.17	0.07365390
115	23C	25H	0.27274814	-0.29658882	1.09966	Y	2.80	0.99176132
112	23C	40C	0.24329267	-0.53479725	1.53749	Y	3.40	0.75866917
114	23C	24H	0.27171259	-0.90108705	1.10178	Y	2.80	1.01430919
80	6N	1Ga	0.06479759	0.12922747	2.20394	Y	3.42	0.25713459
88	15N	1Ga	0.06917627	0.14841946	2.16657	Y	3.42	0.30632747
96	5N	1Ga	0.07280694	0.16363890	2.14124	Y	3.42	0.27349847
100	4O	1Ga	0.07456139	0.25482251	2.03950	Y	3.39	0.70874396
103	1Ga	50F	0.08589907	0.40637130	1.91963	Y	3.34	1.09879349

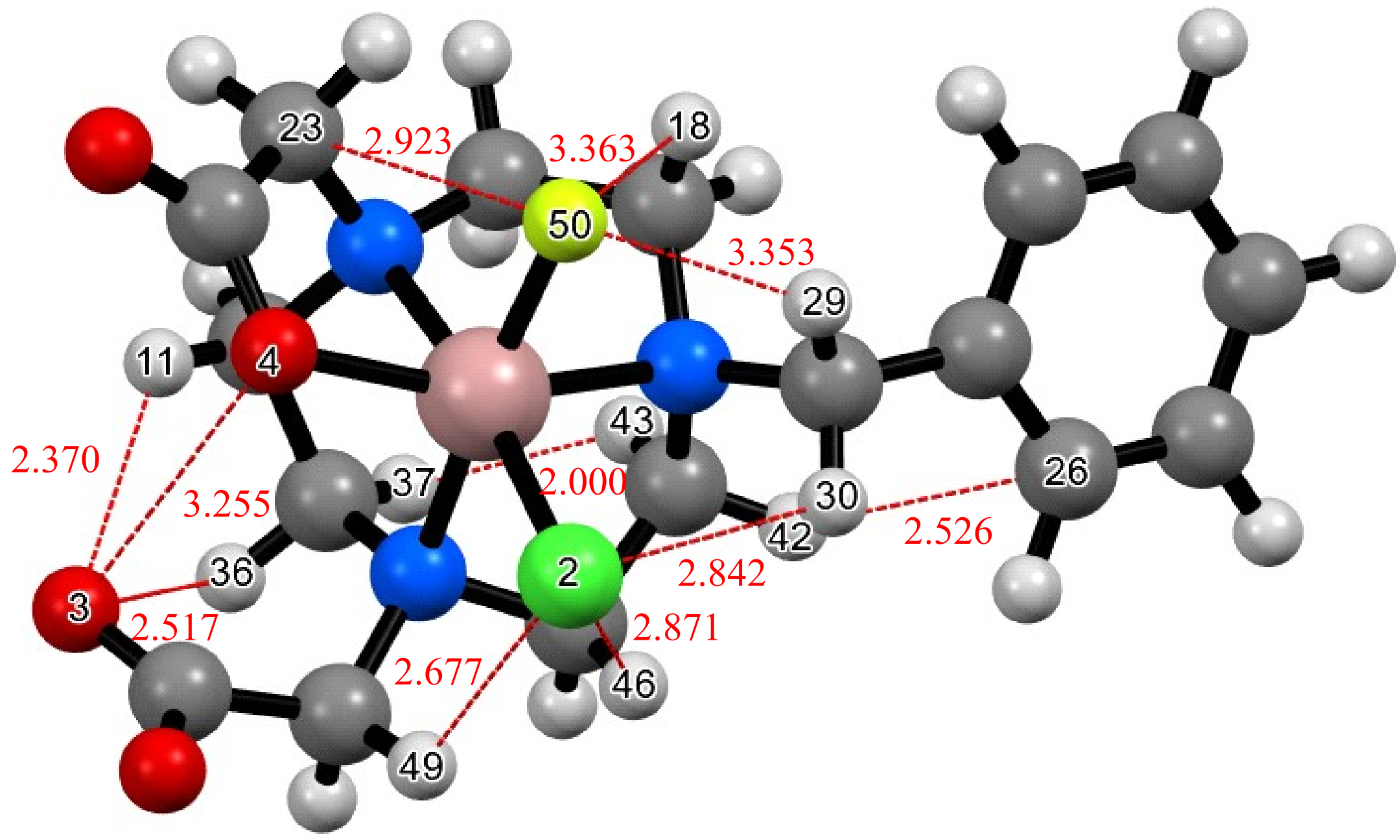

9k) Solution-phase Calculations – Gallium Ionic Complex transition state (**ICts**)

Table for Hydrogen Bond Strengths using ICts /Bp86-D3 6-311++G(d,p) SMD=water MULTIWFN outputs—using calculated PEDs (Potential Energy Densities, V(r))

MultiWfn identifies 113 CPS.

System has 50 Atoms, 55 "formal bonds" and 113 CPs

i.e. expect 8 "non-bonded interactions" 3 Cl···H, 1 O···H, 1 H···H, 2 F···H, 1 O···O

CP No.	V(r)/au	V(r) /kcal mol^{-1}	Gaussian Atom numbering	Bond Type	
60	-0.00510773	-3.20510116	49H-2Cl	Cl—H	Non-bonded interactions
62	-0.00412031	-2.58549179	46H-2Cl	Cl—H	
74	-0.00512735	-3.21740899	30H-2Cl	Cl—H	
79	0.05367182	33.67906716	43H-22H	H—H	
85	-0.00735492	-4.61521439	3O-11H	O—H	
97	-0.01875377	-11.76799185	3O-4O	O—O	
100	-0.01232439	-7.73355579	29H-50F	F—H	
106	-0.01044035	-6.55131744	50F-18H	F—H	
110	-0.25250533	-158.44709338	23C—40C	C—C	known bonds
111	-0.29508995	-185.16894262	23C—24H	C—H	
112	-0.29694202	-186.33111699	23C—25H	C—H	
76	-0.05748126	-36.06949254	6N-1Ga	Ga-N	Bonds around Ga
82	-0.06793610	-42.62990287	2Cl-1Ga	Ga-Cl	
83	-0.05432580	-34.08943875	3O-1Ga	Ga-O	
84	-0.04199102	-26.34936512	15N-1Ga	Ga-N	
94	-0.08140956	-51.08450146	5N-1Ga	Ga-N	
98	-0.12894877	-80.91535368	1Ga-50F	Ga-F	

102	-0.03463307	-21.73224982	4O-1Ga	Ga-O	

CP	Atom A	Atom B	Bond Density ρ	$\nabla^2 \rho$	A-B distance Å	Forma lBond ?	Sum of vdW radii[45] Å	Computed Mayer Bond Order
60	49H	2Cl	0.00941770	0.03135510	2.87758	N	2.85	0.06575202
62	46H	2Cl	0.00791307	0.02502434	2.93352	N	2.85	0.12017954
74	30H	2Cl	0.00989923	0.03221272	2.79542	N	2.85	0.18557051
79	43H	22H	0.01450699	-0.00872149	1.99776	N	2.20	0.00909424
85	3O	11H	0.01155535	0.03744480	2.49405	N	2.62	0.00713937
97	3O	4O	0.02215600	0.09957440	2.49176	N	3.04	-
100	29H	50F	0.01673192	0.05905023	2.21098	N	2.57	-
106	50F	18H	0.01412501	0.05112540	2.33642	N	2.57	-
110	23C	40C	0.24606976	-0.54853726	1.53003	Y	3.40	0.82123714
111	23C	24H	0.27150873	-0.88611083	1.10197	Y	2.80	1.03377814
112	23C	25H	0.27164211	-0.89034707	1.10120	Y	2.80	0.96072892
76	6N	1Ga	0.05640175	0.10650169	2.27564	Y	3.42	0.30111001
82	2Cl	1Ga	0.06403902	0.10095968	2.35305	Y	3.62	0.30098325
83	3O	1Ga	0.05050507	0.13291103	2.22684	Y	3.39	0.51031368
84	15N	1Ga	0.04569976	0.08691416	2.37976	Y	3.42	0.21486755
94	5N	1Ga	0.07043936	0.14798470	2.15691	Y	3.42	0.25260496
98	50F	1Ga	0.08600304	0.40172166	1.91885	Y	3.34	1.03247431
102	4O	1Ga	0.03714792	0.09424056	2.37484	Y	3.39	0.48762331

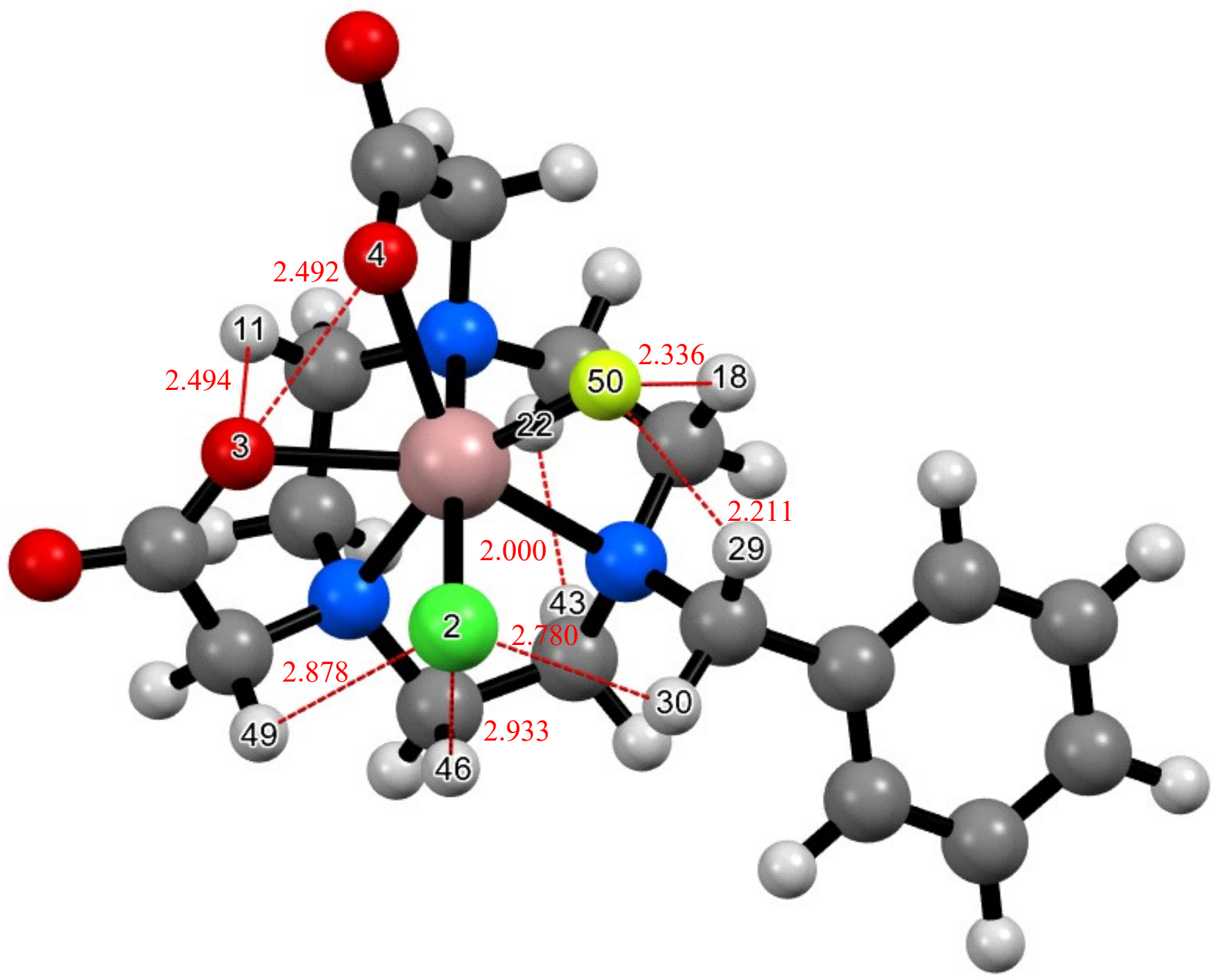

9I) Solution-phase Calculations – Gallium Ionic Complex 2 (**IC2**)

Table for Hydrogen Bond Strengths using IC2 /Bp86-D3 6-311++G(d,p) SMD=water MULTIWFN outputs—using calculated PEDs (Potential Energy Densities, V(r))

MultiWfn identifies 112 CPS.

System has 50 Atoms, 54 "formal bonds" and 112 CPs

i.e. expect 8 "non-bonded interactions" 3 Cl···H, 3 O···H, 1 C···H, 1 F···C

CP No.	V(r)/au	V(r) /kcal mol^{-1}	Gaussian Atom numbering	Bond Type	
58	-0.00597383	-3.74857897	46H-2Cl	Cl—H	Non-bonded interactions
61	-0.00532745	-3.34297683	42H-26C	C—H	
63	-0.00621054	-3.89711358	49H-2Cl	Cl—H	
73	-0.00721344	-4.52643571	30H-2Cl	Cl—H	
88	-0.00786561	-4.93567322	3O-11H	O—H	
106	-0.00809039	-5.07672134	18H-8O	O—H	
107	-0.00943903	-5.92299066	21H-8O	O—H	
108	-0.00748535	-4.69705728	50F-40C	F—C	
105	-0.30333378	-190.34194933	23C—24H	C—H	known bonds
109	-0.29058361	-182.34121772	23C—25H	C—C	
110	-0.23746135	-149.00699543	23C—40C	C—H	
77	-0.08458924	-53.07974597	6N-1Ga	Ga-N	Bonds around Ga
80	-0.06326945	-39.70158164	2Cl-1Ga	Ga-Cl	
84	-0.08174092	-51.29242542	15N-1Ga	Ga-N	
86	-0.10219509	-64.12742098	3O-1Ga	Ga-O	
94	-0.07878110	-49.43514214	5N-1Ga	Ga-N	
97	-0.15502328	-97.27710506	1Ga-50F	Ga-F	

Note: No second Ga-O bond/interaction

CP	Atom A	Atom B	Bond Density ρ	$\nabla^2 \rho$	A-B distance Å	Forma lBond ?	Sum of vdW radii[45] Å	Computed Mayer Bond Order
58	46H	2Cl	0.01124424	0.03502438	2.73500	N	2.85	0.13886445
61	42H	26C	0.01029037	0.03479036	2.55392	N	2.80	0.18509168
63	49H	2Cl	0.01122603	0.03670476	2.77175	N	2.85	0.06408662
73	30H	2Cl	0.01316609	0.04328202	2.63156	N	2.85	0.17372235
88	3O	11H	0.01187313	0.04073558	2.51056	N	2.62	0.01704098
106	18H	8O	0.01311332	0.04161737	2.39229	N	2.62	0.03395931
107	21H	8O	0.01446850	0.04746883	2.42279	N	2.62	0.00829202
108	50F	40C	0.01033350	0.04007757	2.84122	N	3.17	0.10356424
105	23C	24H	0.27651509	-0.91860463	1.09697	Y	2.80	0.98975555
109	23C	25H	0.26698970	-0.85615515	1.10803	Y	2.80	0.94320621
110	23C	40C	0.23842831	-0.51378008	1.54464	Y	3.40	0.87924281
77	6N	1Ga	0.07140751	0.15540994	2.15437	Y	3.42	0.32821908
80	2Cl	1Ga	0.06042372	0.09741268	2.38452	Y	3.62	0.42185516
84	15N	1Ga	0.07033539	0.14919395	2.16052	Y	3.42	0.27359650
86	3O	1Ga	0.07588576	0.26472715	2.03111	Y	3.39	0.56276705

94	5N	1Ga	0.06821993	0.14534165	2.17309	Y	3.42	0.17438136
97	50F	1Ga	0.09644594	0.50641583	1.86832	Y	3.34	0.95758043

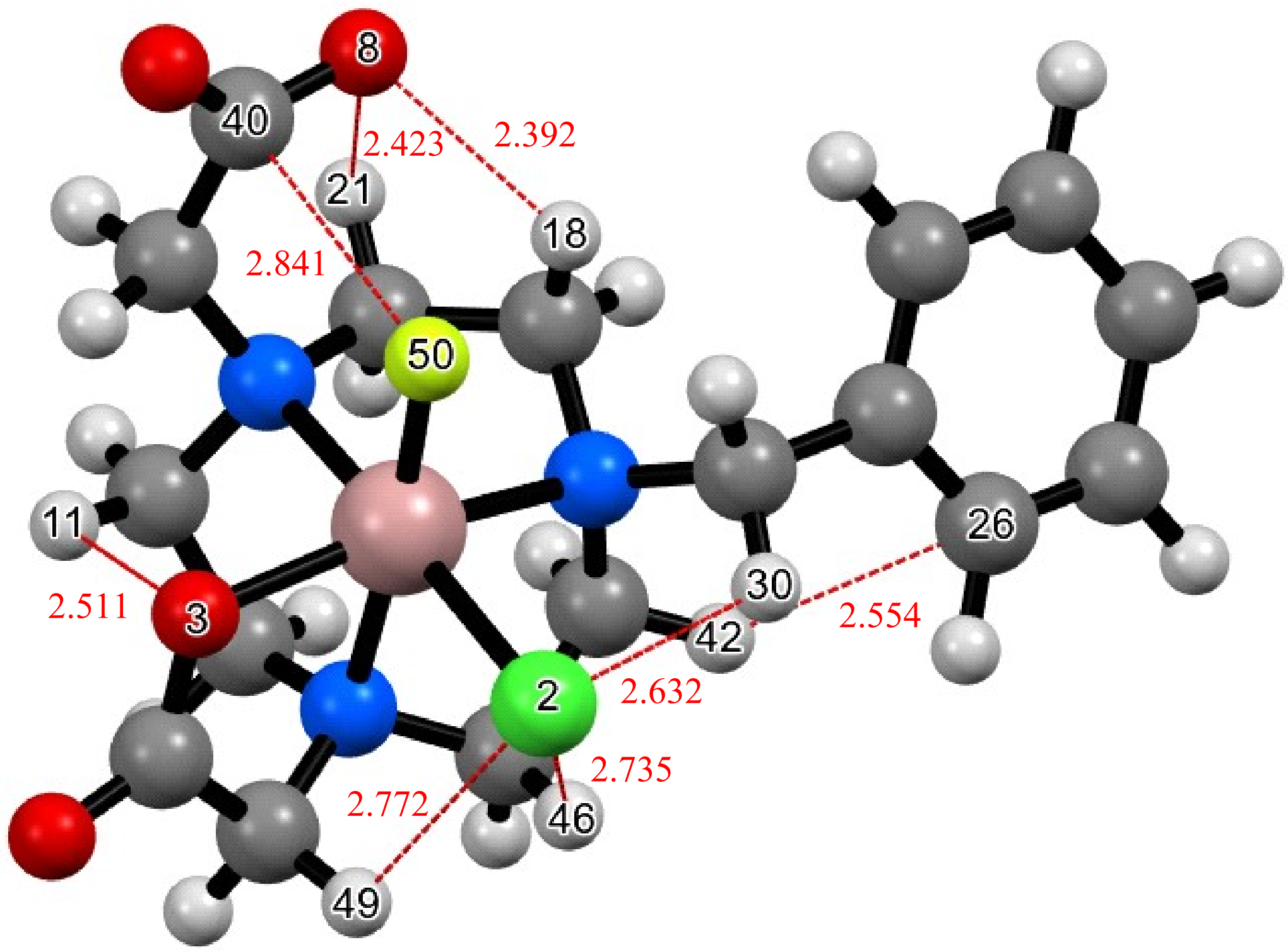

9m) Solution-phase Calculations – Gallium Transition State 2 (**TS2**)

Table for Hydrogen Bond Strengths using TS2b /Bp86-D3 6-311++G(d,p) SMD=water MULTIWFN outputs—using calculated PEDs (Potential Energy Densities, V(r))

MultiWfn identifies 111 CPS.

System has 50 Atoms, 53 "formal bonds" and 111 CPs

i.e. expect 8 "non-bonded interactions" 3 Cl···H, 1 O···H, 1 Ga···Cl, 1 F···Cl, 1 F···H, 1 O···F

CP No.	V(r)/au	V(r) /kcal mol^{-1}	Gaussian Atom numbering	Bond Type	
51	-0.001466	-0.920137	49H—2Cl	Cl—H	Non-bonded interactions
53	-0.008390	-5.264892	2Cl—46H	Cl—H	
63	-0.004077	-2.558576	2Cl—30H	Cl—H	
68	-0.001820	-1.141769	2Cl—1Ga	Cl—Ga	
73	-0.002027	-1.271902	2Cl—50F	Cl—F	
90	-0.008639	-5.420838	29H—50F	F—H	
103	-0.006825	-4.282942	50F—8O	F—O	
106	-0.010173	-6.383330	18H—8O	O—H	
108	-0.295732	-185.572035	23C—25H	C—H	known bonds
109	-0.245779	-154.226343	23C—40C	C—C	
107	-0.296785	-186.232391	23C—24H	C—H	
88	-0.178384	-111.935911	1Ga—50F	Ga-F	Bonds around Ga
78	-0.096770	-60.723355	6N—1Ga	Ga-N	
84	-0.091391	-57.347764	15N—1Ga	Ga-N	
92	-0.112580	-70.644123	5N—1Ga	Ga-N	

83	-0.116158	-72.889048	3O—1Ga	Ga-O	

Note: No second Ga-O bond/interaction.

CP	Atom A	Atom B	Bond Density ρ	$\nabla^2 \rho$	A-B distance Å	Formal Bond?	Sum of vdW radii[45] Å	Computed Mayer Bond Order
51	2Cl	49H	0.013707	0.038358	2.57866	N	2.85	0.18924382
53	2Cl	46H	0.016318	0.04613	2.47952	N	2.85	0.25852230
63	2Cl	30H	0.009322	0.024652	2.77252	N	2.85	0.25128272
68	2Cl	1Ga	0.004933	0.011523	3.75717	N	3.62	-0.15551383
73	2Cl	50F	0.004758	0.014053	3.63006	N	3.22	-0.03392074
90	50F	29H	0.011816	0.044577	2.43463	N	2.57	0.01387251
103	50F	8O	0.009422	0.030752	2.97611	N	2.99	0.02111590
106	8O	18H	0.016138	0.053878	2.26691	N	2.62	0.02264367
88	1Ga	50F	0.105908	0.596781	1.82958	Y	3.34	1.09780403
108	23C	25H	0.271973	-0.89001	1.10201	Y	2.80	0.94568492
109	23C	40C	0.242447	-0.53087	1.53713	Y	3.40	0.83523756
107	23C	24H	0.272319	-0.89605	1.10101	Y	2.80	0.96168756
78	6N	1Ga	0.078681	0.175522	2.10984	Y	3.42	0.34768207
84	15N	1Ga	0.076350	0.162878	2.12511	Y	3.42	0.31574825
92	5N	1Ga	0.086864	0.213904	2.05401	Y	3.42	0.31142288
83	3O	1Ga	0.083000	0.304896	1.99284	Y	3.39	0.56085644

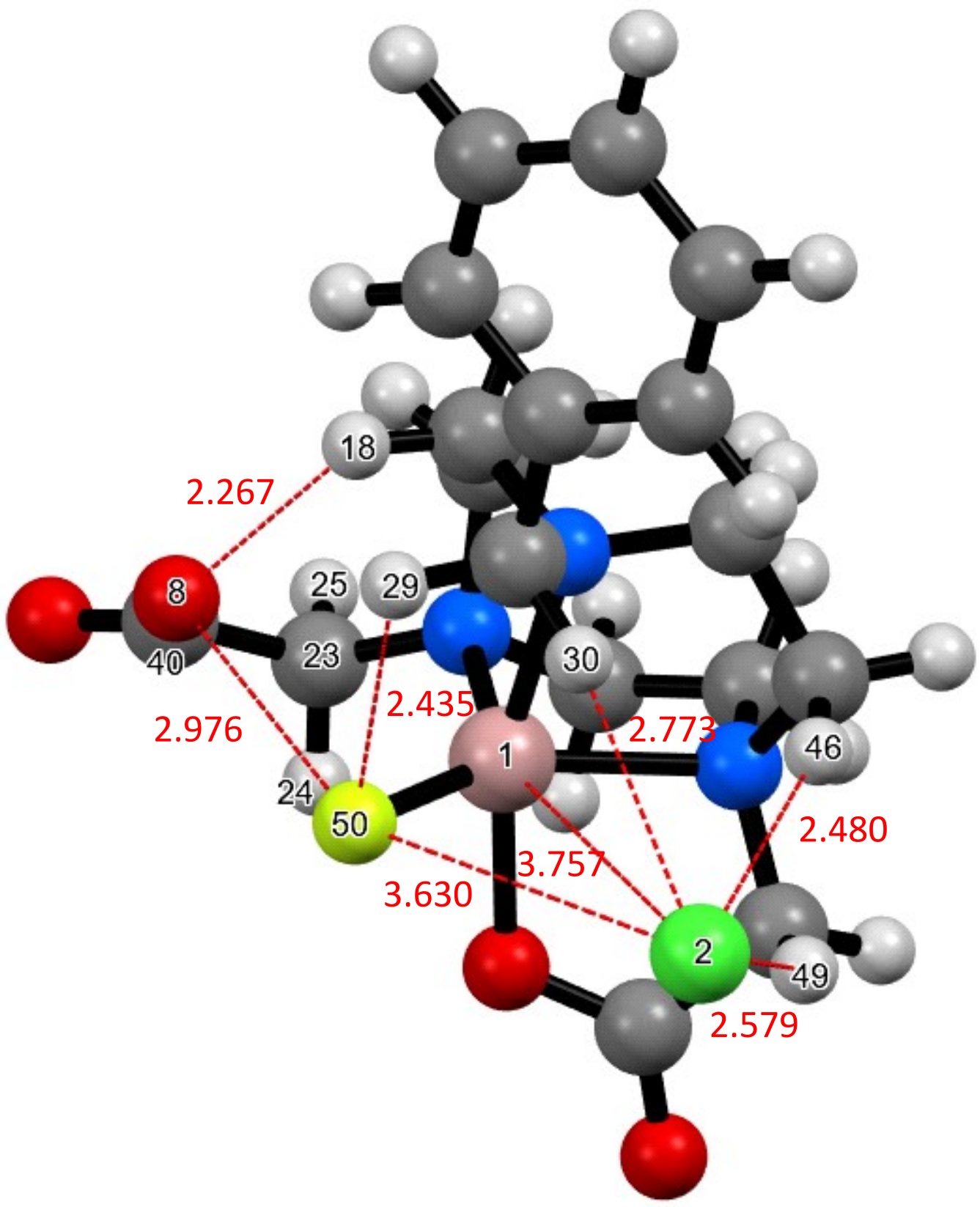

9n) Solution-phase Calculations – Gallium Product Complex (**PC**)

Table for Hydrogen Bond Strengths using PC /Bp86-D3 6-311++G(d,p) SMD=water MULTIWFN outputs—using calculated PEDs (Potential Energy Densities, V(r))

MultiWfn identifies 110 CPS.

System has 50 Atoms, 54 "formal bonds" and 110 CPs

i.e. expect 6 "non-bonded interactions" 3 Cl···H, 1 O···H, 1 C···H, 1 H···F

CP No.	V(r)/au	V(r) /kcal mol^{-1}	Gaussian Atom numbering	Bond Type	
75	-0.007316	-4.591076	11H—3O	O—H	Non-bonded interactions
92	-0.005415	-3.397620	42H—26C	C—H	
97	-0.008822	-5.535852	30H—50F	F—H	
107	-0.003087	-1.936946	30H—2Cl	Cl—H	
109	-0.005375	-3.372889	27H—2Cl	Cl—H	
110	0.031978	-20.065991	46H—2Cl	Cl—H	
51	-0.296044	-185.767815	23C—25H	C—H	known bonds
55	-0.250925	-157.455707	23C—40C	C—C	
54	-0.294145	-184.576209	23C—24H	C—H	
90	-0.153866	-96.550685	1Ga—50F	Ga-F	Bonds around Ga
71	-0.086324	-54.168234	5N-1Ga	Ga-N	
78	-0.086383	-54.205319	15N-1Ga	Ga-N	
88	-0.094826	-59.503297	6N-1Ga	Ga-N	
73	-0.118288	-74.225656	O3-1Ga	Ga-O	
82	-0.101215	-63.512412	O8-1Ga	Ga-O	

Note: No Ga-Cl bond located

CP	Atom A	Atom B	Bond Density ρ	$\nabla^2 \rho$	A-B distance Å	Formal Bond?	Sum of vdW radii[45] Å	Computed Mayer Bond Order
75	3O	11H	0.011054	0.037975	2.54976	N	2.62	0.00095268
92	26C	42H	0.010467	0.03549	2.54493	N	2.80	0.12389507
97	50F	30H	0.011725	0.045722	2.46369	N	2.57	-
107	2Cl	30H	0.007123	0.018375	2.93399	N	2.85	0.19456746
109	2Cl	27H	0.011794	0.032378	2.63149	N	2.85	0.29627845
110	2Cl	46H	0.011308	0.031978	2.66754	N	2.85	0.30070568
51	23C	25H	0.272984	-0.89847	1.10187	Y	2.80	1.01110594
55	23C	40C	0.244681	-0.54021	1.53367	Y	3.40	0.85538441
54	23C	24H	0.270849	-0.88895	1.10187	Y	280	0.97898634
90	1Ga	50F	0.095757	0.499209	1.87254	Y	3.34	0.45455370
71	5N	1Ga	0.072028	0.158798	2.14970	Y	3.42	0.22665576
78	15N	1Ga	0.072560	0.158285	2.14493	Y	3.42	0.06932589
88	6N	1Ga	0.076566	0.177311	2.11710	Y	3.42	0.14607041
73	3O	1Ga	0.083498	0.314436	2.03665	Y	3.39	0.31534936
82	8O	1Ga	0.075205	0.260255	1.98732	Y	3.39	0.26020000

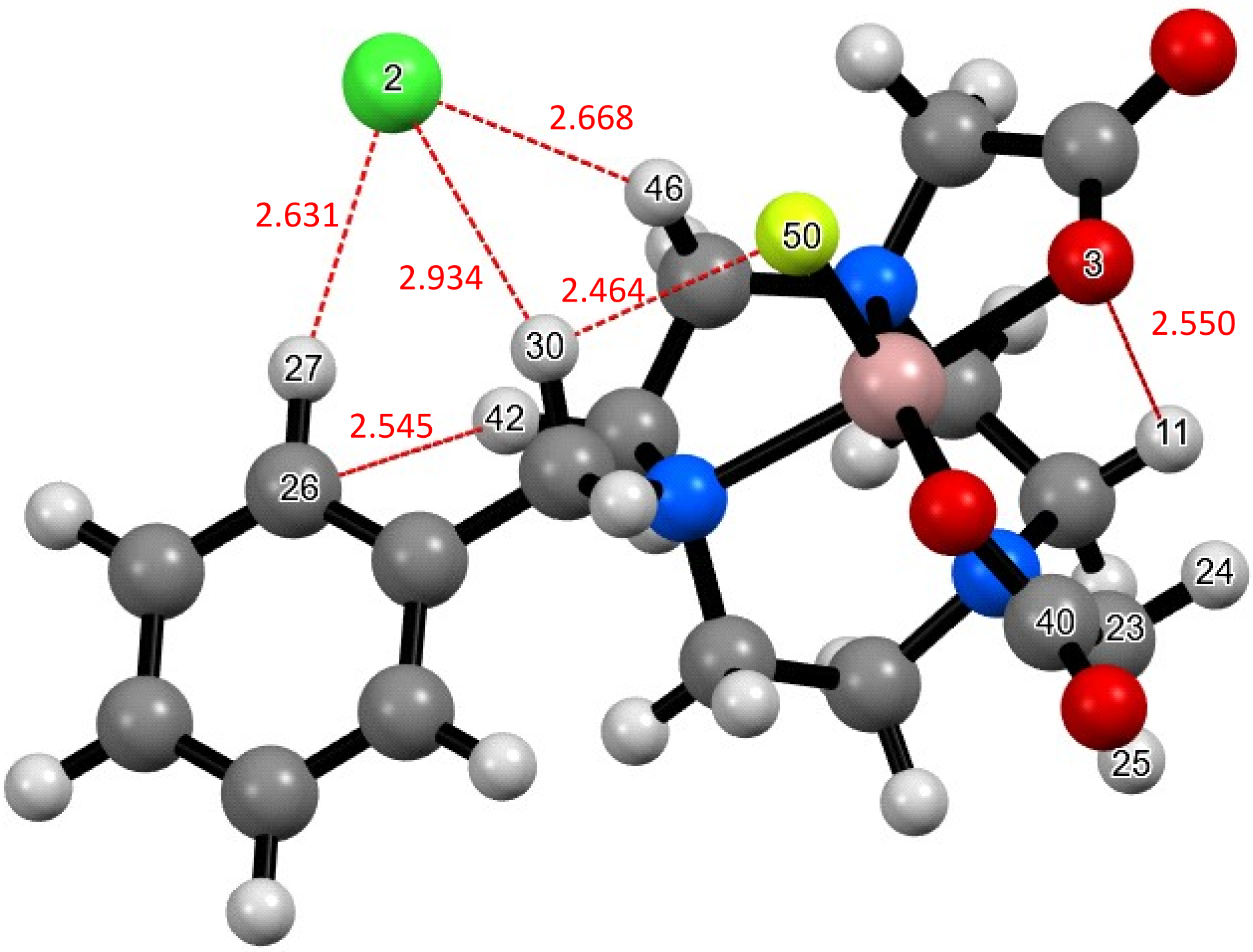

9o) Solution-phase Calculations – Aluminium Reaction Complex (**RC**)

Table for Hydrogen Bond Strengths using RC /BP86-D3 6-311++G(d,p) SMD=water MULTIWFN outputs—using calculated PEDs (Potential Energy Densities, V(r))

MultiWfn identifies 111 CPS.

System has 50 Atoms, 54 "formal bonds" and 111 CPs

i.e. expect 7 "non-bonded interactions" 2 Cl---H, 1 O---H, 1 C---H, 2 H---F, 1 F--O

CP No.	V(r)/au	V(r) /kcal mol^{-1}	Gaussian Atom numbering	Bond Type	
59	-0.005541	-3.477005	25C-41H	C—H	Non-bonded interactions
66	-0.005368	-3.368157	45H-1Cl	H—Cl	
75	-0.008266	-5.186943	29H-1Cl	H—Cl	
95	-0.008779	-5.508758	10H – 2O	H—O	
104	-0.013139	-8.244585	13H – 49F	H—F	
105	-0.012278	-7.704302	17H – 49F	H—F	
111	-0.001199	-0.752380	49F – 7O	F—O	
106	-0.294311	-184.680215	22C—23H	C—H	known bonds
108	-0.253823	-159.273717	22C—39C	C—C	
109	-0.295715	-185.561355	22C—24H	C—H	
78	-0.074505	-46.751880	5N-50Al	Al—N	Bonds around Ga
83	-0.066079	-41.464296	14N-50Al	Al—N	
84	-0.065575	-41.148372	1Cl-50Al	Al—Cl	
93	-0.091379	-57.340059	2O-50Al	Al—O	
96	-0.069966	-43.903916	4N-50Al	Al—N	
100	-0.106643	-66.918461	3O-50Al	Al—O	

Note: No Ga-F bond located

CP	Atom A	Atom B	Bond Density ρ	$\nabla^2 \rho$	A-B distance Å	Formal Bond?	Sum of vdW radii[45] Å	Computed Mayer Bond Order
59	25C	41H	0.010589	0.036478	2.54091	N	2.90	0.16662793
66	1Cl	45H	0.010007	0.032049	2.82554	N	2.95	0.06783253
75	1Cl	29H	0.014244	0.048411	2.60719	N	2.95	0.18114608
95	2O	10H	0.012697	0.045625	2.49412	N	2.72	0.00087769
104	49F	13H	0.018988	0.058293	2.10581	N	2.67	-
105	49F	17H	0.017864	0.055050	2.14434	N	2.67	0.03808812
111	7O	49F	0.002634	0.009242	3.66723	N	2.99	-
106	22C	23H	0.270945	-0.889108	1.10165	Y	2.90	0.96915483
108	22C	39C	0.246157	-0.547618	1.53136	Y	3.40	0.78732452
109	22C	24H	0.272861	-0.898285	1.10004	Y	2.90	1.00313671
78	5N	50Al	0.055070	0.243951	2.07442	Y	3.39	0.81131891
83	14N	50Al	0.051454	0.209189	2.11149	Y	3.39	1.08394235
84	1Cl	50Al	0.051395	0.173482	2.27526	Y	3.59	-
93	2O	50Al	0.060661	0.359283	1.93021	Y	3.36	1.13754588
96	4N	50Al	0.052647	0.226211	2.09592	Y	3.39	0.41472436
100	3O	50Al	0.067304	0.428616	1.88356	Y	3.36	0.87728439

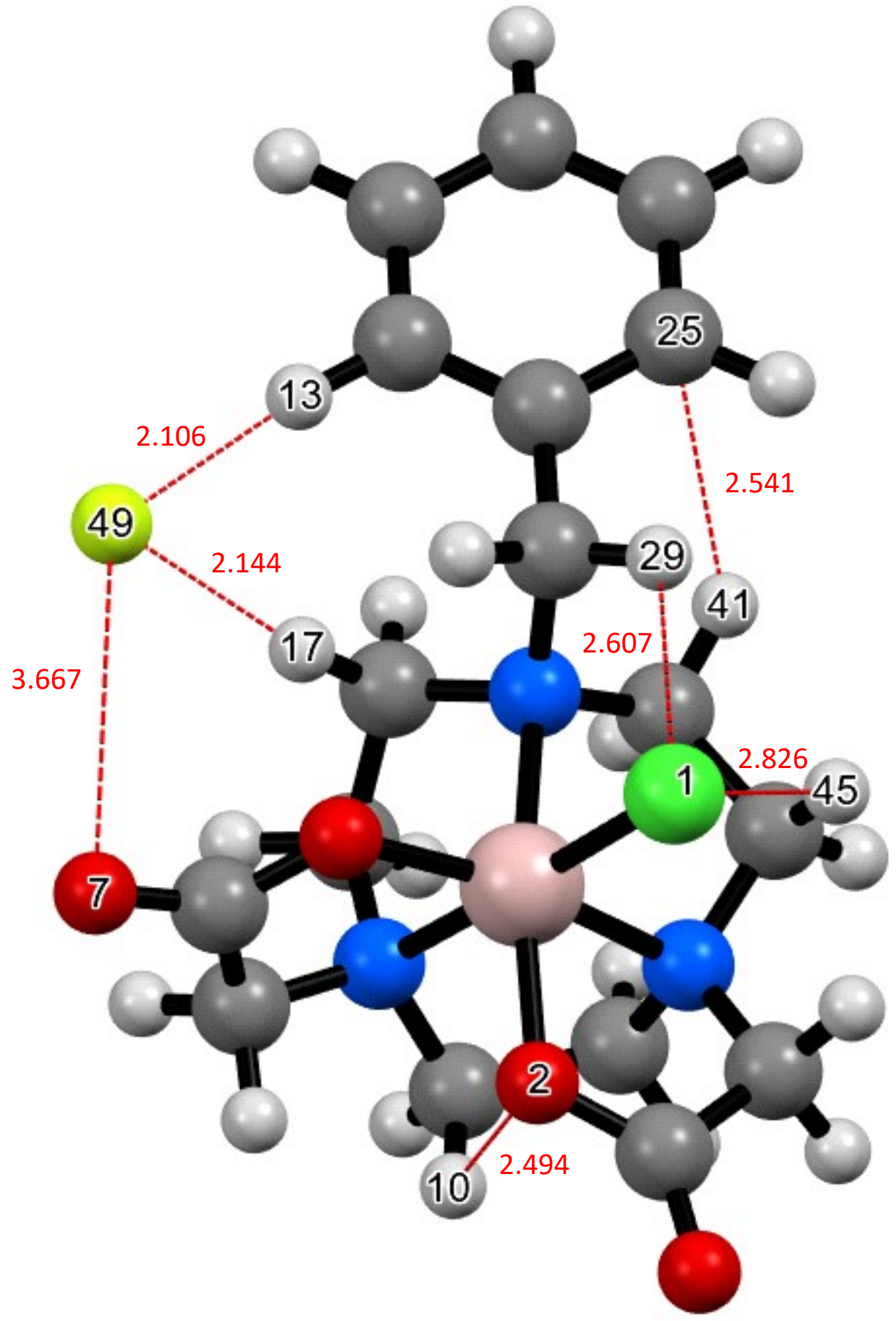

9p) Solution-phase Calculations – Aluminium Transition State 1 (**TS1**)

Table for Hydrogen Bond Strengths using TS1 /BP86-D3 6-311++G(d,p) SMD=water MULTIWFN outputs—using calculated PEDs (Potential Energy Densities, V(r))

MultiWfn identifies 110 CPS. System has 50 Atoms, 54 "formal bonds" and 110 CPs
i.e. expect 6 "non-bonded interactions" 1 Cl---H, 1 O---H, 2 H---F, 1 F—O, 1 H---H

CP No.	V(r)/au	V(r) /kcal mol^{-1}	Gaussian Atom numbering	Bond Type	
55	-0.020716	-12.999389	49F-17H	H—F	Non-bonded interactions
58	-0.017180	-10.780242	49F-3O	F—O	
60	-0.021379	-13.415305	49F-28H	H—F	
85	-0.006567	-4.121007	1Cl-29H	H—Cl	
88	-0.009882	-6.200996	10H-2O	H—O	
90	-0.009027	-5.664515	21H-42H	H—H	
52	-0.297520	- 186.693979	22C—24H	C—H	known bonds
56	-0.257330	- 161.474299	22C—39C	C—C	
59	-0.295508	- 185.431575	22C—23H	C—H	
70	-0.089183	-55.962304	3O-50Al	Al—O	Bonds arou
75	-0.069888	-43.854893	4N-50Al	Al—N	
78	-0.040671	-25.520783	14N-50Al	Al—N	

80	-0.072787	-45.673769	1Cl-50Al	Al—Cl	nd Ga
84	-0.050170	-31.481474	2O-50Al	Al—O	
91	-0.054544	-34.226178	5N-50Al	Al—N	

Note: No Ga-F bond located

CP	Atom A	Atom B	Bond Density ρ	$\nabla^2 \rho$	A-B distance Å	Formal Bond?	Sum of vdW radii[45] Å	Computed Mayer Bond Order
55	49F	17H	0.025997	0.092309	1.99004	N	2.67	0.03504335
58	49F	3O	0.020139	0.076705	2.56573	N	2.99	-0.06914962
60	49F	28H	0.026752	0.094172	1.96650	N	2.67	0.05515049
85	1Cl	29H	0.011755	0.040126	2.71768	N	2.95	0.15309364
88	10H	2O	0.014902	0.051137	2.36783	N	2.72	0.02037690
90	21H	42H	0.014758	0.055021	1.99236	N	2.40	0.01088118
52	22C	24H	0.272369	-0.895949	1.09987	Y	2.90	0.93467708
56	22C	39C	0.248508	-0.559215	1.52618	Y	3.40	0.78238439
59	22C	23H	0.272002	-0.891857	1.10034	Y	2.90	1.01628017
70	3O	50Al	0.060303	0.351516	1.93145	Y	3.36	0.77895185
75	4N	50Al	0.053699	0.220775	2.09188	Y	3.39	0.82919141
78	14N	50Al	0.038171	0.107152	2.28267	Y	3.39	0.54495865
80	1Cl	50Al	0.055704	0.190980	2.23778	Y	3.59	-1.30897761
84	2O	50Al	0.040364	0.171689	2.11926	Y	3.36	0.73746672
91	5N	50Al	0.045280	0.166630	2.17684	Y	3.39	0.78593247

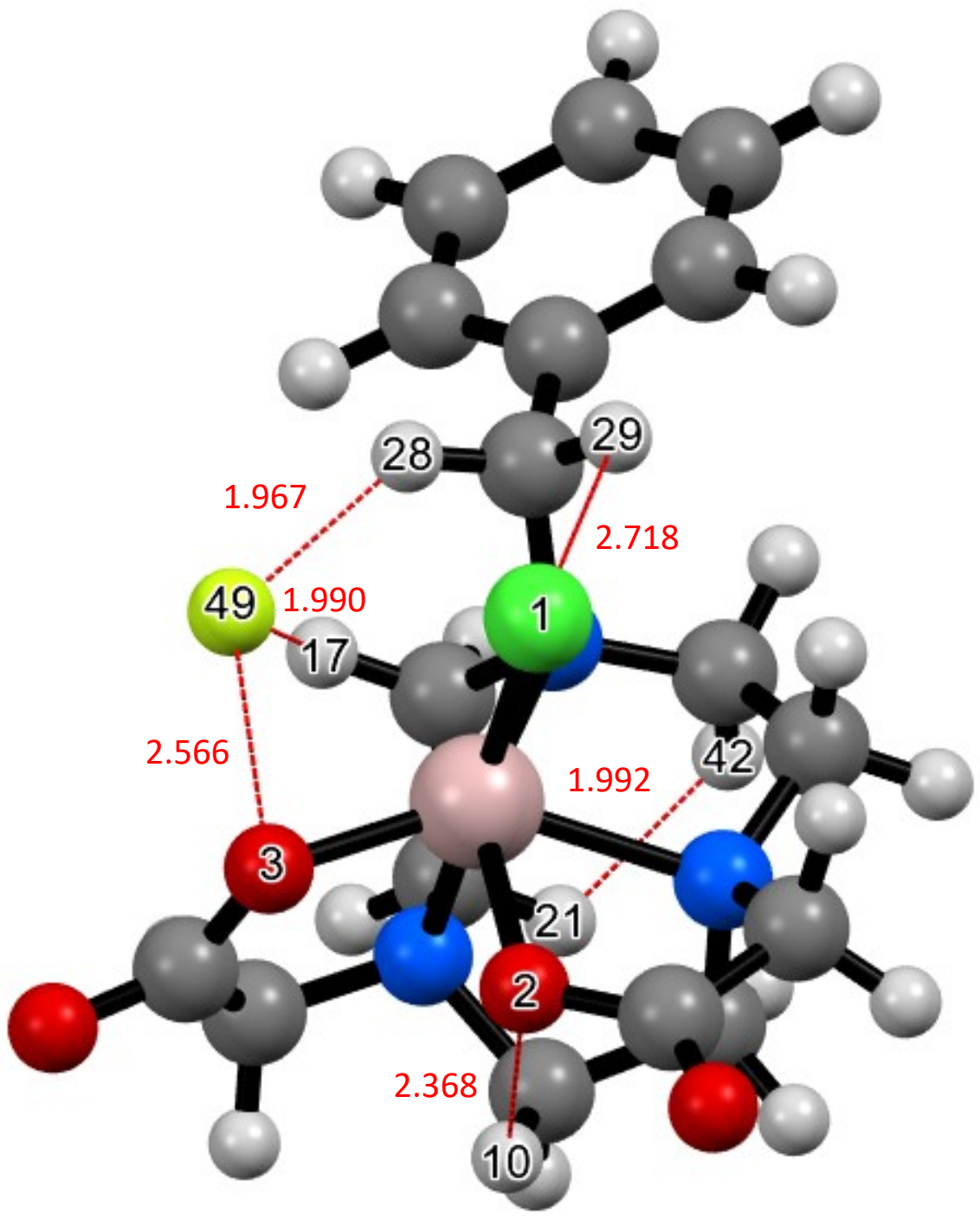

9q) Solution-phase Calculations – Aluminium Ionic Complex 1 (**IC1**)

Table for Hydrogen Bond Strengths using IC1 /BP86-D3 6-311++G(d,p) SMD=water MULTIWFN outputs—using calculated PEDs (Potential Energy Densities, V(r))

MultiWfn identifies 111 CPS.

System has 50 Atoms, 55 "formal bonds" and 111 CPs

i.e. expect 6 "non-bonded interactions" 2 Cl---H, 1 O---H, 2 H---F, 1 H--H

CP No.	V(r)/au	V(r) /kcal mol^{-1}	Gaussian Atom numbering	Bond Type	
62	-0.005986	-3.756231	48H-1Cl	H-Cl	Non-bonded interactions
73	-0.003949	-2.477700	29H-1Cl	H-Cl	
77	-0.008447	-5.300459	42H-21H	H-H	
83	-0.008992	-5.642722	2O-10H	O-H	
98	-0.013397	-8.406640	28H-49F	H-F	
104	-0.012178	-7.641619	49F-17H	H-F	
108	-0.294933	-185.070612	22C—23H	C—H	known bonds
109	-0.256339	-160.853027	22C—39C	C—C	
110	-0.296881	-186.293020	22C—24H	C—H	
78	-0.037740	-23.682086	5N-50Al	Al—N	Bonds around Ga
81	-0.059093	-37.080860	1Cl-50Al	Al—Cl	
84	-0.049388	-30.991137	2O-50Al	Al—O	
85	-0.019933	-12.507864	14N-50Al	Al—N	
93	-0.064710	-40.605701	4N-50Al	Al—N	
96	-0.115570	-72.520277	49F-50Al	Al-F	
100	-0.058193	-36.515945	3O-50Al	Al-O	

(No second Ga-O bond/interaction)

CP	Atom A	Atom B	Bond Density ρ	$\nabla^2 \rho$	A-B distance Å	Formal Bond?	Sum of vdW radii[45] Å	Computed Mayer Bond Order
62	48H	1Cl	0.010848	0.035691	2.78899	N	2.95	0.07623631
73	29H	1Cl	0.007962	0.025311	2.91722	N	2.95	0.12820111
77	42H	21H	0.013859	0.052033	2.02632	N	2.40	0.01431129
83	2O	10H	0.013699	0.046330	2.41516	N	2.72	0.01390788
98	28H	49F	0.017688	0.064637	2.18697	N	2.67	0.03191556
104	49F	17H	0.015827	0.059814	2.28562	N	2.67	-
108	22C	23H	0.271581	-0.887534	1.10146	Y	2.90	1.03717976
109	22C	39C	0.248048	-0.557976	1.52692	Y	3.40	0.78945897
110	22C	24H	0.271654	-0.890729	1.10094	Y	2.90	0.94349589
78	5N	50Al	0.035791	0.100694	2.30765	Y	3.39	0.70148312
81	1Cl	50Al	0.048287	0.152214	2.30597	Y	3.59	-
84	2O	50Al	0.040230	0.170311	2.11765	Y	3.36	0.63298435
85	14N	50Al	0.023427	0.040838	2.55856	Y	3.39	0.48111247
93	4N	50Al	0.050834	0.207787	2.11013	Y	3.39	0.71324585
96	49F	50Al	0.069076	0.498616	1.81535	Y	3.31	1.04763041
100	3O	50Al	0.044678	0.213316	2.06393	Y	3.36	0.59297560

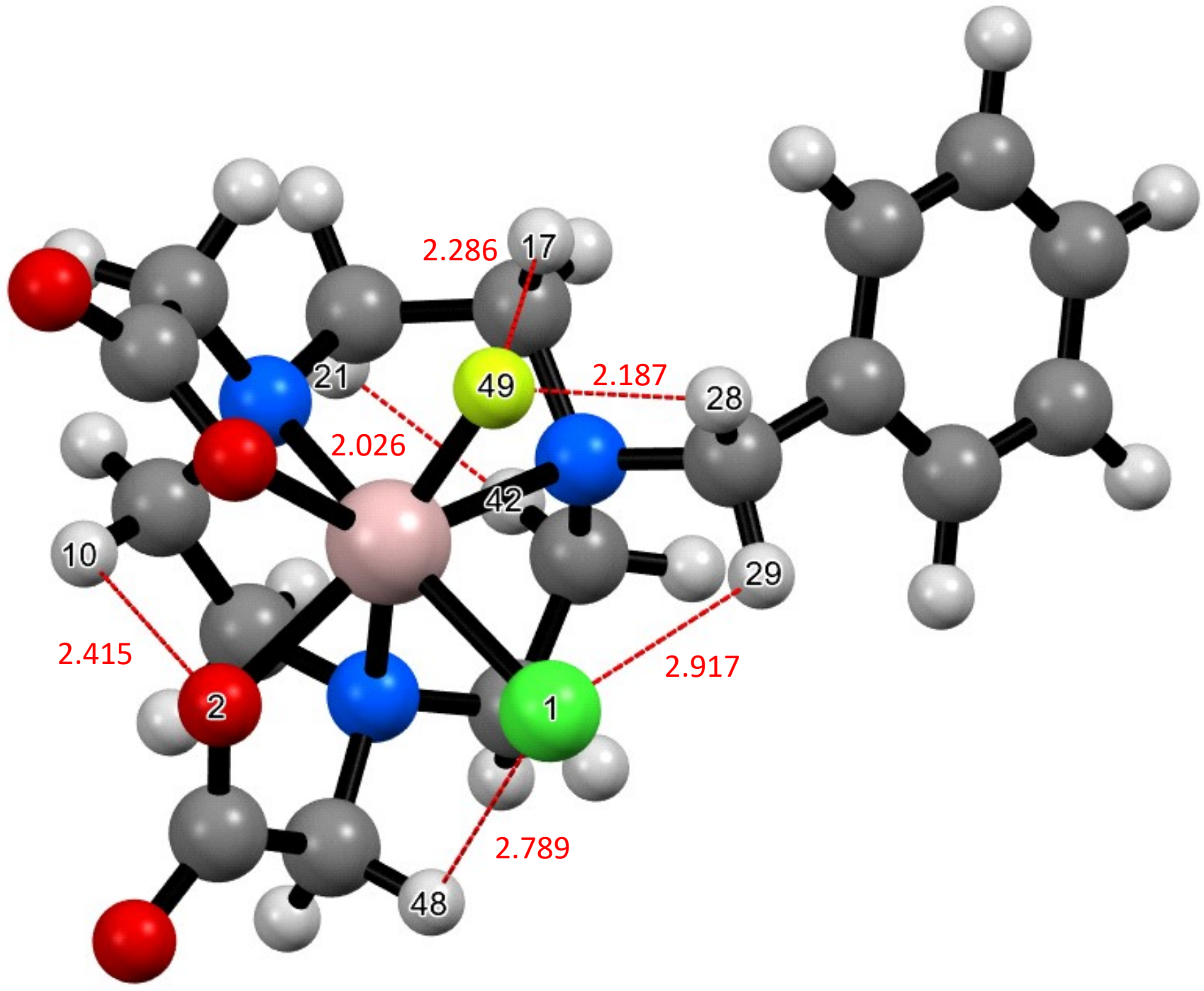

9r) Solution-phase Calculations – Aluminium Ionic Complex transition state (**ICts**)

Table for Hydrogen Bond Strengths using ICts /BP86-D3 6-311++G(d,p) SMD=water MULTIWFN outputs—using calculated PEDs (Potential Energy Densities, V(r))

MultiWfn identifies 114 CPS.

System has 50 Atoms, 55 "formal bonds" and 114 CPs

i.e. expect 9 "non-bonded interactions" 3 Cl---H, 1 O---H, 2 H---F, 1 O—O, 1 H---H, 1 C---H

CP No.	V(r)/au	V(r) /kcal mol^{-1}	Gaussian Atom numbering	Bond Type	
59	-0.005939	-3.726753	48H-1Cl	H-Cl	Non-bonded interactions
62	-0.004557	-2.859323	45H-1Cl	H-Cl	
64	-0.005594	-3.510434	41H-25C	H-C	
75	-0.005251	-3.295291	29H-1Cl	H-Cl	
79	-0.008841	-5.547834	42H-21H	H-H	
86	-0.007877	-4.942512	2O-10H	H-O	
98	-0.022717	-14.254606	2O-3O	O-O	
100	-0.012333	-7.739163	28H-49F	H-F	
107	-0.010918	-6.850811	49F-17H	H-F	
111	-0.256536	-160.976509	22C—39C	C—C	known bonds
112	-0.294977	-185.098033	22C—23H	C—H	
113	-0.296814	-186.251023	22C—24H	C—H	
80	-0.041375	-25.962814	5N-50Al	Al—N	B

83	-0.053913	-33.830250	1Cl-50Al	Al—Cl	onds around Ga
84	-0.062838	-39.430931	2O-50Al	Al—O	
85	-0.025176	-15.798144	14N-50Al	Al—N	
95	-0.062433	-39.176972	4N-50Al	Al—N	
99	-0.122232	-76.700566	49F-50Al	Al-F	
102	-0.034696	-21.771808	3O-50Al	Al-O	

CP	Atom A	Atom B	Bond Density ρ	$\nabla^2 \rho$	A-B distance Å	Formal Bond?	Sum of vdW radii[45] Å	Computed Mayer Bond Order
59	48H	1Cl	0.010645	0.035235	2.81506	N	2.95	0.05728448
62	45H	1Cl	0.008668	0.026990	2.88827	N	2.95	0.09651122
64	41H	25C	0.010683	0.036247	2.54282	N	2.90	0.18306864
75	29H	1Cl	0.010052	0.032605	2.79201	N	2.95	0.13804807
79	42H	21H	0.014740	0.054085	1.98918	N	2.40	0.01427945
86	2O	10H	0.012084	0.040559	2.49121	N	2.72	0.00750249
98	2O	3O	0.027214	0.111206	2.41328	N	3.04	-
100	28H	49F	0.016499	0.059832	2.22100	N	2.67	0.03213419
107	49F	17H	0.014360	0.054229	2.16286	N	2.67	-
111	22C	39C	0.248224	-0.559288	1.52634	Y	3.40	0.77464984
112	22C	23H	0.271453	-0.885725	1.10182	Y	2.90	1.03396369
113	22C	24H	0.271416	-0.888502	1.10139	Y	2.90	0.95757075
80	5N	50Al	0.037822	0.113866	2.27703	Y	3.39	0.73906006
83	1Cl	50Al	0.045339	0.137192	2.33779	Y	3.59	-
84	2O	50Al	0.047621	0.233146	2.03571	Y	3.36	0.77673980
85	14N	50Al	0.027069	0.057609	2.46494	Y	3.39	0.56052886
95	4N	50Al	0.049777	0.199252	2.12148	Y	3.39	0.74876812
99	49F	50Al	0.071793	0.532603	1.79928	Y	3.31	1.12523204
102	3O	50Al	0.031104	0.105471	2.24964	Y	3.36	0.60254517

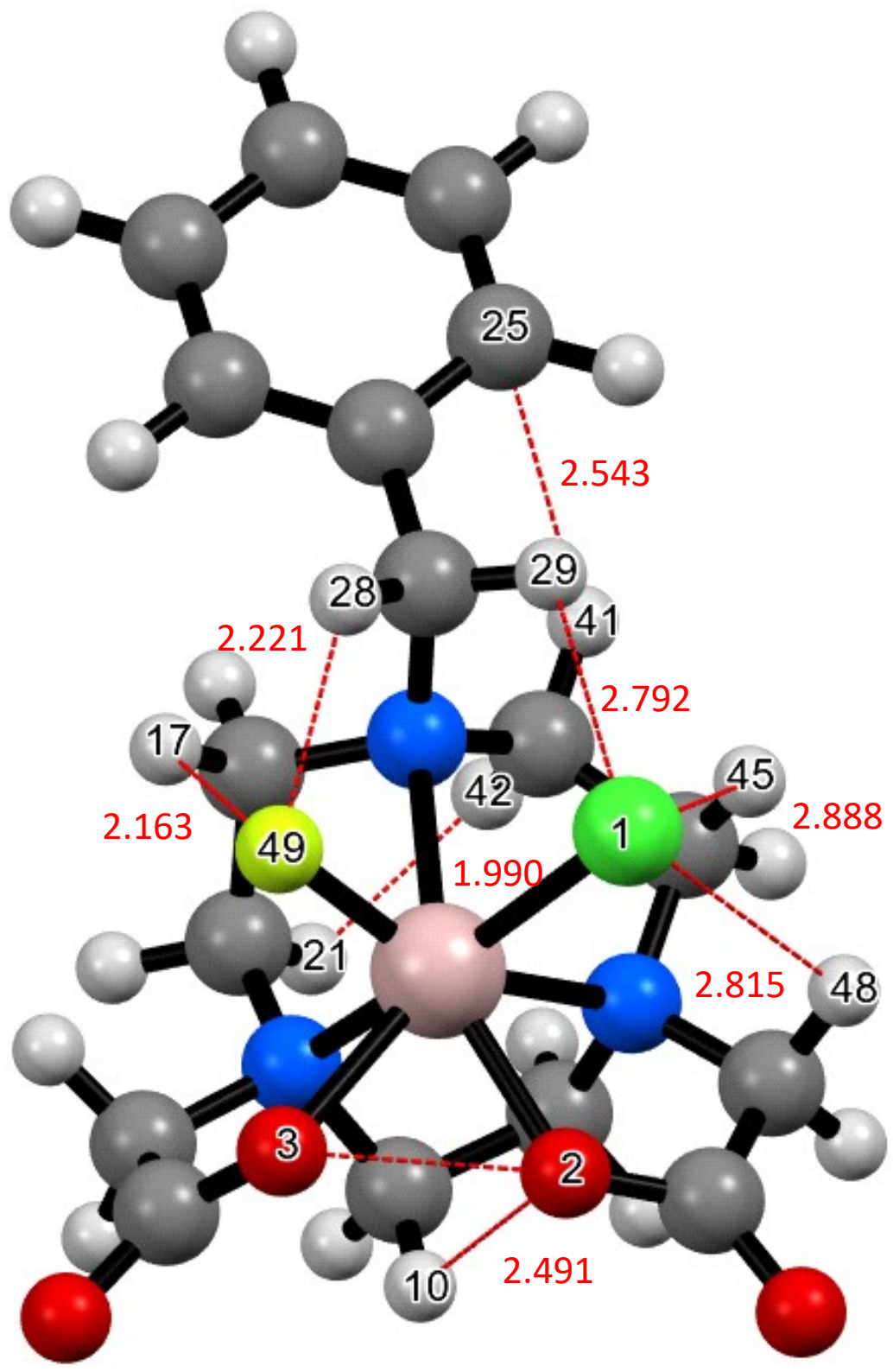
25
2.543
28
29
41
2.221
2.792
17
42
45
2.163
49
1
2.888
1.990
21
2.815
48
3
2
10
2.491

9s) Solution-phase Calculations – Aluminium Ionic Complex 2 (**IC2**)

Table for Hydrogen Bond Strengths using IC2 /BP86-D3 6-311++G(d,p) SMD=water MULTIWFN outputs—using calculated PEDs (Potential Energy Densities, V(r))

MultiWfn identifies 112 CPS.

System has 50 Atoms, 54 “formal bonds” and 112 CPs

i.e. expect 8 “non-bonded interactions” 3 Cl---H, 3 O---H, 1 H---C, 1 F—C.

CP No.	V(r)/au	V(r) /kcal mol^{-1}	Gaussian Atom numbering	Bond Type	
58	-0.006876	-4.314506	45H-1Cl	H-Cl	Non-bonded interactions
61	-0.005439	-3.413048	41H-25C	H-C	
63	-0.007022	-4.406142	48H-1Cl	H-Cl	
73	-0.008282	-5.196665	29H-1Cl	H-Cl	
88	-0.008318	-5.219347	2O-10H	H-O	
106	-0.007760	-4.869515	17H-7O	H-O	
107	-0.008085	-5.073533	49F-39C	F-C	
108	-0.009402	-5.899850	20H-7O	H-O	
105	-0.303639	-190.533664	22C—23H	C—H	known bonds
109	-0.290921	-182.552753	22C—24H	C—H	
110	-0.236763	-148.568620	22C—39C	C—C	
77	-0.067886	-42.598664	5N-50Al	Al—N	Bonds around Ga
82	-0.052132	-32.712686	1Cl-50Al	Al—Cl	
84	-0.064892	-40.719691	14N-50Al	Al—N	
86	-0.093276	-58.530755	2O-50Al	Al—O	
92	-0.062834	-39.428138	4N-50Al	Al—N	
97	-0.142906	-89.673706	49F-50Al	Al—F	

CP	Atom A	Atom B	Bond Density ρ	$\nabla^2 \rho$	A-B distance Å	Formal Bond?	Sum of vdW radii[45] Å	Computed Mayer Bond Order
58	45H	1Cl	0.012567	0.039481	2.68181	N	2.95	0.11905877
61	41H	25C	0.010460	0.035460	2.54581	N	2.90	0.16603267
63	48H	1Cl	0.012240	0.040385	2.73914	N	2.95	0.02161901
73	29H	1Cl	0.014549	0.048161	2.58430	N	2.95	0.13252481
88	10H	2O	0.012069	0.043412	2.52424	N	2.72	0.00693894
106	17H	7O	0.012612	0.039812	2.41277	N	2.72	-
107	49F	39C	0.010961	0.042908	2.80950	N	3.17	0.15621777
108	20H	7O	0.014485	0.047155	2.41886	N	2.72	0.01042579
105	22C	23H	0.276685	-0.919031	1.09694	Y	2.90	0.99990022
109	22C	24H	0.267175	-0.857112	1.10762	Y	2.90	0.93479976
110	22C	39C	0.238139	-0.512715	1.54628	Y	3.40	0.84471016
77	5N	50Al	0.051747	0.217776	2.10618	Y	3.39	0.50467296
82	1Cl	50Al	0.043906	0.135718	2.35059	Y	3.59	-
84	14N	50Al	0.050696	0.207851	2.11439	Y	3.39	0.60008263
86	2O	50Al	0.061452	0.372379	1.92070	Y	3.36	0.90845850
92	4N	50Al	0.049251	0.202482	2.12524	Y	3.39	0.15751373
97	49F	50Al	0.079174	0.634224	1.75857	Y	3.31	1.27206722

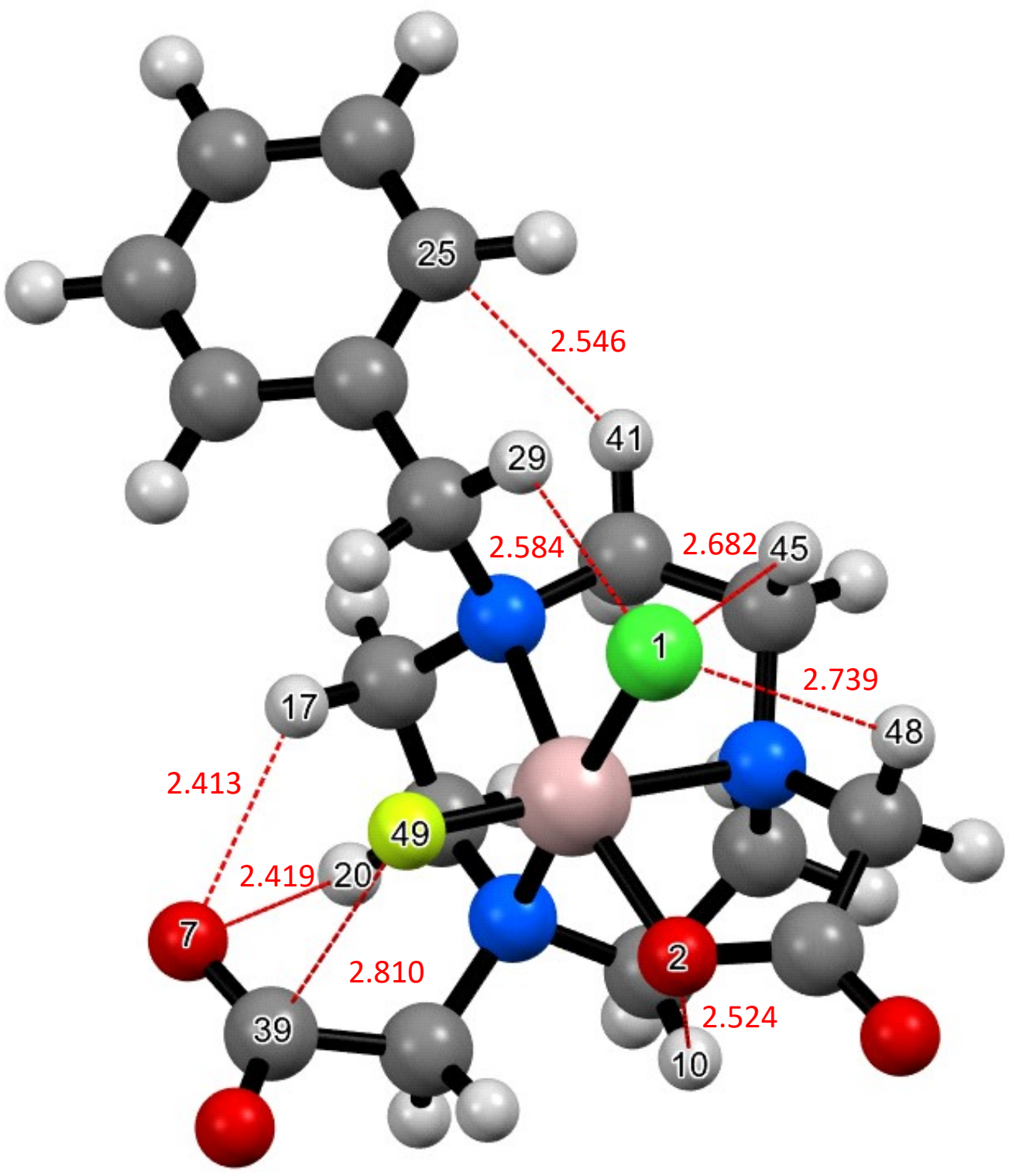

9t) Solution-phase Calculations – Aluminium Transition State 2 (**TS2**)

Table for Hydrogen Bond Strengths using TS2 /BP86-D3 6-311++G(d,p) SMD=water MULTIWFN outputs—using calculated PEDs (Potential Energy Densities, V(r))

MultiWfn identifies 110 CPS.

System has 50 Atoms, 53 “formal bonds” and 110 CPs

i.e. expect 7 “non-bonded interactions” 3 Cl---H, 1 O---H, 1 H---F, 1 F—O, 1 F---Cl.

CP No.	V(r)/au	V(r) /kcal mol^{-1}	Gaussian Atom numbering	Bond Type	
51	-0.007652	-4.801637	48H-1Cl	H—Cl	
53	-0.008937	-5.608240	1Cl-45H	H—Cl	
63	-0.004266	-2.676937	1Cl-29H	H—Cl	
72	-0.002998	-1.881271	1Cl-49F	F—Cl	
89	-0.010824	-6.792268	49F-28H	H—F	
102	-0.009263	-5.812413	49F-7O	F—O	
105	-0.009931	-6.231751	17H-7O	H—O	
106	-0.296163	-185.842453	22C—23H	C—H	known bonds
107	-0.295935	-185.699399	22C—24H	C—H	
108	-0.248890	-156.178677	22C—39C	C—C	
78	-0.077001	-48.317877	5N-50Al	Al—N	Bonds around Ga
82	-0.105310	-66.082284	2O-50Al	Al—O	
83	-0.074245	-46.588824	14N-50Al	Al—N	
86	-0.161273	-101.198572	49F-50Al	Al—F	

90	-0.086142	-54.054158	4N-50Al	Al—N	

CP	Atom A	Atom B	Bond Density ρ	$\nabla^2 \rho$	A-B distance Å	Formal Bond?	Sum of vdW radii[45] Å	Computed Mayer Bond Order
51	48H	1Cl	0.015204	0.043069	2.52465	N	2.95	0.17833197
53	45H	1Cl	0.017005	0.048307	2.46085	N	2.95	0.29451421
63	29H	1Cl	0.009645	0.025654	2.75530	N	2.95	0.21394750
72	49F	1Cl	0.005972	0.019293	3.47074	N	3.22	-
89	28H	49F	0.013928	0.055442	2.36995	N	2.67	0.05386293
102	49F	7O	0.012319	0.041283	2.82922	N	2.99	-
105	17H	7O	0.015710	0.052162	2.28841	N	2.72	0.04195935
106	22C	23H	0.271801	-0.892203	1.10156	Y	2.90	0.99012606
107	22C	24H	0.272150	-0.890854	1.10167	Y	2.90	0.92894010
108	22C	39C	0.244160	-0.539254	1.53424	Y	3.40	0.91390878
78	5N	50Al	0.056888	0.246595	2.06658	Y	3.39	0.12201629
82	2O	50Al	0.066685	0.418895	1.88953	Y	3.36	1.54835044
83	14N	50Al	0.055841	0.234120	2.07676	Y	3.39	0.55483629
86	49F	50Al	0.085621	0.720212	1.72843	Y	3.31	2.16019817
90	4N	50Al	0.061546	0.286459	2.02098	Y	3.39	-

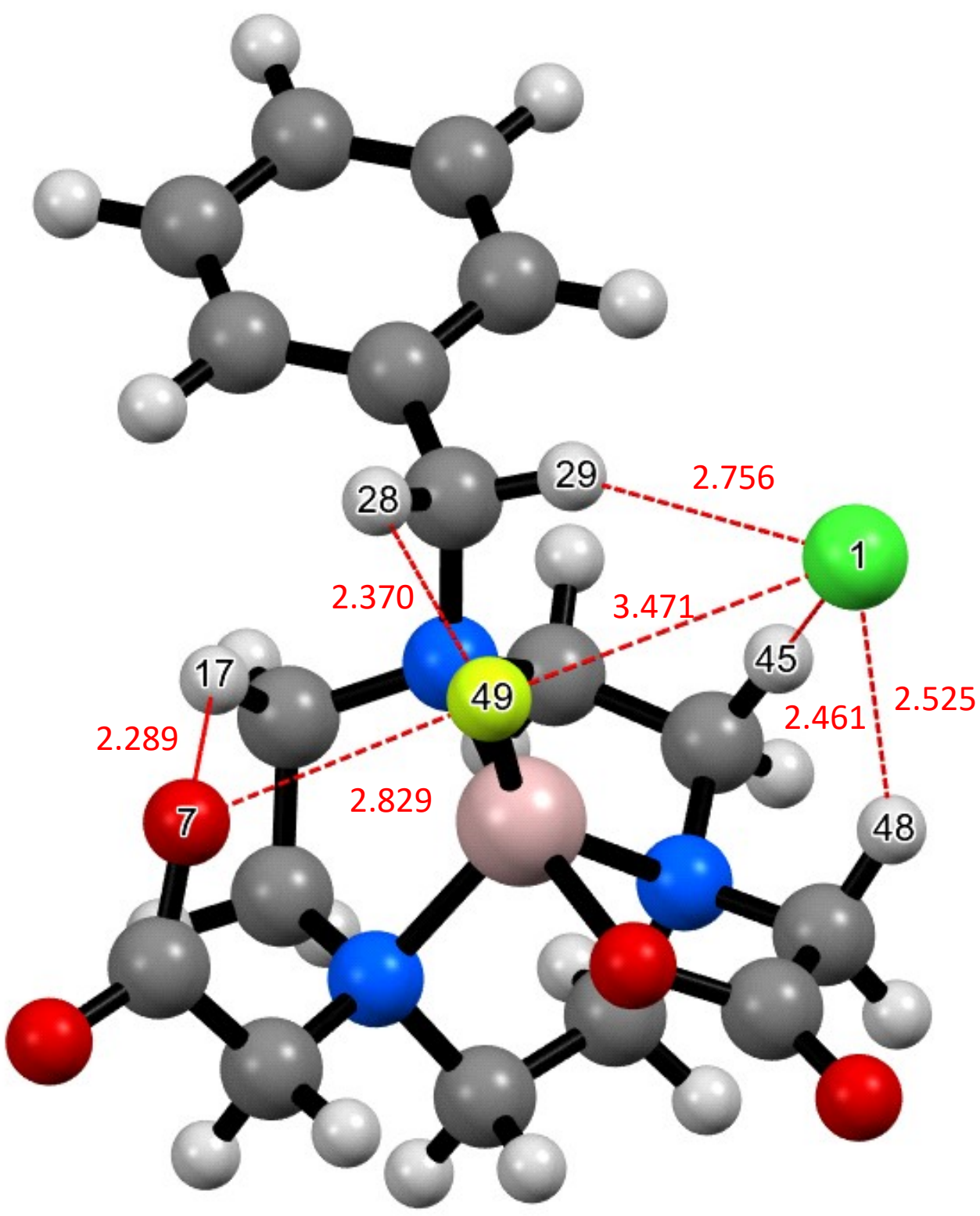

9u) Solution-phase Calculations – Aluminium Product Complex (**PC**)

Table for Hydrogen Bond Strengths using TS2 /BP86-D3 6-311++G(d,p) SMD=water MULTIWFN outputs—using calculated PEDs (Potential Energy Densities, V(r))

MultiWfn identifies 110 CPS.

System has 50 Atoms, 54 "formal bonds" and 110 CPs

i.e. expect 6 "non-bonded interactions" 3 Cl---H, 1 O---H, 1 H---F, 1 C---H.

CP No.	V(r)/au	V(r) /kcal mol^{-1}	Gaussian Atom numbering	Bond Type	
84	-0.010287	-6.454991	29H-49F	H—F	
87	-0.008406	-5.275008	10H-2O	H—O	
89	-0.005722	-3.590394	25C-41H	C—H	
96	-0.002766	-1.735788	29H-1Cl	H—Cl	
100	-0.005374	-3.372023	26H-1Cl	H—Cl	
107	-0.005036	-3.160060	45H-1Cl	H—Cl	
54	-0.295887	-185.669301	22C—24H	C—H	known bonds
57	-0.253895	-159.319262	22C—39C	C—C	
61	-0.294053	-184.518141	22C—23H	C—H	
72	-0.103145	-64.723540	7O-50Al	Al-O	Bonds around Ga
76	-0.068890	-43.228744	4N-50Al	Al—N	
78	-0.067802	-42.545980	14N-50Al	Al—N	
82	-0.090161	-56.575934	2O-50Al	Al—O	
83	-0.140410	-88.107493	49F-50Al	Al—F	
90	-0.076204	-47.817900	5N-50Al	Al—N	

CP	Atom A	Atom B	Bond Density ρ	$\nabla^2 \rho$	A-B distance Å	Formal Bond?	Sum of vdW radii[45] Å	Computed Mayer Bond Order
84	29H	49F	0.013221	0.052676	2.40199	N	2.67	0.02049751
87	10H	2O	0.012174	0.043785	2.51671	N	2.72	0.00590220
89	41H	25C	0.011027	0.037431	2.51675	N	2.90	0.13153987
96	29H	1Cl	0.006433	0.016436	2.99457	N	2.95	0.11879405
100	26H	1Cl	0.011826	0.032419	2.62884	N	2.95	0.27196476
107	45H	1Cl	0.010903	0.030670	2.68716	N	2.95	0.30117929
54	22C	24H	0.272848	-0.897647	1.10177	Y	2.90	0.99346000
57	22C	39C	0.246217	-0.548009	1.53134	Y	3.40	0.83492031
61	22C	23H	0.270813	-0.888393	1.10011	Y	2.90	0.97367534
72	7O	50Al	0.065585	0.415856	1.89208	Y	3.36	0.62750933
76	4N	50Al	0.051940	0.222007	2.10224	Y	3.39	0.31801845
78	14N	50Al	0.051823	0.219966	2.10144	Y	3.39	0.53185263
82	2O	50Al	0.059879	0.356793	1.93305	Y	3.36	0.49647791
83	49F	50Al	0.078160	0.619357	1.76461	Y	3.31	0.50923386
90	5N	50Al	0.055623	0.252050	2.06575	Y	3.39	0.27539635

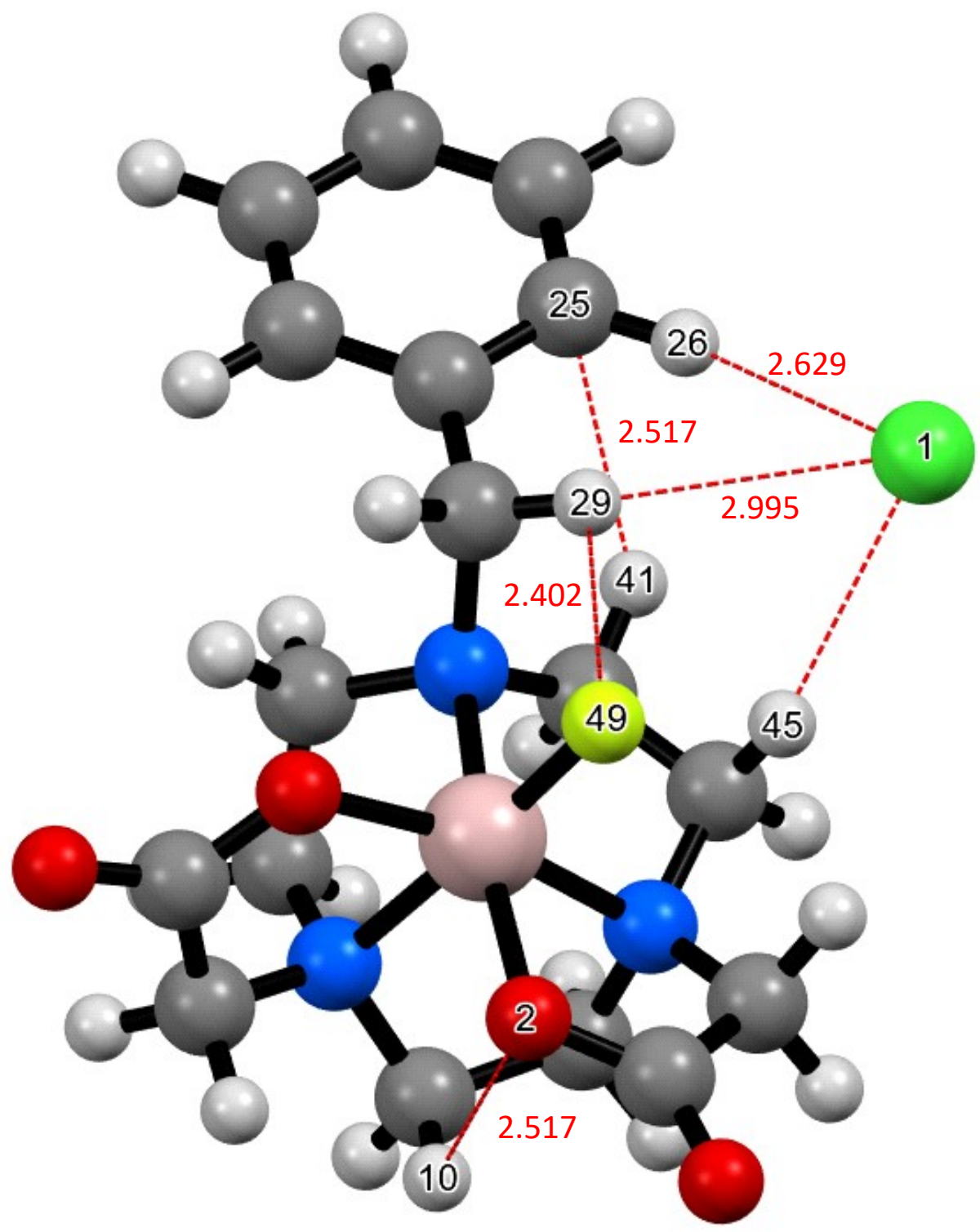

25
26
2.629
2.517
1
29
2.995
2.402
41
49
45
2
2.517
10

9v) Comparison of RC structures from AIM data

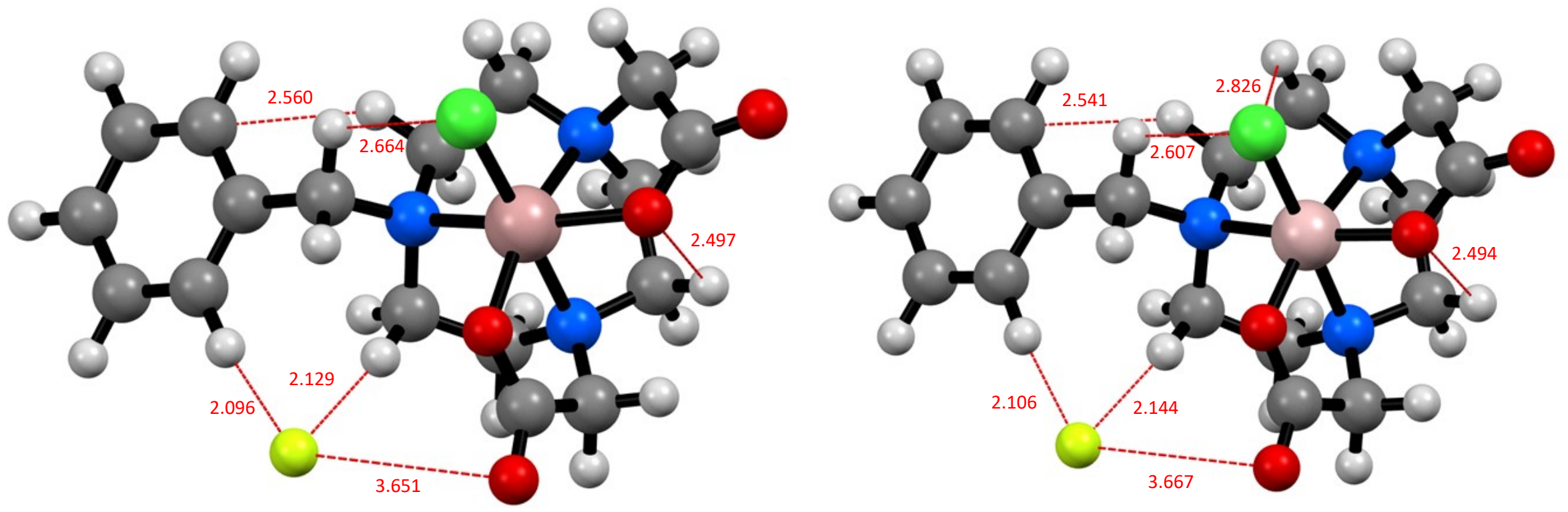

(a) (b)

Computed structures of the **RC** for the reaction F^- + [MCl(Bn-NODA)] at the BP86-D3/6-311++G(d,p) level in solution (H_2O) (a) M = Ga and (b) M = Al, showing located non-bonding interactions. Atom key: **Ga**, **Cl**, **F**, **O**, **N**, **C**, **H**.

9w) Comparison of IC1 structures from AIM data

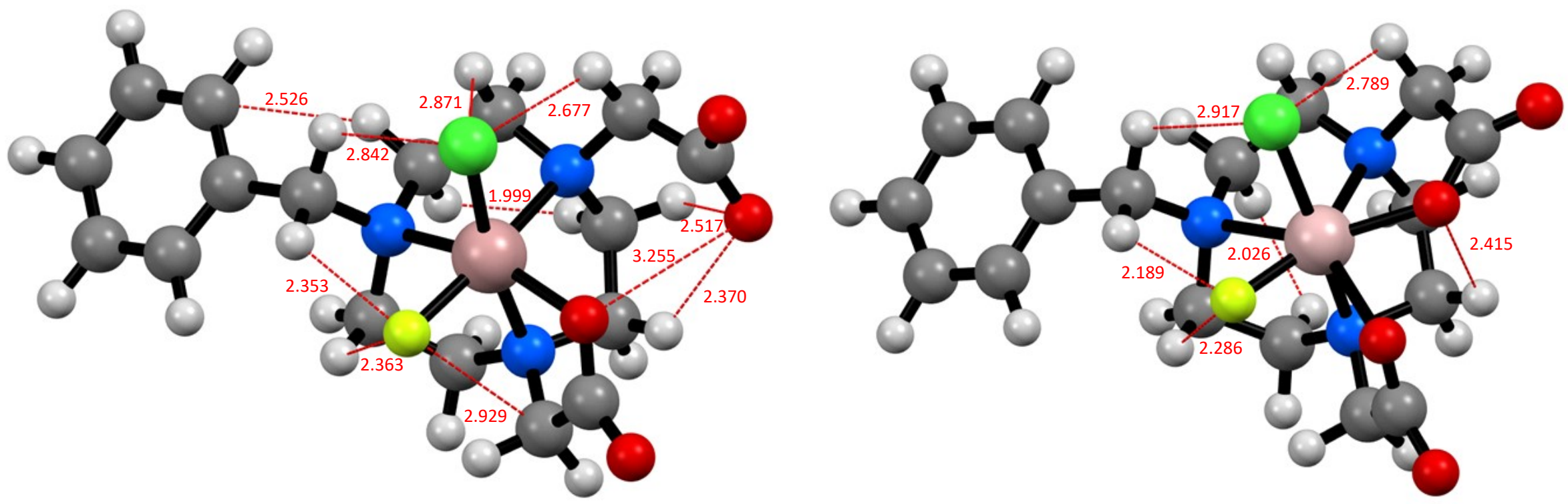

(a) (b)

Computed structures of the **IC1** for the reaction F^- + [MCl(Bn-NODA)] at the BP86-D3/6-311++G(d,p) level in solution (H_2O) (a) M = Ga and (b) M = Al, showing located non-bonding interactions. Atom key: **Ga**, **Cl**, **F**, **O**, **N**, **C**, **H**.

10. Geometric data and energies for Ga(L), Ga(L)$^+$, Al(L), Al(L)$^+$

10a) Gas phase Ga(L)$^+$ & Ga(L)^0 geometries

	**Ga(L)^0**	**Ga(L)$^+$**
Ga-O	1.99476<br>2.01391	1.86840<br>1.88691
Ga-N	2.27115<br>2.36475<br>2.32650	2.16262<br>2.12372<br>2.04504
N-Ga-O	153.26374<br>154.24092	164.68229<br>148.03564
**Energy**	-3052.369891	-3052.196853
**Energy G**	-3052.038407	-3051.861309

**Ga(L)$^+$**	**Ga(L)^0**
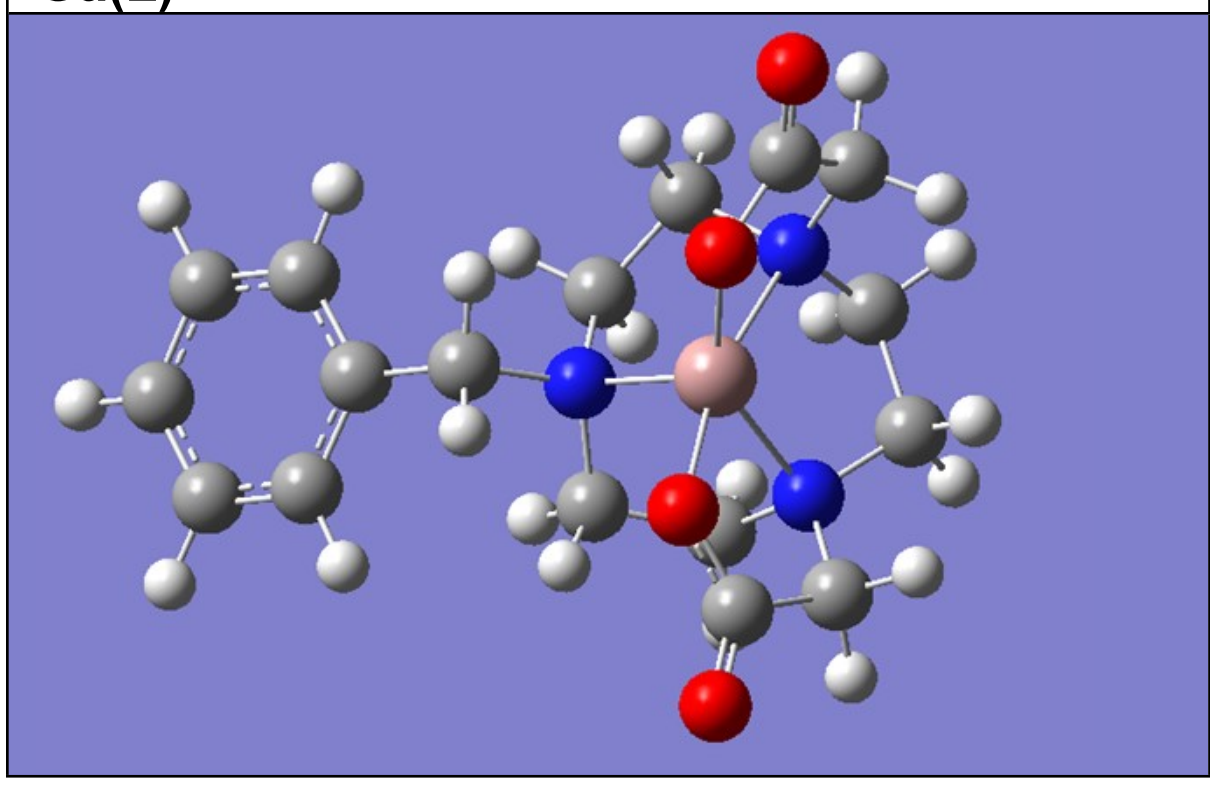	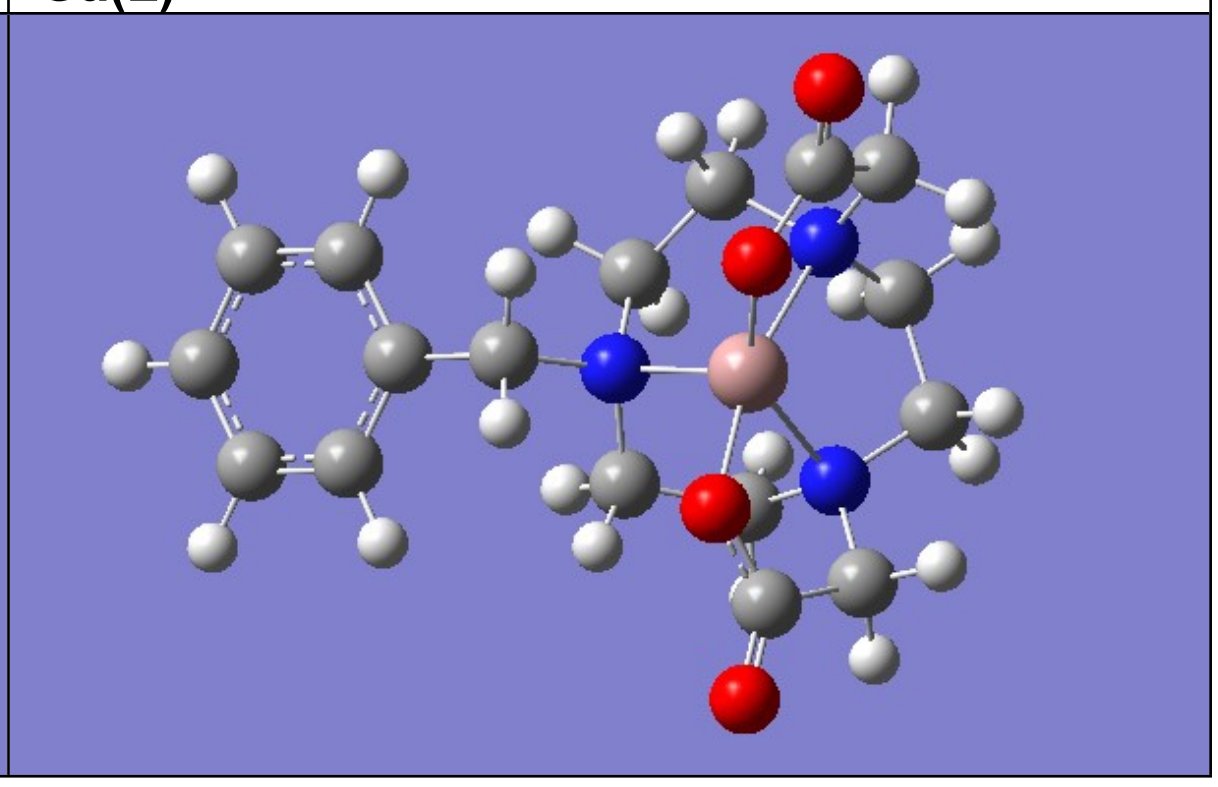

10b) Solution phase Ga(L)$^+$ & Ga(L)^0 geometries

	**Ga(L)^0**	**Ga(L)$^+$**
Ga-O	2.12108<br>2.13737	1.93470<br>1.92238
Ga-N	2.26098<br>2.22166<br>2.19002	2.07833<br>2.03316<br>2.09352
N-Ga-O	157.89222<br>158.20648	162.21169<br>146.50093
**Energy**	-3052.428297	-3052.332896
**Energy G**	-3052.095870	-3051.995985

**Ga(L)$^+$**	**Ga(L)^0**
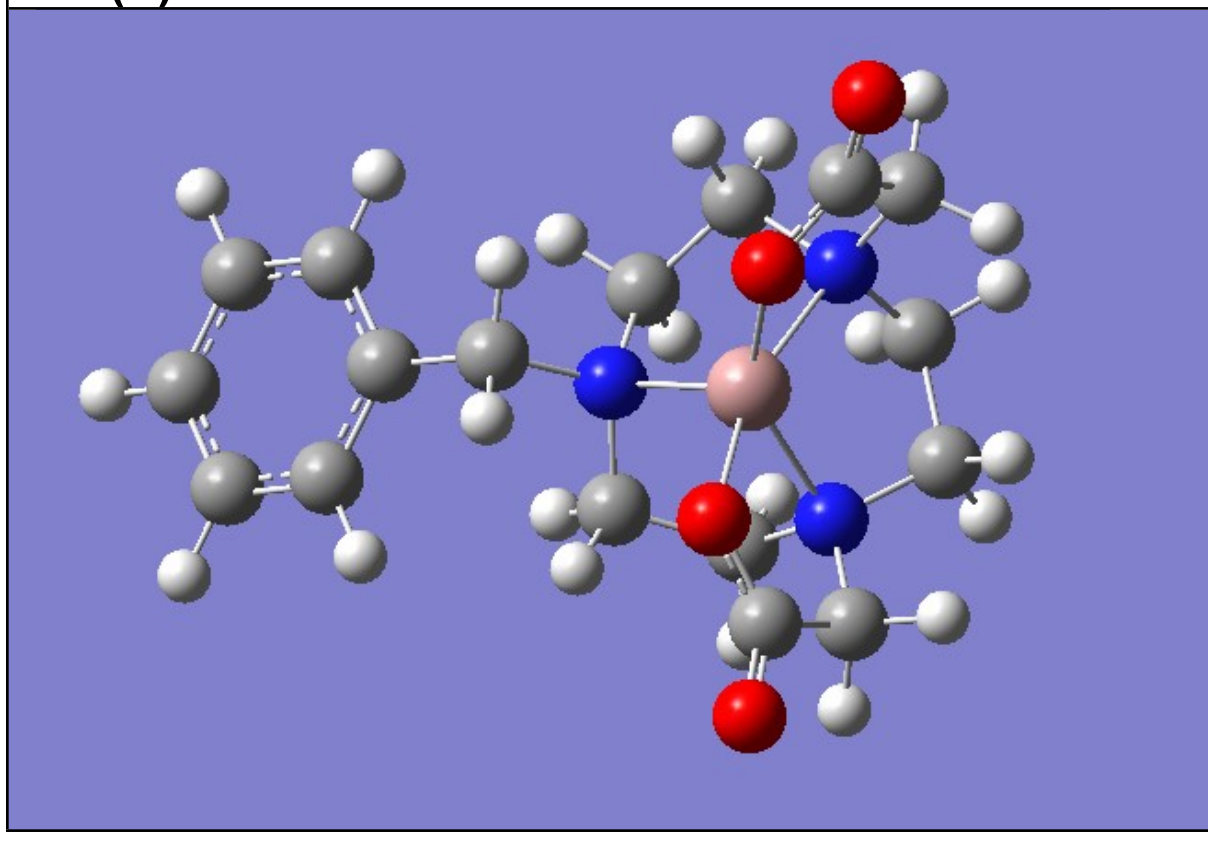	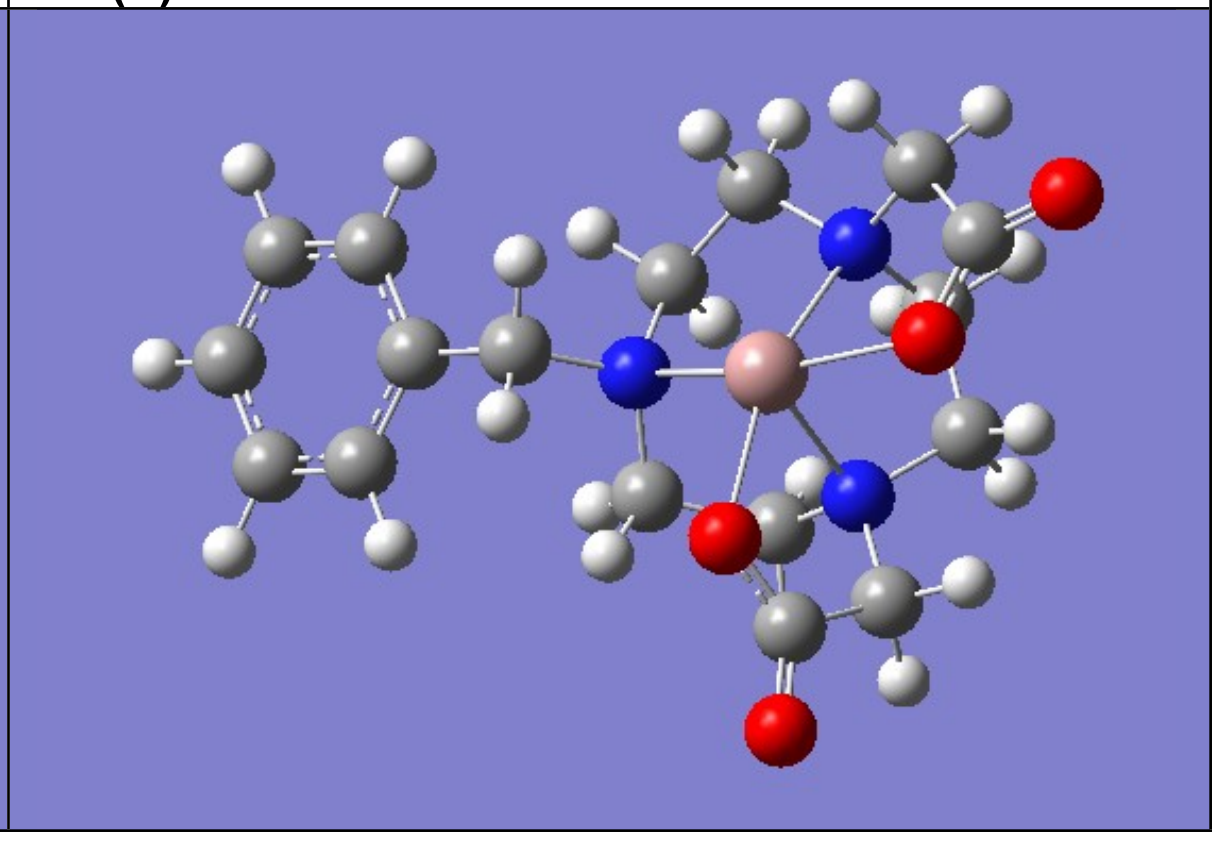

10c) Solution phase Al(L)$^+$ & Al(L)^0 geometries

	**Al(L)$^+$**	**Al(L)^0**
Al-O	1.82836<br>1.84025	1.94295<br>1.92570
Al-N	1.97693<br>2.03912<br>2.01867	2.14352<br>2.11447<br>2.12447
N-Al-O	164.67594<br>146.53873	164.34749<br>162.97245
**Energy**	-1369.773647	-1369.848114
**Energy G**	-1369.435306	-1369.512073

**Al(L)$^+$**	**Al(L)^0**
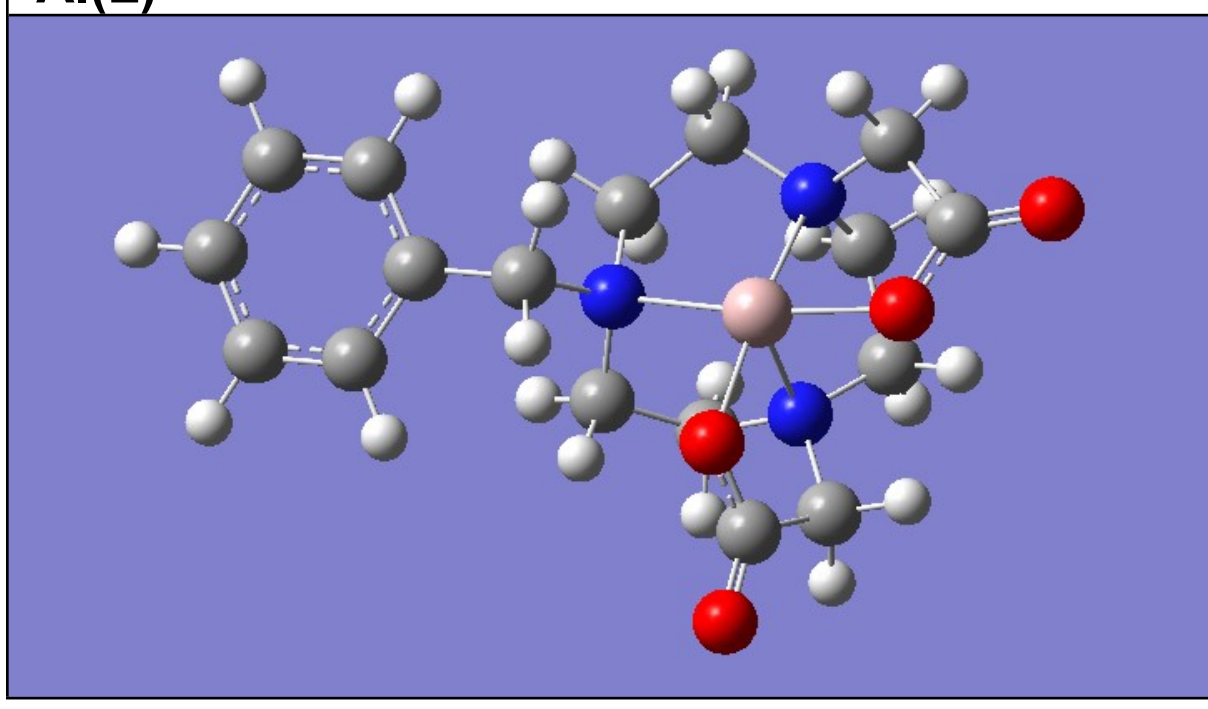	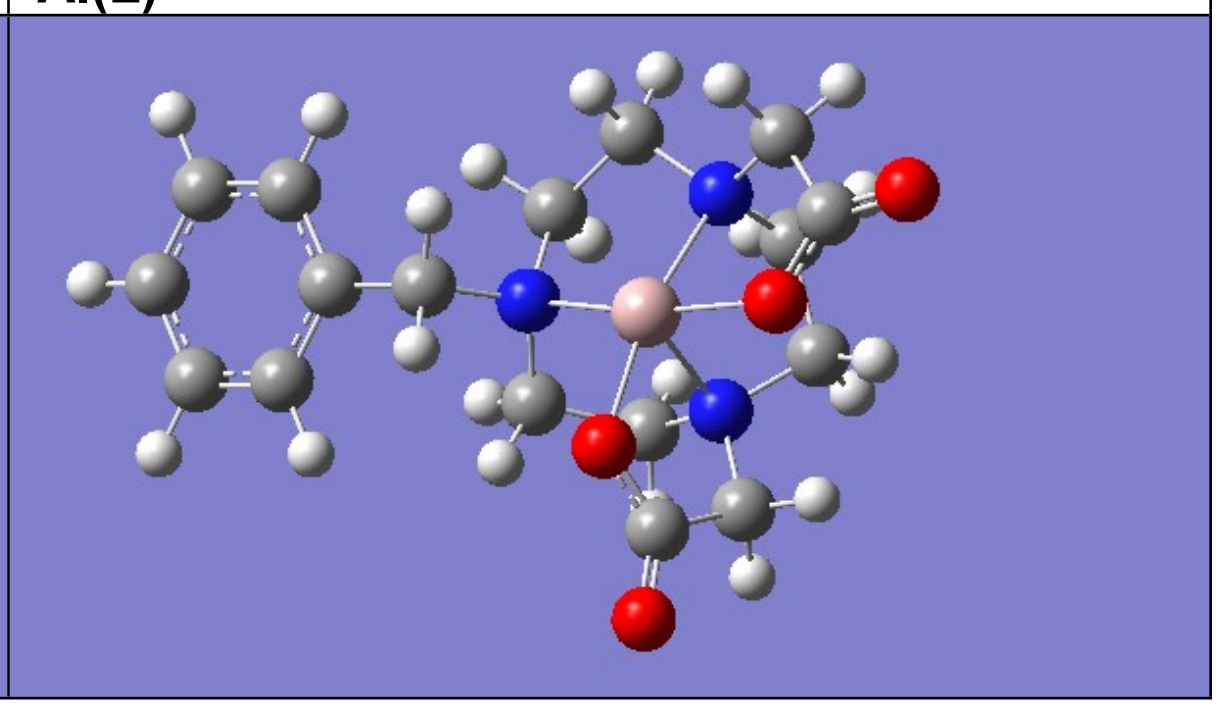

11. Electron Affinity calculations and tables for F, Cl, OH

11a) Benchmarking for Cl / F electron affinity

Optimisation calculations of Cl & F (charge= 0, doublet) and Cl^- & F^- (charge= -1, singlet) with each of the basis sets combinations below to calculate computed electron affinity values.

Table of energies from calculations (au)

	Cl0	Cl-	F0	F-
M06-2X/aug-cc-pvdz	-460.12378	-460.25741	-99.705436	-99.823195
M06-2X /aug-cc-pvtz	-460.14121	-460.27490	-99.732322	-99.853430
M06-2X /aug-cc-pvqz	-460.14322	-460.27604	-99.736680	-99.856997
M06-2X/6-311G(d,p)	-460.13165	-460.26609	-99.720948	-99.788666
BP86 / 6-31G	-460.15458	-460.27554	-99.704968	-99.747983
BP86-D3/ aug-cc-pVTZ	-460.19889	-460.33765	-99.757524	-99.895679
BP86-D3/6-311G(d,p)	-460.18933	-460.32774	-99.745344	-99.818081
BP86-D3/6-311+G(d,p)	-460.19012	-460.33082	-99.752242	-99.888239
BP86-D3/6-311++G(d,p)	-460.19012	-460.33082	-99.752242	-99.888239
BP86-D3/6-311G(2d,p)	-460.18950	-460.32774	-99.745552	-99.818081
BP86-D3/6-311G(df,p)	-460.19069	-460.32774	-99.746298	-99.818081
BP86-D3/6-311++G(3df, 3pd)	-460.19171	-460.33082	-99.753403	-99.888239
B3LYP / 6-31G	-460.13137	-460.24873	-99.713650	-99.752968
B3LYP-D3/6-311G(d,p)	-460.16616	-460.30071	-99.753809	-99.821336
B3LYP-D3/aug-cc-pVTZ	-460.17533	-460.31052	-99.766141	-99.895852
B3LYP-D3/6-311+G(d,p)	-460.166882	-460.303727	-99.760580	-99.888693
B3LYP-D3/6-311++G(d,p)	-460.166882	-460.303727	-99.760580	-99.888693

Table of computed EA vs experimental EA (eV)

	**Cl**	**eV**	**F**	**eV *(b)***
**EXPERIMENTAL**		Ref 47 **3.61**		Ref 46 **3.41**
M06-2X/aug-cc-pvdz	0.13363	**3.63**	0.117759	**3.18**
M06-2X /aug-cc-pvtz	0.13369	**3.63**	0.121108	**3.27**
M06-2X /aug-cc-pvqz	0.13282	**3.61**	0.120317	**3.25**
M06-2X/6-311G(d,p)	0.13444	**3.63**	0.067718	**1.83**
BP86 / 6-31G	0.12096	**3.29**	0.043015	**1.17**
BP86-D3/ aug-cc-pVTZ	0.13876	**3.75**	0.138155	**3.73**
BP86-D3/6-311G(d,p)	0.13841	**3.74**	0.072737	**1.96**
BP86-D3/6-311+G(d,p)	0.1407	**3.80**	0.135997	**3.67**
BP86-D3/6-311++G(d,p)	0.1407	**3.80**	0.135997	**3.67**
BP86-D3/6-311G(2d,p)	0.13824	**3.73**	0.072529	**1.96**
BP86-D3/6-311G(df,p)	0.13705	**3.70**	0.071783	**1.94**
BP86-D3/6-311++G(3df, 3pd)	0.13911	**3.79**	0.134836	**3.67**
B3LYP / 6-31G	0.11736	**3.19**	0.039318	**1.07**
B3LYP-D3/6-311G(d,p)	0.13455	**3.63**	0.067527	**1.82**
B3LYP-D3/aug-cc-pVTZ	0.13519	**3.65**	0.129711	**3.50**
B3LYP-D3/6-311+G(d,p)	0.136845	**3.72**	0.128113	**3.49**
B3LYP-D3/6-311++G(d,p)	0.136845	**3.72**	0.128113	**3.49**

(Note*: reference 48* lists 6-311++G(3df, 3pd) as the gold standard)

11b) Benchmarking for OH electron affinity

Gas phase: (Experimental EA = 1.83 eV (ref 49,50)

Basis Set	OH^- ground state Total energy	Harmonic Wavenumber (cm^{-1})	Min. energy Bond length	OH^0 opt ground state Total energy (au)	Harmonic Wavenumber (cm^{-1})	Min. energy Bond length	Computed Adiabatic (eV)	Fixed point calculation (ie OH^0 at bond length of OH^-) Energy (au)
BP86-D3/6-311G(d,p)	-75.764596	3393.00	0.98789	-75.750256	3586.94	0.98509	0.3900	-75.750250
BP86-D3/6-311+G(d,p)	-75.830852	3639.56	0.97619	-75.758059	3590.53	0.98578	1.9800	-75.757981
BP86-D3 6-311++G(d,p)	-75.830986	3631.45	0.97664	-75.758140	3591.12	0.98578	1.9814	-75.758068
BP86-D3/6-311G(2d,p)	-75.765411	3409.88	0.98704	-75.751284	3591.16	0.98557	0.3843	-75.751282
BP86-D3/6-311G(df,p)	-75.765145	3386.01	0.98720	-75.751158	3584.11	0.98442	0.3804	-75.751152
BP86-D3/6-311++G(3df, 3pd)	-75.834498	3643.44	0.97488	-75.76203	3596.37	0.98424	1.9711	-75.761955
BP86-D3/ aug-cc-pVTZ	-75.840216	3627.15	0.97597	-75.764207	3576.69	0.98533	2.0674	-75.764133

Basis Set	OH^- ground state Total energy	Harmonic Wavenumber (cm^{-1})	Min. energy Bond length	OH^0 opt ground state Total energy (au)	Harmonic Wavenumber (cm^{-1})	Min. energy Bond length	Computed Adiabatic (eV)	Fixed point calculation (i.e. OH^0 at bond length of OH^-) Energy (au)
BP86-D3/6-311G(d,p)	-75.932945	3619.56	0.97419	-75.758717	3580.00	0.98582	4.7390	-75.758602
BP86-D3/6-311+G(d,p)	-75.980328	3700.48	0.97304	-75.766969	3571.30	0.98731	5.8034	-75.766796
BP86-D3 6-311++G(d,p)	-75.980355	3700.31	0.97305	-75.767053	3571.89	0.98731	5.8018	-75.766881
BP86-D3/6-311G(2d,p)	-75.933464	3623.83	0.97436	-75.759303	3579.70	0.98652	4.7372	-75.759178
BP86-D3/6-311G(df,p)	-75.933482	3617.15	0.97316	-75.759585	3578.07	0.98507	4.7300	-75.759465
BP86-D3/6-311++G(3df, 3pd)	-75.982671	3706.18	0.97124	-75.769848	3574.24	0.98558	5.7888	-75.769672
BP86-D3/ aug-cc-pVTZ	-75.983525	3684.27	0.97214	-75.771805	3556.65	0.98663	5.7588	-75.771627

Computed adiabatic = difference between energies (converted to eV)

Solution phase:

Computed adiabatic = difference between energies (converted to eV)

11c) Justification for the choice of basis set

At the outset, two basis sets were tested for use with the BP86-D3 functional, 6-311G(d,p) and 6-311++G(d,p), for their ability to calculate the known gas-phase electron affinities (EAs) of atomic fluorine (46) and chlorine(47) as these calculated values should provide a measure of the ability of these two levels of calculation to compute reliable relative energies for the reactions considered. Several other basis sets were also tested in this way for use with this functional. The results of these EA calculations are shown in the table below. Inspection of this table shows that all of the selected basis sets, including the 6-311G(d,p) basis set, do quite well in calculating the EA of Cl (all computed values are within 0.2 eV of the experimental value). However, larger differences are observed for the calculated EAs of F. The 6-311G(d,p) basis gives a value which is 1.7 eV too low (at 1.96 eV compared to the experimental value of 3.41 eV) whereas adding extra diffuse functions to give 6-311++G(d,p) gives a value of 3.67 eV, within 0.26 eV of the experimental value. For Pople-style basis sets, 6-311++G(3df,3pd) is considered as the benchmark-quality basis set for thermochemical calculations (48) and the EAs for Cl and F computed with this basis set are the same as the values obtained with the 6-311++G(d,p) basis set. Therefore, it is clear that the BP86-D3/6-311++G(d,p) level of calculation is to be preferred over the BP86-D3/6-311G(d,p) level for calculating the EAs of Cl and F and, by implication, reaction energies involving F^- and Cl^-. EAs of F and Cl calculated with some other basis sets and functional combinations are listed in the Table S11a.

Comparison of Computed and Experimental Electron Affinities (EAs) for Atomic Fluorine and Chlorine, and the OH radical:

	**CHLORINE eV**	**FLUORINE eV**	**OH**
**EXPERIMENTAL**	**3.61 (ref.47)**	**3.41 (ref.46)**	**1.83 eV (refs.(49,50)**
BP86-D3/6-311++G(3df, 3pd)	**3.79**	**3.67**	**1.97**
BP86-D3/6-311++G(d,p)	**3.80**	**3.67**	**1.98**
BP86-D3/6-311G(d,p)	**3.74**	**1.96**	**0.39**
BP86-D3/6-311+G(d,p)	**3.80**	**3.67**	**1.98**
BP86-D3/6-311G(2d,p)	**3.73**	**1.96**	**0.38**
BP86-D3/6-311G(df,p)	**3.70**	**1.94**	**0.38**
BP86-D3/ aug-cc-pVTZ	**3.75**	**3.73**	**2.06**

For OH, the gas-phase adiabatic electron affinity (EA) was computed using all the basis sets listed in Table 1 and the results compared with the experimental value of 1.83 eV (49,50). For each basis set, this was done by computing the total electronic energy at the computed minimum energy of $OH^-(X^1\Sigma^+)$ and the total electronic energy at the computed minimum energy of $OH(X^2\Pi)$ and subtracting the total energies. Also, using the computed vibrational wavenumbers in each state a correction for zero-point energy to the computed EA was made. The results are also shown in section 11b. As was found for F and Cl, the 6-311G(d,p) basis gives a value which is too low (at 0.39 eV compared to the experimental value of 1.83 eV) whereas adding extra diffuse functions to give the basis set 6-311++G(d.p) gives a value of 1.98 eV (0.15 eV above the experimental value). Therefore, all reaction energies and free energies quoted in the text for reactions involving F, Cl and OH and their negative ions will, therefore, be at the BP86-D3/6-311++G(d,p) level.

12. Extra information on the DFT calculations

The functional used in this work BP86-D3 is a GGA functional with the Grimme correction for dispersion. At the outset of the work, we considered other functionals (e.g. M06-2X, a meta hybrid functional, and B3LYP-D3, a hybrid functional with the Grimme correction) and we have used M06-2X as the chosen functional in other studies on smaller systems to those considered here. We chose BP86-D3 on the basis of the likely computational time for each calculation for the molecular sizes and number of stationary points needed to be considered in this work. The paper by Goerigk and Grimme[55] has carried out a thorough benchmarking of a wide range of functionals (47 in total) and has found that BP86-D3 gives comparable results to the M06-2X functional (and the B3LYP-D3 functional) with the same basis sets for basic properties, reaction energies and non-covalent interactions. (See also subsequent paper with additional benchmarking by Grimme and coworkers).[56]

The use of the 2 functionals (BP86-D3 and M06-2X with the same basis set 6-311++G(d,p)) was investigated for the reaction below (in water)

$F^{-} + AlL(Cl) \rightarrow AlL(F) + Cl^{-}$

The same stationary points were located in the M06-2X calculation as found in the BP86-D3 calculation (see Figure 2). The relative energies were similar in both cases, with the M06-2X reaction energy (ΔE) being slightly more negative.

For all reactions and all stationary points located, (including transition states and intermediates) in each case a range of different conformers were considered as starting points in the optimisation procedures. In each case, the same stationary point was obtained with appropriate IRC checks being made.

13. References

8. Shetty, D.; Choi, S. Y.; Jeong, J. M.; Lee, J. Y.; Hoigebazar, L.; Lee, Y.-S.; Lee, D. S.; Chung, J. K.; Lee, M. C.; Chung, Y. K., Stable aluminium fluoride chelates with triazacyclononane derivatives proved by X-ray crystallography and ^{18}F-labeling study. *Chem. Commun.* **47**, 2011, 9732-9734.

27. Bhalla, R.; Levason, W.; Luthra, S. K.; McRobbie, G.; Sanderson, G.; Reid, G., Radiofluorination of a pre-formed Gallium (III) Aza-macrocyclic Complex: Towards next generation Positron Emission Tomography (PET) imaging agents. *Chem. Eur. J.* **21**, 2015, 4688-4694.

44. Bader, R. F. W., A Quantum Theory of Molecular Structure and its Applications. *Chem. Rev.* **91**,1991, 893-926.

45. Rozas, I.; Alkorta, I.; Elguero, J., Behaviour of Ylides Containing N, O and C Atoms as Hydrogen Bond Acceptors. *J. Am. Chem. Soc.* **122**, 2000, 11154-11161.

46. Blondel, C.; Delsart, C.; Goldfarb, F., Electron spectrometry at the μeV level and the electron affinities of Si and F. *J. Physics B*, **34**, 2001, L281-L288.

47. Berzinsh, U.; Gustafsson, M.; Hanstorp, D.; Klinkmuller, A.; Ljungblad, U.; Martensson-Pendrill, A. M., Isotope shift in the electron affinity of chlorine. *Phys. Rev. A*, **51**, 1995, 231-238.

48. Wang, X. J.; Ding, J. H.; Tian, X., Achieving accuracy and economy for calculating vertical detachment energies of molecular anions: A model chemistry composite methods. *ChemPhysChem,* **25**, 2024, e202300642 (5 pages).

49. Celotta, R. J.; Bennett, R. A.; Hall, J. L., Laser photo-detachment determination of the electron affinities of OH, NH_2, NH, SO_2 and S_2. *J. Chem. Phys.* **60**, 1974, 1740-1745.

50. Smith, J. R.; Kim, J. B.; Lineberger, W. C., High-resolution threshold photoelectron spectroscopy of OH^-. *Phys. Rev. A,* **55**, 1997, 2036-2043.

51. Mantina, M.; Chamberlin, A. C.; Valero, R.; Cramer, C. J., Truhlar, D. G., Consistent van der Waals Radii for the whole of the main group, *J. Phys. Chem. A,* **113**, 2009, 5806-5812.

55. Goerigk, L.; Grimme, S., A thorough benchmark of density functional methods for general main group thermochemistry, kinetics, and noncovalent interactions. *Phys. Chem. Chem. Phys.* **13** (*14*), 2011, 6670–6688

56. Goerigk, L.; Hansen, H.; Bauer, C.; Ehrlich, S.; Najibi, A.; Grimme, S., A look at the density functional theory zoo with the advanced GMTKN55 database for general main group thermochemistry, kinetics and noncovalent interactions, *Phys. Chem. Chem. Phys.* **19** (*48*), 2017, 32184–32215.
